# Whole grain consumption and risk of cardiovascular disease, cancer, and all cause and cause specific mortality: systematic review and dose-response meta-analysis of prospective studies

**DOI:** 10.1136/bmj.i2716

**Published:** 2016-06-14

**Authors:** Dagfinn Aune, NaNa Keum, Edward Giovannucci, Lars T Fadnes, Paolo Boffetta, Darren C Greenwood, Serena Tonstad, Lars J Vatten, Elio Riboli, Teresa Norat

**Affiliations:** 1 Department of Public Health and General Practice, Faculty of Medicine, Norwegian University of Science and Technology, Trondheim, Norway; 2 Department of Epidemiology and Biostatistics, School of Public Health, Imperial College London, London, UK; 3 Department of Nutrition, Harvard T H Chan School of Public Health, Boston, MA, USA; 4 Department of Epidemiology, Harvard T H Chan School of Public Health, Boston, MA, USA; 5 Channing Division of Network Medicine, Department of Medicine, Brigham and Women’s Hospital and Harvard Medical School, Boston, MA, USA; 6 Centre for International Health, Department of Global Public Health and Primary Care and Department of Clinical Dentistry, University of Bergen, Bergen, Norway; 7 The Tisch Cancer Institute, Icahn School of Medicine at Mount Sinai, New York, NY, USA; 8 Biostatistics Unit, Centre for Epidemiology and Biostatistics, University of Leeds, Leeds, UK; 9 Section of Preventive Cardiology, Department of Endocrinology, Morbid Obesity and Preventive Medicine, Oslo University Hospital Ullevål, Oslo, Norway

## Abstract

**Objective** To quantify the dose-response relation between consumption of whole grain and specific types of grains and the risk of cardiovascular disease, total cancer, and all cause and cause specific mortality.

**Data sources** PubMed and Embase searched up to 3 April 2016.

**Study selection** Prospective studies reporting adjusted relative risk estimates for the association between intake of whole grains or specific types of grains and cardiovascular disease, total cancer, all cause or cause specific mortality.

**Data synthesis** Summary relative risks and 95% confidence intervals calculated with a random effects model.

**Results** 45 studies (64 publications) were included. The summary relative risks per 90 g/day increase in whole grain intake (90 g is equivalent to three servings—for example, two slices of bread and one bowl of cereal or one and a half pieces of pita bread made from whole grains) was 0.81 (95% confidence interval 0.75 to 0.87; I^2^=9%, n=7 studies) for coronary heart disease, 0.88 (0.75 to 1.03; I^2^=56%, n=6) for stroke, and 0.78 (0.73 to 0.85; I^2^=40%, n=10) for cardiovascular disease, with similar results when studies were stratified by whether the outcome was incidence or mortality. The relative risks for morality were 0.85 (0.80 to 0.91; I^2^=37%, n=6) for total cancer, 0.83 (0.77 to 0.90; I^2^=83%, n=11) for all causes, 0.78 (0.70 to 0.87; I^2^=0%, n=4) for respiratory disease, 0.49 (0.23 to 1.05; I^2^=85%, n=4) for diabetes, 0.74 (0.56 to 0.96; I^2^=0%, n=3) for infectious diseases, 1.15 (0.66 to 2.02; I^2^=79%, n=2) for diseases of the nervous system disease, and 0.78 (0.75 to 0.82; I^2^=0%, n=5) for all non-cardiovascular, non-cancer causes. Reductions in risk were observed up to an intake of 210-225 g/day (seven to seven and a half servings per day) for most of the outcomes. Intakes of specific types of whole grains including whole grain bread, whole grain breakfast cereals, and added bran, as well as total bread and total breakfast cereals were also associated with reduced risks of cardiovascular disease and/or all cause mortality, but there was little evidence of an association with refined grains, white rice, total rice, or total grains.

**Conclusions** This meta-analysis provides further evidence that whole grain intake is associated with a reduced risk of coronary heart disease, cardiovascular disease, and total cancer, and mortality from all causes, respiratory diseases, infectious diseases, diabetes, and all non-cardiovascular, non-cancer causes. These findings support dietary guidelines that recommend increased intake of whole grain to reduce the risk of chronic diseases and premature mortality.

## Introduction

Cardiovascular disease and cancer remain the two most common causes of death and in 2013 accounted for 25.5 million deaths worldwide.^1^ Grains are one of the major staple foods consumed around the world and provide 56% of the energy and 50% of the protein intake. They constitute the largest component of recommended daily intake in all dietary guidelines.^2^ Because of their important role in most diets around the world, interest in the health effects of grain consumption, and in particular whole grains, is increasing.^3^ ^4^ A high intake of whole grains has been associated with a reduced risk of type 2 diabetes,^5^ coronary heart disease,^6^ and obesity.^6^

Whole grains contain endosperm, germ, and bran, in contrast with refined grains, which have the germ and bran removed during the milling process. Whole grains are good sources of fibre, B vitamins, and some trace minerals such as iron, magnesium, and zinc.^7^ These nutrients are found in the outer layer of the grains or the bran that function as a protective shell for the germ and endosperm inside. The germ is nourishment for the seed and contains antioxidants, vitamin E, and some B vitamins, while the endosperm provides carbohydrates, protein, and energy.^7^ Consumption of whole grains differs considerably between populations,^8^ with the main source being whole grain bread in Scandinavian countries,^9^ whole grain bread and breakfast cereals in the United States,^10^ brown rice, unrefined maize and sorghum in some African countries,^11^ and brown rice in Asia,^12^ although most of the rice consumed in Asia is white rice.^13^ ^14^

Several previous prospective studies have found a lower risk of coronary heart disease,^4^ ^9^ ^15^ ^16^ ^17^ stroke,^16^ ^18^ cardiovascular disease,^4^ ^19^ ^20^ and all cause mortality^4^ ^9^ ^16^ ^20^ ^21^ ^22^ associated with a high intake of whole grains, though not all studies reported a clear association.^23^ ^24^ ^25^ ^26^ ^27^ We have previously reported an inverse association between dietary fibre and whole grain intake and risk of colorectal cancer,^28^ and a previous review of mostly case-control studies reported a lower risk of several individual cancers, mainly of the digestive system, with higher intake of whole grains,^3^ but data from cohort studies are limited. Whether whole grain consumption is associated with risk of total cancer is not clear, and clarification of this question would be important from a public health point of view. Epidemiological studies on whole grains and total cancer, however, have reported mixed results, with some studies suggesting a possible inverse association,^4^ ^9^ ^22^ ^29^ while others have shown no clear association.^20^ ^27^ Of the cohort studies on whole grains and cardiovascular disease or all cause mortality, some^16^ ^20^ ^22^ but not all^4^ ^15^ ^17^ ^21^ ^29^ studies reported a possible plateau effect, with most of the benefit observed at relatively low levels of intake. Although two previous meta-analyses suggested an inverse association between high versus low intake of whole grains and coronary heart disease,^6^ ^30^ no dose-response analyses were conducted, thus questions remain about the strength and shape of the dose-response relation between whole grains and coronary heart disease and the amount of whole grains that need to be eaten to reduce risk of coronary heart disease and other chronic diseases. Whole grain intake has also been inversely associated with other less common causes of death including deaths from infection,^4^ ^20^ ^22^ respiratory disease,^4^ ^9^ ^20^ ^22^ diabetes,^9^ ^20^ ^22^ and kidney disease^20^ in some studies, but the available data are limited.

Despite a growing body of epidemiological evidence for the health benefits of whole grain consumption, dietary recommendations have often been unclear or inconsistent with regard to the amount of whole grains that should be eaten to reduce the risk of chronic disease. For example the World Cancer Research Fund 2007 report recommended that people should “eat relatively unprocessed cereals (grains) and/or pulses with every meal,”^31^ while in the United Kingdom there is no specific recommendation other than “choosing whole grain, brown or high fibre varieties wherever you can,” but no specific quantities of whole grains were recommended.^32^ In the US and Canada the recommendation is that “all adults eat at least half their grains as whole grains” so at least three servings of whole grains should be consumed each day,^33^ while in Scandinavian countries intake of at least 75 g per day of whole grain (dry weight), which equals about 250 g a day (eight servings/day) of whole grain products (fresh weight), is recommended.^34^ There might be several reasons for the inconsistent dietary guidelines for whole grain intake, including difficulties in measuring intake, differences in the consumption patterns between populations, or lack of data on intake in some populations, but it might also be because most previous meta-analyses considered only selected disease endpoints and did not conduct dose-response analyses.^6^ ^30^ We found a reduced risk of incidence of type 2 diabetes associated with up to two to three servings a day (60-90 g/day) of whole grain but no further reductions in risk with higher intakes,^5^ while in a second meta-analysis of whole grain intake and colorectal cancer a linear inverse association was observed with intakes of up to 180 g/day.^28^ Whether the association is linear or reaches a plateau for other chronic disease outcomes and all cause mortality, or whether only specific types of whole grains are associated with chronic disease and all cause mortality, would be important to clarify to provide more detailed and consistent dietary recommendations with regard to the amount and types of whole grains that should be consumed to reduce the risk of chronic disease and premature mortality. Answering this question would also clarify whether there are additional benefits with high intakes such as those recommended in the Scandinavian guidelines^34^ and whether such high recommendations are justified. Several large cohort studies including more than 22 000 cases of cardiovascular disease and more than 662 000 participants^9^ ^20^ ^22^ ^27^ ^35^ have been published since or were missed^36^ by the previous meta-analyses of whole grains and cardiovascular disease.^6^ ^30^

To provide a more comprehensive, up to date, and detailed assessment of whole grain intake and several health outcomes we conducted a systematic review and meta-analysis of whole grain consumption in relation to coronary heart disease, stroke, cardiovascular disease, and total cancer and all cause mortality, as well as less common causes of mortality including respiratory disease, infectious disease, diabetes, neurological disease, and all non-cardiovascular, non-cancer causes combined. We aimed to clarify the strength and the shape of the dose-response relation between whole grain intake and these outcomes. We also summarised data on specific types of whole grains as well as on refined grains and total grains. Because of the limited amount of data, however, the main focus of our current analysis is on whole grains.

## Methods

### Search strategy and inclusion criteria

We searched the PubMed and Embase databases from their inception (1966 and 1947, respectively) to 31 May 2014 and later updated the search to 3 April 2016. Details of the search terms are provided in table S1 in appendix 1. We included prospective studies of grain intake and incidence or mortality from coronary heart disease, stroke, cardiovascular disease, total cancer, and all cause and cause specific mortality if they reported adjusted relative risk estimates and 95% confidence intervals. For the dose-response analyses a quantitative measure of the intake for at least three categories of grain intake or a risk estimate for grain intake on a continuous scale had to be available. We searched the references of the retrieved reports for any additional studies. A list of the excluded studies is provided in table S2 in appendix 1. We followed standard criteria (PRISMA criteria) for reporting meta-analyses.^37^ The authors of one study^22^ were contacted for clarification of the amount of whole grain intake, which was reported in ounces/day (1 ounce=28 g) in the publication but was clarified to be in ounces/1000 kcal/day by the authors. In another study,^38^ the authors were contacted for clarification of the increment for bread which was clarified to be 100 g/d rather than 10 g/d.

### Patient involvement

No patients were involved in setting the research question or the outcome measures, nor were they involved in developing plans for design, or implementation of the study. No patients were asked to advise on interpretation or writing up of results. There are no plans to disseminate the results of the research to study participants or the relevant patient community.

### Data extraction

From each study we extracted name of first author, publication year, country, name of the study, follow-up period, sample size and number of cases or deaths, type of outcome, sex, age, type of grains, amount or frequency of intake, relative risks and 95% confidence intervals, and variables adjusted for in the analysis. Data were extracted by one author (DA) and checked by another author (DCG) for accuracy.

### Statistical methods

We calculated summary relative risks of cardiovascular disease, total cancer, and mortality for the highest versus the lowest level of intake and for 90 g a day increment (three servings/day; the approximate median range across studies) using the random effects model by DerSimonian and Laird,^39^ which takes into account variation (heterogeneity) both within and between studies. The average of the natural logarithm of the relative risks was estimated and the relative risk from each study was weighted with random effects weights. When studies reported data separately by sex we pooled the relative risks using a fixed effects model before inclusion in the meta-analysis. A two tailed P<0.05 was considered significant.

We conducted linear dose-response analyses using the method by Greenland and Longnecker^40^ to compute study specific slopes (linear trends) and 95% confidence intervals from the natural logarithm of the relative risks across categories of grain intake. For each category of grain intake we used the mean or median if it was reported in the publication and estimated the midpoint of the upper and lower bound for the remaining studies. When extreme categories were open ended or had extreme upper or lower values we used the width of the adjacent interval to calculate an upper or lower cut-off value. For total grains, whole grains, and refined grains we used 30 g as a serving size (one slice of bread or one bowl of breakfast cereal) to recalculate results from studies reporting data in g/day to servings/day, as in our previous analyses.^5^ ^28^ For intake of pasta we used 150 g as a serving size while for total rice we used 167.25 g as a serving size (cooked weight) based on a weighted average of the serving size for white rice (158 g/day) and brown rice (195 g/day), weighted by the proportion of rice intake of each type (75% white rice and 25% brown rice),^41^ unless a serving size was specified in the paper. Separate analyses were conducted for studies reporting on total whole grains and specific subtypes of whole grains. We assessed a potential non-linear dose-response relation between grain intake and cardiovascular disease, cancer, and all cause and cause specific mortality using restricted cubic splines with three knots at 10%, 50%, and 90% centiles of the distribution, which were combined using multivariate meta-analysis.^42^ ^43^ The 95% confidence intervals were derived from the standard errors of the differences in linear predictors between each given point on the dose-response curve and a stated reference value, computed from the covariate values and the covariance matrix of the estimated coefficients.^44^ A likelihood ratio test was used to assess the difference between the non-linear and linear models to test for non-linearity.^45^

Heterogeneity between studies was evaluated with Q and I^2^ statistics.^46^ For the Q statistic a P<0.10 was considered to be significant. I^2^ is the amount of total variation explained by variation between studies. We carried out subgroup and meta-regression analyses stratified by study characteristics (duration of follow-up, sex, geographical location, number of cases, whether the method of dietary assessment had been validated, study quality, and adjustment for confounding factors) to investigate potential sources of heterogeneity. Influence analyses in which we excluded one study at a time from each analysis were conducted to investigate the robustness of the findings. We assessed publication bias with Egger’s test^47^ and have provided funnel plots in analyses including 10 or more studies. Study quality was assessed with the Newcastle-Ottawa scale, which awards 0-9 points based on the selection, comparability, and outcome assessment.^48^ We considered studies with 0-3, 4-6, and 7-9 points to represent low, medium, and high quality studies, respectively. Stata version 12.0 software (StataCorp, TX) was used for the analyses.

## Results

We included 45 cohort studies (64 publications)^4^ ^9^ ^15^ ^16^ ^17^ ^18^ ^19^ ^20^ ^21^ ^22^ ^23^ ^24^ ^25^ ^26^ ^27^ ^29^ ^35^ ^36^ ^41^ ^38^ ^49^ ^50^ ^51^ ^52^ ^53^ ^54^ ^55^ ^56^ ^57^ ^58^ ^59^ ^60^ ^61^ ^62^ ^63^ ^64^ ^65^ ^66^ ^67^ ^68^ ^69^ ^70^ ^71^ ^72^ ^73^ ^74^ ^75^ ^76^ ^77^ ^78^ ^79^ ^80^ ^81^ ^82^ ^83^ ^84^ ^85^ ^86^ ^87^ ^88^ ^89^ ^90^ ^91^ ^92^ in the analyses of grain intake and coronary heart disease, stroke, cardiovascular disease, total cancer, and all cause mortality and other causes of mortality (table S3-S12 in appendix 1, fig 1). Twenty studies were from Europe, 16 were from the US, and nine were from Asia. The studies included in the analyses of whole grains included 7068 cases of coronary heart disease, 2337 cases of stroke, 26 243 cases of cardiovascular disease, 34 346 deaths from cancer, and 100 726 all cause deaths. The number of participants ranged from 245 012 to 705 253. Tables S3-S12 in appendix 1 provide a summary of the study characteristics. Figure 1 shows a flowchart of the study selection. Figures S1-S20 in appendix 2 show the results from the high versus low analyses and scatter plots from the non-linear dose-response analyses. Figures S21-S102 in appendix 2 show results for specific types of grains, refined grains, and total grains.

**Figure f1:**
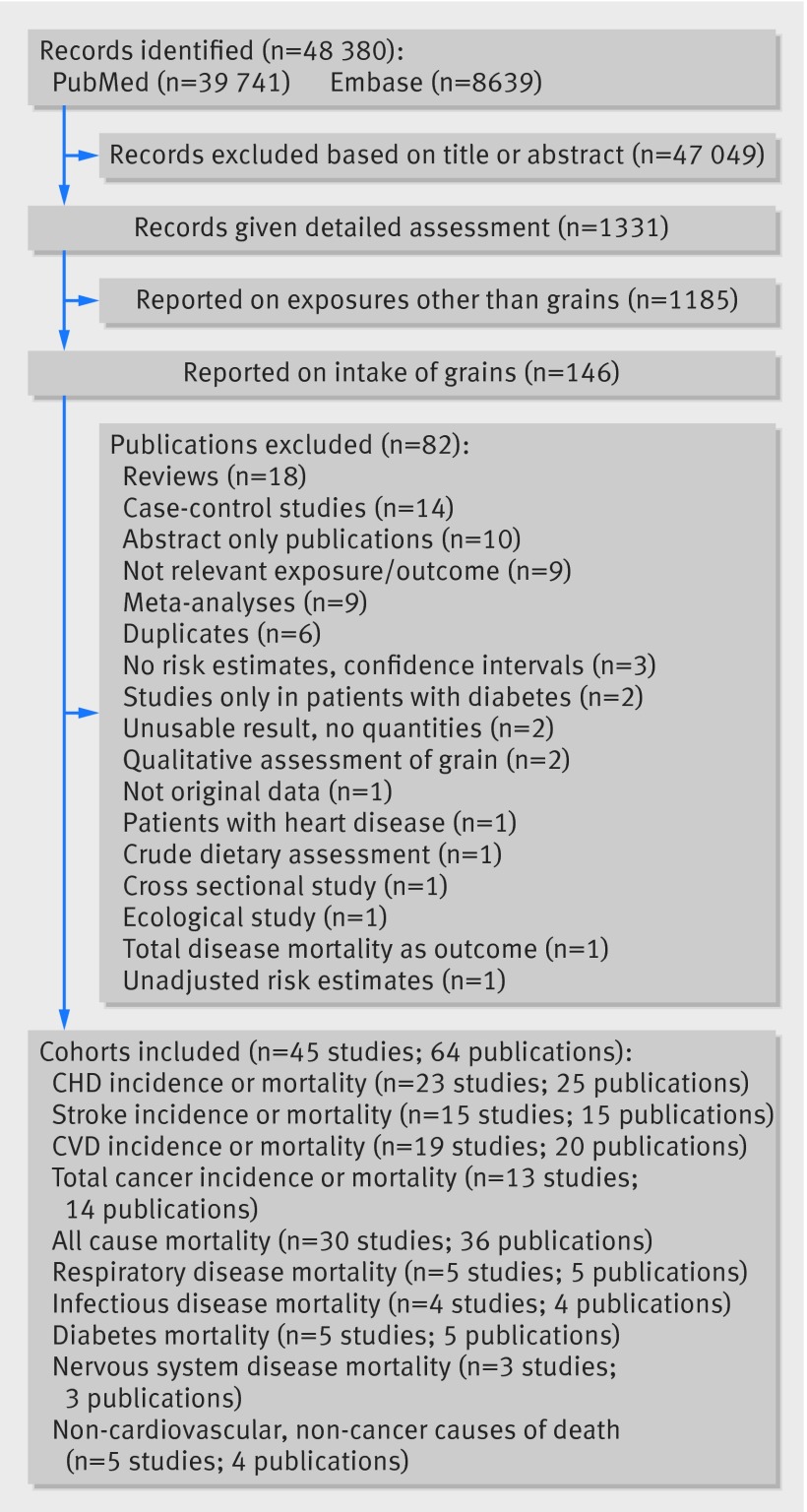
**Fig 1** Flow chart of study selection

### Whole grains and coronary heart disease

Seven cohort studies^4^ ^9^ ^15^ ^16^ ^17^ ^23^ ^63^ investigated the association between whole grain intake and risk of coronary heart disease and included a total of 7068 cases and 316 491 participants (one additional publication was included only in the subgroup analysis of incidence of coronary heart disease^64^ as it overlapped with another publication^9^). The summary relative risk for high versus low intake was 0.79 (95% confidence interval 0.73 to 0.86; I^2^=0%, P_heterogeneity_=0.63) (fig S1 in appendix 2, table 1). The summary relative risk per 90 g/day was 0.81 (0.75 to 0.87; I^2^=9%, P_heterogeneity_=0.36) (fig 2, table 1). Although the test for non-linearity was significant for the association between whole grain intake and coronary heart disease (P<0.001), with a slightly steeper reduction in risk up to three servings a day than more than three servings a day, there was a clear dose-response relation, and there were further reductions in risk up to 210 g/day (fig 2, fig S2 in appendix 2, table S13 in appendix 1).

**Table 1 tbl1:** Intake of total whole grains and effect on coronary heart disease, stroke, cardiovascular disease, total cancer, all cause mortality, and cause specific mortality. Analysis of low versus high intake and dose-response analysis

	High *v* low analysis					Dose-response analysis				
	No of studies	RR* (95% CI)	I^2^	P value†		Dose (g/day)	No of studies	RR* (95% CI)	I^2^	P value†
**Incidence**										
Coronary heart disease	5	0.80 (0.74 to 0.87)	0	0.62		90	5	0.84 (0.77 to 0.92)	34	0.20
Stroke	3	0.86 (0.60 to 1.20)	65	0.06		90	3	0.84 (0.59 to 1.20)	74	0.02
Cardiovascular disease	2	0.89 (0.81 to 0.99)	0	0.40		90	2	0.87 (0.78 to 0.97)	0	0.85
**Incidence or mortality**										
Coronary heart disease	6	0.79 (0.73 to 0.86)	0	0.63		90	7	0.81 (0.75 to 0.87)	9	0.36
Stroke	5	0.87 (0.72 to 1.05)	32	0.21		90	6	0.88 (0.75 to 1.03)	56	0.04
Cardiovascular disease	9	0.84 (0.80 to 0.87)	0	0.48		90	10	0.78 (0.73 to 0.85)	40	0.09
**Mortality**										
Coronary heart disease	2	0.65 (0.52 to 0.83)	33	0.22		90	3	0.81 (0.74 to 0.89)	10	0.33
Stroke	2	0.85 (0.64 to 1.13)	0	0.99		90	3	0.86 (0.74 to 0.99)	34	0.20
Cardiovascular disease	7	0.81 (0.75 to 0.87)	37	0.15		90	8	0.71 (0.61 to 0.82)	72	0.001
Total cancer	6	0.89 (0.82 to 0.96)	72	0.003		90	6	0.85 (0.80 to 0.91)	37	0.16
All cause	9	0.82 (0.77 to 0.88)	83	<0.001		90	11	0.83 (0.77 to 0.90)	83	<0.001
Respiratory disease	4	0.81 (0.69 to 0.94)	63	0.05		90	4	0.78 (0.70 to 0.87)	0	0.46
Diabetes	4	0.64 (0.42 to 0.98)	64	0.04		90	4	0.49 (0.23 to 1.05)	85	<0.001
Infectious disease	3	0.80 (0.68 to 0.96)	0	0.68		90	3	0.74 (0.56 to 0.96)	0	0.85
Nervous system disease	2	1.13 (0.89 to 1.43)	29	0.24		90	2	1.15 (0.66 to 2.02)	79	0.03
Non-cardiovascular, non-cancer causes	5	0.79 (0.69 to 0.92)	86	<0.001		90	5	0.78 (0.75 to 0.82)	0	0.99

*RR<1 favours those with higher intake

†P for heterogeneity.

**Figure f2:**
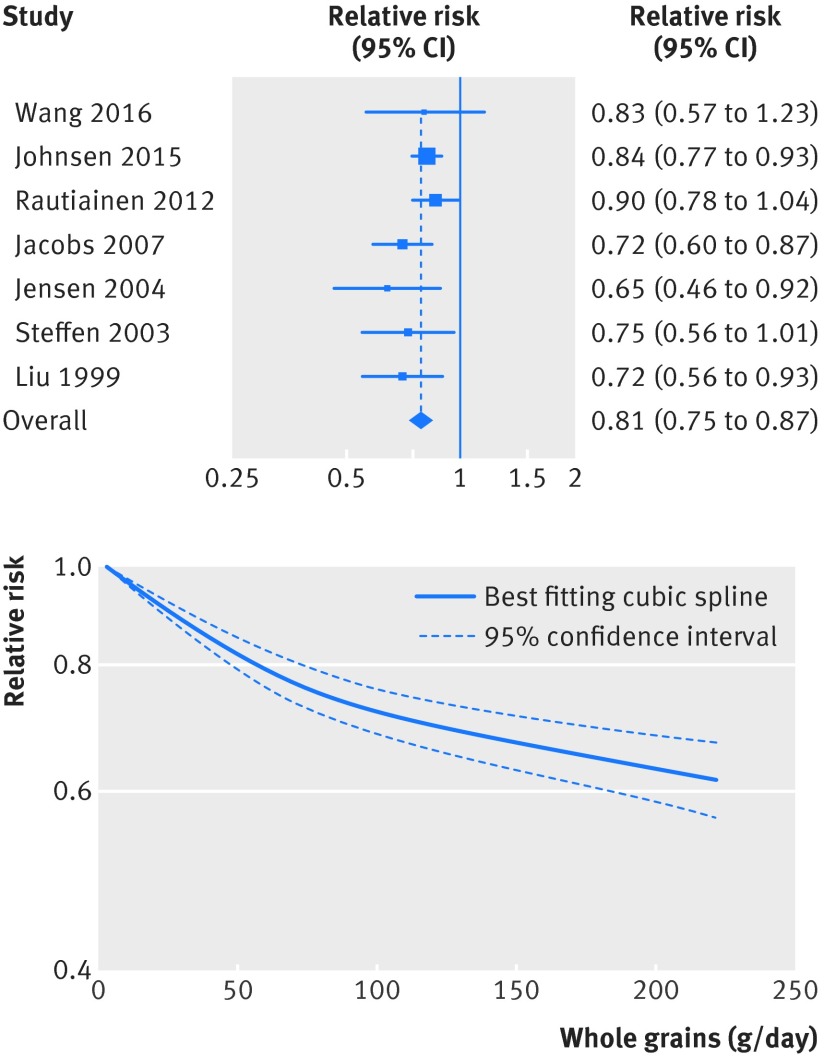
**Fig 2** Forest plot for consumption of whole grains (per 90 g/day) and risk of coronary heart disease, with graph illustrating non-linear response

Subtypes of whole grains including whole grain bread,^9^ ^15^ ^29^ ^51^ ^52^ ^60^ ^62^ whole grain breakfast cereals,^9^ ^15^ ^51^ ^53^ and added bran^15^ ^17^ ^54^ were inversely associated with coronary heart disease, but no association was observed for germ,^15^ ^17^ refined grains,^4^ ^16^ ^58^ ^63^ white bread,^51^ ^60^ refined grain breakfast cereals,^51^ ^53^ total rice,^41^ ^56^ ^59^ or total grains,^55^ ^61^ ^62^ ^63^ while rye was only inversely associated in the high versus low analysis and not in the dose-response analysis (figs S21-S44 in appendix 2, table 2).^9^ ^49^ ^57^

**Table 2 tbl2:** Intakes of subtypes of grains and effect on coronary heart disease, stroke, cardiovascular disease, cancer, and mortality. Analysis of high versus low intake and dose-response analysis

Type of grain	High *v* low analysis					Dose-response analysis				
	No of studies	RR* (95% CI)	I^2^	P value†		Dose (g/day)	No of studies	RR* (95% CI)	I^2^	P value†
**Coronary heart disease**										
Whole grain bread	7	0.83 (0.75 to 0.92)	0	0.64		90	5	0.83 (0.76 to 0.92)	0	0.53
Whole grain breakfast cereals	4	0.72 (0.64 to 0.82)	0	0.92		30	4	0.81 (0.75 to 0.88)	0	0.69
Rye products	2	0.81 (0.70 to 0.94)	0	0.47		30	2	0.97 (0.91 to 1.05)	54	0.14
Added bran	3	0.78 (0.63 to 0.95)	65	0.06		10	2	0.72 (0.58 to 0.89)	34	0.22
Germ	2	0.73 (0.33 to 1.64)	65	0.09		2	2	0.88 (0.76 to 1.03)	0	0.65
Refined grains	4	1.16 (0.84 to 1.59)	48	0.12		90	5	1.13 (0.90 to 1.42)	57	0.05
White bread	2	1.07 (0.86 to 1.34)	50	0.16		90	2	0.96 (0.53 to 1.76)	86	0.007
Refined grain breakfast cereals	2	1.15 (0.79 to 1.67)	70	0.07		30	2	1.14 (0.75 to 1.73)	72	0.06
Total rice	4	0.98 (0.90 to 1.07)	0	0.44		100	4	0.99 (0.95 to 1.03)	7	0.36
Total grains	3	1.07 (0.91 to 1.25)	0	0.47		90	2	1.07 (0.88 to 1.30)	0	0.40
**Stroke**										
Whole grain bread	2	0.88 (0.75 to 1.03)	0	0.89		90	1	0.88 (0.72 to 1.07)	—	—
Whole grain breakfast cereals	2	0.99 (0.53 to 1.86)	77	0.04		30	2	1.07 (0.69 to 1.64)	78	0.03
Refined grains	4	0.95 (0.78 to 1.14)	23	0.28		90	5	0.91 (0.81 to 1.02)	29	0.23
Total rice	4	1.02 (0.94 to 1.11)	0	0.95		100	4	1.00 (0.97 to 1.03)	0	0.87
Total grains	4	0.89 (0.79 to 1.00)	6	0.36		90	5	0.93 (0.85 to 1.02)	62	0.03
**Cardiovascular disease**										
Whole grain bread	4	0.83 (0.75 to 0.92)	0	0.78		90	3	0.87 (0.80 to 0.95)	0	0.71
Whole grain breakfast cereals	2	0.74 (0.65 to 0.84)	4	0.31		30	2	0.84 (0.78 to 0.90)	0	0.82
Bran	3	0.82 (0.76 to 0.88)	0	0.64		10	2	0.85 (0.79 to 0.90)	0	0.37
Germ	2	1.06 (0.97 to 1.16)	0	0.41		2	2	1.05 (0.96 to 1.15)	0	0.41
Refined grains	2	1.02 (0.91 to 1.14)	16	0.27		90	3	0.98 (0.90 to 1.06)	56	0.11
Total breakfast cereals	2	0.80 (0.70 to 0.90)	55	0.14		30	3	0.80 (0.68 to 0.93)	73	0.03
Total rice	3	0.96 (0.90 to 1.03)	0	0.54		100	3	0.98 (0.95 to 1.00)	0	0.47
Total grains	3	0.94 (0.84 to 1.06)	0	0.47		90	1	0.83 (0.70 to 1.00)	—	—
**Total cancer**										
Whole grain bread	3	0.89 (0.78 to 1.01)	42	0.18		90	3	0.91 (0.85 to 0.96)	0	0.63
Brown rice	3	1.07 (0.91 to 1.26)	27	0.26		100	3	0.98 (0.92 to 1.04)	0	0.61
Refined grains	1	0.98 (0.82 to 1.16)	—	—		90	2	0.94 (0.90 to 0.99)	0	0.60
White rice	3	0.87 (0.76 to 1.01)	53	0.12		100	3	0.98 (0.92 to 1.05)	49	0.14
Total breakfast cereals	1	0.90 (0.86 to 0.95)	—	—		30	2	0.90 (0.82 to 1.00)	36	0.21
Total rice	4	0.95 (0.88 to 1.02)	65	0.03		100	4	0.98 (0.95 to 1.01)	55	0.08
Total grains	1	0.92 (0.80 to 1.06)	—	—		90	2	0.97 (0.96 to 0.99)	0	0.51
**All cause mortality**										
Whole grain bread	5	0.81 (0.74 to 0.88)	57	0.05		90	2	0.85 (0.82 to 0.89)	0	0.36
Whole grain breakfast cereals	3	0.79 (0.72 to 0.86)	50	0.14		30	2	0.87 (0.84 to 0.90)	0	0.85
Oats or oatmeal	3	0.89 (0.76 to 1.04)	90	<0.001		20	1	0.88 (0.83 to 0.92)	—	—
Refined grains	2	1.02 (0.93 to 1.12)	0	0.64		90	4	0.95 (0.91 to 0.99)	20	0.29
Pasta	1	0.61 (0.26 to 1.45)	—	—		150	2	0.85 (0.74 to 0.99)	54	0.14
Total bread	3	0.77 (0.72 to 0.81)	0	0.42		90	3	0.83 (0.80 to 0.85)	0	0.41
Total breakfast cereals	2	0.87 (0.81 to 0.93)	47	0.17		30	3	0.89 (0.83 to 0.96)	92	<0.001
Total grains	13	0.91 (0.87 to 0.95)	4	0.41		90	7	0.96 (0.90 to 1.02)	71	0.002

*RR<1 favours those with higher intake.

†P for heterogeneity.

### Whole grains and stroke

Six cohort studies^4^ ^9^ ^16^ ^18^ ^24^ ^63^ were included in the analysis of whole grain intake and risk of stroke and included 2337 cases and 245 012 participants. The pooled relative risk for high versus low intake was 0.87 (95% confidence interval 0.72 to 1.05; I^2^=32%, P_heterogeneity_=0.21 (fig S3 in appendix 2, table 1). The summary relative risk per 90 g/day was 0.88 (0.75 to 1.03; I^2^=56%, P_heterogeneity_=0.04) (fig 3, table 1). There was evidence of non-linearity between whole grain and risk of stroke (P<0.001), and there was no further reduction in risk above 120-150 g/day (fig 3, fig S4 in appendix 2, table S13 in appendix 1).

**Figure f3:**
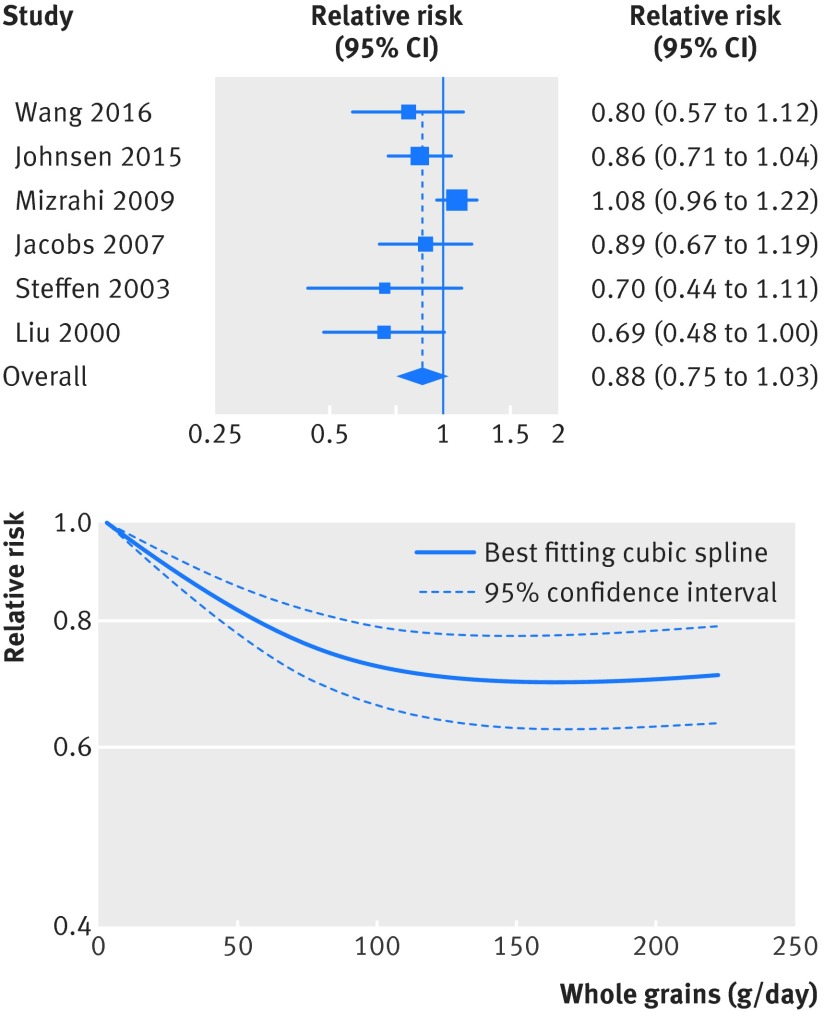
**Fig 3** Forest plot for consumption of whole grains (per 90 g/day) and risk of stroke, with graph illustrating non-linear response

We found no clear association between intake of whole grain bread,^9^ ^52^ whole grain breakfast cereals,^9^ ^53^ refined grains,^4^ ^16^ ^18^ ^24^ ^63^ total rice,^41^ ^56^ ^59^ ^68^ or total grains^18^ ^24^ ^61^ ^63^ ^67^ ^69^ and risk of stroke (figs S45-S56 in appendix 2, table 2).

### Whole grains and cardiovascular disease

Ten cohort studies (nine publications)^4^ ^9^ ^19^ ^20^ ^21^ ^22^ ^26^ ^27^ ^63^ investigated whole grain intake and risk of cardiovascular disease and included 26 243 cases and 704 317 participants. The summary relative risk for high versus low intake was 0.84 (95% confidence interval 0.80 to 0.87; I^2^=0%, P_heterogeneity_=0.48) (fig S5 in appendix 2, table 1). The summary relative risk was 0.78 (0.73 to 0.85; I^2^=40%, P_heterogeneity_=0.09) per 90 g/day (fig 4, table 1). There was evidence of a non-linear association between whole grain intake and risk of cardiovascular disease (P<0.001), with a stronger reduction in risk from no intake up to 50 g/day than with higher intakes, but with slight further reductions in risk with intakes up to 200 g/day (fig 4, fig S6 in appendix 2, table S13 in appendix 1).

**Figure f4:**
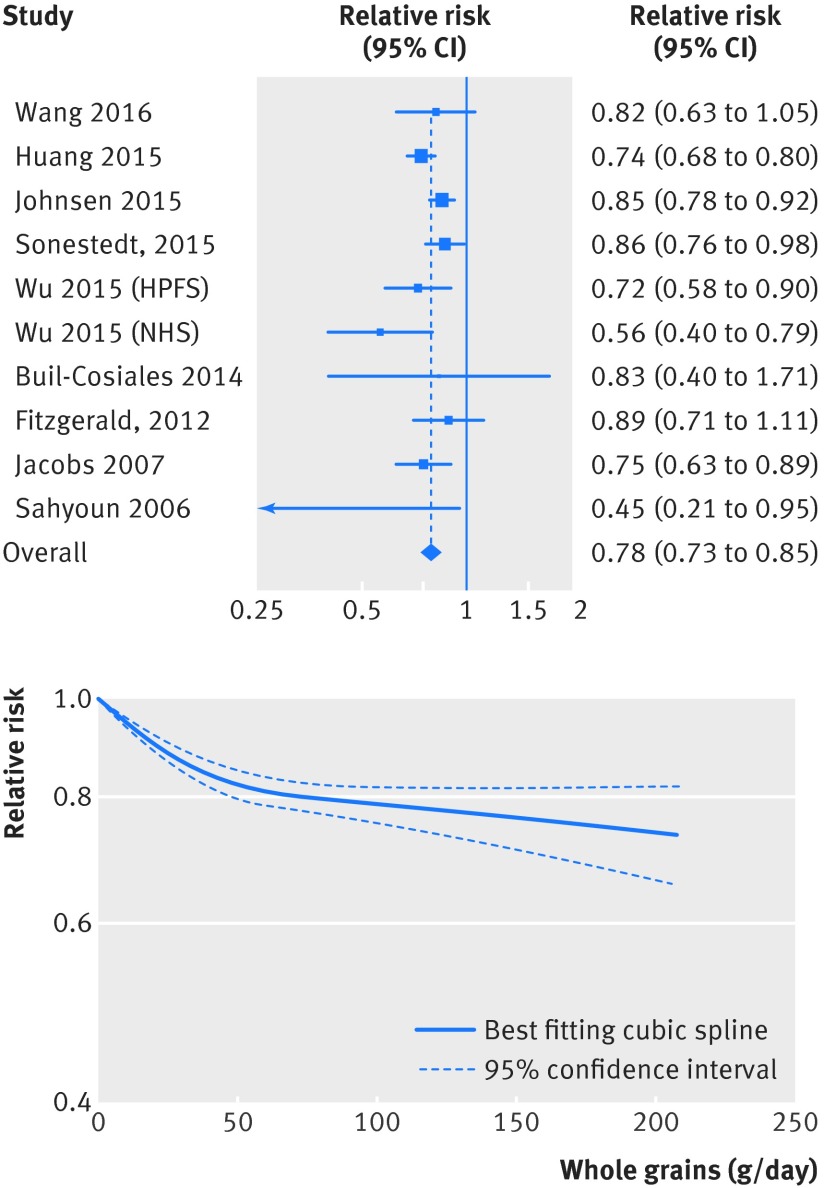
**Fig 4** Forest plot for consumption of whole grains (per 90 g/day) and risk of cardiovascular disease, with graph illustrating non-linear response

Intake of whole grain bread,^9^ ^29^ ^36^ ^52^ ^62^ whole grain breakfast cereals,^9^ ^53^ total breakfast cereals,^36^ ^53^ ^71^ and bran,^20^ ^54^ but not germ,^20^ refined grains,^4^ ^20^ ^35^ ^63^ total rice,^41^ ^56^ ^59^ or total grains,^61^ ^62^ ^70^ were inversely associated with the risk of cardiovascular disease (figs S57-S72 in appendix 2, table 2).

### Whole grains and total cancer

Six cohort studies (five publications)^4^ ^9^ ^20^ ^22^ ^27^ were included in the analysis of whole grain intake and risk of total cancer and included 34 346 deaths from cancer among 640 065 participants. The summary relative risk for the high versus the low intake was 0.89 (95% confidence interval 0.82 to 0.96; I^2^=72%, P_heterogeneity_=0.003) (fig S7 in appendix 2, table 1). The summary relative risk per 90 g/day was 0.85 (0.80 to 0.91; I^2^=37%, P_heterogeneity_=0.16) (fig 5, table 1). The heterogeneity seemed to be explained by one large US study,^22^ and when this was excluded there was no evidence of heterogeneity (I^2^=0%, P=0.74) and the association remained similar (summary relative risk 0.87, 0.83 to 0.92). There was no evidence of a non-linear association between whole grain intake and total cancer (P=0.15; fig 5 fig S8 in appendix 2, table S13 in appendix 1).

**Figure f5:**
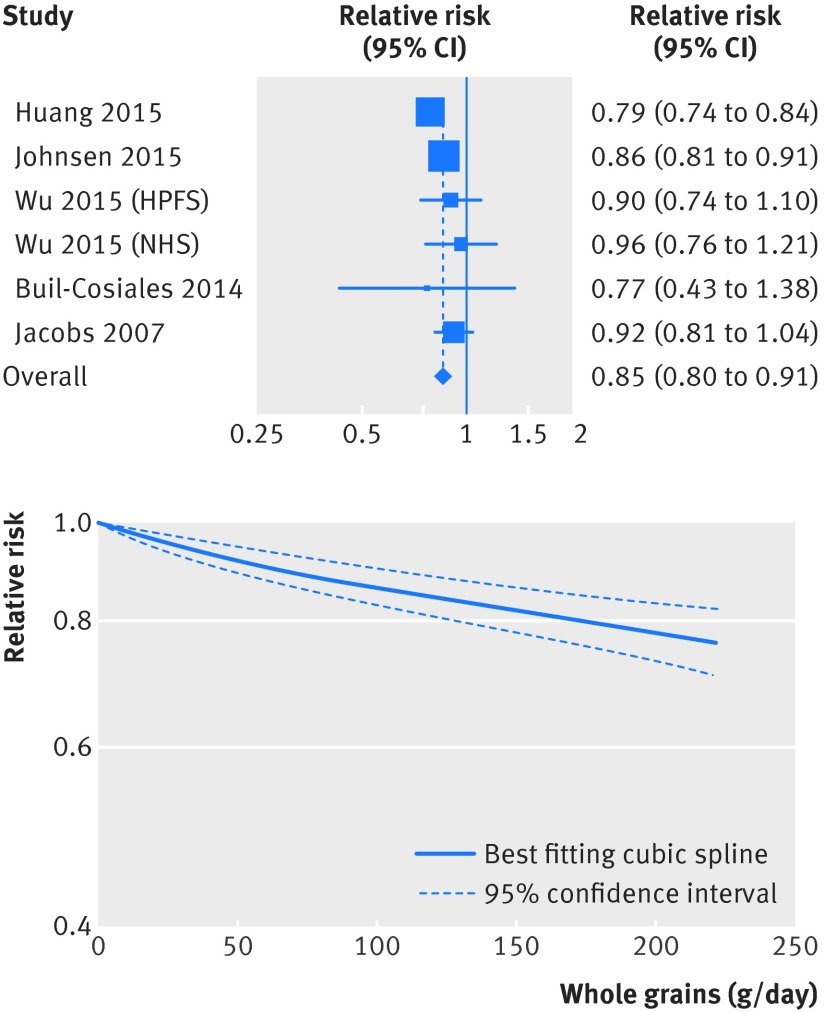
**Fig 5** Forest plot for consumption of whole grains (per 90 g/day) and risk of total cancer, with graph illustrating non-linear response

There was an inverse association between intake of whole grain bread^9^ ^29^ ^36^ ^52^ and total cancer, and there were weak inverse associations between intake of refined grains,^4^ ^20^ and total grains^74^ ^75^ and total cancer in the dose-response analysis, but no association was observed between brown rice,^92^ white rice,^92^ total breakfast cereals,^36^ ^71^ and total rice^73^ ^92^ and total cancer (figs S73-S86 in appendix 2, table 2).

### Whole grains and all cause mortality

Eleven cohort studies (10 publications)^4^ ^9^ ^16^ ^19^ ^20^ ^21^ ^22^ ^25^ ^27^ investigated the association between whole grain intake and all cause mortality and included 100 726 deaths and 705 253 participants. The pooled relative risk for high versus low intake was 0.82 (95% confidence interval 0.77 to 0.88; I^2^=83%, P_heterogeneity_<0.001) (fig S9 in appendix 2, table 1). The summary relative risk was 0.83 (0.77 to 0.90; I^2^=83%, P_heterogeneity_<0.001) per 90 g/day (fig 6, table 1). The heterogeneity was reduced when we excluded two outlying studies^21^ ^25^ (I^2^=66%, P=0.003), but the association was not substantially altered (summary relative risk 0.81, 0.76 to 0.86). Although the test for non-linearity was significant (P<0.001), and steeper reductions in risk were observed at lower intakes, there was a clear dose-response relation, and the lowest risk was observed at 225 g/day (fig 6, fig S10 in appendix 2, table S13 in appendix 1).

**Figure f6:**
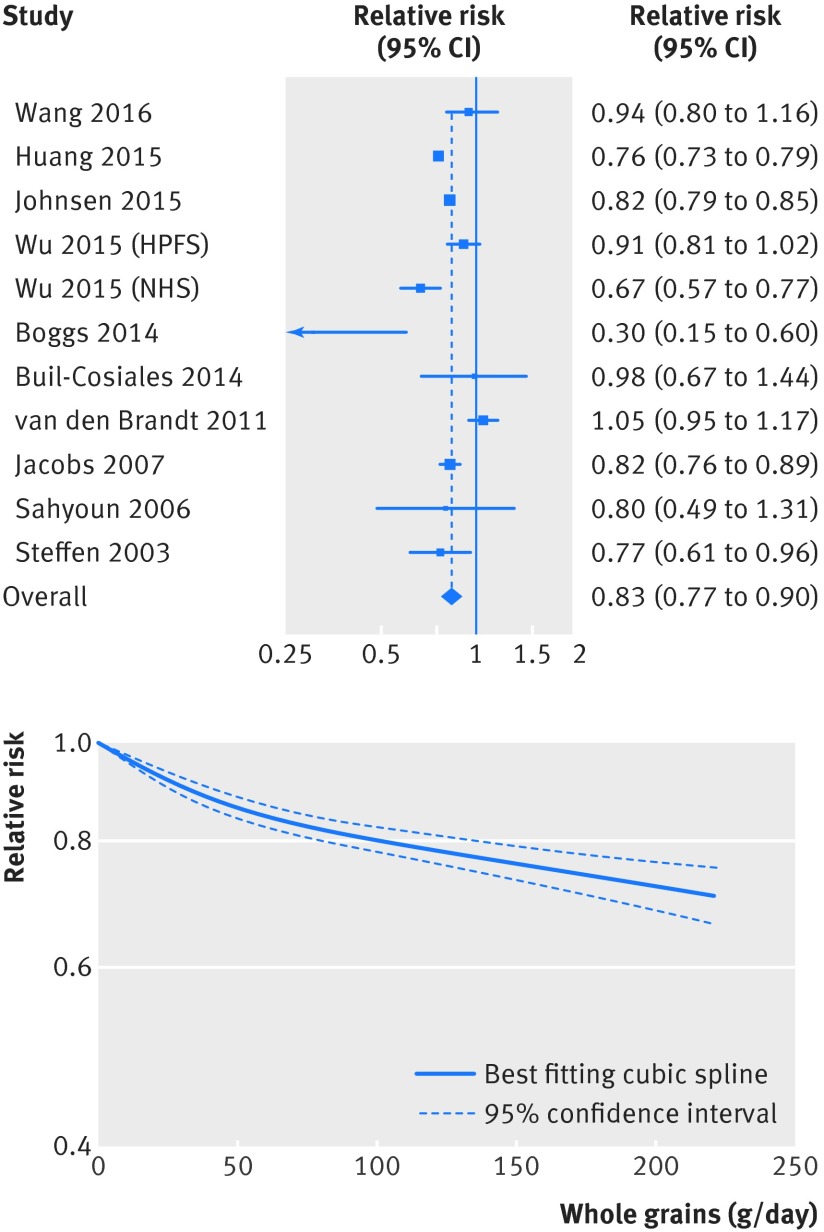
**Fig 6** Forest plot for consumption of whole grains (per 90 g/day) and risk of all cause mortality, with graph illustrating non-linear response

Intakes of whole grain bread,^9^ ^29^ ^52^ ^62^ ^90^ whole grain breakfast cereals,^9^ ^53^ ^87^ pasta, 38 80 total bread, 38 76 77 and total breakfast cereals,^53^ ^71^ ^38^ were inversely associated with all cause mortality, and, in addition, total grain consumption^61^ ^62^ ^63^ ^65^ ^66^ ^76^ ^82^ ^83^ ^84^ ^85^ ^87^ ^88^ ^89^ ^91^ was inversely associated with mortality in the high versus low analysis, but not in the dose-response analysis, while refined grain intake was weakly inversely associated with mortality in the dose-response analysis, but not in the high versus low analysis^4^ ^16^ ^20^ ^63^ (table 2). There was no association between intake of oats or oatmeal^9^ ^86^ ^90^ and mortality (figs S87-S102 in appendix 2, table 2).

### Whole grains and other causes of death

Inverse associations were also observed for the association between whole grains and mortality from respiratory disease (fig 7, figs S11-S12 in appendix 2, table 1, 6617 deaths, 632 849 participants),^4^ ^9^ ^20^ ^22^ diabetes (fig 8, figs S13-S14 in appendix 2, table 1, 808 deaths, 632 849 participants),^4^ ^9^ ^20^ ^22^ infectious diseases (fig 9, fig S15-S16 in appendix 2, table 1, 1386 deaths, 512 839 participants),^4^ ^20^ ^22^ and non-cardiovascular, non-cancer causes (fig 11, figs S19-S20 in appendix 2, table 1, 25 697 deaths, 640 065 participants),^4^ ^9^ ^20^ ^22^ ^27^ but not for diseases of the nervous system (fig 10, fig S17-S18 in appendix 2, table 1, 2285 deaths, 145 397 participants).^4^ ^20^ There was evidence of non-linearity in the analyses of mortality from respiratory disease (P=0.001), diabetes (P<0.001), infectious diseases (P=0.003), and diseases of the nervous system (P<0.001), with most of the reduction in risk observed with intakes up to about 60-90 g/day for diabetes and infectious diseases, but with further reductions in risk with higher intakes for respiratory disease mortality (figs 7-10[Fig f7 f8 f9 f10], figs S12, S14, S16, S18, S20 in appendix 2). The analysis of mortality from diseases of the nervous system showed a slight positive association at low intakes, but no association at intakes of 90 g/day, while the association with all non-cardiovascular, non-cancer causes of death showed little evidence of non-linearity (P=0.06; figs 10-11[Fig f10 f11], table S14 in appendix 1).

**Figure f7:**
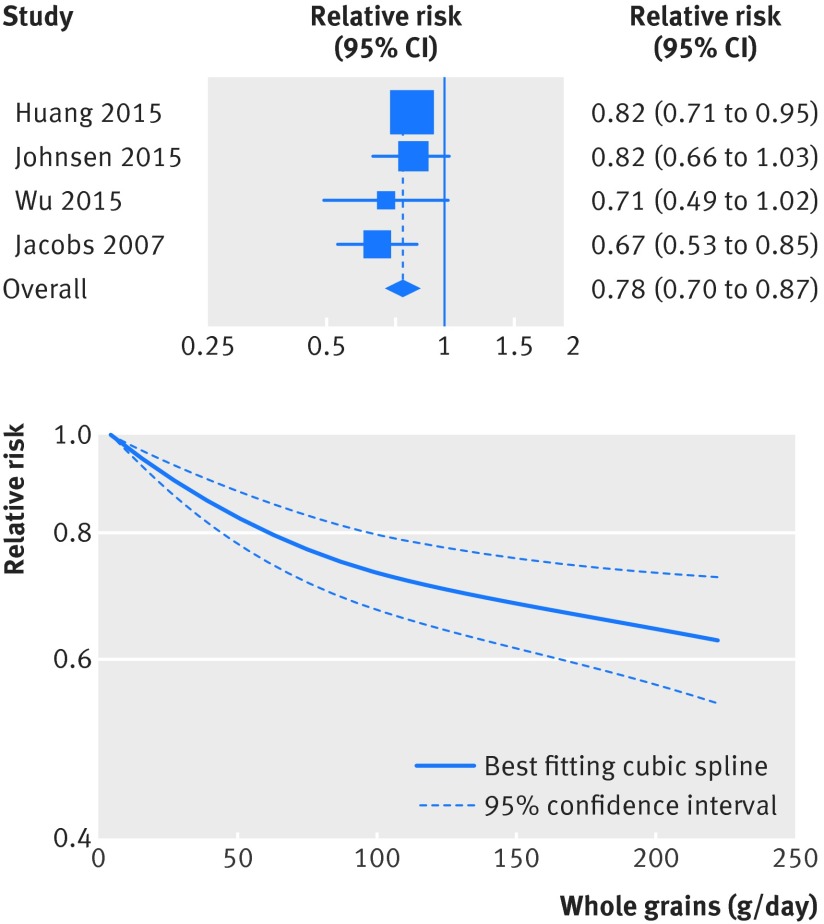
**Fig 7** Forest plot for consumption of whole grains (per 90 g/day) and risk of mortality from respiratory disease, with graph illustrating non-linear response

**Figure f8:**
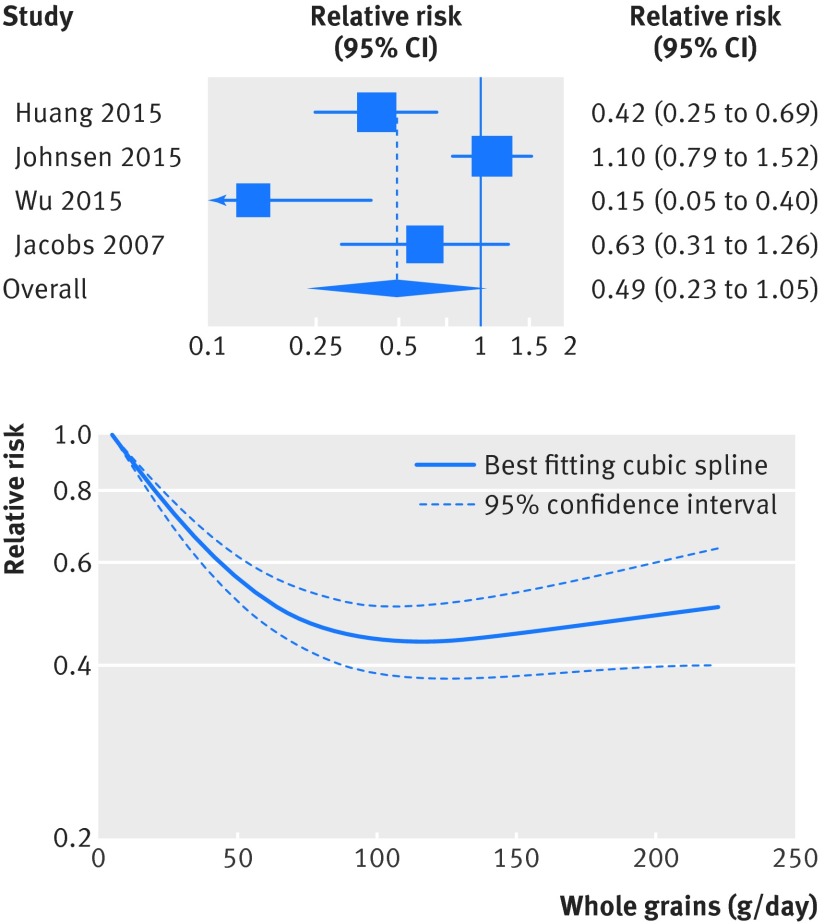
**Fig 8** Forest plot for consumption of whole grains (per 90 g/day) and risk of mortality from diabetes, with graph illustrating non-linear response

**Figure f9:**
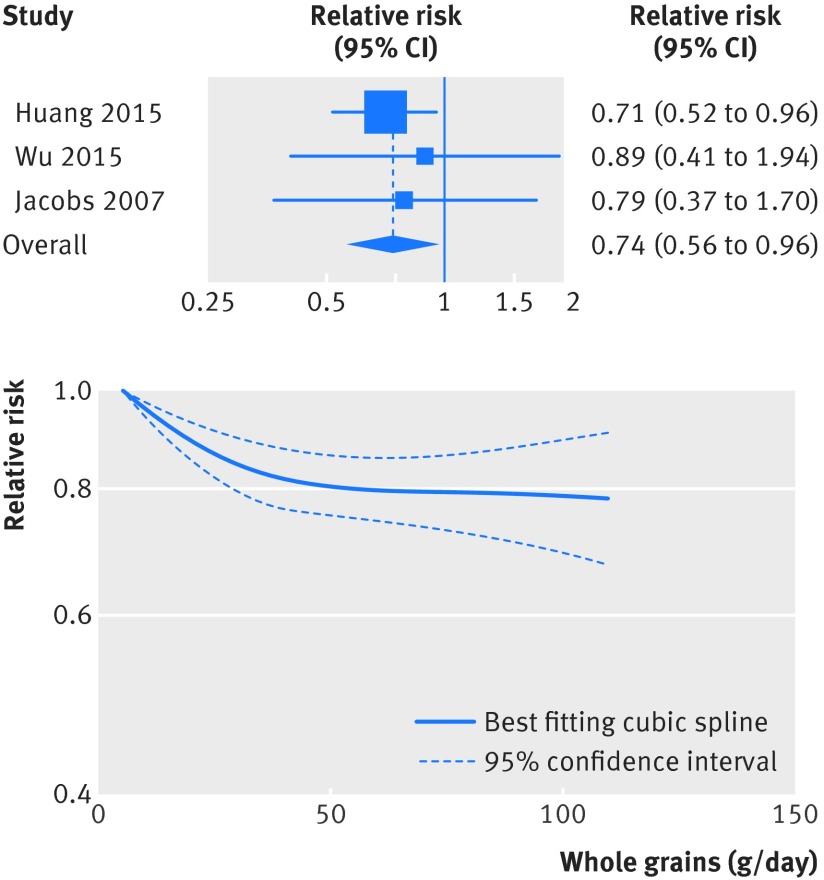
**Fig 9** Forest plot for consumption of whole grains (per 90 g/day) and risk of mortality from infectious diseases, with graph illustrating non-linear response

**Figure f11:**
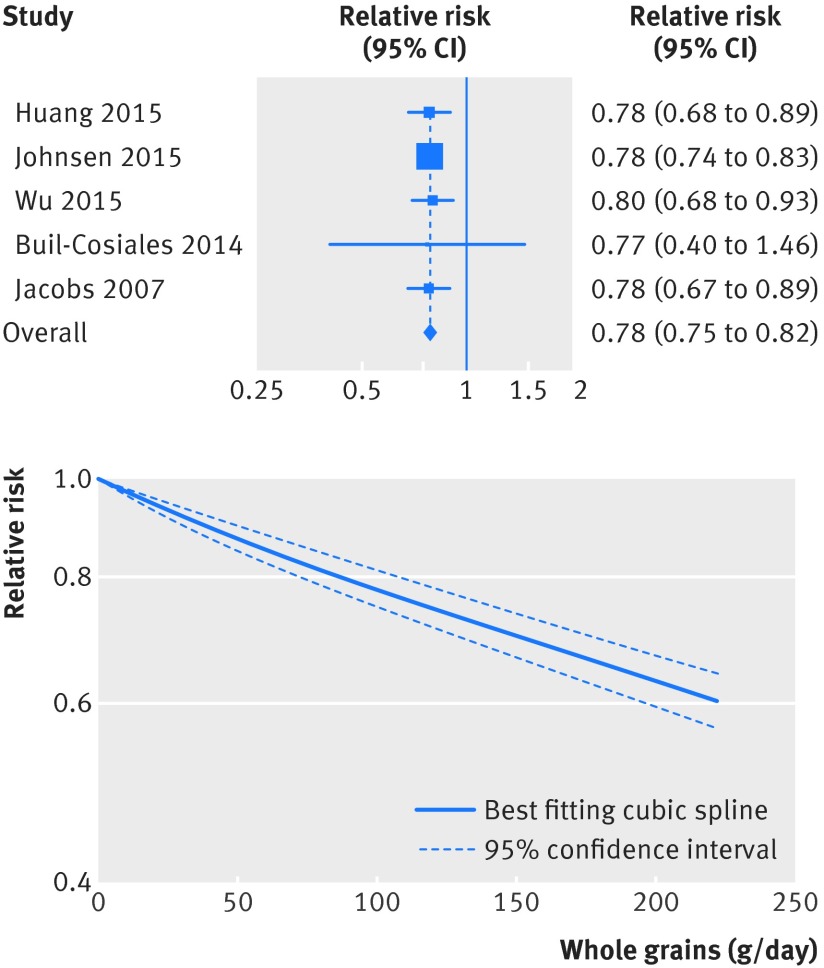
**Fig 11** Forest plot for consumption of whole grains (per 90 g/day) and risk of mortality from non-cardiovascular, non-cancer causes, with graph illustrating non-linear response

**Figure f10:**
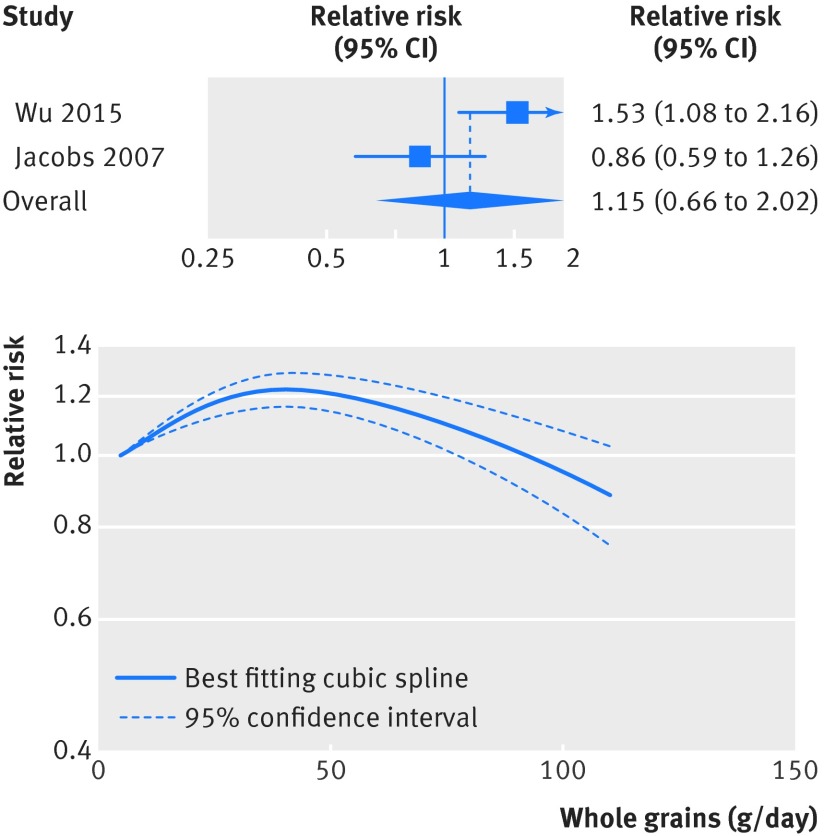
**Fig 10** Forest plot for consumption of whole grains (per 90 g/day) and risk of mortality from diseases of nervous system, with graph illustrating non-linear response

### Publication bias, subgroup and meta-regression analyses, study quality, and influence analyses

There was no evidence of small study bias such as publication bias with Egger’s test for coronary heart disease (P=0.11), cardiovascular disease (P=0.31), total cancer (P=0.44), and all cause mortality (P=0.99) (figs S103-S104 in appendix 2), but some indication for stroke (P=0.01). There were, however, few studies in this analysis, and exclusion of one outlying study^18^ from the analysis attenuated Egger’s test to non-significance (P=0.13) and made the summary estimate significant (0.82, 95% confidence interval 0.72 to 0.93). There was little evidence of heterogeneity between subgroups in subgroup and meta-regression analyses stratified by study characteristics including duration of follow-up, sex, type of outcome, geographical location, number of cases or deaths, or adjustment for confounding factors (table S15-S16 in appendix 1). The association between whole grain intake and coronary heart disease, cardiovascular disease, and total cancer was consistent among both US and European studies, while the association with stroke and all cause mortality was observed only in US studies. For all cause mortality, however, exclusion of one study,^25^ in which intake of whole grains was extremely low (and which partly explained the heterogeneity), made the summary estimate significant for the European studies as well (summary relative risk 0.82, 0.79 to 0.85; I^2^=0%, P_heterogeneity_=0.36). In the analysis of whole grain intake and stroke there was a significant association among studies with stroke mortality as the outcome, among US studies, and among studies with a validated dietary assessment, however, there was no significant difference between these subgroup analyses (table S15 in appendix 1). There was also little evidence of heterogeneity in the remaining subgroup analyses (tables S15-S16 in appendix 1).

Mean (median) study quality scores for the studies on whole grains were 7.9 (8.0) for coronary heart disease, 7.7 (8.0) for stroke, 7.7 (8.0) for cardiovascular disease, 7.8 (8.0) for total cancer, and 7.9 (8.0) for mortality, out of a maximum of 9 points (tables S17-S21 in appendix 1).

In sensitivity analyses in which we excluded one study at a time from each analysis the summary estimates were not substantially altered for coronary heart disease, cardiovascular disease, total cancer, and all cause mortality, but for stroke there was one study^24^ that explained the lack of association (figs S105-S109 in appendix 2).

## Discussion

In this dose-response meta-analysis we found an inverse association between whole grain intake and outcomes of several major chronic diseases, including coronary heart disease, stroke, cardiovascular disease overall, total cancer, and all cause mortality as well as less common causes of death such as from respiratory disease, diabetes, infectious disease, and all non-cardiovascular, non-cancer causes. There were reductions of 21%, 16%, 11%, and 18%, respectively, in the relative risk of coronary heart disease, cardiovascular disease, total cancer, and all cause mortality for the highest versus lowest category of whole grain intake. In the dose-response analyses there were reductions of 19%, 22%, 15%, and 17%, respectively, in the relative risk per 90 g/day (one serving equals 30 g), while the association for stroke was significant only in the non-linear dose-response analysis. There were also reductions of 19%, 36%, 20%, and 21% in the relative risk of mortality from respiratory disease, diabetes, infectious disease, and all non-cardiovascular, non-cancer causes, respectively, with a high versus low intake of whole grains. No evidence of an association was observed for mortality from nervous system disorders in the high versus low or linear dose-response analysis. There was some evidence of a slight positive association at low intakes but not at intakes of 90 g/day or more. There was indication of non-linearity in several of the dose-response analyses, with somewhat steeper reductions in risk at lower levels of intake in most of the analyses. There were, however, further reductions in the risk of coronary heart disease and mortality from cancer, respiratory disease, and all non-cardiovascular, non-cancer causes of death as well from all cause mortality up to intakes as high as 210-225 g/day (seven to seven and a half servings a day).

Although current dietary guidelines recommend whole grains rather than refined grains, recommendations have often not quantified the amount of whole grain intake that should be consumed,^31^ thus the current analysis provides a considerable improvement of the evidence base for the level of whole grains that should be consumed to reduce the risk of chronic diseases and mortality. Relatively few people might have three or more servings a day of whole grains. As indicated by the benefits we observed in the non-linear dose-response analyses at an intake of even one or two servings a day in relation to most of the outcomes, however, even moderate increases in whole grain intake could reduce the risk of premature mortality. Also, as many people might have a total grain intake of three or more servings a day, replacement of most or all of the refined grains with whole grains could increase whole grain intake substantially.

Although there was some evidence of non-linear associations between whole grain intake and coronary heart disease, stroke, cardiovascular disease, and mortality from all causes, respiratory disease, diabetes, and infections, with stronger reductions in risk observed at lower levels of intake, in most of the analyses there was a clear dose-response relation with further reductions with intakes up to seven to seven and a half servings a day (210-225 g/day). In addition, there were inverse associations for some subtypes of whole grains or total grains and coronary heart disease (whole grain bread, whole grain breakfast cereals, added bran), cardiovascular disease (whole grain bread, whole grain breakfast cereals, bran, total breakfast cereals), total cancer (whole grain bread, total grains), and all cause mortality (whole grain bread, whole grain cereals, total grains, total bread, pasta), which supports the findings for whole grain intake overall. There was little evidence of an association between intake of refined grains and any of the outcomes. The number of studies in the analyses of grain subtypes, however, was low. Given that whole grain consumption differs substantially between populations, both with regard to type and amount, and because most of the current data are from US and European studies it is possible that effect sizes might differ in other populations.

### Limitations of the study

Our meta-analysis has some limitations that should be mentioned. There was high heterogeneity in the analysis of whole grains and all cause mortality. With the exception of one study from the Netherlands,^25^ which had a small range of whole grain intake (the interquartile range was 0-0 and 10.6-13.5 g/d in men/women, respectively), however, the heterogeneity seemed to be more due to differences in the strength of the association between studies than to differences in the direction of the association. Exclusion of two outlying studies^21^ ^25^ reduced the heterogeneity in the analysis of all cause mortality but did not substantially alter the summary estimates.

Although we took into account the different amounts and ranges of whole grain intake between studies in the dose-response analysis, studies could also have differed by the types of whole grains consumed, by how accurately they measured whole grain intake, or by how they defined whole grains. This could have contributed to heterogeneity between studies. In addition, given the diversity of whole grain products available it is difficult to assess intake accurately in epidemiological studies, and some degree of measurement error is inevitable. A recent review recommended reporting intake as the actual amount of whole grain per dry weight.^93^ As some studies have classified some whole grain items (breakfast cereals, muesli) as whole grain foods if they have a whole grain content of ≥25% or >50% of the weight of the product, then a grain product could be considered whole grain if its whole grain content varied between 25-100 g or 51-100 g per 100 g of the product. Somebody could consume a product with 24 g or 50 g of whole grain per 100 g of the product and still be considered to eat no whole grain, leading to misclassification of the exposure. Most of the studies seemed to report intake as the amount or frequency of whole grain food or product intake (fresh weight including water content), while only two publications^9^ ^20^ reported intakes in actual amount of whole grain food (dry weight). One study that reported results for both whole grain products (fresh weight) and actual whole grain intake (dry weight) in relation to mortality, however, found similar associations for the two.^9^ Most of the associations were similar for different types of whole grains, and, in addition, most of the US studies seemed to define whole grains similarly, while few of the European studies provided a definition of whole grain.

People with a high intake of whole grains might have different lifestyles, diets,^20^ ^94^ or socioeconomic status^94^ than those with a low intake, thus confounding by other lifestyle factors is a potential source of bias. In subgroup analyses we found that the associations observed persisted among studies that adjusted for smoking, alcohol, physical activity, BMI, and other dietary factors such as sugar sweetened beverages, red meat, and fruit and vegetables. Though differences in socioeconomic factors or deprivation could also have influenced the findings, both the Nurses’ Health Study and the Health Professionals Follow-up Study, cohorts in which there would be relatively little confounding by socioeconomic status or deprivation, found similar results to the overall analysis, and there was no evidence of heterogeneity in the results stratified by adjustment for education.

The number of studies that investigated subtypes of whole grains and total or refined grains was limited. Any further studies should therefore try to clarify associations between specific subtypes of grains and cardiovascular disease, cancer, and mortality, as well as less common causes of mortality. As in any meta-analysis of published studies publication bias could have influenced the results. Though we found some indication of small study effects such as publication bias in the analysis of stroke, there was no evidence of publication bias for the remaining outcomes, although the number of studies was moderate and power to detect such bias is low when there are few studies.

### Strengths of the study

Strengths of the current study include the comprehensive analyses of intake of whole grain and subtypes of grain in relation to a range of chronic disease and mortality outcomes including high versus low analyses; linear and non-linear dose-response analyses; the detailed subgroup, sensitivity, and influence analyses; the large numbers of cases or deaths and participants included; and the high quality of the studies included.

### Mechanisms

Several mechanisms could explain the beneficial effect observed between whole grain intake and coronary heart disease, cardiovascular disease, cancer, and all cause and cause specific mortality. Whole grains are rich in fibre, which can reduce the postprandial glucose and insulin responses leading to better glycaemic control.^95^ Epidemiological studies have suggested a lower risk of overweight and obesity^6^ ^96^ ^97^ and of type 2 diabetes^5^ ^6^ among people with a high whole grain intake. Though both adiposity and type 2 diabetes are established risk factors for cardiovascular disease, cancer, and mortality, in our analysis all the studies adjusted for BMI, suggesting an association independent of BMI. One study on whole grains and coronary heart disease^17^ and another study on mortality^9^ found little difference between hazard ratios adjusted or not adjusted for BMI, so if anything BMI might mediate only a small part of the association.

Higher whole grain intake has been associated with a lower prevalence or risk of hypertension or raised blood pressure,^95^ ^98^ ^99^ hypertriglyceridaemia,^95^ ^100^ and lower concentrations of total and low density lipoprotein cholesterol,^97^ ^100^ which are important cardiovascular risk factors. Higher fibre intake has been associated with reduced risk of coronary heart disease,^101^ stroke,^102^ some cancers,^28^ ^103^ and all cause mortality.^104^ ^105^ ^106^ Fibre intake, in particular soluble fibre, might reduce cholesterol concentrations by inhibiting reabsorption of bile acid and by bacterial fermentation of fibre in the colon, which results in the production of short chain fatty acids, which inhibit cholesterol synthesis in the liver.^107^ Dietary fibre can reduce the risk of cancer by mechanic removal of damaged cells from the digestive tract,^108^ increasing stool bulk, diluting carcinogens, decreasing transit time, altering the gut microbiota,^28^ ^109^ ^110^ ^111^ and binding oestrogens in the colon and increasing the faecal excretion of oestrogens, leading to lower oestrogen concentrations.^103^ ^112^

Whole grain consumption has been found to be inversely associated with mortality from inflammatory diseases,^4^ and an intervention study found reduced concentrations of fasting serum glucose, measures of lipid peroxidation, and homocysteine concentrations among participants fed a whole grain/legume powder supplement.^113^ Whole grain intake has been associated with lower levels of inflammatory markers (PAI-1, CRP)^114^ ^115^ ^116^ ^117^ and liver enzymes (GGT, ASAT),^117^ higher levels of which have been associated with increased risk of cardiovascular disease, cancer, and mortality.^118^ ^119^ ^120^ Whole grain intake has also been associated with higher levels of adiponectin,^116^ which increases insulin sensitivity and reduces inflammation. Whole grains also contain several other potentially beneficial components that could explain some of the current findings.^121^

Further studies are needed to clarify whether there is an underlying mechanism for the non-linear association between whole grain intake and cardiovascular disease, all cause mortality, and mortality from respiratory disease, diabetes, and infectious diseases. A high intake of whole grains could also reduce the risk of chronic disease and mortality indirectly, by displacement of unhealthy foods or drinks. The association for cardiovascular disease and mortality, however, persisted in studies that adjusted for intake of red and processed meat and sugar sweetened beverages.

### Policy implications and future research

We found that a high whole grain intake was associated with reduced risk of coronary heart disease, cardiovascular disease, total cancer, and all cause mortality as well as mortality from respiratory disease, infections, diabetes, and all non-cardiovascular, non-cancer causes combined. This is strong evidence that a high intake of whole grains is beneficial for several health outcomes in a dose-response manner. In addition, a high intake of whole grains has previously been associated with reduced risk of colorectal cancer,^28^ type 2 diabetes,^5^ and overweight or obesity.^6^ Altogether these findings have important public health implications as whole grain intake can be modified relatively easily by replacing refined grains and could have a large effect on the burden of chronic disease if adopted in the general population. As shown in the current meta-analysis, a high intake of whole grain is not only associated with reduced risk of cardiovascular disease and diabetes but also with mortality from cancer, respiratory disease, infectious disease, and all non-cancer, non-cardiovascular causes combined. The current findings therefore strongly support existing dietary recommendations to increase whole grain consumption in the general population. From a practical angle a whole grain product intake of 90 g/day can be achieved, for example, by eating a portion of whole grain breakfast cereals (30-40 g) at breakfast and a piece of whole grain pita bread for dinner (60 g). The non-linear analyses suggested that the reduction in risk of mortality is steepest at the lowest level of whole grain intake (people who increase from no intake of whole grain to two servings/day) and that perhaps targeting subjects with a very low intake might have a greater impact. Further reductions were observed up to 210-225 g/day (seven to seven and a half servings a day), however, suggesting further benefits with even higher intakes. Most of the studies included in the analyses of whole grains were from the US, and only a few European studies have been published so far. Whole grain intake is higher in Northern Europe^34^ ^122^ than in the US, and populations in Northern Europe might therefore be promising for further studies of the association between whole grains and health outcomes, both in terms of examining more extreme intakes and specific types of whole grains. Further studies are needed in other geographical locations, as are studies of specific diseases and less common causes of death and that incorporate biomarkers of whole grain intake.^123^

In conclusion our results provide further evidence for the beneficial effects of diets high in whole grains on the risk of coronary heart disease, cardiovascular disease, total cancer, and all cause mortality, as well as mortality from respiratory disease, infections, diabetes, and all non-cardiovascular, non-cancer causes combined. Reductions in risk are observed up to 210-225 g/day or seven to seven and a half servings a day, and the current findings support dietary recommendations to increase intake of whole grains and as much as possible to choose whole grains rather than refined grains.

**What is already known on this topic**

A high intake of whole grains has been associated with a lower risk of type 2 diabetes, cardiovascular disease, and weight gain
Recommendations for whole grain intake have often been unclear or inconsistent with regard to the amount and types of whole grain foods that should be consumed to reduce chronic disease and risk of mortality

**What this study adds**

A high intake of whole grains was associated with reduced risk of coronary heart disease, cardiovascular disease, total cancer, and all cause mortality, as well as mortality from respiratory disease, infectious disease, diabetes, and all non-cardiovascular, non-cancer causes
Reductions in risk were observed up to an intake of 210-225 g/day (seven to seven and a half servings/day) and for whole grain bread, whole grain breakfast cereals, and added bran
The results strongly support dietary recommendations to increase intake of whole grain foods in the general population to reduce risk of chronic diseases and premature mortality

## Web Extra

Extra material supplied by the author

## Notes

We thank Tao Huang and Lu Qi (Department of Nutrition, Harvard T Chan School of Public Health) for clarification of the data from the NIH-AARP Diet and Health Study, and Diewertje Sluik (Division of Human Nutrition, Wageningen University) for clarification of the data from the European Prospective Investigation into Cancer and Nutrition study.

Contributors: DA and TN conceived and designed the study. DA, NK, EG, LTF, PB, TN, DCG, ER, and ST acquired, analysed, and interpreted the data. DCG checked data extractions. DA drafted the manuscript, which was critically revised for important intellectual content by all authors. DA and DCG carried out the statistical analysis. DA, LJV, ST, and ER obtained funding. TN supervised the study. All authors have read and approved the final manuscript. DA is guarantor and had full access to all the data and takes responsibility for the integrity of the data and the accuracy of the data analysis.

Ethical approval: Not required.

Transparency: The lead author affirms that the manuscript is an honest, accurate, and transparent account of the study being reported; that no important aspects of the study have been omitted; and that any discrepancies from the study as planned have been disclosed.

Data sharing: No additional data available.
