# Supplementary material for: Whole grain consumption and risk of cardiovascular disease, cancer, and all cause and cause specific mortality: systematic review and dose-response meta-analysis of prospective studies
Source: BMJ. 2016 Jun 14;353:i2716. doi: 10.1136/bmj.i2716 (PMC4908315; doi:10.1136/bmj.i2716)
Supplement: Supplementary file 1 — Appendix 1: Supplementary tables S1-S21 [file aund029745.ww1_default.pdf]

**Appendix 1: Supplementary tables [posted as supplied by author]**

**Table S1. Search strategy in PubMed and Embase**

| |
|---|
| 1. fruits |
| 2. vegetables |
| 3. fruit |
| 4. vegetable |
| 5. berry |
| 6. berries |
| 7. citrus |
| 8. "citrus fruits" |
| 9. cruciferae |
| 10. "cruciferous vegetables" |
| 11. cabbages |
| 12. "allium vegetables" |
| 13. strawberry |
| 14. strawberries |
| 15. tomato |
| 16. tomatoes |
| 17. cereal |
| 18. cereals |
| 19. "breakfast cereal" |
| 20. grain |
| 21. grains |
| 22. "whole grain" |
| 23. "whole grains" |
| 24. rice |
| 25. bread |
| 26. nut |
| 27. seed |
| 28. peanut |
| 29. peanuts |
| 30. legumes |
| 31. soy |
| 32. soya |
| 33. chickpeas |
| 34. chickpea |
| 35. bean |
| 36. beans |
| 37. lentil |
| 38. legume |
| 39. legumes |
| 40. fiber |
| 41. "dietary fiber" |
| 42. "fruit fiber" |
| 43. "vegetable fiber" |
| 44. "legume fiber" |
| 45. "cereal fiber" |
| 46. fibre |
| 47. "dietary fibre" |

| |
|---|
| 48. “fruit fibre” |
| 49. “vegetable fibre” |
| 50. “cereal fibre” |
| 51. “DASH diet” |
| 52. diet |
| 53. foods |
| 54. “dietary patterns” |
| 55. “dietary pattern” |
| 56. “dietary score” |
| 57. “diet score” |
| 58. “diet index” |
| 59. “food index” |
| 60. “nutrient index” |
| 61. “Mediterranean diet” |
| 62. “vitamin C” |
| 63 “ascorbic acid” |
| 64. “vitamin E” |
| 65. carotenoids |
| 66. carotenoid |
| 67. flavonoid |
| 68. flavonoids |
| 69. (1 OR 2 OR 3 OR 4 OR 5 OR 6 OR 7 OR 8 OR 9 OR 10 OR 11 OR 12 OR 13 OR 14 OR 15 OR 16 OR 17 OR 18 OR 19 OR 20 OR 21 OR 22 OR 23 OR 24 OR 25 OR 26 OR 27 OR 28 OR 29 OR 30 OR 31 OR 32 OR 33 OR 34 OR 35 OR 36 OR 37 OR 38 OR 39 OR 40 OR 41 OR 42 OR 43 OR 44 OR 45 OR 46 OR 47 OR 48 OR 49 OR 50 OR 51 OR 52 OR 53 OR 54 OR 55 OR 56 OR 57 OR 58 OR 59 OR 60 OR 61 OR 62 OR 63 OR 64 OR 65 OR 66 OR 67 OR 68) |
| 70. “coronary heart disease” |
| 71. “heart disease” |
| 72. “ischemic heart disease” |
| 73. “ischaemic heart disease” |
| 74. CHD |
| 75. “coronary artery disease” |
| 76. “myocardial infarction” |
| 77. stroke |
| 78. “ischemic stroke” |
| 79. “haemorrhagic stroke” |
| 80. “cardiovascular disease” |
| 81. CVD |
| 82. cancer |
| 83. “total cancer” |
| 84. mortality |
| 85. “all-cause mortality” |
| 86. “total mortality” |
| 87. survival |
| 88. (70 OR 71 OR 72 OR 73 OR 74 OR 75 OR 76 OR 77 OR 78 OR 79 OR 80 OR 81 OR 82 OR 83 OR 84 OR 85 OR 86 OR 87) |
| 89. “case-control” |
| 90. cohort |

| |
|---|
| 91. cohorts |
| 92. prospective |
| 93. longitudinal |
| 94. retrospective |
| 95. “follow-up” |
| 96. “cross-sectional” |
| 97. “population-based” |
| 98. “relative risk |
| 99. “odds ratio” |
| 100 “hazard ratio” |
| 101 “incidence rate ratio” |
| 102 (89 OR 90 OR 91 OR 92 OR 93 OR 94 OR 95 OR 96 OR 97 OR 98 OR 99 OR 100 OR 101) |
| 103. 69 AND 88 AND 102 |

**Table S2. List of excluded studies and reason for exclusion**

| Exclusion reason | Reference number |
|---|---|
| Abstract only publication | (1-10) |
| Case-control study | (11-24) |
| Cross-sectional study | (25) |
| Crude dietary assessment | (26) |
| Diabetes patient population | (27;28) |
| Duplicates | (29-34) |
| Ecological study | (35) |
| Meta-analysis | (36-44) |
| No confidence intervals | (45;46) |
| No risk estimates | (47) |
| Not original data | (48) |
| Not usable result | (49) |
| Not relevant exposure | (50;51) |
| Not relevant outcome | (52-58) |
| Patients with heart disease | (59) |
| Qualitative assessment (whole grain vs. refined grains) | (60;61) |
| Quantity not provided | (62) |
| Review | (63-80) |
| Total disease mortality as outcome (not all-cause mortality), quantity not provided | (81) |
| Unadjusted risk estimates | (82) |

Reference List


1. Wengreen H, Quach A, Cutler A, Munger R, Corcoran C. Whole-grain intake and risk of all-cause mortality among elderly men and women: The Cache, County Study on Memory, Health and Aging. *FASEB Journal* 2012;26:119.2.

2. Sonestedt E, Hellstrand S, Orho-Melander M. Carbohydrate-rich foods and risk of cardiovascular disease in the Malmo diet and cancer cohort. *European Journal of Epidemiology* 2013;28 (1 Suppl):S184: P-302.

3. Yu D, Shu X-O, Li H et al. High intakes of dietary carbohydrate and rice were associated with increased risk of coronary heart disease in chinese men and women. *Circulation* 2013;127:AMP24.

4. Qi L, Xu M, Lee A, Cho S. Ready to eat cereal consumption with total and cause-specific mortality: Prospective analysis of 367,442 individuals. *FASEB Journal* 2014;28:810.20.

5. Qi L, Xu M, Huang T, Lee A, Cho S. Consumption of whole grain and cereal fiber with total and cause-specific mortality: Prospective analysis of 367,442 individuals. *FASEB Journal* 2014;28:628.17.

6. Kokubo Y. Updates on stroke epidemiology in Japan. *Neuroepidemiology* 2014;43:80: PS10-4.

7. McKeown NM, Hruby A, Landberg R, Herrington DM, Lichtenstein AH. Plasma alkylresorcinol, a biomarker of whole-grain intake, is not associated with progression of coronary artery atherosclerosis in postmenopausal women with coronary artery disease. *Circulation* 2014;129:AP408.

8. Wu H, Flint AJ, Van DR et al. Whole grain intake and risk of all-cause and cause-specific mortality in us men and women. *Circulation* 2014;129:A18.

9. Bongard V, Arveiler D, Dallongeville J et al. Food groups associated with a reduced risk of 15year all-cause death. *FASEB Journal* 2015;29:736.32.

10. Krittanawong C, Tunhasiriwet A, Bisanz KJ et al. Is white rice consumption a risk for cardiovascular disease? A systematic review and meta-analysis. *Circulation* 2015;132(Suppl. 3): Abstract 17006.

11. Panagiotakos DB, Rallidis LS, Katsiotis E, Pitsavos C, Stefanadis C, Kremastinos DT. Background dietary habits are strongly associated with the development of myocardial infarction at young ages: A case-control study. *e-SPEN* 2008;3:e328-e334.

12. Gramenzi A, Gentile A, Fasoli M, Negri E, Parazzini F, La VC. Association between certain foods and risk of acute myocardial infarction in women. *BMJ* 1990;300:771-3.

13. Genchev GD, Georgieva LM, Weijenberg MP, Powles JW. Does alcohol protect against ischaemic heart disease in Bulgaria? A case-control study of non-fatal myocardial infarction in Sofia. *Cent Eur J Public Health* 2001;9:83-6.

14. Sasazuki S. Case-control study of nonfatal myocardial infarction in relation to selected foods in Japanese men and women. *Jpn Circ J* 2001;65:200-6.

15. Martinez-Gonzalez MA, Fernandez-Jarne E, Serrano-Martinez M, Marti A, Martinez JA, Martin-Moreno JM. Mediterranean diet and reduction in the risk of a first acute myocardial infarction: an operational healthy dietary score. *Eur J Nutr* 2002;41:153-60.

16. Martinez-Gonzalez MA, Fernandez-Jarne E, Martinez-Losa E, Prado-Santamaria M, Brugarolas-Brufau C, Serrano-Martinez M. Role of fibre and fruit in the Mediterranean diet to protect against myocardial infarction: a case-control study in Spain. *Eur J Clin Nutr* 2002;56:715-22.

17. Rastogi T, Reddy KS, Vaz M et al. Diet and risk of ischemic heart disease in India. *Am J Clin Nutr* 2004;79:582-92.

18. Tavani A, Bertuzzi M, Gallus S, Negri E, La VC. Risk factors for non-fatal acute myocardial infarction in Italian women. *Prev Med* 2004;39:128-34.

19. Lockheart MS, Steffen LM, Rebnord HM et al. Dietary patterns, food groups and myocardial infarction: a case-control study. *Br J Nutr* 2007;98:380-7.

20. Iqbal R, Anand S, Ounpuu S et al. Dietary patterns and the risk of acute myocardial infarction in 52 countries: results of the INTERHEART study. *Circulation* 2008;118:1929-37.

21. Liang W, Lee AH, Binns CW. White rice-based food consumption and ischemic stroke risk: a case-control study in southern China. *J Stroke Cerebrovasc Dis* 2010;19:480-4.

22. Guo J, Li W, Wang Y et al. Influence of dietary patterns on the risk of acute myocardial infarction in China population: the INTERHEART China study. *Chin Med J (Engl )* 2013;126:464-70.

23. Turati F, Pelucchi C, Galeone C, Praud D, Tavani A, La VC. Mediterranean diet and non-fatal acute myocardial infarction: a case-control study from Italy. *Public Health Nutr* 2015;18:713-20.

24. Lipoeto NI, Agus Z, Oenzil F, Wahlqvist M, Wattanapenpaiboon N. Dietary intake and the risk of coronary heart disease among the coconut-consuming Minangkabau in West Sumatra, Indonesia. *Asia Pac J Clin Nutr* 2004;13:377-84.

25. Magnanti SM, Fernandes J, Hirshberg SE, Lofgren I. Whole grain intake and coronary heart disease risk in young adults. *FASEB Journal* 2011;25:339.7.

26. Vormund K, Braun J, Rohrmann S, Bopp M, Ballmer P, Faeh D. Mediterranean diet and mortality in Switzerland: an alpine paradox? *Eur J Nutr* 2015;54:139-48.

27. Trichopoulou A, Psaltopoulou T, Orfanos P, Trichopoulos D. Diet and physical activity in relation to overall mortality amongst adult diabetics in a general population cohort. *J Intern Med* 2006;259:583-91.

28. He M, van Dam RM, Rimm E, Hu FB, Qi L. Whole-grain, cereal fiber, bran, and germ intake and the risks of all-cause and cardiovascular disease-specific mortality among women with type 2 diabetes mellitus. *Circulation* 2010;121:2162-8.

29. Jacobs DR, Jr., Meyer KA, Kushi LH, Folsom AR. Is whole grain intake associated with reduced total and cause-specific death rates in older women? The Iowa Women's Health Study. *Am J Public Health* 1999;89:322-9.

30. Trichopoulou A, Costacou T, Bamia C, Trichopoulos D. Adherence to a Mediterranean diet and survival in a Greek population. *N Engl J Med* 2003;348:2599-608.

31. Benetou V, Trichopoulou A, Orfanos P et al. Conformity to traditional Mediterranean diet and cancer incidence: the Greek EPIC cohort. *Br J Cancer* 2008;99:191-5.

32. Burger KN, Beulens JW, van der Schouw YT et al. Dietary fiber, carbohydrate quality and quantity, and mortality risk of individuals with diabetes mellitus. *PLoS One* 2012;7:e43127.

33. Schroder H, Salas-Salvado J, Martinez-Gonzalez MA et al. Baseline adherence to the Mediterranean diet and major cardiovascular events: Prevencion con Dieta Mediterranea trial. *JAMA Intern Med* 2014;174:1690-2.

34. Key TJ, Thorogood M, Appleby PN, Burr ML. Dietary habits and mortality in 11,000 vegetarians and health conscious people: results of a 17 year follow up. *BMJ* 1996;313:775-9.

35. Armstrong BK, Mann JI, Adelstein AM, Eskin F. Commodity consumption and ischemic heart disease mortality, with special reference to dietary practices. *J Chronic Dis* 1975;28:455-69.

36. Jacobs DR, Jr., Marquart L, Slavin J, Kushi LH. Whole-grain intake and cancer: an expanded review and meta-analysis. *Nutr Cancer* 1998;30:85-96.

37. Mellen PB, Walsh TF, Herrington DM. Whole grain intake and cardiovascular disease: a meta-analysis. *Nutr Metab Cardiovasc Dis* 2008;18:283-90.

38. Mente A, de KL, Shannon HS, Anand SS. A systematic review of the evidence supporting a causal link between dietary factors and coronary heart disease. *Arch Intern Med* 2009;169:659-69.

39. Chacko S, Ye EQ, Chou EL, Kugizaki M, Liu S. Relation of whole grain intake to risk of type 2 diabetes, cardiovascular disease and weight gain: A systematic review and meta-analysis. *FASEB Journal* 2012; 26(Suppl. 1):239.1

40. Ye EQ, Chacko SA, Chou EL, Kugizaki M, Liu S. Greater whole-grain intake is associated with lower risk of type 2 diabetes, cardiovascular disease, and weight gain. *Journal of Nutrition* 2012;142:1304-13.

41. Tang G, Wang D, Long J, Yang F, Si L. Meta-analysis of the association between whole grain intake and coronary heart disease risk. *Am J Cardiol* 2015;115:625-9.

42. Jacobs J, Marquart L, Slavin J, Kushi LH. Whole-grain intake and cancer: An expanded review and meta-analysis. *Nutrition and Cancer* 1998;30:1998.

43. Wu D, Guan Y, Lv S, Wang H, Li J. No Evidence of Increased Risk of Stroke with Consumption of Refined Grains: A Meta-analysis of Prospective Cohort Studies. *J Stroke Cerebrovasc Dis* 2015;24:2738-46.

44. Fang L, Li W, Zhang W, Wang Y, Fu S. Association between whole grain intake and stroke risk: evidence from a meta-analysis. *Int J Clin Exp Med* 2015;8:16978-83.

45. Burr ML, Sweetnam PM. Vegetarianism, dietary fiber, and mortality. *Am J Clin Nutr* 1982;36:873-7.

46. Tanaka H, Date C, Hayashi M et al. Trends in death and consultation rates of ischemic heart disease in Japan and the risk factors in a rural community. *Jpn Circ J* 1987;51:306-13.

47. Nube M, Kok FJ, Vandenbroucke JP, van dH-W, van der Heide RM. Scoring of prudent dietary habits and its relation to 25-year survival. *J Am Diet Assoc* 1987;87:171-5.

48. de Oliveira Otto MC, Afshin A, Micha R et al. The Impact of Dietary and Metabolic Risk Factors on Cardiovascular Diseases and Type 2 Diabetes Mortality in Brazil. *PLoS One* 2016;11:e0151503.

49. Reedy J, Krebs-Smith SM, Miller PE et al. Higher diet quality is associated with decreased risk of all-cause, cardiovascular disease, and cancer mortality among older adults. *J Nutr* 2014;144:881-9.

50. Ishihara T, Kobayashi E, Okubo Y et al. Association between cadmium concentration in rice and mortality in the Jinzu River basin, Japan. *Toxicology* 2001;163:28.

51. Nakamura Y, Ueshima H, Okamura T et al. A Japanese diet and 19-year mortality: national integrated project for prospective observation of non-communicable diseases and its trends in the aged, 1980. *Br J Nutr* 2009;101:1696-705.

52. Erkkila AT, Herrington DM, Mozaffarian D, Lichtenstein AH. Cereal fiber and whole-grain intake are associated with reduced progression of coronary-artery atherosclerosis in postmenopausal women with coronary artery disease. *American Heart Journal* 2005;150:94-101.

53. McCullough ML, Feskanich D, Rimm EB et al. Adherence to the Dietary Guidelines for Americans and risk of major chronic disease in men. *Am J Clin Nutr* 2000;72:1223-31.

54. McCullough ML, Feskanich D, Stampfer MJ et al. Adherence to the Dietary Guidelines for Americans and risk of major chronic disease in women. *Am J Clin Nutr* 2000;72:1214-22.

55. La Vecchia C, Chatenoud L, Negri E, Franceschi S. Session: Whole cereal grains, fibre and human cancer wholegrain cereals and cancer in Italy. *Proceedings of the Nutrition Society* 2003;62:45-9.

56. Djousse L, Gaziano JM. Breakfast cereals and risk of heart failure in the physicians' health study I. *Arch Intern Med* 2007;167:2080-5.

57. Nettleton JA, Steffen LM, Loehr LR, Rosamond WD, Folsom AR. Incident heart failure is associated with lower whole-grain intake and greater high-fat dairy and egg intake in the Atherosclerosis Risk in Communities (ARIC) study. *J Am Diet Assoc* 2008;108:1881-7.

58. Flint AJ, Hu FB, Glynn RJ et al. Whole grains and incident hypertension in men. *Am J Clin Nutr* 2009;90:493-8.

59. Karim MA, Majumder AA, Islam KQ et al. Risk factors and in-hospital outcome of acute ST segment elevation myocardial infarction in young Bangladeshi adults. *BMC Cardiovasc Disord* 2015;15:73.

60. Fraser GE, Sabate J, Beeson WL, Strahan TM. A possible protective effect of nut consumption on risk of coronary heart disease. The Adventist Health Study. *Arch Intern Med* 1992;152:1416-24.

61. Whiteman D, Muir J, Jones L, Murphy M, Key T. Dietary questions as determinants of mortality: the OXCHECK experience. *Public Health Nutr* 1999;2:477-87.

62. Yu D, Zhang X, Xiang YB et al. Adherence to dietary guidelines and mortality: a report from prospective cohort studies of 134,000 Chinese adults in urban Shanghai. *Am J Clin Nutr* 2014;100:693-700.

63. Kushi LH, Meyer KA, Jacobs DR, Jr. Cereals, legumes, and chronic disease risk reduction: evidence from epidemiologic studies. *Am J Clin Nutr* 1999;70:451S-8S.

64. Liu S, Manson JE. Dietary carbohydrates, physical inactivity, obesity, and the "metabolic syndrome" as predictors of coronary heart disease. *Current Opinion in Lipidology* 2001;12:395-404.

65. McKeown NM, Jacques P. Whole grain intake and risk of ischemic stroke in women. *Nutr Rev* 2001;59:149-52.

66. Hu FB, Willett WC. Optimal diets for prevention of coronary heart disease. *JAMA* 2002;288:2569-78.

67. Truswell AS. Cereal grains and coronary heart disease. *Eur J Clin Nutr* 2002;56:1-14.

68. Hu FB. Plant-based foods and prevention of cardiovascular disease: an overview. *Am J Clin Nutr* 2003;78:544S-51S.

69. Jacobs DR, Jr., Meyer HE, Solvoll K. [Consumption of whole grain foods and chronic disease]. *Tidsskr Nor Laegeforen* 2004;124:1399-401.

70. Slavin J. Whole grains and human health. *Nutr Res Rev* 2004;17:99-110.

71. Jacobs J, Gallaher DD. Whole grain intake and cardiovascular disease: A review. *Current Atherosclerosis Reports* 2004;6:415-23.

72. Seal CJ. Whole grains and CVD risk. *Proc Nutr Soc* 2006;65:24-34.

73. Sherzai A, Heim LT, Boothby C, Sherzai AD. Stroke, food groups, and dietary patterns: A systematic review. *Nutrition Reviews* 2012;70:423-35.

74. Williams PG. Evaluation of the evidence between consumption of refined grains and health outcomes. *Nutrition Reviews* 2012;70:80-99.

75. Akesson A, Andersen LF, Kristjansdottir AG et al. Health effects associated with foods characteristic of the Nordic diet: a systematic literature review. *Food Nutr Res* 2013;57.

76. Lillioja S, Neal AL, Tapsell L, Jacobs DR, Jr. Whole grains, type 2 diabetes, coronary heart disease, and hypertension: links to the aleurone preferred over indigestible fiber. *Biofactors* 2013;39:242-58.

77. Frolich W, Aman P, Tetens I. Whole grain foods and health - A Scandinavian perspective. *Food and Nutrition Research* 2013;57.

78. Cho SS, Qi L, Fahey GC, Jr., Klurfeld DM. Consumption of cereal fiber, mixtures of whole grains and bran, and whole grains and risk reduction in type 2 diabetes, obesity, and cardiovascular disease. *Am J Clin Nutr* 2013;98:594-619.

79. Boffetta P, Thies F, Kris-Etherton P. Epidemiological studies of oats consumption and risk of cancer and overall mortality. *Br J Nutr* 2014;112 Suppl 2:S14-S18.

80. Flight I, Clifton P. Cereal grains and legumes in the prevention of coronary heart disease and stroke: a review of the literature. *Eur J Clin Nutr* 2006;60:1145-59.

81. Yu D, Sonderman J, Buchowski MS et al. Healthy Eating and Risks of Total and Cause-Specific Death among Low-Income Populations of African-Americans and Other Adults in the Southeastern United States: A Prospective Cohort Study. *PLoS Med* 2015;12:e1001830.

82. Holmberg S, Thelin A, Stiernstrom E-L. Food choices and coronary heart disease: A population based cohort study of rural Swedish men with 12 years of follow-up. *International Journal of Environmental Research and Public Health* 2009;6:2626-38.

**Table S3. Whole grains and refined grains and coronary heart disease**

| Author, publication year, country | Study name | Study period | Number of participants, gender, age, number of cases/deaths | CHD incidence or mortality | Dietary assessment | Exposure and subgroup | Whole grain consumption frequency or amount | Relative risks (95% confidence intervals) | Adjustment for confounding factors |
|---|---|---|---|---|---|---|---|---|---|
| Pietinen P et al, 1996, Finland | Alpha-Tocopherol Beta-Carotene Cancer Prevention Study | 1986-1987 -1993, 6.1 years follow-up | 21930 smoking men, age 50-69 years: 635 CHD deaths | Mortality | Validated FFQ, 276 food items | Rye products | 16 g/day<br>54.9<br>82.0<br>115.0<br>172.2 | 1.00<br>0.87 (0.68-1.10)<br>0.86 (0.68-1.10)<br>0.79 (0.61-1.01)<br>0.75 (0.58-0.98) | Age, treatment group, smoking, BMI, blood pressure, intake of energy, alcohol, SFA, education, physical activity |
| | | | | | | Other cereal products | 47.0<br>83.3<br>114.8<br>150.6<br>214.5 | 1.00<br>0.94 (0.73-1.21)<br>0.93 (0.72-1.21)<br>1.03 (0.79-1.34)<br>1.05 (0.79-1.40) | |
| Gartside PS et al, 1998, USA | National Health and Nutrition Examination Follow-up Study 1 | 1971-1987, 16 years follow-up | 5811 men and women, age 40-74 years: 1976 CHD cases | Incidence | NA | Bread | H vs l | 1.16, p=0.05 | Age, sex, race, geographic region, serum cholesterol, education, physical exercise, physical activity, smoking, BMI, alcohol, fish, dessert, cheese |
| Jacobs DR Jr et al, 1998, USA | Iowa Women's Health Study | 1986-1995, 8.64 years of follow-up | 34492 women, age 55-69 years: 438 ischemic heart disease cases | Incidence | FFQ, 127 food items | Dark bread | 0-0.5 serv/wk<br>1.0-3.0<br>5.5-7.0<br>17.5-42.0 | 1.00<br>0.81 (0.62-1.06)<br>0.62 (0.46-0.82)<br>0.67 (0.49-0.91) | Age, total energy, education, marital status, high blood pressure, diabetes, BMI, waist-to-hip ratio, physical activity, pack-years, alcohol, use of vitamin supplements, oral contraceptive use, HRT, Keys score, fruit and vegetable intake (except juice), red meat, fish and seafood, sucrose |
| | | | | | | Whole-grain breakfast cereal | 0 serv/wk<br>0.5-1.0<br>3.0<br>5.5-7.0 | 1.00<br>0.82 (0.62-1.08)<br>0.78 (0.58-1.06)<br>0.77 (0.56-1.04) | |
| | | | | | | Other whole grains | 0 serv/wk<br>0.5-1.0<br>1.5-5.0<br>5.5-91.0 | 1.00<br>1.43 (1.01-2.02)<br>1.17 (0.81-1.68)<br>1.26 (0.81-1.95) | |
| | | | | | | White bread | 0 serv/wk<br>0.5-1.0<br>3.0-5.5<br>7.0-42.0 | 1.00<br>0.90 (0.64-1.27)<br>1.43 (1.08-1.89)<br>1.24 (0.94-1.64) | |

| | | | | | | | | | |
|---|---|---|---|---|---|---|---|---|---|
| | | | | | | Refined-grain breakfast cereal | 0 serv/wk | 1.00 | |
| | | | | | | | 0.5-1.0 | 1.11 (0.81-1.53) | |
| | | | | | | | 3.0 | 0.82 (0.52-1.28) | |
| | | | | | | | 5.5-7.0 | 1.45 (0.99-2.13) | |
| | | | | | | Sweets or desserts | 0-2.0 serv/wk | 1.00 | |
| | | | | | | | 2.5-5.0 | 0.94 (0.70-1.26) | |
| | | | | | | | 5.5-7.0 | 1.13 (0.78-1.64) | |
| | | | | | | | 7.5-11.0 | 1.17 (0.81-1.67) | |
| | | | | | | | 11.5-143.0 | 0.86 (0.56-1.31) | |
| | | | | | | Other refined grains | 0-1.5 serv/wk | 1.00 | |
| | | | | | | | 2.0-3.0 | 0.94 (0.74-1.20) | |
| | | | | | | | 3.5-5.0 | 0.90 (0.65-1.25) | |
| | | | | | | | 5.5-50.0 | 0.79 (0.52-1.21) | |
| | | | | | | Crackers | 0 serv/wk | 1.00 | |
| | | | | | | | 0.5 | 0.91 (0.66-1.25) | |
| | | | | | | | 1.0 | 0.99 (0.71-1.40) | |
| | | | | | | | 3.0 | 0.87 (0.63-1.22) | |
| | | | | | | | 5.5-42.0 | 0.81 (0.57-1.14) | |

| | | | | | | | | | |
|---|---|---|---|---|---|---|---|---|---|
| Liu S et al, 1999, USA | Nurses' Health Study | 1984-1994, 10 years follow-up | 75521 women, age 38-63 years: 761 CHD cases | Incidence | Validated FFQ, 126 food items | Whole grain | 0.13 serv/day<br>0.43<br>0.85<br>1.31<br>2.70 | 1.00<br>0.92 (0.75-1.14)<br>0.93 (0.75-1.15)<br>0.83 (0.66-1.05)<br>0.75 (0.59-0.95) | Age, BMI, cigarette smoking, alcohol, parental or family history of myocardial infarction before age 60, hypertension, hypercholesterolemia, menopausal status, HRT, protein intake, aspirin use, multiple/vitamin E use, vigorous activity, total energy, SFA, PUFA, MUFA, trans-FA |
| | | | | | | Dark bread | 0 serv/day<br>0.07<br>0.43<br>0.71<br>1.30 | 1.00<br>1.13 (0.89-1.43)<br>0.92 (0.72-1.18)<br>0.97 (0.74-1.29)<br>0.98 (0.77-1.25) | |
| | | | | | | Whole-grain breakfast cereal | 0 serv/day<br>0.07<br>0.22<br>0.43<br>0.93 | 1.00<br>0.89 (0.72-1.10)<br>0.72 (0.56-0.92)<br>0.82 (0.66-1.02)<br>0.76 (0.57-1.00) | |
| | | | | | | Popcorn | 0 serv/day<br>0.07<br>0.33<br>0.62<br>1.00 | 1.00<br>0.86 (0.74-1.01)<br>0.88 (0.66-1.18)<br>0.42 (0.18-0.92)<br>0.92 (0.45-1.87) | |
| | | | | | | Cooked oatmeal | 0 serv/day<br>0.07<br>0.33<br>0.67<br>1.00 | 1.00<br>0.92 (0.79-1.08)<br>0.70 (0.49-0.98)<br>1.41 (0.75-2.26)<br>1.10 (0.45-2.68) | |
| | | | | | | Brown rice | 0 serv/day<br>0.07<br>0.31<br>0.79 | 1.00<br>0.86 (0.72-1.02)<br>0.77 (0.45-1.32)<br>0.45 (0.06-3.20) | |
| | | | | | | Wheat germ | 0 serv/day<br>0.07<br>0.36<br>0.93 | 1.00<br>0.55 (0.37-0.81)<br>1.11 (0.64-1.93)<br>0.41 (0.15-1.10) | |
| | | | | | | Bran | 0 serv/day<br>0.07<br>0.36<br>1.00 | 1.00<br>0.76 (0.59-0.98)<br>0.68 (0.44-1.03)<br>0.63 (0.42-0.95) | |
| | | | | | | Other grains | 0 serv/day<br>0.07 | 1.00<br>0.79 (0.57-1.08) | |

| | | | | | | | | | |
|---|---|---|---|---|---|---|---|---|---|
| Jacobs DR et al, 2001, Norway | Norwegian County Study | 1977-1983 – 1994, 14.4 years follow-up | 33848 men and women, age 35-56 years: 553 CHD deaths | Mortality | FFQ, 66 food items | Whole grain bread score | 0.05-0.60<br>0.83-0.83<br>0.90-1.13<br>1.35-1.80<br>2.25-5.40 | 1.00<br>0.99 (0.75-1.31)<br>0.94 (0.73-1.22)<br>0.88 (0.67-1.16)<br>0.76 (0.56-1.02) | Age, energy intake, sex, smoking status, physical activity during leisure, physical activity during work, cod liver oil, multivitamin use, SFA, SBP, serum total cholesterol, BMI |
| Appleby PN et al, 2002, UK | The Health Food Shoppers Study | 1973-1979 – 1997, 19.8 years follow-up | 10741 men and women, age 16-89 years: 605 ischemic heart disease deaths | Mortality | FFQ | Wholemeal bread<br>Bran cereals | Daily vs less<br>Daily vs less | 0.86 (0.72-1.03)<br>1.13 (0.94-1.35) | Age at recruitment, sex, smoking, fresh fruit, nuts/dried fruit, raw vegetables salads, mutual adjustment: wholemeal bread and bran cereals |
| Liu S et al, 2003, USA | Physicians' Health Study | 1982-1988, 5.5 years follow-up | 86190 men, age 40-84 years: 488 MI cases | Incidence | Validated FFQ | Whole grain breakfast cereals<br><br><br><br>Refined grain breakfast cereals<br><br><br><br>Total breakfast cereals | Rarely<br>1 serv/wk<br>2-6/wk<br>≥1/day<br>Rarely<br>1 serv/wk<br>2-6/wk<br>≥1/day<br>Rarely<br>1 serv/wk<br>2-6/wk<br>≥1/day | 1.00<br>0.83 (0.56-1.23)<br>0.79 (0.56-1.10)<br>0.71 (0.51-0.98)<br>1.00<br>1.08 (0.81-144)<br>1.05 (0.76-1.44)<br>0.96 (0.68-1.36)<br>1.00<br>0.87 (0.67-1.14)<br>0.84 (0.62-1.07)<br>0.76 (0.54-0.94) | Age, cigarette smoking, alcohol intake, physical activity, BMI, history of type 2 diabetes, high cholesterol, hypertension, use of multivitamins |
| Steffen LM et al, 2003, USA | Atherosclerosis Risk in Communities Study | 1987-1989 - 1999, 11 years follow-up | 11940 men and women, age 45-64 years: 535 fatal or nonfatal coronary artery disease cases | Incidence | Validated FFQ, 61 food items | Whole grain<br><br><br><br><br>Refined grain | 0.1 serv/day<br>0.5<br>1.0<br>1.5<br>3.0<br>0.5 serv/day<br>1.5<br>2.0<br>3.0<br>5.0 | 1.00<br>0.76 (0.58-0.99)<br>0.93 (0.72-1.21)<br>0.73 (0.55-0.98)<br>0.72 (0.53-0.97)<br>1.00<br>0.91 (0.65-1.27)<br>1.14 (0.83-1.56)<br>1.28 (0.93-1.75)<br>1.17 (0.82-1.66) | Age, race, sex, time-dependent energy intake, education, smoking status, pack-years of smoking, physical activity, alcohol intake, HRT (women), BMI, waist-to-hip ratio, SBP, antihypertensive medication use, HDL-cholesterol, LDL-cholesterol |
| Jensen MK et al, 2004, USA | Health Professionals Follow-up Study | 1986-2000, 14 years follow-up | 42850 men, age 40-75 years: 1818 CHD cases | Incidence | Validated FFQ, 131 food items | Whole grains<br><br><br><br><br>Added bran | 3.5 g/d<br>9.6<br>16.0<br>24.7<br>42.4<br>0 g/d | 1.00<br>0.96 (0.83-1.10)<br>0.94 (0.81-1.09)<br>0.86 (0.74-1.01)<br>0.84 (0.71-0.98)<br>1.00 | Age, energy, smoking, alcohol, physical activity, family history of MI, use of vitamin E supplements, SFA, PUFA, trans FA, fruit, vegetables, fish, BMI, mutual adjustment between |

| | | | | | | | | | |
|---|---|---|---|---|---|---|---|---|---|
| | | | | | | <br><br><br><br>Germ | 0.30<br>1.40<br>4.23<br>11.10<br>0 g/d<br>0.20<br>0.83 | 0.81 (0.70-0.95)<br>0.79 (0.67-0.92)<br>0.80 (0.68-0.93)<br>0.72 (0.61-0.84)<br>1.00<br>0.93 (0.81-1.07)<br>0.98 (0.85-1.12) | whole grains, added bran, and added germ |
| Mink PJ et al, 2007, USA | Iowa Women's Health Study | 1986-2002, 16 years follow-up | 34489 women, age 55-69 years: 1329 CHD deaths | Mortality | FFQ, 127 food items | Bran, added to food | 0 serv/wk<br>・0 | 1.00<br>0.91 (0.78-1.06) | Age, energy intake, marital status, education, blood pressure, diabetes, BMI, waist-to-hip ratio, physical activity, smoking, HRT |
| Jacobs DR, 2007, USA | Iowa Women's Health Study | 1986 – 2003, 17 years follow-up | 27312 women, age 55-69 years: 1034 CHD deaths | Mortality | FFQ, 127 food items | Whole grains<br><br><br><br><br>Refined grains | 1.8 serv/wk<br>5.6<br>8.8<br>14.5<br>25.6<br>0-5.75 serv/wk<br>6-9.5<br>9.6-13.5<br>14-22<br>≥22.5 | 1.00<br>1.01 (0.84-1.22)<br>0.85 (0.70-1.04)<br>0.79 (0.64-0.97)<br>0.72 (0.57-0.90)<br>1.00<br>0.96 (0.79-1.16)<br>0.90 (0.74-1.11)<br>0.80 (0.64-0.99)<br>0.89 (0.70-1.14) | Age, energy intake, BMI, waist-to-hip ratio, smoking, education, physical activity, estrogen use, multivitamin supplement use, intake of alcohol, alcohol$^2$, coffee, red meat, fish and seafood, total fruit and vegetables, mutual adjustment between whole grains and refined grains |
| Buckland G et al, 2009, Spain | European Prospective Investigation into Cancer and Nutrition – Spain | 1992-1996 – 2004, 10.4 years follow-up | 41078 men and women, age 29-69 years: 609 CHD cases | Incidence | Dietary history interview, validated FFQ, ~600 foods | Cereals | 0-72.5 g/d<br>>72.5-102.6<br>>102.6-501.3 | 1.00<br>1.01 (0.83-1.24)<br>1.12 (0.92-1.38) | Age, sex, center, education, physical activity, BMI, smoking status, diabetes, hypertension, hyperlipidemia, total calories |
| Eshak ES et al, 2011, Japan | Japan Collaborative Cohort Study | 1988-1990 – 2003, 14.1 years follow-up | 83752 men and women, age 40-79 years: 707 CHD deaths | Mortality | Validated FFQ, 40 food items | Rice, men<br><br><br><br><br>Rice, women | 280 g/d<br>420<br>449<br>583<br>711<br>279 g/d<br>359<br>420<br>453<br>560 | 1.00<br>1.04 (0.79-1.37)<br>0.73 (0.51-1.05)<br>0.85 (0.60-1.19)<br>0.70 (0.49-0.99)<br>1.00<br>1.01 (0.64-1.59)<br>0.83 (0.57-1.21)<br>1.25 (0.74-2.10)<br>1.08 (0.66-1.77) | Age, history of hypertension, history of diabetes, BMI, alcohol, smoking status, exercise, walking, education, perceived mental stress, sleep duration, fish, meat, fruit, dairy products, soy, total energy, sodium, Key's dietary score |

| | | | | | | | | | |
|---|---|---|---|---|---|---|---|---|---|
| Rautiainen S et al, 2012, Sweden | Swedish Mammmography Cohort | 1997-2007, 9.9 years follow-up | 32561 women, age 49-83 years: 1114 MI cases | Incidence | Validated FFQ, 96 food items | Whole grains | ≤2.3 serv/d<br>2.4-3.4<br>3.4-4.7<br>≥4.7 | 1.00<br>0.95 (0.81-1.13)<br>0.88 (0.74-1.04)<br>0.89 (0.74-1.07) | Age, education, smoking, BMI, physical activity, hypertension, hypercholesterolemia, family history – MI, aspirin use, HRT, dietary supplement use, total energy, alcohol |
| Simila ME et al, 2013, Finland | Alpha-Tocopherol Beta-Carotene Cancer Prevention Study | 1985-1988 – 2004, 19 years follow-up | 21995 male smokers, age 50-69 years: 4379 CHD cases | Incidence | Validated FFQ, 276 food items | Rye | Per 100 g/d | 0.99 (0.94-1.03) | Age, intervention group |
| Yu D et al, 2013, China | Shanghai Women's Health Study | 1997-2000 – 2009, 9.8 years follow-up | 64854 women, age 40-70 years: 120 CHD cases | Incidence | Validated FFQ, 77 food items | White rice and refined wheat products | 250 g/d<br>274<br>290<br>311 | 1.00<br>0.97 (0.49-1.93)<br>1.41 (0.69-2.90)<br>1.53 (0.64-3.68) | Age, birth cohort, education, income, smoking status, alcohol, physical activity, waist-to-hip ratio, hypertension, total energy, SFA, protein |
| Yu D et al, 2013, China | Shanghai Men's Health Study | 2002-2006 – 2009, 5.4 years follow-up | 52512 men, age 40-74 years: 189 CHD cases | Incidence | Validated FFQ, 81 food items | White rice and refined wheat products | 253 g/d<br>290<br>327<br>367 | 1.00<br>1.15 (0.69-1.90)<br>1.38 (0.76-2.51)<br>2.01 (0.96-4.23) | Age, birth cohort, education, income, smoking status, alcohol, physical activity, waist-to-hip ratio, hypertension, total energy, SFA, protein |
| Eshak ES et al, 2014, Japan | Japan Public Health Center-based Prospective Study | 1990/1993 – 2007/2009, 15.2 years follow-up | 91223 men and women, age 40-69 years: 1088 IHD cases<br>605 IHD deaths | Incidence and mortality | Validated FFQ, 44/52 food items | Rice, CHD incidence<br><br><br><br><br>Rice, CHD mortality | 251 g/d<br>326<br>377<br>430<br>542<br>251 g/d<br>326<br>377<br>430<br>542 | 1.00<br>0.93 (0.76-1.14)<br>0.99 (0.80-1.22)<br>0.95 (0.77-1.19)<br>1.08 (0.84-1.38)<br>1.00<br>0.81 (0.61-1.06)<br>0.93 (0.70-1.23)<br>0.85 (0.64-1.12)<br>0.93 (0.68-1.27) | Age, sex, public health center area, hypertension, diabetes, use of lipid-lowering drugs, BMI, smoking status, ethanol intake, leisure-time sports activity, occupation, seafood, meat, fruit, vegetables, soy, SFAs, sodium, total energy, women: HRT, menopausal status |
| Rebello SA et al, 2014, Singapore | Singapore Chinese Health Study | 1993-1998 – 2011, 15 years follow-up | 53469 men and women, age 45-74 years: 1660 IHD deaths | Mortality | Validated FFQ, 165 food items | Whole-wheat bread, men<br><br><br><br>Whole-wheat bread, women<br><br><br><br>White bread, men | 0.00 slices/d<br>0.33<br>1.00<br>Per serv/d<br>0.00 slices/d<br>0.33<br>1.00<br>Per serv/d<br>0.00 slices/d | 1.00<br>0.93 (0.77-1.12)<br>0.94 (0.66-1.33)<br>0.99 (0.78-1.27)<br>1.00<br>0.93 (0.74-1.17)<br>0.51 (0.30-0.89)<br>0.58 (0.38-0.89)<br>1.00 | Age, year of interview, father's dialect, total energy intake, cigarette smoking, alcohol, physical activity, sleep duration, education, BMI, hypertension, PUFA/SFA ratio, rice, noodles, vegetables, fruit, fish, red meat, poultry, eggs, legumes, soy protein, white bread and whole- |

| | | | | | | | | | |
|---|---|---|---|---|---|---|---|---|---|
| | | | | | | | 0.33 | 1.09 (0.92-1.29) | wheat bread mutually adjusted, |
| | | | | | | | 1.00 | 1.12 (0.90-1.39) | women: menopausal status, |
| | | | | | | | Per serv/d | 0.91 (0.79-1.05) | HRT use |
| | | | | | | White bread, women | 0.00 slices/d | 1.00 | |
| | | | | | | | 0.33 | 0.91 (0.74-1.11) | |
| | | | | | | | 1.00 | 0.79 (0.60-1.04) | |
| | | | | | | | Per serv/d | 0.84 (0.68-1.04) | |
| | | | | | | Rice, men | 2.35 serv/d | 1.00 | |
| | | | | | | | 3.40 | 0.96 (0.76-1.20) | |
| | | | | | | | 4.10 | 0.95 (0.75-1.20) | |
| | | | | | | | 4.80 | 0.98 (0.77-1.25) | |
| | | | | | | | 6.74 | 1.02 (0.79-1.31) | |
| | | | | | | | Per serv/d | 1.00 (0.95-1.06) | |
| | | | | | | Rice, women | 2.40 serv/d | 1.00 | |
| | | | | | | | 3.39 | 1.07 (0.79-1.45) | |
| | | | | | | | 4.08 | 1.20 (0.88-1.63) | |
| | | | | | | | 4.80 | 1.07 (0.77-1.48) | |
| | | | | | | | 5.77 | 1.10 (0.77-1.58) | |
| | | | | | | | Per serv/d | 1.00 (0.92-1.08) | |
| | | | | | | Noodles, men | 0.11 serv/d | 1.00 | |
| | | | | | | | 0.31 | 0.96 (0.79-1.18) | |
| | | | | | | | 0.47 | 1.07 (0.87-1.31) | |
| | | | | | | | 0.66 | 1.19 (0.96-1.46) | |
| | | | | | | | 1.08 | 1.32 (1.07-1.62) | |
| | | | | | | | Per serv/d | 1.30 (1.11-1.53) | |
| | | | | | | Noodles, women | 0.15 serv/d | 1.00 | |
| | | | | | | | 0.32 | 1.01 (0.77-1.33) | |
| | | | | | | | 0.46 | 0.95 (0.71-1.27) | |
| | | | | | | | 0.65 | 1.14 (0.86-1.53) | |
| | | | | | | | 1.07 | 1.38 (1.02-1.85) | |
| | | | | | | | Per serv/d | 1.38 (1.09-1.75) | |
| Muraki I et al, 2014, USA | Nurses' Health Study | 1984-2010, 26 years follow-up | 73228 women, age 38-63 years: 3060 CAD cases | Incidence | Validated FFQ, 118-166 food items | White rice | <1 serv/wk | 1.00 | Age, sex, cohort, ethnicity, BMI, smoking status, cigarettes per day, alcohol, physical activity, family history – MI, multivitamin use, current aspirin use, prevalent hypertension, prevalent hypercholesterolemia, prevalent diabetes, total energy |
| | | | | | | | 1 | 1.04 (0.98-1.09) | |
| | | | | | | | 2-4 | 1.05 (0.98-1.12) | |
| | | | | | | | ≥5 | 0.84 (0.69-1.02) | |
| | | | | | | | Per 3 serv/wk | 1.01 (0.94-1.07) | |
| | Nurses' Health Study 2 | 1991-2011, 20 years | 92158 women, age 27-44 years: 534 CAD cases | | | Brown rice | <1 serv/wk | 1.00 | |
| | | | | | | | 1 | 0.99 (0.92-1.06) | |
| | | | | | | | 2-4 | 1.05 (0.95-1.16) | |

| | | | | | | | | | |
|---|---|---|---|---|---|---|---|---|---|
| | <br><br>Health Professionals Follow-up Study | follow-up<br><br>1986-2010, 24 years follow-up | <br><br>42170 men, age 40-75 years: 4125 CAD cases | | | <br><br>Total rice | ≥5<br>Per 3 serv/wk<br><1 serv/wk<br>1<br>2-4<br>≥5<br>Per 3 serv/wk | 0.80 (0.57-1.12)<br>1.00 (0.91-1.11)<br>1.00<br>1.03 (0.98-1.09)<br>1.03 (0.97-1.09)<br>0.97 (0.86-1.08)<br>0.99 (0.94-1.05) | intake, modified alternate Healthy Eating Index score, women: menopausal status, oral contraceptive use (NHS2), postmenopausal hormone use |
| Tognon G et al, 2014, Denmark | The 1982-83 Danish Monitoring trends and determinants of Cardiovascular disease study (MONICA) | 1982-1983 – 2007, 14 years follow-up | 948 women and 901 men, age NA: 161 MI cases<br>64 MI deaths | Incidence and mortality | Validated 7 day food record, 100 food items | Cereals, MI incidence<br>Cereals, MI death | >median vs. <median<br>>median vs. <median | 0.90 (0.66-1.24)<br>0.69 (0.41-1.16) | Age, sex, BMI, education, physical activity, cigarette smoking |
| Atkins JL et al, 2014, United Kingdom | British Regional Heart Study | 1998-2000 – 2010, 11.3 years follow-up | 3328 men, age 60-79 years: 307 CHD cases | Incidence | Validated FFQ, 86 food items | Cereals<br>Bread | Daily vs. <1 day/wk<br>Whole grain vs. none | 1.19 (0.73-1.93)<br>0.76 (0.18-3.15) | Age, smoking, alcohol, physical activity, social class, BMI, energy intake, diet score without respective components |
| Johnsen NF et al, 2015, Norway, Sweden, Denmark | HELGA Cohort (Norwegian Women and Cancer Study, Northern Sweden Health and Disease Study, Danish Diet, Cancer and Health Study – part of the EPIC study) | 1992-1998 – 2008-2009, 11.1 (Norway), 14.2 (Sweden), 11.9 years (Denmark) follow-up | 120010 men and women, age 30-64 years: 858/298 CHD deaths | Mortality | Validated FFQ, 88 food items (Norway), 98 food items (Sweden) 173 food items (Denmark) | Whole grain breakfast cereals, women<br><br><br><br>Non-white bread<br><br><br><br>Crisp bread<br><br><br><br>Total whole grain products<br><br><br>Oat | 0 g/d<br>0.8<br>12<br>50<br>25 g/d<br>80<br>113<br>180<br>0.6 g/d<br>2<br>6<br>31<br>56 g/d<br>100<br>131<br>201<br>0 g/d<br>0.4<br>4<br>19<br>8 g/d | 1.00<br>0.71 (0.43-1.16)<br>0.64 (0.47-0.87)<br>0.53 (0.37-0.77)<br>1.00<br>0.72 (0.52-0.99)<br>0.54 (0.37-0.79)<br>0.72 (0.47-1.09)<br>1.00<br>0.81 (0.56-1.18)<br>0.95 (0.68-1.33)<br>0.94 (0.66-1.34)<br>1.00<br>0.72 (0.54-0.97)<br>0.64 (0.46-0.89)<br>0.56 (0.40-0.78)<br>1.00<br>0.74 (0.52-1.05)<br>0.85 (0.59-1.21)<br>0.66 (0.45-0.96)<br>1.00 | Age, follow-up time, education, smoking status/years since quit/ cigarettes per day, alcohol, BMI, total energy |

| | | | | | | | | | |
|---|---|---|---|---|---|---|---|---|---|
| | | | | | | Rye | 18 | 0.79 (0.57-1.11) | |
| | | | | | | | 22 | 1.02 (0.75-1.38) | |
| | | | | | | | 41 | 0.69 (0.46-1.05) | |
| | | | | | | | 0.4 g/d | 1.00 | |
| | | | | | | Wheat | 3 | 0.83 (0.57-1.21) | |
| | | | | | | | 10 | 0.87 (0.60-1.27) | |
| | | | | | | | 37 | 0.67 (0.45-1.00) | |
| | | | | | | | 20 g/d | 1.00 | |
| | | | | | | Total whole grain | 33 | 0.83 (0.60-1.14) | |
| | | | | | | types | 49 | 0.59 (0.42-0.82) | |
| | | | | | | | 74 | 0.65 (0.46-0.91) | |
| | | | | | | | 0 g/d | 1.00 | |
| | | | | | | | 0.8 | 0.88 (0.69-1.11) | |
| | | | | | | Whole grain | 7 | 0.87 (0.72-1.05) | |
| | | | | | | breakfast cereals, | 50 | 0.75 (0.61-0.91) | |
| | | | | | | men | 13 g/d | 1.00 | |
| | | | | | | | 66 | 1.13 (0.89-1.42) | |
| | | | | | | | 118 | 0.95 (0.74-1.22) | |
| | | | | | | Non-white bread | 201 | 0.87 (0.65-1.17) | |
| | | | | | | | 1 g/d | 1.00 | |
| | | | | | | | 2 | 0.98 (0.81-1.19) | |
| | | | | | | | 4 | 0.95 (0.78-1.16) | |
| | | | | | | Crisp bread | 34 | 1.23 (0.88-1.71) | |
| | | | | | | | 64 g/d | 1.00 | |
| | | | | | | | 107 | 0.94 (0.78-1.15) | |
| | | | | | | | 156 | 0.80 (0.66-0.97) | |
| | | | | | | Total whole grain | 222 | 0.85 (0.70-1.04) | |
| | | | | | | products | 0 g/d | 1.00 | |
| | | | | | | | 0.4 | 1.01 (0.83-1.23) | |
| | | | | | | | 3 | 1.01 (0.80-1.28) | |
| | | | | | | Oat | 30 | 0.82 (0.65-1.03) | |
| | | | | | | | 7 g/d | 1.00 | |
| | | | | | | | 21 | 0.77 (0.62-0.95) | |
| | | | | | | | 38 | 0.81 (0.67-0.97) | |
| | | | | | | Rye | 56 | 0.88 (0.73-1.07) | |
| | | | | | | | 0.1 g/d | 1.00 | |
| | | | | | | | 1 | 0.80 (0.66-0.97) | |
| | | | | | | | 5 | 0.70 (0.55-0.88) | |
| | | | | | | Wheat | 10 | 0.84 (0.65-1.08) | |

| | | | | | | | | | |
|---|---|---|---|---|---|---|---|---|---|
| | | | | | | Total whole grain types | 21 g/d<br>37<br>54<br>80 | 1.00<br>0.79 (0.65-0.96)<br>0.80 (0.66-0.97)<br>0.74 (0.61-0.91) | |
| Helnæs A et al, 2016, Denmark | Danish Diet, Cancer and Health Cohort | 1993-1997 - 2009, 13.6 years follow-up | 54871 men and women, age 50-64 years: 2329 MI cases | Incidence | Validated FFQ, 192 food items | Total whole grain products, men | 66 g/d<br>116<br>163<br>224<br>Per 50 g/d | 1.00<br>0.83 (0.73-0.95)<br>0.81 (0.71-0.93)<br>0.77 (0.67-0.89)<br>0.94 (0.91-0.97) | Age, education, smoking status, pack-years, alcohol from beer and spirits, alcohol from wine, sports, menopausal status, HRT, fruit, vegetables, fish, red meat, processed meat |
| | | | | | | Total whole grains | 22 g/d<br>38<br>52<br>74<br>Per 25 g/d | 1.00<br>0.84 (0.73-0.95)<br>0.86 (0.76-0.99)<br>0.75 (0.65-0.86)<br>0.88 (0.83-0.93) | |
| | | | | | | Total whole grain products, women | 63 g/d<br>103<br>135<br>201<br>Per 50 g/d | 1.00<br>0.83 (0.67-1.02)<br>0.78 (0.63-0.96)<br>0.71 (0.56-0.89)<br>0.90 (0.84-0.97) | |
| | | | | | | Total whole grains | 20 g/d<br>29<br>42<br>63<br>Per 25 g/d | 1.00<br>0.86 (0.70-1.06)<br>0.77 (0.62-0.95)<br>0.73 (0.58-0.91)<br>0.87 (0.78-0.96) | |
| | | | | | | Rye bread, men | Per 25 g/d | 0.96 (0.94-0.99) | + BMI, waist circumference, SBP, hypertension, serum cholesterol, hypercholesterolemia |
| | | | | | | Whole grain bread | Per 25 g/d | 0.99 (0.97-1.02) | |
| | | | | | | Oatmeal | Per 25 g/d | 0.84 (0.78-0.91) | |
| | | | | | | Crispbread | Per 25 g/d | 0.97 (0.81-1.17) | |
| | | | | | | Wheat | Per 10 g/d | 0.96 (0.84-1.09) | |
| | | | | | | Rye | Per 10 g/d | 0.96 (0.93-0.99) | |
| | | | | | | Oats | Per 10 g/d | 0.93 (0.88-0.97) | |
| | | | | | | Rye bread, women | Per 25 g/d | 0.93 (0.88-0.98) | |
| | | | | | | Whole grain bread | Per 25 g/d | 0.98 (0.93-1.03) | |
| | | | | | | Oatmeal | Per 25 g/d | 0.89 (0.77-1.02) | |
| | | | | | | Crispbread | Per 25 g/d | 1.12 (0.92-1.36) | |

| | | | | | | | | | |
|---|---|---|---|---|---|---|---|---|---|
| | | | | | | Wheat<br>Rye<br>Oats<br>Total whole grain products, men<br><br><br><br><br>Total whole grains<br><br><br><br><br>Total whole grain products, women<br><br><br><br><br>Total whole grains<br><br><br><br><br>Rye bread, men<br>Whole grain bread<br>Oatmeal<br>Crispbread<br>Wheat<br>Rye<br>Oats<br>Rye bread, women<br>Whole grain bread<br>Oatmeal<br>Crispbread<br>Wheat<br>Rye<br>Oats | Per 10 g/d<br>Per 10 g/d<br>Per 10 g/d<br>66 g/d<br>116<br>163<br>224<br>Per 50 g/d<br>22 g/d<br>38<br>52<br>74<br>Per 25 g/d<br>63 g/d<br>103<br>135<br>201<br>Per 50 g/d<br>20 g/d<br>29<br>42<br>63<br>Per 25 g/d<br>Per 25 g/d<br>Per 25 g/d<br>Per 25 g/d<br>Per 25 g/d<br>Per 10 g/d<br>Per 10 g/d<br>Per 10 g/d<br>Per 25 g/d<br>Per 25 g/d<br>Per 25 g/d<br>Per 25 g/d<br>Per 10 g/d<br>Per 10 g/d<br>Per 10 g/d | 0.89 (0.70-1.13)<br>0.96 (0.90-1.01)<br>0.94 (0.86-1.02)<br>1.00<br>0.85 (0.75-0.98)<br>0.85 (0.74-0.98)<br>0.89 (0.77-1.02)<br>0.97 (0.94-1.01)<br>1.00<br>0.87 (0.76-0.99)<br>0.93 (0.82-1.07)<br>0.88 (0.76-1.02)<br>0.94 (0.88-0.99)<br>1.00<br>0.82 (0.66-1.02)<br>0.80 (0.64-0.98)<br>0.72 (0.57-0.91)<br>0.91 (0.85-0.98)<br>1.00<br>0.84 (0.68-1.04)<br>0.79 (0.64-0.98)<br>0.76 (0.61-0.96)<br>0.89 (0.80-0.99)<br>0.98 (0.96-1.01)<br>1.01 (0.98-1.03)<br>0.89 (0.82-0.96)<br>0.93 (0.77-1.11)<br>1.03 (0.90-1.17)<br>0.98 (0.95-1.00)<br>0.96 (0.91-1.00)<br>0.93 (0.88-0.98)<br>0.98 (0.93-1.02)<br>0.92 (0.80-1.05)<br>1.12 (0.92-1.36)<br>0.90 (0.71-1.14)<br>0.96 (0.91-1.01)<br>0.96 (0.88-1.05) | |
| Wang JB et al, 2016, China | Linxian Nutrition Intervention Trial | 1984-1991 - 2010, 19- | 2445 men and women, age 40- | Mortality | FFQ, 64 food items | All grains<br>Non-whole grains | Per 1 time/day<br>Per 1 time/day | 1.00 (0.92-1.09)<br>1.05 (0.94-1.16) | Age, sex, commune, smoking, drinking, season, BMI |

| | | | | | | | | | |
|---|---|---|---|---|---|---|---|---|---|
| | cohort | 26 years follow-up | 69 years: 355 heart disease deaths | | | Whole grains | Per 1 time/day | 0.94 (0.83-1.07) | |

BMI; body mass index, CHD; coronary heart disease, FFQ; food frequency questionnaire, H vs l; High vs low, HDL-cholesterol; high-density lipoprotein cholesterol, HRT; hormone replacement therapy, IHD; ischemic heart disease, LDL; low density lipoprotein cholesterol, MI; myocardial infarction, MUFA; monounsaturated fatty acids, NHS2; Nurses' Health Study 2, PUFA; polyunsaturated fatty acids, SBP; systolic blood pressure, SFA; saturated fatty acids, trans-FA: trans fatty acids

**Table S4. Whole grains and refined grains and stroke**

| Author, publication year, country | Study name | Study period | Number of participants, gender, age, number of cases/deaths | Stroke incidence or mortality | Dietary assessment | Exposure and subgroup | Whole grain consumption frequency or amount | Relative risks (95% confidence intervals) | Adjustment for confounding factors |
|---|---|---|---|---|---|---|---|---|---|
| Liu S et al, 2000, USA | Nurses' Health Study | 1984-1996, 12 years follow-up | 75521 women, age 38-63 years: 352 ischemic stroke cases | Incidence | Validated FFQ, 126 food items | Whole grains<br><br><br><br><br>Refined grains<br><br><br><br><br>Total grains | 0.13 serv/day<br>0.43<br>0.85<br>1.31<br>2.70<br>1<br>2<br>3<br>4<br>5<br>1<br>2<br>3<br>4<br>5 | 1.00<br>0.72 (0.53-1.00)<br>0.78 (0.58-1.08)<br>0.60 (0.43-0.86)<br>0.69 (0.50-0.98)<br>1.00<br>1.11 (0.81-1.52)<br>1.18 (0.85-1.64)<br>0.94 (0.66-1.35)<br>0.97 (0.67-1.42)<br>1.00<br>0.83 (0.60-1.15)<br>0.82 (0.59-1.16)<br>0.62 (0.43-0.92)<br>0.79 (0.54-1.18) | Age, BMI, physical activity, cigarette smoking, alcohol, parental history of MI before age 60 years, aspirin use, menopausal status, HRT, hypertension, high blood cholesterol, use of multivitamins and vitamin E supplements, SFA, Trans FA, total energy |
| Appleby PN et al, 2002, UK | The Health Food Shoppers Study | 1973-1979 – 1997, 19.8 years follow-up | 10741 men and women, age 16-89 years: 356 cerebro-vascular disease deaths | Mortality | FFQ | Wholemeal bread<br>Bran cereals | Daily vs less<br>Daily vs less | 0.89 (0.70-1.13)<br>0.92 (0.73-1.17) | Age, sex, smoking, fresh fruit, nuts/dried fruit, raw vegetables salads, mutual adjustment between wholemeal bread and bran cereals |
| Liu S et al, 2003, USA | Physicians' Health Study | 1982-1988, 5.5 years follow-up | 86190 men, age 40-84 years: 146 stroke cases | Incidence | Validated FFQ | Whole grain breakfast cereals<br><br><br><br>Refined-grain breakfast cereals<br><br><br><br>Total breakfast cereals | Rarely<br>1 serv/wk<br>2-6/wk<br>≥1/day<br>Rarely<br>1 serv/wk<br>2-6/wk<br>≥1/day<br>Rarely<br>1 serv/wk<br>2-6/wk<br>≥1/day | 1.00<br>0.92 (0.42-1.99)<br>1.68 (1.00-2.80)<br>1.41 (0.85-2.34)<br>1.00<br>0.79 (0.43-1.45)<br>0.79 (0.42-1.49)<br>1.22 (0.71-2.11)<br>1.00<br>0.98 (0.56-1.74)<br>1.40 (0.84-2.32)<br>1.54 (0.94-2.52) | Age, cigarette smoking, alcohol intake, physical activity, BMI, history of type 2 diabetes mellitus, high cholesterol, hypertension, use of multivitamins |

| | | | | | | | | | |
|---|---|---|---|---|---|---|---|---|---|
| Steffen LM et al, 2003, USA | Atherosclerosis Risk in Communities Study | 1987-1989 - 1999, 11 years follow-up | 15792 men and women, age 45-64 years: 214 fatal or incident ischemic stroke | Incidence | Validated FFQ, 61 food items | Whole grain<br><br><br><br><br>Refined grain | 0.1 serv/day<br>0.5<br>1.0<br>1.5<br>3.0<br>0.5 serv/day<br>1.5<br>2.0<br>3.0<br>5.0 | 1.00<br>1.11 (0.75-1.64)<br>0.79 (0.50-1.21)<br>0.89 (0.57-1.39)<br>0.75 (0.46-1.22)<br>1.00<br>1.10 (0.71-1.73)<br>1.00 (0.63-1.58)<br>0.68 (0.41-1.13)<br>0.82 (0.48-1.40) | Age at baseline, race, sex, time-dependent energy intake, education, smoking status, pack-years of smoking, physical activity, alcohol intake, HRT (women), BMI, waist-to-hip ratio, SBP, antihypertensive medication use |
| Jacobs DR, 2007, USA | Iowa Women's Health Study | 1986 – 2003, 17 years follow-up | 27312 women, age 55-69 years:<br>414 stroke deaths<br>113 intracranial hemorrhagic stroke deaths<br>251 non-hemorrhagic stroke deaths | Mortality | Validated FFQ, 127 food items | Whole grains, all<br><br><br><br><br>Whole grains, hemorrhagic strokes<br><br><br><br>Whole grains, non-hemorrhagic strokes<br><br><br><br>Refined grains, all<br><br><br><br><br>Refined grains, hemorrhagic strokes<br><br><br><br>Refined grains, non-hemorrhagic strokes | 1.8 serv/wk<br>5.6<br>8.8<br>14.5<br>25.6<br>1.8 serv/wk<br>5.6<br>8.8<br>14.5<br>25.6<br>1.8 serv/wk<br>5.6<br>8.8<br>14.5<br>25.6<br>0-5.75 serv/wk<br>6-9.5<br>9.6-13.5<br>14-22<br>≥22.5<br>0-5.75 serv/wk<br>6-9.5<br>9.6-13.5<br>14-22<br>≥22.5<br>0-5.75 serv/wk<br>6-9.5<br>9.6-13.5<br>14-22 | 1.00<br>0.91 (0.67-1.24)<br>0.84 (0.61-1.15)<br>0.88 (0.64-1.22)<br>0.85 (0.60-1.21)<br>1.00<br>1.28 (0.68-2.42)<br>1.33 (0.71-2.49)<br>1.01 (0.51-1.99)<br>1.28 (0.64-2.56)<br>1.00<br>0.79 (0.53-1.18)<br>0.74 (0.49-1.11)<br>0.87 (0.58-1.31)<br>0.88 (0.57-1.36)<br>1.00<br>1.15 (0.85-1.56)<br>1.09 (0.78-1.51)<br>0.86 (0.60-1.22)<br>1.30 (0.88-1.91)<br>1.00<br>0.84 (0.48-1.47)<br>0.82 (0.45-1.50)<br>0.61 (0.31-1.20)<br>1.10 (0.54-2.23)<br>1.00<br>1.22 (0.82-1.80)<br>1.16 (0.76-1.76)<br>0.87 (0.55-1.38) | Age, energy intake, BMI, waist-to-hip ratio, smoking, education, physical activity, HRT, multivitamin supplement use, intake of alcohol, alcohol$^2$, coffee, red meat, fish and seafood, total fruit and vegetables, mutual adjustment between whole grains and refined grains |

| | | | | | | | | | |
|---|---|---|---|---|---|---|---|---|---|
| | | | | | | | ≥22.5 | 1.19 (0.72-1.97) | |
| Mizrahi A et al, 2009, Finland | Finnish Mobile Clinic Health Examination Survey | 1968-1972 - 1994, 24 years follow-up | 3932 men and women, age 40-74 years: 625 stroke cases | Incidence | Dietary history interview | Cereals, cerebrovascular disease | 10-223/20-156 g/d<br>224-295/157-210<br>296-390/211-285<br>391-1535/286-1092 | 1.00<br>1.09 (0.87-1.36)<br>1.09 (0.86-1.39)<br>1.09 (0.82-1.45) | Age, sex, BMI, smoking, physical activity, serum cholesterol, blood pressure, energy |
| | | | | | | Whole grains | 0-139/0-89 g/d<br>140-201/90-134<br>202-279/135-194<br>280-1321/195-963 | 1.00<br>0.98 (0.78-1.23)<br>1.18 (0.93-1.48)<br>1.12 (0.87-1.45) | |
| | | | | | | Refined grains | 0-50/0-43 g/d<br>51-82/44-68<br>83-124/69-99<br>125-567/100-457 | 1.00<br>0.93 (0.74-1.15)<br>0.88 (0.70-1.10)<br>0.88 (0.69-1.14) | |
| | | | | | | Cereals, ischemic stroke | 10-223/20-156 g/d<br>224-295/157-210<br>296-390/211-285<br>391-1535/286-1092 | 1.00<br>0.96 (0.71-1.31)<br>1.12 (0.81-1.53)<br>0.97 (0.66-1.43) | |
| | | | | | | Whole grains | 0-139/0-89 g/d<br>140-201/90-134<br>202-279/135-194<br>280-1321/195-963 | 1.00<br>0.95 (0.70-1.29)<br>1.11 (0.81-1.51)<br>1.06 (0.75-1.50) | |
| | | | | | | Refined grains | 0-50/0-43 g/d<br>51-82/44-68<br>83-124/69-99<br>125-567/100-457 | 1.00<br>0.81 (0.60-1.10)<br>0.95 (0.71-1.28)<br>0.85 (0.61-1.19) | |
| | | | | | | Cereals, intracerebral stroke | 10-223/20-156 g/d<br>224-295/157-210<br>296-390/211-285<br>391-1535/286-1092 | 1.00<br>1.72 (0.88-3.36)<br>0.64 (0.27-1.53)<br>1.14 (0.47-2.78) | |
| | | | | | | Whole grains | 0-139/0-89 g/d<br>140-201/90-134<br>202-279/135-194<br>280-1321/195-963 | 1.00<br>1.01 (0.49-2.08)<br>1.31 (0.64-2.68)<br>1.19 (0.53-2.67) | |
| | | | | | | Refined grains | 0-50/0-43 g/d<br>51-82/44-68<br>83-124/69-99<br>125-567/100-457 | 1.00<br>0.70 (0.36-1.36)<br>0.66 (0.33-1.33)<br>0.66 (0.31-1.42) | |

| | | | | | | | | | |
|---|---|---|---|---|---|---|---|---|---|
| Larsson SC et al, 2009, Finland | Alpha-Tocopherol, Beta-Carotene Cancer Prevention Study | 1985-1988 - 2004, 13.6 years follow-up | 26556 male smokers, age 50-69 years:<br>2702 cerebral infarctions<br>383 intracerebral hemorrhages<br>196 subarachnoid hemorrhages | Incidence | Validated FFQ, 276 food items | Cereals, cerebral infarction | 116.4 g/d<br>165.6<br>205.2<br>249.9<br>327.4 | 1.00<br>0.98 (0.87-1.10)<br>0.97 (0.85-1.10)<br>0.93 (0.81-1.07)<br>0.87 (0.74-1.03) | Age, supplementation group, cigarettes per day, BMI, SBP, DBP, serum total cholesterol, serum HDL-cholesterol, diabetes, coronary heart disease, leisure-time physical activity, alcohol, total energy |
| | | | | | | Cereals, intracerebral hemorrhage | 116.4 g/d<br>165.6<br>205.2<br>249.9<br>327.4 | 1.00<br>0.85 (0.63-1.16)<br>0.88 (0.64-1.22)<br>0.70 (0.48-1.01)<br>0.64 (0.41-1.01) | |
| | | | | | | Cereals, subarachnoid hemorrhage | 116.4 g/d<br>165.6<br>205.2<br>249.9<br>327.4 | 1.00<br>1.12 (0.69-1.81)<br>0.84 (0.50-1.42)<br>1.24 (0.74-2.07)<br>1.00 (0.54-1.84) | |
| Oba S et al, 2010, Japan | Takayama Study | 1992 – 1999, 7 years follow-up | 12561 men and 15301 women, age ≥35 years:<br>247 stroke deaths<br>126 ischemic stroke deaths<br>94 hemorrhagic stroke deaths | Mortality | Validated FFQ, 169 food items | Rice, men, total stroke | 2.3 serv/d (67.6 g)<br>3.2<br>3.7<br>4.0 | 1.00<br>0.95 (0.59-1.52)<br>0.53 (0.26-1.04)<br>0.84 (0.43-1.62) | Age, BMI, smoking status, physical activity, hypertension, education, total energy, alcohol, dietary fiber, salt, total fat |
| | | | | | | Rice, women | 1.9 serv/d<br>2.3<br>2.7<br>3.2 | 1.00<br>1.47 (0.78-2.79)<br>1.22 (0.62-2.37)<br>1.37 (0.64-2.94) | |
| | | | | | | Rice, men, ischemic stroke | 2.3 serv/d (67.6 g)<br>3.2<br>3.7<br>4.0 | 1.00<br>0.97 (0.51-1.82)<br>0.52 (0.19-1.41)<br>1.21 (0.61-2.37) | |
| | | | | | | Rice, women | 1.9 serv/d<br>2.3<br>2.7<br>3.2 | 1.00<br>1.53 (0.64-3.68)<br>1.14 (0.49-2.67)<br>1.67 (0.69-4.07) | |
| | | | | | | Rice, men, hemorrhagic stroke | 2.3 serv/d (67.6 g)<br>3.2<br>3.7<br>4.0 | 1.00<br>0.73 (0.35-1.50)<br>0.39 (0.15-1.00)<br>0.71 (0.34-1.49) | |
| | | | | | | Rice, women | 1.9 serv/d<br>2.3<br>2.7<br>3.2 | 1.00<br>0.98 (0.33-2.91)<br>1.81 (0.71-4.66)<br>2.36 (0.92-6.03) | |

| | | | | | | | | | |
|---|---|---|---|---|---|---|---|---|---|
| Eshak ES et al, 2011, Japan | Japan Collaborative Cohort Study | 1988-1990 – 2003, 14.1 years follow-up | 83752 men and women, age 40-79 years: 1640 stroke deaths | Mortality | Validated FFQ, 40 food items | Rice, men<br><br><br><br><br>Rice, women | 280 g/d<br>420<br>449<br>583<br>711<br>279 g/d<br>359<br>420<br>453<br>560 | 1.00<br>0.96 (0.79-1.17)<br>0.96 (0.76-1.22)<br>0.78 (0.61-1.00)<br>1.02 (0.82-1.31)<br>1.00<br>0.85 (0.64-1.12)<br>0.89 (0.72-1.11)<br>0.89 (0.64-1.26)<br>0.99 (0.75-1.31) | Age, history of hypertension, history of diabetes, BMI, alcohol, smoking status, exercise, walking, education, perceived mental stress, sleep duration, fish, meat, fruit, dairy products, soy, total energy |
| Misirili G et al, 2012, Greece | European Prospective Investigation into Cancer and Nutrition - Greece | 1994-1999 – 2009, 10.6 years follow-up | 23601 men and women, age 25-67 years: 395 incidence cases<br>196 deaths cerebro-vascular disease | Incidence and mortality | FFQ, 150 food items | Cereals, incidence<br>Cereals, mortality | Per 70 g/d<br>Per 70 g/d | 1.02 (0.89-1.16)<br>0.97 (0.79-1.19) | Age, education, smoking status, BMI, physical activity, hypertension, diabetes, total energy intake |
| Eshak E et al, 2014, Japan | Japan Public Health Center-based Prospective Study | 1990/1993 – 2007/2009, 15.2 years follow-up | 91223 men and women, age 40-69 years: 4395 total strokes<br>1777 hemorrhagic strokes<br>2590 ischemic strokes<br>1153 stroke deaths | Incidence | Validated FFQ, 44/52 food items | Rice, stroke incidence<br><br><br><br><br><br>Rice, hemorrhagic stroke incidence<br><br><br><br>Rice, ischemic stroke incidence<br><br><br><br>Rice, stroke mortality | 251 g/d<br>326<br>377<br>430<br>542<br>251 g/d<br>326<br>377<br>430<br>542<br>251 g/d<br>326<br>377<br>430<br>542<br>251 g/d<br>326<br>377<br>430<br>542 | 1.00<br>1.07 (0.93-1.17)<br>0.94 (0.85-1.08)<br>0.93 (0.84-1.13)<br>1.01 (0.90-1.14)<br>1.00<br>1.05 (0.90-1.22)<br>0.95 (0.81-1.12)<br>0.95 (0.81-1.11)<br>0.96 (0.79-1.15)<br>1.00<br>1.07 (0.92-1.23)<br>0.99 (0.81-<br>1.07)?<br>0.99 (0.81-1.16)<br>1.05 (0.90-1.22)<br>1.00<br>1.07 (0.88-1.30)<br>1.07 (0.88-1.32)<br>1.00 (0.82-1.23)<br>1.03 (0.82-1.30) | Age, sex, public health center area, hypertension, diabetes, use of lipid-lowering drugs, BMI, smoking status, ethanol intake, leisure-time sports activity, occupation, seafood, meat, fruit, vegetables, soy, SFA, sodium, total energy, menopausal status, HRT |

| | | | | | | | | | |
|---|---|---|---|---|---|---|---|---|---|
| Muraki I et al, 2014, USA | Nurses' Health Study<br><br><br><br>Nurses' Health Study 2<br><br><br><br>Health Professionals Follow-up Study | 1984-2010, 26 years follow-up<br><br><br>1991-2011, 20 years follow-up<br><br><br>1986-2010, 24 years follow-up | 73228 women, age 38-63 years: 2703 stroke cases<br><br>92158 women, age 27-44 years: 494 stroke cases<br><br>42170 men, age 40-75 years: 1475 stroke cases | Incidence | Validated FFQ, 118-166 food items | White rice<br><br><br><br><br>Brown rice<br><br><br><br><br>Total rice | <1 serv/wk<br>1<br>2-4<br>≥5<br>Per 3 serv/wk<br><1 serv/wk<br>1<br>2-4<br>≥5<br>Per 3 serv/wk<br><1 serv/wk<br>1<br>2-4<br>≥5<br>Per 3 serv/wk | 1.00<br>0.97 (0.90-1.03)<br>0.97 (0.89-1.05)<br>1.25 (0.99-1.57)<br>1.02 (0.94-1.11)<br>1.00<br>1.03 (0.94-1.14)<br>1.05 (0.92-1.19)<br>1.39 (0.99-1.96)<br>1.11 (0.98-1.26)<br>1.00<br>0.94 (0.87-1.01)<br>0.98 (0.91-1.06)<br>1.04 (0.89-1.21)<br>1.02 (0.95-1.10) | Age, ethnicity, BMI, smoking status, alcohol, physical activity, family history of MI, menopausal status, OC use (NHS2), HRT, multivitamin use, current aspirin use, prevalent hypertension, prevalent hypercholesterolemia, prevalent diabetes, total energy intake, alternate Healthy Eating Index score |
| Tognon G et al, 2014, Denmark | The 1982-83 Danish Monitoring trends and determinants of Cardiovascular disease study (MONICA) | 1982-1983 – 2007, 14 years follow-up | 948 women and 901 men, age NA: 167 stroke cases<br>40 stroke deaths | Incidence and mortality | 7 day food record | Cereals, incidence<br><br>Cereals, mortality | >median vs. <median<br>>median vs. <median | 0.82 (0.60-1.11)<br><br>1.00 (0.53-1.89) | Age, sex, BMI, education, physical activity, cigarette smoking |
| Johnsen NF et al, 2015, Norway, Sweden, Denmark | HELGA Cohort (Norwegian Women and Cancer Study, Northern Sweden Health and Disease Study, Danish Diet, Cancer and Health Study – part of the EPIC study) | 1992-1998 – 2008-2009, 11.1 (Norway), 14.2 (Sweden), 11.9 years (Denmark) follow-up | 120010 men and women, age 30-64 years: 137/143 stroke deaths | Mortality | Validated FFQ, 88 food items (Norway), 98 food items (Sweden) 173 food items (Denmark) | Whole grain breakfast cereals, women<br><br>Non-white bread<br><br><br><br>Crisp bread<br><br><br><br>Total whole grain products | 0 g/d<br>0.8<br>12<br>50<br>25 g/d<br>80<br>113<br>180<br>0.6 g/d<br>2<br>6<br>31<br>56 g/d<br>100<br>131<br>201 | 1.00<br>0.70 (0.33-1.47)<br>0.73 (0.43-1.23)<br>0.66 (0.43-1.02)<br>1.00<br>0.86 (0.48-1.54)<br>1.02 (0.55-1.87)<br>0.75 (0.41-1.37)<br>1.00<br>1.03 (0.62-1.74)<br>0.91 (0.54-1.56)<br>1.22 (0.68-2.18)<br>1.00<br>0.78 (0.50-1.22)<br>0.57 (0.35-0.94)<br>0.85 (0.53-1.37) | Age, follow-up time, education, smoking status/years since quit/ cigarettes per day, alcohol, BMI, total energy |

| | | | | | | | | | |
|---|---|---|---|---|---|---|---|---|---|
| | | | | | | Oat | 0 g/d<br>0.4<br>4<br>19 | 1.00<br>1.03 (0.64-1.67)<br>0.66 (0.38-1.16)<br>0.63 (0.34-1.15) | |
| | | | | | | Rye | 8 g/d<br>18<br>22<br>41 | 1.00<br>0.71 (0.41-1.22)<br>0.81 (0.47-1.37)<br>0.91 (0.53-1.57) | |
| | | | | | | Wheat | 0.4 g/d<br>3<br>10<br>37 | 1.00<br>0.72 (0.44-1.18)<br>0.83 (0.50-1.38)<br>0.55 (0.23-1.30) | |
| | | | | | | Total whole grain types | 20 g/d<br>33<br>49<br>74 | 1.00<br>0.55 (0.34-0.88)<br>0.50 (0.31-0.82)<br>0.80 (0.48-1.33) | |
| | | | | | | Whole grain breakfast cereals, men | 0 g/d<br>0.8<br>7<br>50 | 1.00<br>0.92 (0.59-1.45)<br>0.86 (0.56-1.32)<br>0.87 (0.47-1.28) | |
| | | | | | | Non-white bread | 13 g/d<br>66<br>118<br>201 | 1.00<br>0.74 (0.44-1.22)<br>0.56 (0.35-0.91)<br>0.86 (0.50-1.46) | |
| | | | | | | Crisp bread | 1 g/d<br>2<br>4<br>34 | 1.00<br>1.10 (0.67-1.79)<br>0.97 (0.60-1.57)<br>1.30 (0.71-2.39) | |
| | | | | | | Total whole grain products | 64 g/d<br>107<br>156<br>222 | 1.00<br>1.01 (0.66-1.56)<br>0.55 (0.35-0.87)<br>0.86 (0.54-1.37) | |
| | | | | | | Oat | 0 g/d<br>0.4<br>3<br>30 | 1.00<br>0.67 (0.42-1.07)<br>1.03 (0.60-1.76)<br>0.71 (0.41-1.21) | |
| | | | | | | Rye | 7 g/d<br>21<br>38 | 1.00<br>0.77 (0.48-1.23)<br>0.72 (0.39-1.31) | |

| | | | | | | | | | |
|---|---|---|---|---|---|---|---|---|---|
| | | | | | | Wheat<br><br><br><br><br>Total whole grain types | 56<br>0.1 g/d<br>1<br>5<br>10<br>21 g/d<br>37<br>54<br>80 | 0.61 (0.36-1.05)<br>1.00<br>0.59 (0.35-0.99)<br>0.72 (0.41-1.27)<br>0.74 (0.41-1.32)<br>1.00<br>0.70 (0.44-1.10)<br>0.69 (0.43-1.11)<br>0.71 (0.44-1.15) | |
| Wang JB et al, 2016, China | Linxian Nutrition Intervention Trial cohort | 1984-1991 - 2010, 19-26 years follow-up | 2445 men and women, age 40-69 years: 452 stroke deaths | Mortality | FFQ, 64 food items | All grains<br>Non-whole grains<br>Whole grains | Per 1 time/day<br>Per 1 time/day<br>Per 1 time/day | 0.90 (0.83-0.97)<br>0.90 (0.82-0.98)<br>0.93 (0.83-1.04) | Age, sex, commune, smoking, drinking, season, BMI |

BMI; body mass index, DBP; diastolic blood pressure, FFQ; food frequency questionnaire, HDL-cholesterol; high-density lipoprotein cholesterol, HRT; hormone replacement therapy, MI; myocardial infarction, SBP; systolic blood pressure, SFA; saturated fatty acids, trans-FA: trans fatty acids

## Table S5. Whole grains and refined grains and cardiovascular disease

| Author, publication year, country | Study name | Study period | Number of participants, gender, age, number of cases/deaths | CVD incidence or mortality | Dietary assessment | Exposure and subgroup | Whole grain consumption frequency or amount | Relative risks (95% confidence intervals) | Adjustment for confounding factors |
|---|---|---|---|---|---|---|---|---|---|
| Jacobs DR et al, 2001, Norway | Norwegian County Study | 1977-1983 – 1994, 14.4 years follow-up | 33848 men and women, age 35-56 years: 758 CVD deaths | Mortality | FFQ, 66 food items | Whole grain bread score | 0.05-0.60<br>0.83-0.83<br>0.90-1.13<br>1.35-1.80<br>2.25-5.40 | 1.00<br>0.93 (0.73-1.18)<br>0.84 (0.68-1.05)<br>0.84 (0.66-1.05)<br>0.77 (0.60-0.98) | Age, energy intake, sex, smoking status, physical activity during leisure, physical activity during work, cod liver oil, multivitamin use, SFA, SBP, serum total cholesterol, BMI |
| Appleby PN et al, 2002, UK | The Health Food Shoppers Study | 1973-1979 – 1997, 19.8 years follow-up | 10741 men and women, age 16-89 years: 1202 circulatory disease deaths | Mortality | FFQ | Wholemeal bread<br>Bran cereals | Daily vs less<br>Daily vs less | 0.86 (0.76-0.98)<br>1.04 (0.92-1.18) | Age at recruitment, sex, smoking, fresh fruit, nuts/dried fruit, raw vegetables salads, mutual adjustment between wholemeal bread and bran cereals |
| Liu S et al, 2003, USA | Physicians' Health Study | 1982-1988, 5.5 years follow-up | 86190 men, mean age 40-84 years: 1381 CVD deaths | Mortality | Validated FFQ | Whole grain breakfast cereals<br><br><br><br>Refined-grain breakfast cereals<br><br><br><br>Total breakfast cereals | Rarely<br>1 serv/wk<br>2-6/wk<br>≥1/day<br>Rarely<br>1 serv/wk<br>2-6/wk<br>≥1/day<br>Rarely<br>1 serv/wk<br>2-6/wk<br>≥1/day | 1.00<br>0.93 (0.75-1.17)<br>0.82 (0.68-0.98)<br>0.80 (0.66-0.97)<br>1.00<br>1.18 (0.99-1.40)<br>1.08 (0.89-1.31)<br>1.04 (0.84-1.27)<br>1.00<br>1.04 (0.89-1.22)<br>0.93 (0.79-1.10)<br>0.87 (0.74-1.03) | Age, cigarette smoking, alcohol intake, physical activity, BMI, type 2 diabetes, high cholesterol, hypertension, use of multivitamins |
| Sahyoun NR et al, 2006, USA | NA | 1981-1984 – 1995, 12-15 years follow-up | 535 men and women, age ≥60 years: 89 CVD deaths | Mortality | 3-day food record | Whole grain | 0.31 serv/d<br>0.86<br>1.49<br>2.90 | 1.00<br>0.77 (0.41-1.43)<br>0.76 (0.41-1.41)<br>0.48 (0.25-0.96) | Age, sex, race, education, marital status, smoking, alcohol intake, exercise, BMI, energy intake, SFA, antihypertensive or lipid-lowering therapy |
| Mink PJ et al, 2007, USA | Iowa Women's Health Study | 1986-2002, 16 years follow-up | 34489 women, age 55-69 years: 2316 CVD | Mortality | Validated FFQ, 127 food items | Bran, added to food | 0 serv/wk<br>・0 | 1.00<br>0.86 (0.76-0.97) | Age, energy intake, marital status, education, blood pressure, diabetes, BMI, waist- |

| | | | | | | | | | |
|---|---|---|---|---|---|---|---|---|---|
| | | | deaths | | | | | | to-hip ratio, physical activity, smoking, HRT |
| Jacobs DR, 2007, USA | Iowa Women's Health Study | 1986-2003, 17 years follow-up | 27312 women, age 55-69 years: 1900 CVD deaths | Mortality | Validated FFQ, 127 food items | Whole grains<br><br><br><br><br>Refined grains | 1.8 serv/wk<br>5.6<br>8.8<br>14.5<br>25.6<br>0-5.75 serv/wk<br>6-9.5<br>9.6-13.5<br>14-22<br>≥22.5 | 1.00<br>0.96 (0.84-1.10)<br>0.83 (0.72-0.96)<br>0.83 (0.71-0.96)<br>0.73 (0.62-0.86)<br>1.00<br>0.94 (0.82-1.08)<br>0.89 (0.76-1.03)<br>0.75 (0.64-0.88)<br>0.94 (0.78-1.12) | Age, energy intake, BMI, waist-to-hip ratio, smoking, education, physical activity, HRT, multivitamin supplement use, intake of alcohol, coffee, red meat, fish and seafood, total fruit and vegetables, mutual adjustment between whole grains and refined grains |
| Gardener H et al, 2011, USA | The Northern Manhattan Study | NA – NA, 9 years follow-up | 2568 men and women, age >40 years: 314 vascular deaths | Mortality | FFQ | Cereals | ≥61 vs. <61 g/d | 0.98 (0.79-1.23) | Age, sex, race-ethnicity, education, moderate to heavy physical activity, energy, cigarette smoking |
| Eshak ES et al, 2011, Japan | Japan Collaborative Cohort Study | 1988-1990 – 2003, 14.1 years follow-up | 83752 men and women, age 40-79 years: 3514 CVD deaths | Mortality | Validated FFQ, 40 food items | Rice, men<br><br><br><br><br>Rice, women | 280 g/d<br>420<br>449<br>583<br>711<br>279 g/d<br>359<br>420<br>453<br>560 | 1.00<br>0.90 (0.79-1.03)<br>0.87 (0.74-1.02)<br>0.79 (0.67-0.93)<br>0.82 (0.70-0.97)<br>1.00<br>0.94 (0.78-1.14)<br>0.90 (0.77-1.05)<br>1.20 (0.94-1.51)<br>1.07 (0.88-1.34) | Age, history of hypertension, history of diabetes, BMI, alcohol, smoking status, exercise, walking, education, perceived mental stress, sleep duration, fish, meat, fruit, dairy products, soy, total energy |
| Fitzgerald KC et al, 2012, USA | Women's Health Study | 1992-1994 – 2004, 14.6 years follow-up | 34827 women, age ≥45 years: 1094 CVD cases | Incidence | Validated FFQ, 133 food items | Whole grains | <0.50 serv/d<br>0.50-0.93<br>0.94-1.36<br>1.40-2.20<br>≥2.21 | 1.00<br>1.01 (0.84-1.21)<br>1.03 (0.86-1.24)<br>0.86 (0.71-1.05)<br>0.96 (0.79-1.17) | Age, randomization status, smoking, postmenopausal status, HRT, alcohol intake, energy, physical activity, cigarettes per day, highest education level |
| Von Ruesten A et al, 2013, Germany | European Prospective Investigation into Cancer and Nutrition–Potsdam study | 1994/1998–NA, 8 years follow-up | 23531 men and women, age 35–65 years: 363 CVD cases | Incidence | Validated FFQ, 148 food items | Whole grain bread<br>Other bread<br>Grain flakes, muesli<br>Cornflakes, crisps<br>Pasta, rice | Per 50 g/d<br>Per 50 g/d<br>Per 50 g/d<br>Per 50 g/d<br>Per 100 g/d | 0.96 (0.81-1.14)<br>0.99 (0.85-1.16)<br>0.54 (0.28-1.01)<br>1.89 (0.79-4.51)<br>1.01 (0.44-2.34) | Age, sex, smoking status, pack-years of smoking, alcohol, leisure-time physical activity, BMI, waist-to-hip ratio, prevalent hypertension, high blood lipid levels, education, vitamin supplementation, total |

| | | | | | | | | | |
|---|---|---|---|---|---|---|---|---|---|
| | | | | | | | | | energy, non-consumption of the food group, other food groups |
| Eshak E et al, 2014, Japan | Japan Public Health Center-based Prospective Study | 1990/1993 – 2007/2009, 15.2 years follow-up | 91223 men and women, age 40-69 years: 2705 CVD deaths | Mortality | Validated FFQ, 44/52 food items | Rice | 251 g/d<br>326<br>377<br>430<br>542 | 1.00<br>0.96 (0.85-1.09)<br>1.00 (0.88-1.15)<br>0.81 (0.80-1.11)<br>0.97 (0.84-1.13) | Age, sex, public health center area, hypertension, diabetes, use of lipid-lowering drugs, BMI, smoking status, ethanol intake, leisure-time sports activity, occupation, seafood, meat, fruit, vegetables, soy, SFAs, sodium, total energy, and for women: menopausal status, HRT |
| Buil-Cosiales P et al, 2014, Spain | Prevencion con DietaMediterranea (PREDIMED) study | 2003-2009 – 2012, 5.9 years follow-up | 7216 men and women, age 55-75 years: 103 CVD deaths | Mortality | Validated FFQ, 137 food items | Whole grains | 0 g/d<br>1<br>7<br>33<br>89 | 1.00<br>0.79 (0.39-1.59)<br>0.76 (0.33-1.76)<br>0.81 (0.35-1.90)<br>0.73 (0.34-1.58) | Age, sex, smoking status, diabetes, BMI, SBP, DBP, recruitment center, statins, alcohol, education, physical activity, total energy, vegetables, fruits |
| Atkins JL et al, 2014, United Kingdom | British Regional Heart Study | 1998-2000 – 2010, 11.3 years follow-up | 3328 men, age 60-79 years:<br>582 CVD cases<br>327 CVD deaths | Incidence and mortality | Validated FFQ, 86 food items | Cereals, incidence<br>Bread<br><br>Cereals, mortality<br>Bread | Daily vs. <1 day/wk<br>Whole grain vs. none<br>Daily vs. <1 day/wk<br>Whole grain vs. none | 1.13 (0.79-1.62)<br>0.60 (0.22-1.65)<br><br>1.37 (0.83-2.25)<br>0.57 (0.14-2.40) | Age, smoking, alcohol, physical activity, social class, BMI, energy intake, diet score without respective components |
| Muraki I et al, 2014, USA | Nurses' Health Study<br><br><br><br><br>Nurses' Health Study 2<br><br><br><br><br>Health Professionals Follow-up Study | 1984-2010, 26 years follow-up<br><br><br><br><br>1991-2011, 20 years follow-up<br><br><br><br><br>1986-2010, 24 years follow-up | 73228 women, age 38-63 years: 5763 CVD cases<br><br>92158 women, age 27-44 years: 1028 CVD cases<br><br>42170 men, age 40-75 years: 5600 CVD cases | Incidence | Validated FFQ, 118-166 food items | White rice<br><br><br><br><br>Brown rice<br><br><br><br><br>Total rice | <1 serv/wk<br>1<br>2-4<br>≥5<br>Per 3 serv/wk<br><1 serv/wk<br>1<br>2-4<br>≥5<br>Per 3 serv/wk<br><1 serv/wk<br>1<br>2-4<br>≥5<br>Per 3 serv/wk | 1.00<br>1.01 (0.97-1.05)<br>1.02 (0.97-1.07)<br>0.98 (0.84-1.14)<br>1.01 (0.96-1.06)<br>1.00<br>1.01 (0.95-1.07)<br>1.05 (0.97-1.13)<br>1.01 (0.79-1.28)<br>1.04 (0.96-1.13)<br>1.00<br>1.00 (0.95-1.04)<br>1.01 (0.96-1.06)<br>0.99 (0.90-1.08)<br>1.00 (0.96-1.05) | Age, ethnicity, BMI, smoking status, alcohol, physical activity, family history of MI, menopausal status, oral contraceptive use (NHS2), HRT, multivitamin use, current aspirin use, prevalent hypertension, prevalent hypercholesterolemia, prevalent diabetes, total energy intake, alternate Healthy Eating Index score |

| | | | | | | | | | |
|---|---|---|---|---|---|---|---|---|---|
| Tognon G et al, 2014, Denmark | The 1982-83 Danish Monitoring trends and determinants of Cardiovascular disease study (MONICA) | 1982-1983 – 2007, 14 years follow-up | 948 women and 901 men, age NA: 755 CVD cases<br>223 CVD deaths | Incidence and mortality | Validated 7 day food record | Cereals, incidence<br><br>Cereals, mortality | >median vs. <median<br>>median vs. <median | 0.90 (0.78-1.04)<br><br>0.76 (0.58-1.00) | Age, sex, BMI, education, physical activity, cigarette smoking |
| Wu H et al, 2015, USA | Nurses' Health Study | 1984-2010, 26 years follow-up | 74341 women, age 38-63 years: 2989 CVD deaths | Mortality | Validated FFQ, 126 food items | Whole grains<br><br><br><br><br>Total bran<br><br><br><br><br>Total germ | 4.2 g/d<br>9.7<br>14.7<br>21.1<br>33.0<br>0.7 g/d<br>2.0<br>3.5<br>5.7<br>10.4<br>0.2 g/d<br>0.4<br>0.6<br>0.9<br>1.6 | 1.00<br>0.97 (0.87-1.08)<br>0.96 (0.86-1.08)<br>0.82 (0.73-0.92)<br>0.86 (0.76-0.96)<br>1.00<br>0.89 (0.79-0.99)<br>0.94 (0.83-1.07)<br>0.80 (0.70-0.92)<br>0.80 (0.70-0.91)<br>1.00<br>1.07 (0.95-1.20)<br>1.01 (0.89-1.15)<br>1.04 (0.91-1.19)<br>1.11 (0.97-1.27) | Age, ethnicity, BMI, smoking status, cigarettes per day, pack-years smoked, years since quitting smoking, alcohol, physical activity, family history of diabetes, cancer and heart disease, multivitamin use, aspirin use, hypertension, high cholesterol, diabetes, total energy, healthy eating index (excluding whole grains), postmenopausal status, HRT |
| Wu H et al, 2015, USA | Health Professionals Follow-up Study | 1986-2010, 24 years follow-up | 43744 men, age 32-87 years: 3621 CVD deaths | Mortality | Validated FFQ, 131 food items | Whole grains<br><br><br><br><br>Total bran<br><br><br><br><br>Total germ | 5.9 g/d<br>14.4<br>22.1<br>31.3<br>47.8<br>0.7 g/d<br>2.6<br>5.0<br>8.2<br>15.0<br>0.2 g/d<br>0.6<br>0.9<br>1.3<br>2.3 | 1.00<br>0.92 (0.84-1.02)<br>0.92 (0.83-1.02)<br>0.91 (0.82-1.01)<br>0.84 (0.75-0.93)<br>1.00<br>0.93 (0.84-1.04)<br>0.93 (0.83-1.05)<br>0.86 (0.77-0.97)<br>0.80 (0.71-0.90)<br>1.00<br>1.03 (0.93-1.14)<br>0.94 (0.84-1.05)<br>1.03 (0.92-1.16)<br>1.03 (0.92-1.16) | Age, ethnicity, BMI, smoking status, cigarettes per day, pack-years smoked, years since quitting smoking, alcohol, physical activity, family history of diabetes, cancer and heart disease, multivitamin use, aspirin use, hypertension, high cholesterol, diabetes, total energy, healthy eating index (excluding whole grains) |

| | | | | | | | | | |
|---|---|---|---|---|---|---|---|---|---|
| Wu H et al, 2015, USA | Nurses' Health Study & Health Professionals Follow-up Study | 1984-2010, 26 years follow-up 1986-2010, 24 years follow-up | 74341 women, age 38-63 years: 2989 CVD deaths 43744 men, age 32-87 years: 3621 CVD deaths | Mortality | Validated FFQ, 126/131 food items | Naturally occurring bran<br><br><br><br>Added bran<br><br><br><br><br>Refined grains | 0-0.4/0-0.4 g/d<br>0.5-1.4/0.5-1.9<br>1.5-2.99/2.0-3.9<br>≥3.0/≥4.0<br>0-0.4/0-0.4 g/d<br>0.5-1.4/0.5-1.9<br>1.5-2.9/2.0-3.9<br>3.0-5.9/4.0-9.9<br>≥6.0/≥10.0<br>Per 28 g/d | 1.00<br>0.98 (0.90-1.07)<br>0.92 (0.84-1.01)<br>0.89 (0.81-0.98)<br>1.00<br>0.85 (0.78-0.91)<br>0.83 (0.77-0.90)<br>0.82 (0.76-0.88)<br>0.76 (0.70-0.83)<br>0.99 (0.97-1.01) | Age, ethnicity, BMI, smoking status, cigarettes per day, pack-years smoked, years since quitting smoking, alcohol, physical activity, family history of diabetes, cancer and heart disease, multivitamin use, aspirin use, hypertension, high cholesterol, diabetes, total energy, healthy eating index (excluding whole grains), women: postmenopausal status, HRT |
| Huang T et al, 2015, USA | NIH-AARP Diet and Health Study | 1995-1996 – 2008, 14 years follow-up | 367442 men and women, age 50-71 years: 11283 CVD deaths | Mortality | Validated FFQ, 124 food items | Whole grains | 0.13 oz/1000 kcal/d[1]<br>0.30<br>0.47<br>0.69<br>1.20 | 1.00<br>0.93 (0.88-0.98)<br>0.88 (0.83-0.93)<br>0.81 (0.77-0.86)<br>0.83 (0.78-0.88) | Age, sex, number of cigarettes per day, time of smoking cessation, race/ ethnicity, alcohol, education, marital status, health status, BMI, physical activity, red meat, total fruit and vegetables, total energy, HRT (women) |
| Sonestedt E et al, 2015, Sweden | Malmö Diet and Cancer Study | 1991-1996 – 2009, 14 years follow-up | 26445 men and women, age 44-74 years: 2921 ischemic CVD events | Incidence | Validated FFQ, 168 food items, diet history interview | Whole grains<br><br><br><br><br>Refined grains | 0.0 portions/d<br>0.3<br>0.7<br>1.2<br>2.5<br>1.2 portions/d<br>2.0<br>2.5<br>3.1<br>4.3 | 1.00<br>0.89 (0.80-1.00)<br>0.92 (0.82-1.02)<br>0.80 (0.72-0.90)<br>0.87 (0.77-0.97)<br>1.00<br>1.03 (0.92-1.16)<br>1.05 (0.94-1.18)<br>1.05 (0.94-1.18)<br>1.06 (0.95-1.20) | Age, sex, season, diet method version, energy intake, BMI, smoking, alcohol, leisure-time physical activity, education |
| Xu M et al, 2015, USA | NIH-AARP Diet and Health Study | 1995-1996 – 2008, 14 years follow-up | 367442 men and women, age 50-71 years: 11283 CVD deaths | Mortality | Validated FFQ, 124 food items | Ready-to-eat cereals | 0.00 g/d<br>0.67<br>3.48<br>9.33<br>22.48 | 1.00<br>0.90 (0.85-0.95)<br>0.90 (0.85-0.95)<br>0.86 (0.81-0.91)<br>0.76 (0.71-0.81) | Age, sex, smoking status, smoking dose, time since quitting smoking, race/ethnicity, education, marital status, self-rated health status, BMI, physical activity, menopausal hormone therapy, alcohol, red meat, fruits, vegetables, total |

| | | | | | | | | | |
|---|---|---|---|---|---|---|---|---|---|
| | | | | | | | | | energy |
| Wang JB et al, 2016, China[2] | Linxian Nutrition Intervention Trial cohort | 1984-1991 - 2010, 19-26 years follow-up | 2445 men and women, age 40-69 years: 807 CVD deaths | Mortality | FFQ, 64 food items | All grains<br>Non-whole grains<br>Whole grains | Per 1 time/day<br>Per 1 time/day<br>Per 1 time/day | 0.94 (0.89-1.00)<br>0.96 (0.90-1.03)<br>0.93 (0.86-1.02) | Age, sex, commune, smoking, drinking, season, BMI |

BMI; body mass index, CVD; cardiovascular disease, DBP; diastolic blood pressure, FFQ; food frequency questionnaire, HRT; hormone replacement therapy, SBP; systolic blood pressure, SFA; saturated fatty acids

[1] The original paper reports in ounces/d, after contact with the authors it was confirmed that ounces/1000 kcal/d is correct.

[2] Data for heart disease and stroke deaths were pooled using a fixed effects model.

## Table S6. Whole grains and refined grains and total cancer

| Author, publication year, country | Study name | Study period | Number of participants, gender, age, number of cases/deaths | Total cancer incidence or mortality | Dietary assessment | Exposure and subgroup | Whole grain consumption frequency or amount | Relative risks (95% confidence intervals) | Adjustment for confounding factors |
|---|---|---|---|---|---|---|---|---|---|
| Jacobs DR et al, 2001, Norway | Norwegian County Study | 1977-1983 – 1994, 14.4 years follow-up | 33848 men and women, age 35-56 years: 870 cancer deaths | Mortality | FFQ, 66 food items | Whole grain bread score | 0.05-0.60<br>0.83-0.83<br>0.90-1.13<br>1.35-1.80<br>2.25-5.40 | 1.00<br>0.96 (0.76-1.20)<br>0.89 (0.73-1.08)<br>0.90 (0.73-1.11)<br>0.79 (0.62-1.02) | Age, energy, sex, smoking status, leisure-time physical activity, occupational physical activity, cod liver oil use, multivitamins, SFA, SBP, serum total cholesterol, BMI |
| Appleby PN et al, 2002, UK | The Health Food Shoppers Study | 1973-1979 – 1997, 19.8 years follow-up | 11000 men and women, age 16-89 years: 680 cancer deaths | Mortality | FFQ | Wholemeal bread<br>Bran cereals | Daily vs less<br>Daily vs less | 1.01 (0.85-1.20)<br>0.93 (0.78-1.11) | Age at recruitment, sex, smoking, fresh fruit, nuts/dried fruit, raw vegetables salads, mutual adjustment wholemeal bread bran cereals |
| Khan MMH et al, 2004, Japan | The Hokkaido Study | 1984-1985 – 2002, 14.3 years follow-up | 1524 men and 1634 women, age ≥40 years: 155/89 cancer deaths | Mortality | FFQ, 37 food items | Bread, men<br>Instant noodles<br>Noodles<br>Bread, women<br>Instant noodles<br>Noodles | ・2/wk vs ・1mo<br>・2/wk vs ・1mo<br>・2/wk vs ・1mo<br>・2/wk vs ・1mo<br>・2/wk vs ・1mo<br>・2/wk vs ・1mo | 0.8 (0.5-1.2)<br>0.9 (0.6-1.5)<br>1.0 (0.7-1.3)<br>0.9 (0.5-1.5)<br>0.6 (0.2-1.4)<br>1.2 (0.8-1.8) | Age, smoking<br><br><br>Age, health status, health education, health screening, smoking |
| Jacobs DR, 2007, USA | Iowa Women's Health Study | 1986-2003, 17 years follow-up | 27312 women, age 55-69 years: 2099 cancer deaths | Mortality | Validated FFQ, 127 food items | Whole grains<br><br><br><br><br>Refined grains | 1.8 serv/wk<br>5.6<br>8.8<br>14.5<br>25.6<br>0-5.75 serv/wk<br>6-9.5<br>9.6-13.5<br>14-22<br>≥22.5 | 1.00<br>0.86 (0.75-0.99)<br>0.95 (0.83-1.09)<br>0.83 (0.72-0.96)<br>0.89 (0.77-1.04)<br>1.00<br>1.00 (0.88-1.15)<br>0.96 (0.83-1.11)<br>0.98 (0.85-1.14)<br>0.98 (0.82-1.16) | Age, energy intake, BMI, waist-to-hip ratio, smoking, education, physical activity, HRT, multivitamin supplement use, intake of alcohol, coffee, red meat, fish and seafood, total fruit and vegetables, mutual adjustment between whole grains and refined grains |

| | | | | | | | | | |
|---|---|---|---|---|---|---|---|---|---|
| Iso H et al, 2007, Japan | Japan Collaborative Cohort Study | 1988-1990-2003, ~12.8 years follow-up | 42513 men and 57777 women, age 40-79 years: 3579/ 2138 cancer deaths | Mortality | FFQ | Rice, men<br><br><br>Rice, women | <3/d<br>3-4<br>≥5<br><3/d<br>3-4<br>≥5 | 1.00<br>0.99 (0.91-1.07)<br>0.87 (0.80-0.96)<br>1.00<br>0.95 (0.86-1.05)<br>1.04 (0.91-1.18) | Age, area of study |
| Couto E et al, 2011, Europe | European Prospective Investigation into Cancer and Nutrition | 1992-2000 – 2002-2005, 8.7 years follow-up | 142605 men and 335873 women, age 25-70 years: 9669/21062 cancer cases | Incidence | Validated FFQs, 7-day or 14-day record diaries, diet history | Cereals | Per 110 g/d | 0.97 (0.95-0.98) | Age, sex, centre, duration of smoking, smoking status, education, height, BMI, total energy intake, physical activity, women: age at menarche, parity, menopausal status, oral contraceptive use, HRT |
| Von Ruesten A et al, 2013, Germany | European Prospective Investigation into Cancer and Nutrition–Potsdam study | 1994/1998–NA, 8 years follow-up | 23,531 m & w, age 35–65 years: 844 cancer cases | Incidence | Validated FFQ, 148 food items | Whole grain bread<br>Other bread<br>Grain flakes, muesli<br>Cornflakes, crisps<br>Pasta, rice | Per 50 g/d<br>Per 50 g/d<br>Per 50 g/d<br>Per 50 g/d<br>Per 100 g/d | 0.94 (0.84-1.05)<br>0.98 (0.88-1.09)<br>0.98 (0.73-1.30)<br>1.10 (0.57-2.11)<br>0.84 (0.48-1.48) | Age, sex, smoking status, pack-years of smoking, alcohol, leisure-time physical activity, BMI, waist-to-hip ratio, prevalent hypertension, high blood lipid levels, education, vitamin supplementation, total energy, non-consumption of the food group, other food groups |

| | | | | | | | | | |
|---|---|---|---|---|---|---|---|---|---|
| Sharma S et al, 2013, USA | Multiethnic Cohort Study | 1993-1996 – 2001, NA | 146389 men and women, age 45-75 years: 2028/1464 cancer deaths | Mortality | Validated FFQ, 180 food items | Grains, men, all | ≤5.6 serv/(d<br>5.7-7.8<br>7.9-10.8<br>>10.8 | NA (significant heterogeneity by ethnicity) | Age, time on study, years of education, energy intake, smoking status, pack-years, BMI, physical activity, diabetes, alcohol, women: HRT, oophorectomy |
| | | | | | | Grains, African American | ≤5.6 serv/(d<br>5.7-7.8<br>7.9-10.8<br>>10.8 | 1.00<br>0.88 (0.66-1.17)<br>1.18 (0.85-1.65)<br>1.24 (0.83-1.85) | |
| | | | | | | Grains, Native Hawaiian | ≤5.6 serv/(d<br>5.7-7.8<br>7.9-10.8<br>>10.8 | 1.00<br>1.37 (0.76-2.47)<br>0.78 (0.40-1.53)<br>1.27 (0.62-2.58) | |
| | | | | | | Grains, Japanese American | ≤5.6 serv/(d<br>5.7-7.8<br>7.9-10.8<br>>10.8 | 1.00<br>0.84 (0.64-1.10)<br>0.63 (0.47-0.84)<br>0.49 (0.35-0.69) | |
| | | | | | | Grains, Latinos | ≤5.6 serv/(d<br>5.7-7.8<br>7.9-10.8<br>>10.8 | 1.00<br>1.10 (0.82-1.45)<br>0.92 (0.66-1.30)<br>0.95 (0.63-1.42) | |
| | | | | | | Grains, Caucasian | ≤5.6 serv/(d<br>5.7-7.8<br>7.9-10.8<br>>10.8 | 1.00<br>0.86 (0.68-1.10)<br>0.82 (0.61-1.10)<br>1.17 (0.81-1.68) | |
| | | | | | | Grains, women, all | ≤4.5 serv/(d<br>4.6-6.4<br>6.5-8.9<br>>8.9 | 1.00<br>0.87 (0.75-1.02)<br>0.89 (0.74-1.06)<br>0.97 (0.78-1.22) | |
| | | | | | | Grains, African American | ≤4.5 serv/(d<br>4.6-6.4<br>6.5-8.9<br>>8.9 | 1.00<br>0.98 (0.75-1.28)<br>0.82 (0.59-1.13)<br>0.91 (0.62-1.34) | |
| | | | | | | Grains, Native Hawaiian | ≤4.5 serv/(d<br>4.6-6.4<br>6.5-8.9<br>>8.9 | 1.00<br>0.92 (0.47-1.82)<br>1.07 (0.51-2.23)<br>1.31 (0.54-3.15) | |

| | | | | | | | | | |
|---|---|---|---|---|---|---|---|---|---|
| | | | | | | Grains, Japanes5 American | ≤4.5 serv/(d<br>4.6-6.4<br>6.5-8.9<br>>8.9 | 1.00<br>0.62 (0.41-0.92)<br>0.86 (0.56-1.32)<br>1.03 (0.60-1.77) | |
| | | | | | | Grains, Latinos | ≤4.5 serv/(d<br>4.6-6.4<br>6.5-8.9<br>>8.9 | 1.00<br>1.02 (0.68-1.52)<br>1.17 (0.74-1.84)<br>1.33 (0.77-2.28) | |
| | | | | | | Grains, Caucasian | ≤4.5 serv/(d<br>4.6-6.4<br>6.5-8.9<br>>8.9 | 1.00<br>0.84 (0.63-1.12)<br>0.79 (0.56-1.12)<br>0.92 (0.59-1.43) | |
| Buil-Cosiales P et al, 2014, Spain | Prevencion con DietaMediterranea (PREDIMED) study | 2003-2009 – 2012, 5.9 years follow-up | 7216 men and women, age 55-75 years: 169 cancer deaths | Mortality | Validated FFQ, 137 food items | Whole grains | 0 g/d<br>1<br>7<br>33<br>89 | 1.00<br>0.94 (0.55-1.62)<br>0.91 (0.48-1.73)<br>0.83 (0.40-1.69)<br>0.75 (0.40-1.41) | Age, sex, smoking status, diabetes, BMI, SBP, DBP, recruitment center, statins, alcohol, education, physical activity, total energy, vegetables, fruits |
| Wu H et al, 2015, USA | Nurses' Health Study | 1984-2010, 26 years follow-up | 74341 women, age 38-63 years: 5964 cancer deaths | Mortality | Validated FFQ, 126 food items | Whole grains | 4.2 g/d<br>9.7<br>14.7<br>21.1<br>33.0 | 1.00<br>1.02 (0.94-1.10)<br>1.10 (1.02-1.19)<br>1.06 (0.98-1.15)<br>0.99 (0.91-1.07) | Age, ethnicity, BMI, smoking status, cigarettes per day, pack-years smoked, years since quitting smoking, alcohol, physical activity, family history of diabetes, cancer and heart disease, multivitamin use, aspirin use, hypertension, high cholesterol, diabetes, total energy, healthy eating index (excluding whole grains): women: postmenopausal status, HRT |

| | | | | | | | | | |
|---|---|---|---|---|---|---|---|---|---|
| Wu H et al, 2015, USA | Health Professionals Follow-up Study | 1986-2010, 24 years follow-up | 43744 men, age 32-87 years: 3921 cancer deaths | Mortality | Validated FFQ, 131 food items | Whole grains | 5.9 g/d<br>14.4<br>22.1<br>31.3<br>47.8 | 1.00<br>1.01 (0.92-1.11)<br>0.98 (0.88-1.08)<br>1.01 (0.91-1.12)<br>0.95 (0.86-1.05) | Age, ethnicity, BMI, smoking status, cigarettes per day, pack-years smoked, years since quitting smoking, alcohol, physical activity, family history of diabetes, cancer and heart disease, multivitamin use, aspirin use, hypertension, high cholesterol, diabetes, total energy, healthy eating index (excluding whole grains) |
| Wu H et al, 2015, USA | Nurses' Health Study & Health Professionals Follow-up Study | 1984-2010, 26 years follow-up 1986-2010, 24 years follow-up | 74341 women, age 38-63 years: 5964 cancer deaths 43744 men, age 32-87 years: 3921 cancer deaths | Mortality | Validated FFQ, 126/131 food items | Total bran<br><br><br><br><br>Total germ<br><br><br><br><br>Naturally occurring bran<br><br><br>Added bran<br><br><br><br><br>Refined grains | 1<br>2<br>3<br>4<br>5<br>1<br>2<br>3<br>4<br>5<br>0-0.4/0-0.4 g/d<br>0.5-1.4/0.5-1.9<br>1.5-2.99/2.0-3.9<br>≥3.0/≥4.0<br>0-0.4/0-0.4 g/d<br>0.5-1.4/0.5-1.9<br>1.5-2.9/2.0-3.9<br>3.0-5.9/4.0-9.9<br>≥6.0/≥10.0<br>Per 28 g/d | 1.00<br>1.06 (0.99-1.13)<br>1.07 (1.00-1.15)<br>1.11 (1.03-1.20)<br>1.04 (0.97-1.13)<br>1.00<br>1.04 (0.98-1.11)<br>1.01 (0.95-1.08)<br>0.96 (0.89-1.03)<br>0.98 (0.91-1.06)<br>1.00<br>1.02 (0.94-1.10)<br>1.06 (0.98-1.14)<br>1.02 (0.94-1.10)<br>1.00<br>1.09 (1.03-1.16)<br>1.09 (1.03-1.17)<br>1.12 (1.06-1.20)<br>1.04 (0.97-1.12)<br>0.98 (0.97-1.00) | Age, ethnicity, BMI, smoking status, cigarettes per day, pack-years smoked, years since quitting smoking, alcohol, physical activity, family history of diabetes, cancer and heart disease, multivitamin use, aspirin use, hypertension, high cholesterol, diabetes, total energy, healthy eating index (excluding whole grains), women: postmenopausal status, HRT |
| Huang T et al, 2015, USA | NIH-AARP Diet and Health Study | 1995-1996 – 2009, 14 years follow-up | 367442 men and women, age 50-71 years: 19043 cancer deaths | Mortality | Validated FFQ, 124 food items | Whole grains | 0.13 oz/1000 kcal/d[1]<br>0.30<br>0.47<br>0.69<br>1.20 | 1.00<br>0.94 (0.90-0.98)<br>0.91 (0.87-0.95)<br>0.88 (0.84-0.92)<br>0.85 (0.81-0.89) | Age, sex, number of cigarettes per day, time of smoking cessation, race/ ethnicity, alcohol, education, marital status, health status, BMI, physical activity, red meat, total fruit and vegetables, total energy, HRT (women) |

| | | | | | | | | | |
|---|---|---|---|---|---|---|---|---|---|
| Xu M et al, 2015, USA | NIH-AARP Diet and Health Study | 1995-1996 – 2008, 14 years follow-up | 367442 men and women, age 50-71 years: 19043 cancer deaths | Mortality | Validated FFQ, 124 food items | Ready-to-eat cereals | 0.00 g/d<br>0.67<br>3.48<br>9.33<br>22.48 | 1.00<br>0.98 (0.94-1.03)<br>0.95 (0.90-0.99)<br>0.92 (0.88-0.97)<br>0.90 (0.86-0.95) | Age, sex, smoking status, smoking dose, time since quitting smoking, race/ethnicity, education, marital status, self-rated health status, BMI, physical activity, menopausal hormone therapy, alcohol, red meat, fruits, vegetables, total energy |

| | | | | | | | | | |
|---|---|---|---|---|---|---|---|---|---|
| Johnsen NF et al, 2015, Norway, Sweden, Denmark | HELGA Cohort (Norwegian Women and Cancer Study, Northern Sweden Health and Disease Study, Danish Diet, Cancer and Health Study – part of the EPIC study) | 1992-1998 – 2008-2009, 11.1 (Norway), 14.2 (Sweden), 11.9 years (Denmark) follow-up | 120010 men and women, age 30-64 years: 1375/1775 cancer deaths | Mortality | Validated FFQ, 88 food items (Norway), 98 food items (Sweden) 173 food items (Denmark) | Whole grain breakfast cereals, women | 0 g/d<br>0.8<br>12<br>50 | 1.00<br>0.98 (0.83-1.15)<br>0.84 (0.74-0.95)<br>0.85 (0.74-0.98) | Age, follow-up time, education, smoking status/years since quit/ cigarettes per day, alcohol, BMI, total energy |
| | | | | | | Non-white bread | 25 g/d<br>80<br>113<br>180 | 1.00<br>1.01 (0.86-1.18)<br>0.93 (0.79-1.08)<br>0.89 (0.75-1.05) | |
| | | | | | | Crisp bread | 0.6 g/d<br>2<br>6<br>31 | 1.00<br>0.89 (0.77-1.03)<br>0.84 (0.75-0.95)<br>0.87 (0.74-1.02) | |
| | | | | | | Total whole grain products | 56 g/d<br>100<br>131<br>201 | 1.00<br>0.86 (0.76-0.98)<br>0.85 (0.75-0.97)<br>0.86 (0.74-0.99) | |
| | | | | | | Oat | 0 g/d<br>0.4<br>4<br>19 | 1.00<br>0.89 (0.78-1.01)<br>0.77 (0.67-0.90)<br>0.84 (0.71-0.99) | |
| | | | | | | Rye | 8 g/d<br>18<br>22<br>41 | 1.00<br>0.98 (0.87-1.11)<br>1.00 (0.86-1.17)<br>0.99 (0.79-1.25) | |
| | | | | | | Wheat | 0.4 g/d<br>3<br>10<br>37 | 1.00<br>0.76 (0.65-0.88)<br>0.74 (0.63-0.87)<br>0.74 (0.61-0.89) | |
| | | | | | | Total whole grain types | 20 g/d<br>33<br>49<br>74 | 1.00<br>0.99 (0.87-1.13)<br>0.94 (0.82-1.07)<br>0.88 (0.77-1.02) | |

| | | | | | | | | | | |
|---|---|---|---|---|---|---|---|---|---|---|
| | | | | | | Whole grain breakfast cereals, men | 0 g/d<br>0.8<br>7<br>50 | 1.00<br>0.99 (0.81-1.21)<br>0.73 (0.63-0.85)<br>0.75 (0.64-0.87) | |
| | | | | | | Non-white bread | 13 g/d<br>66<br>118<br>201 | 1.00<br>0.92 (0.76-1.12)<br>0.97 (0.79-1.19)<br>0.79 (0.64-0.97) | |
| | | | | | | Crisp bread | 1 g/d<br>2<br>4<br>34 | 1.00<br>0.99 (0.85-1.15)<br>0.92 (0.79-1.07)<br>0.83 (0.67-1.03) | |
| | | | | | | Total whole grain products | 64 g/d<br>107<br>156<br>222 | 1.00<br>0.84 (0.72-0.98)<br>0.74 (0.64-0.86)<br>0.70 (0.60-0.81) | |
| | | | | | | Oat | 0 g/d<br>0.4<br>3<br>30 | 1.00<br>0.80 (0.69-0.94)<br>0.75 (0.64-0.89)<br>0.75 (0.64-0.89) | |
| | | | | | | Rye | 7 g/d<br>21<br>38<br>56 | 1.00<br>0.97 (0.82-1.14)<br>0.80 (0.65-0.98)<br>0.93 (0.78-1.11) | |
| | | | | | | Wheat | 0.1 g/d<br>1<br>5<br>10 | 1.00<br>0.84 (0.72-0.98)<br>0.69 (0.58-0.82)<br>0.58 (0.49-0.69) | |
| | | | | | | Total whole grain types | 21 g/d<br>37<br>54<br>80 | 1.00<br>0.85 (0.73-0.99)<br>0.68 (0.58-0.79)<br>0.74 (0.63-0.87) | |

| | | | | | | | | | |
|---|---|---|---|---|---|---|---|---|---|
| Zhang R et al, 2016, USA | Nurses' Health Study | 1984-2010, 26 years follow-up | 70603 women, age 38-63 years: 15673 cancer cases | Incidence | Validated FFQ, 126 food items | Total rice<br><br><br><br>White rice<br><br><br><br>Brown rice | <1 serv/wk<br>1<br>2-4<br>≥5<br><1 serv/wk<br>1<br>2-4<br>≥5<br><1 serv/wk<br>1<br>2-4<br>≥5 | 1.00<br>1.01 (0.97-1.05)<br>1.00 (0.96-1.04)<br>1.02 (0.93-1.12)<br>1.00<br>1.01 (0.96-1.05)<br>1.02 (0.97-1.06)<br>0.96 (0.81-1.14)<br>1.00<br>1.02 (0.95-1.10)<br>1.00 (0.93-1.09)<br>1.07 (0.84-1.38) | Age, ethnicity, BMI, smoking status, cigarettes per day, physical activity, family history of cancer, multivitamin supplement use, total energy intake, alcohol, fruit, vegetables, red meat, fish, nuts, whole grain (except brown rice), sugar-sweetened beverages, HRT |
| Zhang R et al, 2016, USA | Nurses' Health Study 2 | 1991-2009, 18 years follow-up | 90264 women, age 27-44 years: 5149 cancer cases | Incidence | Validated FFQ, 133 food items | Total rice<br><br><br><br>White rice<br><br><br><br>Brown rice | <1 serv/wk<br>1<br>2-4<br>≥5<br><1 serv/wk<br>1<br>2-4<br>≥5<br><1 serv/wk<br>1<br>2-4<br>≥5 | 1.00<br>0.93 (0.86-1.00)<br>0.93 (0.86-0.99)<br>0.83 (0.73-0.94)<br>1.00<br>0.96 (0.89-1.03)<br>0.96 (0.90-1.03)<br>0.73 (0.59-0.90)<br>1.00<br>1.02 (0.93-1.13)<br>0.95 (0.85-1.06)<br>1.28 (0.96-1.70) | Age, ethnicity, BMI, smoking status, cigarettes per day, physical activity, family history of cancer, multivitamin supplement use, total energy intake, alcohol, fruit, vegetables, red meat, fish, nuts, whole grain (except brown rice), sugar-sweetened beverages, HRT |
| Zhang R et al, 2016, USA | Health Professionals Follow-up Study | 1986-2008, 22 years follow-up | 45382 men, age 40-75 years: 10833 cancer cases | Incidence | Validated FFQ, 131 food items | Total rice<br><br><br><br>White rice<br><br><br><br>Brown rice | <1 serv/wk<br>1<br>2-4<br>≥5<br><1 serv/wk<br>1<br>2-4<br>≥5<br><1 serv/wk<br>1<br>2-4<br>≥5 | 1.00<br>0.99 (0.94-1.04)<br>1.00 (0.95-1.05)<br>1.00 (0.93-1.10)<br>1.00<br>1.02 (0.97-1.07)<br>1.03 (0.98-1.08)<br>0.91 (0.79-1.05)<br>1.00<br>1.00 (0.94-1.07)<br>0.96 (0.91-1.05)<br>0.95 (0.77-1.17) | Age, ethnicity, BMI, smoking status, cigarettes per day, physical activity, family history of cancer, multivitamin supplement use, total energy intake, alcohol, fruit, vegetables, red meat, fish, nuts, whole grain (except brown rice), sugar-sweetened beverages |

BMI; body mass index, DBP; diastolic blood pressure, FFQ; food frequency questionnaire, HRT; hormone replacement therapy, SBP; systolic blood pressure, SFA; saturated fatty acids

[1] The original paper reports in ounces/d, after contact with the authors it was confirmed that ounces/1000 kcal/d is correct.

## Table S7. Whole grains and refined grains and all-cause mortality

| Author, publication year, country | Study name | Study period | Number of participants, gender, age, number of deaths | Dietary assessment | Exposure and subgroup | Whole grain consumption frequency or amount | Relative risks (95% confidence intervals) | Adjustment for confounding factors |
|---|---|---|---|---|---|---|---|---|
| Kahn HA et al, 1984, USA | Adventist Mortality Study | 1960-1980, 21 years follow-up | 27530 men and women, age ≥30 years: 5751 deaths | FFQ, 28 food items | Bread, rolls, biscuits<br><br><br><br>Cereal | <1 /wk<br>1-2<br>3-4<br>6-7<br><1 /wk<br>1-2<br>3-6<br>7 | 1.00<br>0.88 (0.80-0.96)<br>0.81 (0.69-0.95)<br>0.82 (0.73-0.92)[1]<br>1.00<br>0.80 (0.71-0.90)<br>0.77 (0.67-0.88)<br>0.84 (0.75-0.94)[1] | Age, sex, smoking, history of disease, age at initial exposure to the Seventh Day Adventist church |
| Rotevatn S et al, 1989, Norway | NA | 1964-1967 – 1978, 11.5 years follow-up | 10187 men, age 35-74 years: 2458 deaths | FFQ | Bread consumption | ≥6 vs. <6/d | 0.752 | Age, physical exercise, cigarette smoking, alcohol |
| Trichopoulou A, 1995, Greece, | NA | 1988-1990 – 1993-1994, ~3-6 years follow-up | 182 men and women, >70 years: 53 deaths | Validated FFQ, 198 food items/ beverages | Cereal | 20 g/d | 1.02 (0.97-1.07) | Age, smoking status, sex |
| Osler M et al, 1997, Denmark | Euronet SENECA study Denmark | 1988-1989 – 1995, NA | 202 men and women, mean age 73.4 years: 52 deaths | 3-day food record and frequency checklist of foods | Cereals | Per 20 g/d | 1.10 (1.03-1.17) | Age, sex, smoking |
| Fortes C et al, 2000, Italy | NA | 1993-1998, 5 years follow-up | 162 men and women, age ≥65 years: 53 deaths | FFQ, 114 food items | Pasta | <1/wk<br>1-4/wk<br>>4/wk | 1.00<br>0.56 (0.26-1.21)<br>0.61 (0.26-1.45) | Age, sex, education, BMI, smoking, cognitive function, chronic diseases |
| Jacobs DR et al, 2001, Norway | Norwegian County Study | 1977-1983 – 1994, 14.4 years follow-up | 33848 men and women, age 35-56 years: 2058 deaths | FFQ, 66 food items | Whole grain bread score | 0.05-0.60<br>0.83-0.83<br>0.90-1.13<br>1.35-1.80<br>2.25-5.40 | 1.00<br>0.87 (0.75-1.01)<br>0.80 (0.71-0.92)<br>0.85 (0.74-0.98)<br>0.75 (0.65-0.88) | Age, energy, sex, current smoking, past smoking, leisure-time physical activity, occupational physical activity, cod liver oil use, multivitamins, SFA intake, SBP, serum total cholesterol, BMI |

| | | | | | | | | |
|---|---|---|---|---|---|---|---|---|
| Appleby PN et al, 2002, UK | The Health Food Shoppers Study | 1973-1979 – 1997, 19.8 years follow-up | 11000 men and women, age 16-89 years: 2529 deaths | FFQ | Wholemeal bread<br>Bran cereals | Daily vs less<br>Daily vs less | 0.89 (0.82-0.98)<br>1.02 (0.94-1.12) | Age at recruitment, sex, smoking, fresh fruit, nuts/dried fruit, raw vegetables salads, mutual adjustment between wholemeal bread and bran cereals |
| Liu S et al, 2003, USA | Physicians' Health Study | 1982-1988, 5.5 years follow-up | 86190 men, age 40-84 years: 3114 deaths | Validated FFQ | Whole grain breakfast cereals<br><br><br><br>Refined-grain breakfast cereals<br><br><br><br>Total breakfast cereals | Rarely<br>1 serv/wk<br>2-6/wk<br>≥1/day<br>Rarely<br>1 serv/wk<br>2-6/wk<br>≥1/day<br>Rarely<br>1 serv/wk<br>2-6/wk<br>≥1/day | 1.00<br>0.88 (0.76-1.01)<br>0.85 (0.74-0.97)<br>0.83 (0.73-0.94)<br>1.00<br>1.15 (1.02-1.29)<br>1.08 (0.95-1.24)<br>1.09 (0.95-1.25)<br>1.00<br>1.02 (0.92-1.14)<br>0.94 (0.84-1.05)<br>0.92 (0.82-1.02) | Age, cigarette smoking, alcohol intake, physical activity, BMI, diabetes mellitus, high cholesterol, hypertension, multivitamin use |
| Steffen LM et al, 2003, USA | Atherosclerosis Risk in Communities Study | 1987-1989 - 1999, 11 years follow-up | 11940 men and women, age 45-64 years: 867 deaths | Validated FFQ, 61 food items | Whole grain<br><br><br><br><br>Refined grain | 0.1 serv./day<br>0.5<br>1.0<br>1.5<br>3.0<br>0.5<br>1.0<br>2.0<br>3.0<br>5.0 | 1.00<br>0.96 (0.79-1.17)<br>0.80 (0.65-0.99)<br>0.87 (0.70-1.08)<br>0.77 (0.61-0.97)<br>1.00<br>0.96 (0.75-1.23)<br>1.03 (0.81-1.31)<br>0.97 (0.76-1.23)<br>1.08 (0.83-1.40) | Age at baseline, race, sex, time-dependent energy intake, education, smoking status, pack-years of smoking, physical activity, alcohol intake, HRT-women, BMI, waist-to-hip ratio, SBP, antihypertensive medication use |
| Trichopoulou A et al, 2005, Greece | European Prospective Investigation into Cancer and Nutrition – Elderly Study | 1992-2000 – 1999-2003, 7.4 years follow-up | 74607 men and women, age 60 years: 4047 deaths | Validated FFQs, food records | Cereals | Per 104 g/d | 0.94 (0.91-0.98) | Age, sex, country, diabetes, waist-to-hip ratio, BMI, education, smoking status, occupational physical activity, leisure-time physical activity, alcohol, total energy |

| | | | | | | | | |
|---|---|---|---|---|---|---|---|---|
| Hays JC et al, 2005, USA | Established Population for Epidemiologic Studies of the Elderly – Duke University | 1992-1993 – 1996, 4 years follow-up | 1920 men and women, mean age 76.1 (whites)/ 77.0 (black): 226 deaths | Short interview | Grains, white men<br>Grains, black men<br>Grains, white women<br>Grains, black women | ≥2 vs. <2 serv/d<br>≥2 vs. <2 serv/d<br>≥2 vs. <2 serv/d<br>≥2 vs. <2 serv/d | 0.34 (0.10-1.19)<br>0.92 (0.40-2.16)<br>1.42 (0.56-3.58)<br>0.92 (0.54-1.60) | Age, lived alone, below poverty threshold, impaired food related activities of daily living, non-dairy protein, dairy, grains, smoking, alcohol, BMI, waist circumference, cognitive status, self-rated health |
| Knoops KTB et al, 2006, Europe | Healthy Ageing – a Longitudinal Study in Europe (HALE) | 1988-1991 - 2000, 10 years follow-up | 2068 men and 1049 women, mean age 73.7 years: 1382 deaths | Dietary history | Grains | ≥median vs. <median | 0.84 (0.77-0.92) | Age, sex, physical activity, smoking, alcohol, education, BMI, chronic disease at baseline, study centre |
| Sahyoun NR et al, 2006, USA | NA | 1981-1984 – 1995, 12-15 years follow-up | 535 men and women, age ≥60 years: 186 deaths | 3-day food record | Whole grain | 0.31 serv/d<br>0.86<br>1.49<br>2.90 | 1.00<br>1.08 (0.71-1.66)<br>1.24 (0.83-1.86)<br>0.82 (0.52-1.28) | Age, sex, race, education, marital status, smoking, alcohol intake, exercise, BMI, energy intake, SFA, antihypertensive or lipid-lowering therapy |
| Jacobs DR, 2007, USA | Iowa Women's Health Study | 1986 – 2003, 17 years follow-up | 27312 women, age 55-69 years: 5552 deaths | Validated FFQ, 127 food items | Whole grains<br><br><br><br><br>Refined grains | 1.8 serv/wk<br>5.6<br>8.8<br>14.5<br>25.6<br>0-5.75 serv/wk<br>6-9.5<br>9.6-13.5<br>14-22<br>≥22.5 | 1.00<br>0.88 (0.81-0.96)<br>0.88 (0.81-0.96)<br>0.80 (0.73-0.87)<br>0.79 (0.72-0.87)<br>1.00<br>0.98 (0.90-1.06)<br>0.95 (0.87-1.04)<br>0.92 (0.84-1.01)<br>1.01 (0.91-1.12) | Age, energy intake, BMI, waist-to-hip ratio, smoking, education, physical activity, HRT, multivitamin supplement use, intake of alcohol, coffee, red meat, fish and seafood, total fruit and vegetables, mutual adjustment between whole grains and refined grains |
| Iso H et al, 2007, Japan | Japan Collaborative Cohort Study | 1988-1990-2003, ~12.8 years follow-up | 42513 men and 57777 women, age 40-79 years: 9560/6575 deaths | FFQ | Rice, men<br><br><br>Rice, women | <3/d<br>3-4<br>≥5<br><3/d<br>3-4<br>≥5 | 1.00<br>0.92 (0.88-0.97)<br>0.78 (0.73-0.84)<br>1.00<br>0.94 (0.80-0.99)<br>0.95 (0.88-1.03) | Age, area of study |
| Trichopoulou A et al, 2009, Greece | European Prospective Investigation into Cancer and Nutrition - Greece | 1994-1997 - 2002, 8.5 years follow-up | 23349 men and women, age 20-86 years: 1075 deaths | Validated FFQ, 150 food items | Cereals | <median<br>≥Median | 1.00<br>0.99 (0.86-1.13) | Age, sex, education, smoking status, waist-to-hip ratio, BMI, physical activity, total energy intake |

| | | | | | | | | |
|---|---|---|---|---|---|---|---|---|
| Buckland G et al, 2011, Spain | European Prospective Investigation into Cancer and Nutrition – Spain cohort | 1992/1996 - 2008, 13.4 years follow-up | 40622 men and women, age 26-69 years: 1855 deaths | Validated DHQ, 600 food items | Cereals | <151.8 g/d<br>151.8-<214.0<br>≥214.0 | 1.00<br>0.92 (0.82-1.03)<br>0.91 (0.81-1.03) | Age, sex, centre, BMI, waist circumference, education level, physical activity, smoking status and intensity, total energy intake |
| Olsen A et al, 2011, Denmark | Diet, Cancer, and Health Study | 1993-1997 – 2008, 12 years follow-up | 50290 men and women, age 50-64 years: 4126 deaths | Validated FFQ, 192 food items | Rye bread, men<br><br>Oatmeal<br><br>Rye bread, women<br><br>Oatmeal | <63 g/d<br>≥63<br><21 g/d<br>≥21<br><63 g/d<br>≥63<br><21 g/d<br>≥21 | 1.00<br>0.84 (0.75-0.94)<br>1.00<br>0.91 (0.82-1.02)<br>1.00<br>0.90 (0.80-1.01)<br>1.00<br>0.97 (0.84-1.11) | Age, time under study, smoking status, smoking duration, current tobacco consumption, time since cessation, alcohol, education, participation in sports, time spent in sports per week, BMI, red meat, processed meat, total energy, fish, cabbages, apples and pears, root vegetables |
| Van den Brandt PA et al, 2011, Netherlands | Netherlands Cohort Study | 1986-1996, ~11 years follow-up | 120852 men and women, age 55-69 years: 9691 deaths 3576 subcohort members | Validated FFQ, 150 food items | Whole grains, men<br>Whole grains, women | Per 10.6 g/d<br>Per 13.5 g/d | 1.01 (0.99-1.02)<br>1.00 (0.98-1.03) | Age, cigarette smoking, cigarettes per day, years of smoking, BMI, nonoccupational physical activity, hypertension, education, energy intake |
| Tognon G et al, 2011, Sweden | Gerontological and Geriatric Population Studies in Gothenburg | 1971, 1981, 1992, 2000 – 2009, 8.5 years follow-up | 1037 men and women, age 70 years: 630 deaths | Dietary history | Whole grains cereals<br>Cereals | H vs. l<br>H vs. l | 0.85 (0.73-1.00)<br>1.01 (0.86-1.19) | Age, sex, baseline BMI, waist circumference, physical activity, marital status, smoking status, birth cohort, education |
| Martinez-Gonzalez MA et al, 2012, Spain | Seguimiento Universidad de Navarra Project | 1999 – 2009, 6.8 years follow-up | 15535 men and women, mean age 38 years: 185 deaths | Validated FFQ, 136 food items | Cereals | ≥median vs. <median | 0.83 (0.54-1.27) | Age, years of university education, BMI, smoking, physical activity, hours spent watching television, history of depression, hypertension, hypercholesterolemia, total energy, egg intake, potato, adoption of special diets, MUFA/SFA ratio, fruits/nuts, vegetables, legumes, fish, meat/meat products, dairy, alcohol |

| | | | | | | | | |
|---|---|---|---|---|---|---|---|---|
| Sluik D et al, 2014, Europe | European Prospective Investigation into Cancer and Nutrition | 1992-2000 - NA, 9.9 years follow-up | 265295 men and women, age 30-70 years: 830/12135 deaths (diabetes/no diabetes) | Validated FFQs, food records and question-naires | Pasta, diabetes<br>Rice<br>Bread<br>Breakfast cereals<br>Pasta, no diabetes<br>Rice<br>Bread<br>Breakfast cereals | Per 10 g/d<br>Per 10 g/d<br>Per 100 g/d[2]<br>Per 10 g/d<br>Per 10 g/d<br>Per 10 g/d<br>Per 100 g/d[2]<br>Per 10 g/d | 0.93 (0.90-0.96)<br>0.93 (0.86-1.00)<br>0.87 (0.71-1.07)<br>1.00 (0.97-1.02)<br>0.99 (0.98-1.00)<br>0.96 (0.94-0.98)<br>0.81 (0.76-0.82)<br>0.97 (0.97-0.98) | Age, centre, sex, prevalence of heart disease, cancer, stroke, education, diabetes medication use, alcohol, smoking behavior, physical activity, underlying dietary patterns |
| Li K et al, 2014, Germany | European Prospective Investigation into Cancer and Nutrition - Heidelberg Cohort | 1994-1998 – 2009, 11 years follow-up | 10235 men and 12234 women, age ≥40 years: 1599 deaths | Validated FFQ, 148 food items | Cereals, men<br>Cereals, women | Low vs. high<br>Low vs. high | 1.08 (0.96-1.23)<br>1.04 (0.86-1.26) | Age, smoking status, duration and cigarettes per day, BMI, alcohol, leisure-time physical activity, red and processed meat, vegetables and fruits, fish, dairy products |
| Tognon G et al, 2014, Denmark | The 1982-83 Danish Monitoring trends and determinants of Cardiovascular disease study (MONICA) | 1982-1983 – 2007, 14 years follow-up | 948 women and 901 men, age NA: 553 deaths | 7 day food record | Cereals | >median vs. <median | 0.97 (0.82-1.15) | Age, sex, BMI, education, physical activity, cigarette smoking |
| Atkins JL et al, 2014, United Kingdom | British Regional Heart Study | 1998-2000 – 2010, 11.3 years follow-up | 3328 men, age 60-79 years: 933 deaths | Validated FFQ, 86 food items | Cereals<br>Bread | Daily vs. <1 day/wk<br>Whole grain vs. none | 1.15 (0.87-1.52)<br>0.77 (0.32-1.90) | Age, smoking, alcohol, physical activity, social class, BMI, energy intake, diet score without respective components |
| Buil-Cosiales P et al, 2014, Spain | Prevencion con DietaMediterranea (PREDIMED) study | 2003-2009 – 2012, 5.9 years follow-up | 7216 men and women, age 55-75 years: 425 deaths | Validated FFQ, 137 food items | Whole grains, baseline<br><br><br><br>Whole grains, updated | 0 g/d<br>5<br>19<br>84<br>0 g/d<br>1<br>7<br>33<br>89 | 1.00<br>0.78 (0.52-1.17)<br>0.67 (0.43-1.04)<br>0.92 (0.64-1.33)<br>1.00<br>0.90 (0.65-1.26)<br>0.81 (0.55-1.19)<br>0.90 (0.63-1.30)<br>0.93 (0.65-1.31) | Age, sex, smoking status, diabetes, BMI, SBP, DBP, intervention group, recruitment center, statins, alcohol, education, physical activity, total energy, vegetables, fruits |
| Wu H et al, 2015, USA | Nurses' Health Study | 1984-2010, 26 years follow-up | 74341 women, age 38-63 years: 15106 deaths | Validated FFQ, 126 food items | Whole grains | 4.2 g/d<br>9.7<br>14.7 | 1.00<br>0.98 (0.93-1.03)<br>1.00 (0.95-1.05) | Age, ethnicity, BMI, smoking status, cigarettes per day, pack-years smoked, years since |

| | | | | | | | | |
|---|---|---|---|---|---|---|---|---|
| | | | | | | 21.1<br>33.0 | 0.94 (0.89-0.99)<br>0.88 (0.84-0.93) | quitting smoking, alcohol, physical activity, family history of diabetes, cancer and heart disease, multivitamin use, aspirin use, hypertension, high cholesterol, diabetes, total energy, healthy eating index (including fruits, vegetables, nuts and legumes, red or processed meat, sugar-sweetened beverages, alcohol, sodium, trans fat, long-chain n-3 FA, other PUFAs, but excluding whole grains), postmenopausal status, HRT |
| Wu H et al, 2015, USA | Health Professionals Follow-up Study | 1986-2010, 24 years follow-up | 43744 men, age 32-87 years: 11814 deaths | Validated FFQ, 133 food items | Whole grains | 5.9 g/d<br>14.4<br>22.1<br>31.3<br>47.8 | 1.00<br>1.00 (0.94-1.05)<br>0.97 (0.91-1.02)<br>1.01 (0.95-1.07)<br>0.95 (0.89-1.00) | Age, ethnicity, BMI, smoking status, cigarettes per day, pack-years smoked, years since quitting smoking, alcohol, physical activity, family history of diabetes, cancer and heart disease, multivitamin use, aspirin use, hypertension, high cholesterol, diabetes, total energy, healthy eating index (including fruits, vegetables, nuts and legumes, red or processed meat, sugar-sweetened beverages, alcohol, sodium, trans fat, long-chain n-3 FA, other PUFAs, but excluding whole grains) |
| Wu H et al, 2015, USA | Nurses' Health Study & Health Professionals Follow-up Study | 1984-2010, 26 years follow-up<br>1986-2010, 24 years follow-up | 74341 women, age 38-63 years: 15106 deaths<br>43744 men, age 32-87 years: 11814 deaths | Validated FFQ, | Total bran<br><br><br><br><br>Total germ | 1<br>2<br>3<br>4<br>5<br>1<br>2 | 1.00<br>1.00 (0.96-1.04)<br>0.99 (0.95-1.04)<br>1.00 (0.95-1.04)<br>0.94 (0.90-0.99)<br>1.00<br>1.02 (0.98-1.06) | Age, ethnicity, BMI, smoking status, cigarettes per day, pack-years smoked, years since quitting smoking, alcohol, physical activity, family history of diabetes, cancer and heart disease, multivitamin use, |

| | | | | | | | | |
|---|---|---|---|---|---|---|---|---|
| | | | | | Naturally occurring bran<br>Added bran<br>Refined grains | 3<br>4<br>5<br>0-0.4/0-0.4 g/d<br>0.5-1.4/0.5-1.9<br>1.5-2.99/2.0-3.9<br>≥3.0/≥4.0<br>0-0.4/0-0.4 g/d<br>0.5-1.4/0.5-1.9<br>1.5-2.9/2.0-3.9<br>3.0-5.9/4.0-9.9<br>≥6.0/≥10.0<br>Per 28 g/d | 0.98 (0.94-1.03)<br>0.97 (0.93-1.02)<br>1.00 (0.96-1.05)<br>1.00<br>0.99 (0.94-1.03)<br>0.98 (0.93-1.02)<br>0.93 (0.89-0.98)<br>1.00<br>0.99 (0.95-1.02)<br>0.99 (0.95-1.03)<br>1.00 (0.97-1.04)<br>0.93 (0.89-0.97)<br>0.98 (0.97-0.99) | aspirin use, hypertension, high cholesterol, diabetes, total energy, healthy eating index (including fruits, vegetables, nuts and legumes, red or processed meat, sugar-sweetened beverages, alcohol, sodium, trans fat, long-chain n-3 FA, other PUFAs, but excluding whole grains), women: postmenopausal status, HRT |
| Boggs DA et al, 2015, USA | Black Women's Health Study | 1995-2001 – 2011, 16 years follow-up | 37001 women, age 30-69 years: 1678 deaths | Validated FFQ, 68 food items | Whole grains | 0.01 serv/d<br>0.11<br>0.32<br>0.62<br>1.44 | 1.00<br>0.96 (0.83-1.12)<br>0.82 (0.70-0.97)<br>0.85 (0.73-0.99)<br>0.75 (0.64-0.89) | Age, total energy intake, education, marital status, vigorous exercise, TV watching, smoking, alcohol, vegetables, fruits, nuts/legumes, low-fat dairy, red or processed meat, sugar-sweetened beverages, sodium |
| Huang T et al, 2015, USA | NIH-AARP Diet and Health Study | 1995-1996 – 2009, 14 years follow-up | 367442 men and women, age 50-71 years: 46067 deaths | Validated FFQ, 124 food items | Whole grains | 0.13 oz/1000 kcal/d[3]<br>0.30<br>0.47<br>0.69<br>1.20 | 1.00<br>0.93 (0.90-0.95)<br>0.89 (0.87-0.92)<br>0.85 (0.82-0.87)<br>0.83 (0.81-0.86) | Age, sex, number of cigarettes per day, time of smoking cessation, race/ ethnicity, alcohol, education, marital status, health status, BMI, physical activity, red meat, total fruit and vegetables, total energy, HRT (women) |
| Xu M et al, 2015, USA | NIH-AARP Diet and Health Study | 1995-1996 – 2008, 14 years follow-up | 367442 men and women, age 50-71 years: 46067 deaths | Validated FFQ, 124 food items | Ready-to-eat cereals | 0.00 g/d<br>0.67<br>3.48<br>9.33<br>22.48 | 1.00<br>0.94 (0.92-0.97)<br>0.92 (0.89-0.95)<br>0.88 (0.86-0.91)<br>0.85 (0.83-0.88) | Age, sex, smoking status, smoking dose, time since quitting smoking, race/ethnicity, education, marital status, self-rated health status, BMI, physical activity, menopausal hormone therapy, alcohol, red meat, fruits, vegetables, total energy |

| | | | | | | | | |
|---|---|---|---|---|---|---|---|---|
| Prinelli F et al, 2015, Italy | NA | 1991-1995 – 2012, 17.4 years follow-up | 974 men and women, age 40-74 years: 193 deaths | FFQ, 158 food items | Cereals | >median vs. ≤median | 0.91 (0.66-1.26) | Age, sex, education, BMI, physical activity, smoking status, time spent watching TV, energy intake, vegetables, legumes, fruits, potatoes, fish and seafood, dairy products, red meat and meat products, poultry, olive oil, ethanol |
| Roswall N et al, 2015, Sweden | Swedish Women's Lifestyle and Health Cohort | 1991-1992 - 2012, 21.3 years follow-up | 44961 women, age 29-49 years: 1855 deaths | Validated FFQ, ~80 food items | Whole grain bread<br><br>Oatmeal | <median<br>≥median<br><median<br>≥median | 1.00<br>0.83 (0.76-0.92)<br>1.00<br>0.99 (0.90-1.09) | Age, smoking status, duration, current tobacco consumption, time since smoking cessation, education, BMI, alcohol, red meat, processed meat, energy intake |
| Johnsen NF et al, 2015, Norway, Sweden, Denmark | HELGA Cohort (Norwegian Women and Cancer Study, Northern Sweden Health and Disease Study, Danish Diet, Cancer and Health Study – part of the EPIC study) | 1992-1998 – 2008-2009, 11.1 (Norway), 14.2 (Sweden), 11.9 years (Denmark) follow-up | 120010 men and women, age 30-64 years: 4181/3658 deaths | Validated FFQ, 88 food items (Norway), 98 food items (Sweden) 173 food items (Denmark) | Whole grain breakfast cereals, women<br><br><br><br>Non-white bread<br><br><br><br>Crisp bread<br><br><br><br>Total whole grain products<br><br><br><br>Oat<br><br><br><br>Rye<br><br><br><br>Wheat | 0 g/d<br>0.8<br>12<br>50<br>25 g/d<br>80<br>113<br>180<br>0.6 g/d<br>2<br>6<br>31<br>56 g/d<br>100<br>131<br>201<br>0 g/d<br>0.4<br>4<br>19<br>8 g/d<br>18<br>22<br>41<br>0.4 g/d | 1.00<br>0.77 (0.67-0.89)<br>0.79 (0.71-0.88)<br>0.75 (0.69-0.82)<br>1.00<br>0.84 (0.76-0.93)<br>0.74 (0.65-0.84)<br>0.72 (0.65-0.81)<br>1.00<br>0.90 (0.82-0.99)<br>0.86 (0.78-0.94)<br>0.91 (0.81-1.01)<br>1.00<br>0.78 (0.71-0.86)<br>0.77 (0.71-0.85)<br>0.68 (0.62-0.75)<br>1.00<br>0.85 (0.78-0.93)<br>0.74 (0.67-0.82)<br>0.78 (0.70-0.87)<br>1.00<br>0.92 (0.84-1.01)<br>0.81 (0.73-0.90)<br>0.93 (0.83-1.03)<br>1.00 | Age, follow-up time, education, smoking status/years since quit/ cigarettes per day, alcohol, BMI, total energy |

| | | | | | | | | |
|---|---|---|---|---|---|---|---|---|
| | | | | | | 3 | 0.72 (0.65-0.79) | |
| | | | | | | 10 | 0.65 (0.58-0.72) | |
| | | | | | | 37 | 0.63 (0.53-0.74) | |
| | | | | | Total whole grain types | 20 g/d | 1.00 | |
| | | | | | | 33 | 0.80 (0.73-0.87) | |
| | | | | | | 49 | 0.74 (0.67-0.81) | |
| | | | | | | 74 | 0.74 (0.67-0.81) | |
| | | | | | Whole grain breakfast cereals, men | 0 g/d | 1.00 | |
| | | | | | | 0.8 | 0.92 (0.82-1.04) | |
| | | | | | | 7 | 0.82 (0.76-0.89) | |
| | | | | | | 50 | 0.74 (0.68-0.81) | |
| | | | | | Non-white bread | 13 g/d | 1.00 | |
| | | | | | | 66 | 0.92 (0.83-1.03) | |
| | | | | | | 118 | 0.85 (0.75-0.95) | |
| | | | | | | 201 | 0.78 (0.69-0.88) | |
| | | | | | Crisp bread | 1 g/d | 1.00 | |
| | | | | | | 2 | 0.97 (0.89-1.06) | |
| | | | | | | 4 | 0.94 (0.86-1.02) | |
| | | | | | | 34 | 1.03 (0.90-1.17) | |
| | | | | | Total whole grain products | 64 g/d | 1.00 | |
| | | | | | | 107 | 0.87 (0.80-0.95) | |
| | | | | | | 156 | 0.74 (0.68-0.81) | |
| | | | | | | 222 | 0.75 (0.68-0.81) | |
| | | | | | Oat | 0 g/d | 1.00 | |
| | | | | | | 0.4 | 0.87 (0.80-0.95) | |
| | | | | | | 3 | 0.85 (0.77-0.94) | |
| | | | | | | 30 | 0.76 (0.69-0.85) | |
| | | | | | Rye | 7 g/d | 1.00 | |
| | | | | | | 21 | 0.91 (0.83-1.00) | |
| | | | | | | 38 | 0.82 (0.74-0.91) | |
| | | | | | | 56 | 0.86 (0.78-0.95) | |
| | | | | | Wheat | 0.1 g/d | 1.00 | |
| | | | | | | 1 | 0.87 (0.80-0.95) | |
| | | | | | | 5 | 0.76 (0.69-0.84) | |
| | | | | | | 10 | 0.71 (0.64-0.78) | |
| | | | | | Total whole grain types | 21 g/d | 1.00 | |
| | | | | | | 37 | 0.82 (0.75-0.90) | |
| | | | | | | 54 | 0.72 (0.66-0.78) | |
| | | | | | | 80 | 0.75 (0.68-0.82) | |

| | | | | | | | | |
|---|---|---|---|---|---|---|---|---|
| Shi Z et al, 2015, China | Chinese Longitudinal Health Longevity Survey | 1998-1999 - 2011, 4.3 years follow-up | 8959 men and women, age ≥80 years: 6626 deaths | FFQ, 10 items | Staple food (total grains: rice, corn, wheat, other) | ≤200 g/d<br>250-300<br>350-400<br>≥450 | 1.00<br>0.92 (0.86-0.98)<br>0.92 (0.85-0.99)<br>0.91 (0.84-0.98) | Age, sex, job before 60 years age, residence, smoking, alcohol, physical activity, number of chronic diseases, fruit and vegetables |
| Wang JB et al, 2016, China[2] | Linxian Nutrition Intervention Trial cohort | 1984-1991 - 2010, 19-26 years follow-up | 2445 men and women, age 40-69 years: 1501 deaths | FFQ, 64 food items | All grains<br>Non-whole grains<br>Whole grains | Per 1 time/day<br>Per 1 time/day<br>Per 1 time/day | 0.96 (0.93-1.00)<br>0.96 (0.92-1.01)<br>0.98 (0.93-1.05) | Age, sex, commune, smoking, drinking, season, BMI |
| Bongard V et al, 2016, France | MONitoring of trends and determinants in CArdiovascular disease (MONICA) Project | 1995-1997 - 2010, 14.8 years follow-up | 960 men, age 45-64 years: 150 deaths | 3-day food record | Cereals<br><br><br><br>Bread | <21 g/d<br>21-<47<br>47-<78<br>≥78<br><80 g/d<br>80-<120<br>120-<170<br>≥170 | 1.00<br>0.60 (0.39-0.92)<br>0.50 (0.31-0.81)<br>0.76 (0.48-1.19)<br>1.00<br>0.69 (0.45-1.07)<br>0.43 (0.26-0.70)<br>0.80 (0.51-1.26) | Age, center, payment of income tax, obesity, alcohol, smoking status, physical activity, presence of a serious chronic condition, diet quality score |

BMI; body mass index, DBP; diastolic blood pressure, FA; fatty acids, FFQ; food frequency questionnaire, HRT; hormone replacement therapy, MUFA; monounsaturated fatty acids, PUFA; polyunsaturated fatty acids, SBP; systolic blood pressure, SFA; saturated fatty acids, TV; television

[1] The study reported 99% confidence intervals which have been recalculated to 95% confidence intervals.

[2] The original paper reports per 10 g/d for bread, but after contact with authors it was confirmed that 100 g/d is correct.

[3] The original paper reports in ounces/d, after contact with the authors it was confirmed that ounces/1000 kcal/d is correct.

**Table S8. Whole grains and refined grains and respiratory disease mortality**

| Author, publication year, country | Study name | Study period | Number of participants, gender, age, number of cases/deaths | Dietary assessment | Exposure and subgroup | Whole grain consumption frequency or amount | Relative risks (95% confidence intervals) | Adjustment for confounding factors |
|---|---|---|---|---|---|---|---|---|
| Jacobs DR, 2007, USA | Iowa Women's Health Study | 1986 – 2003, 17 years follow-up | 27312 women, age 55-69 years: 569 respiratory disease deaths | Validated FFQ, 127 food items | Whole grains | 1.8 serv/wk<br>5.6<br>8.8<br>14.5<br>25.6 | 1.00<br>0.65 (0.51-0.83)<br>0.65 (0.50-0.84)<br>0.58 (0.44-0.76)<br>0.60 (0.46-0.80) | Age, energy intake, BMI, waist-to-hip ratio, smoking, education, physical activity, HRT, multivitamin supplement use, intake of alcohol, coffee, red meat, fish and seafood, total fruit and vegetables, mutual adjustment between whole grains and refined grains |
| Wu H et al, 2015, USA | Nurses' Health Study & Health Professionals Follow-up Study | 1984-2010, 26 years follow-up 1986-2010, 24 years follow-up | 74341 women, age 38-63 years and 43744 men, age 32-87 years: 2016 respiratory disease deaths | Validated FFQ, 126/133 food items | Whole grains | 4.2/5.9 g/d (w/m)<br>9.7/14.4<br>14.7/22.1<br>21.1/31.1<br>33.0/47.8 | 1.00<br>0.96 (0.84-1.08)<br>1.00 (0.87-1.14)<br>0.88 (0.76-1.02)<br>0.89 (0.77-1.03) | Age, ethnicity, BMI, smoking status, cigarettes per day, pack-years smoked, years since quitting smoking, alcohol, physical activity, family history of diabetes, cancer and heart disease, multivitamin use, aspirin use, hypertension, high cholesterol, diabetes, total energy, healthy eating index (including fruits, vegetables, nuts and legumes, red or processed meat, sugar-sweetened beverages, alcohol, sodium, trans fat, long-chain n-3 FA, other PUFAs, but excluding whole grains), women: postmenopausal status, HRT |

| | | | | | | | | |
|---|---|---|---|---|---|---|---|---|
| Huang T et al, 2015, USA | NIH-AARP Diet and Health Study | 1995-1996 – 2009, 14 years follow-up | 367442 men and women, age 50-71 years: 3796 respiratory disease deaths | Validated FFQ, 124 food items | Whole grains | 0.13 oz/1000 kcal/d[1]<br>0.30<br>0.47<br>0.69<br>1.20 | 1.00<br>0.99 (0.90-1.09)<br>0.94 (0.85-1.03)<br>0.91 (0.82-1.01)<br>0.89 (0.80-0.98) | Age, sex, number of cigarettes per day, time of smoking cessation, race/ ethnicity, alcohol, education, marital status, health status, BMI, physical activity, red meat, total fruit and vegetables, total energy, HRT (women) |
| Xu M et al, 2015, USA | NIH-AARP Diet and Health Study | 1995-1996 – 2008, 14 years follow-up | 367442 men and women, age 50-71 years: 3796 respiratory disease deaths | Validated FFQ, 124 food items | Ready-to-eat cereals | 0.00 g/d<br>0.67<br>3.48<br>9.33<br>22.48 | 1.00<br>0.95 (0.88-1.03)<br>0.94 (0.87-1.02)<br>0.91 (0.84-0.99)<br>0.91 (0.83-0.99) | Age, sex, smoking status, smoking dose, time since quitting smoking, race/ethnicity, education, marital status, self-rated health status, BMI, physical activity, menopausal hormone therapy, alcohol, red meat, fruits, vegetables, total energy |

| | | | | | | | | |
|---|---|---|---|---|---|---|---|---|
| Johnsen NF et al, 2015, Norway, Sweden, Denmark | HELGA Cohort (Norwegian Women and Cancer Study, Northern Sweden Health and Disease Study, Danish Diet, Cancer and Health Study – part of the EPIC study) | 1992-1998 – 2008-2009, 11.1 (Norway), 14.2 (Sweden), 11.9 years (Denmark) follow-up | 120010 men and women, age 30-64 years: 111/125 respiratory disease deaths | Validated FFQ, 88 food items (Norway), 98 food items (Sweden) 173 food items (Denmark) | Whole grain breakfast cereals, women | 0 g/d<br>0.8<br>12<br>50 | 1.00<br>0.28 (0.11-0.69)<br>0.48 (0.28-0.83)<br>0.39 (0.23-0.65) | Age, follow-up time, education, smoking status/years since quit/ cigarettes per day, alcohol, BMI, total energy |
| | | | | | Non-white bread | 25 g/d<br>80<br>113<br>180 | 1.00<br>0.87 (0.50-1.53)<br>0.47 (0.24-0.93)<br>0.74 (0.41-1.35) | |
| | | | | | Crisp bread | 0.6 g/d<br>2<br>6<br>31 | 1.00<br>0.80 (0.51-1.25)<br>0.59 (0.32-1.07)<br>1.03 (0.59-1.79) | |
| | | | | | Total whole grain products | 56 g/d<br>100<br>131<br>201 | 1.00<br>0.56 (0.35-0.89)<br>0.53 (0.33-0.86)<br>0.82 (0.49-1.37) | |
| | | | | | Oat | 0 g/d<br>0.4<br>4<br>19 | 1.00<br>0.56 (0.36-0.88)<br>0.48 (0.27-0.85)<br>0.51 (0.28-0.93) | |
| | | | | | Rye | 8 g/d<br>18<br>22<br>41 | 1.00<br>1.62 (0.86-3.04)<br>1.12 (0.61-2.05)<br>1.12 (0.60-2.10) | |
| | | | | | Wheat | 0.4 g/d<br>3<br>10<br>37 | 1.00<br>0.63 (0.41-0.99)<br>0.57 (0.34-0.94)<br>0.44 (0.09-2.05) | |
| | | | | | Total whole grain types | 20 g/d<br>33<br>49<br>74 | 1.00<br>0.65 (0.40-1.08)<br>0.64 (0.39-1.04)<br>0.47 (0.28-0.77) | |
| | | | | | Whole grain breakfast cereals, men | 0 g/d<br>0.8<br>7<br>50 | 1.00<br>1.50 (0.79-2.82)<br>1.23 (0.76-1.98)<br>0.66 (0.37-1.16) | |

| | | | | | | | | |
|---|---|---|---|---|---|---|---|---|
| | | | | | Non-white bread | 13 g/d | 1.00 | |
| | | | | | | 66 | 1.04 (0.52-2.08) | |
| | | | | | | 118 | 0.62 (0.28-1.35) | |
| | | | | | | 201 | 0.94 (0.46-1.94) | |
| | | | | | Crisp bread | 1 g/d | 1.00 | |
| | | | | | | 2 | 0.77 (0.45-1.32) | |
| | | | | | | 4 | 0.98 (0.59-1.62) | |
| | | | | | | 34 | 1.00 (0.50-2.01) | |
| | | | | | Total whole grain products | 64 g/d | 1.00 | |
| | | | | | | 107 | 1.03 (0.61-1.76) | |
| | | | | | | 156 | 1.05 (0.63-1.77) | |
| | | | | | | 222 | 0.60 (0.33-1.07) | |
| | | | | | Oat | 0 g/d | 1.00 | |
| | | | | | | 0.4 | 0.99 (0.59-1.68) | |
| | | | | | | 3 | 0.87 (0.50-1.51) | |
| | | | | | | 30 | 0.52 (0.28-0.97) | |
| | | | | | Rye | 7 g/d | 1.00 | |
| | | | | | | 21 | 1.16 (0.65-2.10) | |
| | | | | | | 38 | 0.70 (0.32-1.57) | |
| | | | | | | 56 | 1.02 (0.53-1.97) | |
| | | | | | Wheat | 0.1 g/d | 1.00 | |
| | | | | | | 1 | 1.46 (0.83-2.55) | |
| | | | | | | 5 | 1.40 (0.76-2.56) | |
| | | | | | | 10 | 1.06 (0.57-1.95) | |
| | | | | | Total whole grain types | 21 g/d | 1.00 | |
| | | | | | | 37 | 0.96 (0.58-1.61) | |
| | | | | | | 54 | 0.65 (0.38-1.11) | |
| | | | | | | 80 | 0.74 (0.42-1.30) | |

BMI; body mass index, FA; fatty acids, FFQ; food frequency questionnaire, HRT; hormone replacement therapy, PUFA; polyunsaturated fatty acids

[1] The original paper reports in ounces/d, but after contact with the authors it was confirmed that ounces/1000 kcal/d is correct.

**Table S9. Whole grains and refined grains and infectious disease mortality**

| Author, publication year, country | Study name | Study period | Number of participants, gender, age, number of cases/deaths | Dietary assessment | Exposure and subgroup | Whole grain consumption frequency or amount | Relative risks (95% confidence intervals) | Adjustment for confounding factors |
|---|---|---|---|---|---|---|---|---|
| Jacobs DR, 2007, USA | Iowa Women's Health Study | 1986 – 2003, 17 years follow-up | 27312 women, age 55-69 years: 59 infectious disease deaths | Validated FFQ, 127 food items | Whole grains | 1.8 serv/wk<br>5.6<br>8.8<br>14.5<br>25.6 | 1.00<br>0.89 (0.39-2.02)<br>0.92 (0.41-2.09)<br>1.03 (0.46-2.33)<br>0.68 (0.26-1.75) | Age, energy intake, BMI, waist-to-hip ratio, smoking, education, physical activity, HRT, multivitamin supplement use, intake of alcohol, coffee, red meat, fish and seafood, total fruit and vegetables, mutual adjustment between whole grains and refined grains |
| Wu H et al, 2015, USA | Nurses' Health Study & Health Professionals Follow-up Study | 1984-2010, 26 years follow-up 1986-2010, 24 years follow-up | 74341 women, age 38-63 years and 43744 men, age 32-87 years: 405 infectious disease deaths | Validated FFQ, 126/133 food items | Whole grains | 4.2/5.9 g/d (w/m)<br>9.7/14.4<br>14.7/22.1<br>21.1/31.1<br>33.0/47.8 | 1.00<br>0.88 (0.65-1.18)<br>0.76 (0.55-1.05)<br>1.01 (0.75-1.38)<br>0.90 (0.66-1.23) | Age, ethnicity, BMI, smoking status, cigarettes per day, pack-years smoked, years since quitting smoking, alcohol, physical activity, family history of diabetes, cancer and heart disease, multivitamin use, aspirin use, hypertension, high cholesterol, diabetes, total energy, healthy eating index (including fruits, vegetables, nuts and legumes, red or processed meat, sugar-sweetened beverages, alcohol, sodium, trans fat, long-chain n-3 FA, other PUFAs, but excluding whole grains), women: postmenopausal status, HRT |

| | | | | | | | | |
|---|---|---|---|---|---|---|---|---|
| Huang T et al, 2015, USA | NIH-AARP Diet and Health Study | 1995-1996 – 2009, 14 years follow-up | 367442 men and women, age 50-71 years: 922 infectious disease deaths | Validated FFQ, 124 food items | Whole grains | 0.13 oz/1000 kcal/d[1]<br>0.30<br>0.47<br>0.69<br>1.20 | 1.00<br>0.84 (0.70-1.02)<br>0.78 (0.64-0.96)<br>0.79 (0.65-0.97)<br>0.77 (0.62-0.95) | Age, sex, number of cigarettes per day, time of smoking cessation, race/ ethnicity, alcohol, education, marital status, health status, BMI, physical activity, red meat, total fruit and vegetables, total energy, HRT (women) |
| Xu M et al, 2015, USA | NIH-AARP Diet and Health Study | 1995-1996 – 2008, 14 years follow-up | 367442 men and women, age 50-71 years: 922 infectious disease deaths | Validated FFQ, 124 food items | Ready-to-eat cereals | 0.00 g/d<br>0.67<br>3.48<br>9.33<br>22.48 | 1.00<br>0.97 (0.79-1.20)<br>0.85 (0.68-1.06)<br>0.84 (0.68-1.05)<br>0.97 (0.78-1.21) | Age, sex, smoking status, smoking dose, time since quitting smoking, race/ethnicity, education, marital status, self-rated health status, BMI, physical activity, menopausal hormone therapy, alcohol, red meat, fruits, vegetables, total energy |

BMI; body mass index, FA; fatty acids, FFQ; food frequency questionnaire, HRT; hormone replacement therapy, PUFA; polyunsaturated fatty acids

[1] The original paper reports in ounces/d, but after contact with the authors it was confirmed that ounces/1000 kcal/d is correct.

## Table S10. Whole grains and refined grains and diabetes mortality

| Author, publication year, country | Study name | Study period | Number of participants, gender, age, number of cases/deaths | Dietary assessment | Exposure and subgroup | Whole grain consumption frequency or amount | Relative risks (95% confidence intervals) | Adjustment for confounding factors |
|---|---|---|---|---|---|---|---|---|
| Jacobs DR, 2007, USA | Iowa Women's Health Study | 1986 – 2003, 17 years follow-up | 27312 women, age 55-69 years: 60 deaths due to endocrine, nutritional and metabolic disorders (mainly diabetes) | Validated FFQ, 127 food items | Whole grains | 1.8 serv/wk<br>5.6<br>8.8<br>14.5<br>25.6 | 1.00<br>0.44 (0.19-1.02)<br>0.65 (0.31-1.37)<br>0.46 (0.20-1.08)<br>0.55 (0.24-1.28) | Age, energy intake, BMI, waist-to-hip ratio, smoking, education, physical activity, HRT, multivitamin supplement use, intake of alcohol, coffee, red meat, fish and seafood, total fruit and vegetables, mutual adjustment between whole grains and refined grains |
| Wu H et al, 2015, USA | Nurses' Health Study & Health Professionals Follow-up Study | 1984-2010, 26 years follow-up 1986-2010, 24 years follow-up | 74341 women, age 38-63 years and 43744 men, age 32-87 years: 283 diabetes deaths | Validated FFQ | Whole grains | 4.2/5.9 g/d (w/m)<br>9.7/14.4<br>14.7/22.1<br>21.1/31.1<br>33.0/47.8 | 1.00<br>0.59 (0.42-0.83)<br>0.52 (0.35-0.77)<br>0.52 (0.34-0.79)<br>0.50 (0.34-0.75) | Age, ethnicity, BMI, smoking status, cigarettes per day, pack-years smoked, years since quitting smoking, alcohol, physical activity, family history of diabetes, cancer and heart disease, multivitamin use, aspirin use, hypertension, high cholesterol, diabetes, total energy, healthy eating index (including fruits, vegetables, nuts and legumes, red or processed meat, sugar-sweetened beverages, alcohol, sodium, trans fat, long-chain n-3 FA, other PUFAs, but excluding whole grains), women: postmenopausal status, HRT |

| | | | | | | | | |
|---|---|---|---|---|---|---|---|---|
| Huang T et al, 2015, USA | NIH-AARP Diet and Health Study | 1995-1996 – 2009, 14 years follow-up | 367442 men and women, age 50-71 years: 371 diabetes deaths | Validated FFQ, 124 food items | Whole grains | 0.13 oz/1000 kcal/d[1]<br>0.30<br>0.47<br>0.69<br>1.20 | 1.00<br>0.71 (0.53-0.96)<br>0.76 (0.56-1.03)<br>0.72 (0.53-0.99)<br>0.52 (0.37-0.75) | Age, sex, number of cigarettes per day, time of smoking cessation, race/ ethnicity, alcohol, education, marital status, health status, BMI, physical activity, red meat, total fruit and vegetables, total energy, HRT (women) |
| Xu M et al, 2015, USA | NIH-AARP Diet and Health Study | 1995-1996 – 2008, 14 years follow-up | 367442 men and women, age 50-71 years: 371 diabetes deaths | Validated FFQ, 124 food items | Ready-to-eat cereals | 0.00 g/d<br>0.67<br>3.48<br>9.33<br>22.48 | 1.00<br>0.96 (0.68-1.36)<br>1.22 (0.87-1.69)<br>1.14 (0.81-1.61)<br>0.70 (0.47-1.03) | Age, sex, smoking status, smoking dose, time since quitting smoking, race/ethnicity, education, marital status, self-rated health status, BMI, physical activity, menopausal hormone therapy, alcohol, red meat, fruits, vegetables, total energy |

| | | | | | | | | |
|---|---|---|---|---|---|---|---|---|
| Johnsen NF et al, 2015, Norway, Sweden, Denmark | HELGA Cohort (Norwegian Women and Cancer Study, Northern Sweden Health and Disease Study, Danish Diet, Cancer and Health Study – part of the EPIC study) | 1992-1998 – 2008-2009, 11.1 (Norway), 14.2 (Sweden), 11.9 years (Denmark) follow-up | 120010 men and women, age 30-64 years: 70/24 diabetes deaths | Validated FFQ, 88 food items (Norway), 98 food items (Sweden) 173 food items (Denmark) | Whole grain breakfast cereals, women | 0 g/d<br>0.8<br>12<br>50 | 1.00<br>0.80 (0.23-2.77)<br>0.45 (0.13-1.59)<br>0.28 (0.07-1.04) | Age, follow-up time, education, smoking status/years since quit/ cigarettes per day, alcohol, BMI, total energy |
| | | | | | Non-white bread | 25 g/d<br>80<br>113<br>180 | 1.00<br>0.47 (0.15-1.48)<br>0.40 (0.11-1.50)<br>0.55 (0.17-1.73) | |
| | | | | | Crisp bread | 0.6 g/d<br>2<br>6<br>31 | 1.00<br>1.21 (0.41-3.54)<br>1.25 (0.36-4.35)<br>1.09 (0.27-4.43) | |
| | | | | | Total whole grain products | 56 g/d<br>100<br>131<br>201 | 1.00<br>0.41 (0.13-1.29)<br>0.41 (0.13-1.29)<br>0.90 (0.31-2.62) | |
| | | | | | Oat | 0 g/d<br>0.4<br>4<br>19 | 1.00<br>0.88 (0.34-2.27)<br>0.36 (0.09-1.51)<br>0.25 (0.05-1.31) | |
| | | | | | Rye | 8 g/d<br>18<br>22<br>41 | 1.00<br>0.93 (0.28-3.09)<br>0.46 (0.14-1.53)<br>0.70 (0.22-2.25) | |
| | | | | | Wheat | 0.4 g/d<br>3<br>10<br>37 | 1.00<br>0.69 (0.26-1.85)<br>0.61 (0.20-1.88)<br>3.17 (0.27-37.59) | |
| | | | | | Total whole grain types | 20 g/d<br>33<br>49<br>74 | 1.00<br>0.77 (0.25-2.41)<br>0.44 (0.14-1.38)<br>0.64 (0.20-2.04) | |
| | | | | | Whole grain breakfast cereals, men | 0 g/d<br>0.8<br>7<br>50 | 1.00<br>0.59 (0.24-1.50)<br>0.31 (0.14-0.68)<br>0.45 (0.23-0.90) | |

| | | | | | | | | |
|---|---|---|---|---|---|---|---|---|
| | | | | | Non-white bread | 13 g/d<br>66<br>118<br>201 | 1.00<br>1.89 (0.66-5.41)<br>1.59 (0.52-4.82)<br>2.30 (0.79-6.72) | |
| | | | | | Crisp bread | 1 g/d<br>2<br>4<br>34 | 1.00<br>1.33 (0.68-2.61)<br>1.07 (0.53-2.15)<br>2.35 (1.10-5.03) | |
| | | | | | Total whole grain products | 64 g/d<br>107<br>156<br>222 | 1.00<br>1.76 (0.83-3.74)<br>1.09 (0.49-2.42)<br>1.67 (0.79-.3.51) | |
| | | | | | Oat | 0 g/d<br>0.4<br>3<br>30 | 1.00<br>0.60 (0.32-1.12)<br>0.33 (0.16-0.72)<br>0.40 (0.20-0.81) | |
| | | | | | Rye | 7 g/d<br>21<br>38<br>56 | 1.00<br>1.14 (0.53-2.48)<br>0.40 (0.12-1.34)<br>1.80 (0.80-4.05) | |
| | | | | | Wheat | 0.1 g/d<br>1<br>5<br>10 | 1.00<br>0.75 (0.37-1.56)<br>1.07 (0.51-2.22)<br>1.18 (0.60-2.34) | |
| | | | | | Total whole grain types | 21 g/d<br>37<br>54<br>80 | 1.00<br>0.92 (0.45-1.87)<br>0.95 (0.49-1.86)<br>1.18 (0.59-2.38) | |

BMI; body mass index, FA; fatty acids, FFQ; food frequency questionnaire, HRT; hormone replacement therapy, PUFA; polyunsaturated fatty acids

[1] The original paper reports in ounces/d, but after contact with the authors it was confirmed that ounces/1000 kcal/d is correct.

**Table S11. Whole grains and refined grains and nervous system disease mortality**

| Author, publication year, country | Study name | Study period | Number of participants, gender, age, number of cases/deaths | Dietary assessment | Exposure and subgroup | Whole grain consumption frequency or amount | Relative risks (95% confidence intervals) | Adjustment for confounding factors |
|---|---|---|---|---|---|---|---|---|
| Jacobs DR, 2007, USA | Iowa Women's Health Study | 1986 – 2003, 17 years follow-up | 27312 women, age 55-69 years: 241 nervous system deaths | Validated FFQ, 127 food items | Whole grains | 1.8 serv/wk<br>5.6<br>8.8<br>14.5<br>25.6 | 1.00<br>0.84 (0.53-1.32)<br>1.33 (0.88-1.99)<br>0.83 (0.53-1.31)<br>0.89 (0.55-1.42) | Age, energy intake, BMI, waist-to-hip ratio, smoking, education, physical activity, HRT, multivitamin supplement use, intake of alcohol, coffee, red meat, fish and seafood, total fruit and vegetables, mutual adjustment between whole grains and refined grains |
| Wu H et al, 2015, USA | Nurses' Health Study & Health Professionals Follow-up Study | 1984-2010, 26 years follow-up 1986-2010, 24 years follow-up | 74341 women, age 38-63 years and 43744 men, age 32-87 years: 2044 neurodegenerative disease deaths | Validated FFQ | Whole grains | 4.2/5.9 g/d (w/m)<br>9.7/14.4<br>14.7/22.1<br>21.1/31.1<br>33.0/47.8 | 1.00<br>1.24 (1.08-1.42)<br>1.25 (1.09-1.44)<br>1.35 (1.17-1.55)<br>1.20 (1.04-1.38) | Age, ethnicity, BMI, smoking status, cigarettes per day, pack-years smoked, years since quitting smoking, alcohol, physical activity, family history of diabetes, cancer and heart disease, multivitamin use, aspirin use, hypertension, high cholesterol, diabetes, total energy, healthy eating index (including fruits, vegetables, nuts and legumes, red or processed meat, sugar-sweetened beverages, alcohol, sodium, trans fat, long-chain n-3 FA, other PUFAs, but excluding whole grains), women: postmenopausal status, HRT |

**Table S12. Whole grains and refined grains and non-cardiovascular, non-cancer causes of death**

| Author, publication year, country | Study name | Study period | Number of participants, gender, age, number of cases/deaths | Dietary assessment | Exposure and subgroup | Whole grain consumption frequency or amount | Relative risks (95% confidence intervals) | Adjustment for confounding factors |
|---|---|---|---|---|---|---|---|---|
| Jacobs DR, 2007, USA | Iowa Women's Health Study | 1986 – 2003, 17 years follow-up | 27312 women, age 55-69 years: 1072 non-CVD, non-cancer, inflammatory disease deaths<br>482 non-CVD, non-cancer, non-inflammatory disease deaths | Validated FFQ, 127 food items | Whole grains, non-CVD, non-cancer, inflammatory diseases<br><br><br><br><br>Whole grains, non-CVD, non-cancer, non-inflammatory diseases | 1.8 serv/wk<br>5.6<br>8.8<br>14.5<br>25.6<br>1.8 serv/wk<br>5.6<br>8.8<br>14.5<br>25.6 | 1.00<br>0.69 (0.57-0.83)<br>0.79 (0.66-0.95)<br>0.64 (0.53-0.79)<br>0.66 (0.54-0.81)<br>1.00<br>1.25 (0.94-1.67)<br>1.12 (0.83-1.51)<br>1.02 (0.74-1.39)<br>1.02 (0.74-1.42) | Age, energy intake, BMI, waist-to-hip ratio, smoking, education, physical activity, HRT, multivitamin supplement use, intake of alcohol, coffee, red meat, fish and seafood, total fruit and vegetables, mutual adjustment between whole grains and refined grains |
| Buil-Cosiales P et al, 2014, Spain | Prevencion con DietaMediterranea (PREDIMED) study | 2003-2009 – 2012, 5.9 years follow-up | 7216 men and women, age 55-75 years: 153 non-CVD, non-cancer deaths | Validated FFQ, 137 food items | Whole grains | 0 g/d<br>1<br>7<br>33<br>89 | 1.00<br>0.89 (0.49-1.63)<br>1.03 (0.52-2.02)<br>1.03 (0.51-2.10)<br>0.76 (0.38-1.53) | Age, sex, smoking status, diabetes, BMI, SBP, DBP, recruitment center, statins, alcohol, education, physical activity, total energy, vegetables, fruits |

| | | | | | | | | |
|---|---|---|---|---|---|---|---|---|
| Wu H et al, 2015, USA | Nurses' Health Study & Health Professionals Follow-up Study | 1984-2010, 26 years follow-up 1986-2010, 24 years follow-up | 74341 women, age 38-63 years and 43744 men, age 32-87 years: 10425 non-CVD, non-cancer deaths[1] | Validated FFQ | Whole grains | 4.2/5.9 g/d (w/m)<br>9.7/14.4<br>14.7/22.1<br>21.1/31.1<br>33.0/47.8 | 1.00<br>0.98 (0.93-1.04)<br>0.95 (0.89-1.01)<br>0.97 (0.91-1.03)<br>0.91 (0.86-0.97) [1] | Age, ethnicity, BMI, smoking status, cigarettes per day, pack-years smoked, years since quitting smoking, alcohol, physical activity, family history of diabetes, cancer and heart disease, multivitamin use, aspirin use, hypertension, high cholesterol, diabetes, total energy, healthy eating index (including fruits, vegetables, nuts and legumes, red or processed meat, sugar-sweetened beverages, alcohol, sodium, trans fat, long-chain n-3 FA, other PUFAs, but excluding whole grains), women: postmenopausal status, HRT |
| Huang T et al, 2015, USA | NIH-AARP Diet and Health Study | 1995-1996 – 2009, 14 years follow-up | 367442 men and women, age 50-71 years: 10312 non-CVD, non-cancer deaths[1] | Validated FFQ, 124 food items | Whole grains | 0.13 oz/1000 kcal/d[2]<br>0.30<br>0.47<br>0.69<br>1.20 | 1.00<br>0.97 (0.88-1.06)<br>0.97 (0.89-1.06)<br>0.87 (0.79-0.96)<br>0.86 (0.78-0.94) [1] | Age, sex, number of cigarettes per day, time of smoking cessation, race/ ethnicity, alcohol, education, marital status, health status, BMI, physical activity, red meat, total fruit and vegetables, total energy, HRT (women) |

BMI; body mass index, DBP; diastolic blood pressure, FA; fatty acids, FFQ; food frequency questionnaire, HRT; hormone replacement therapy, PUFA; polyunsaturated fatty acids, SBP; systolic blood pressure

[1] For the Nurses' Health Study, Health Professionals Follow-up Study, and NIH-AARP Diet and Health Study data were pooled for all non-CVD, non-cancer causes of death (NHS&HPFS: deaths from respiratory disease, neurodegenerative disease, infectious disease, kidney disease, diabetes, and other causes, and NIH-AARP Diet and Health Study: deaths from diabetes, respiratory disease, infections and other/unknown causes) using a fixed effects model.

[2] The original paper reports in ounces/d, but after contact with the authors it was confirmed that ounces/1000 kcal/d is correct.

**Table S13. Relative risks from nonlinear dose-response analysis of whole grains and coronary heart disease, stroke, cardiovascular disease, total cancer, and all-cause mortality**

| Coronary heart disease | | Stroke | | Cardiovascular disease | | Total cancer | | All-cause mortality | |
|---|---|---|---|---|---|---|---|---|---|
| **g/d** | **RR (95% CI)** | **g/d** | **RR (95% CI)** | **g/d** | **RR (95% CI)** | **g/d** | **RR (95% CI)** | **g/d** | **RR (95% CI)** |
| 0 | 1.00 | 0 | 1.00 | 0 | 1.00 | 0 | 1.00 | 0 | 1.00 |
| 15 | 0.93 (0.92-0.95) | 15 | 0.94 (0.92-0.95) | 15 | 0.93 (0.91-0.94) | 15 | 0.97 (0.96-0.98) | 15 | 0.95 (0.94-0.96) |
| 30 | 0.87 (0.85-0.90) | 30 | 0.88 (0.85-0.91) | 30 | 0.87 (0.85-0.89) | 30 | 0.95 (0.93-0.97) | 30 | 0.90 (0.89-0.92) |
| 45 | 0.82 (0.79-0.85) | 45 | 0.83 (0.78-0.87) | 45 | 0.83 (0.81-0.86) | 45 | 0.93 (0.90-0.95) | 45 | 0.87 (0.85-0.89) |
| 60 | 0.78 (0.75-0.82) | 60 | 0.78 (0.73-0.84) | 60 | 0.81 (0.79-0.84) | 60 | 0.91 (0.88-0.94) | 60 | 0.85 (0.82-0.87) |
| 75 | 0.75 (0.71-0.79) | 75 | 0.75 (0.69-0.81) | 75 | 0.80 (0.77-0.82) | 75 | 0.89 (0.86-0.92) | 75 | 0.83 (0.80-0.85) |
| 90 | 0.72 (0.69-0.76) | 90 | 0.73 (0.66-0.79) | 90 | 0.79 (0.76-0.82) | 90 | 0.87 (0.84-0.91) | 90 | 0.81 (0.79-0.83) |
| 105 | 0.70 (0.66-0.74) | 105 | 0.71 (0.64-0.78) | 105 | 0.78 (0.75-0.82) | 105 | 0.86 (0.83-0.90) | 105 | 0.80 (0.78-0.82) |
| 120 | 0.69 (0.65-0.73) | 120 | 0.70 (0.63-0.77) | 120 | 0.78 (0.74-0.82) | 120 | 0.85 (0.81-0.89) | 120 | 0.78 (0.76-0.81) |
| 135 | 0.67 (0.63-0.71) | 135 | 0.69 (0.62-0.77) | 135 | 0.77 (0.72-0.82) | 135 | 0.83 (0.80-0.87) | 135 | 0.77 (0.75-0.80) |
| 150 | 0.66 (0.62-0.70) | 150 | 0.69 (0.62-0.77) | 150 | 0.76 (0.71-0.82) | 150 | 0.82 (0.78-0.86) | 150 | 0.76 (0.73-0.79) |
| 165 | 0.65 (0.61-0.69) | 165 | 0.69 (0.62-0.77) | 165 | 0.75 (0.70-0.82) | 165 | 0.81 (0.77-0.86) | 165 | 0.75 (0.71-0.78) |
| 180 | 0.64 (0.59-0.68) | 180 | 0.69 (0.62-0.77) | 180 | 0.75 (0.68-0.82) | 180 | 0.80 (0.75-0.85) | 180 | 0.73 (0.70-0.77) |
| 195 | 0.63 (0.58-0.68) | 195 | 0.69 (0.62-0.77) | 195 | 0.74 (0.67-0.82) | 195 | 0.78 (0.73-0.84) | 195 | 0.72 (0.68-0.76) |
| 210 | 0.62 (0.57-0.67) | 210 | 0.70 (0.62-0.78) | 210 | 0.73 (0.66-0.82) | 210 | 0.77 (0.72-0.83) | 210 | 0.71 (0.67-0.75) |
| 225 | 0.61 (0.56-0.66) | 225 | 0.70 (0.63-0.78) | 225 | | 225 | 0.76 (0.71-0.82) | 225 | 0.70 (0.65-0.75) |
| 240 | | 240 | 0.70 (0.63-0.79) | 240 | | 240 | | 240 | |
| $P_{nonlinearity}$ | <0.0001 | | <0.0001 | | <0.0001 | | 0.15 | | <0.0001 |

**Table S14. Relative risks from nonlinear dose-response analysis of whole grains and**

| Respiratory disease mortality | | Diabetes mortality | | Infectious disease mortality | | Nervous system disease mortality | | All noncardiovascular, noncancer causes of death | |
|---|---|---|---|---|---|---|---|---|---|
| **g/d** | **RR (95% CI)** | **g/d** | **RR (95% CI)** | **g/d** | **RR (95% CI)** | **g/d** | **RR (95% CI)** | **g/d** | **RR (95% CI)** |
| 4.8 | 1.00 | 4.8 | 1.00 | 4.8 | 1.00 | 4.8 | 1.00 | 0 | 1.00 |
| 15 | 0.93 (0.91-0.96) | 15 | 0.80 (0.77-0.83) | 15 | 0.89 (0.85-0.93) | 15 | 1.14 (1.11-1.18) | 15 | 0.96 (0.95-0.97) |
| 30 | 0.88 (0.84-0.92) | 30 | 0.65 (0.61-0.70) | 30 | 0.81 (0.75-0.88) | 30 | 1.26 (1.19-1.33) | 30 | 0.92 (0.90-0.94) |
| 45 | 0.83 (0.78-0.88) | 45 | 0.55 (0.50-0.60) | 45 | 0.78 (0.72-0.85) | 45 | 1.28 (1.20-1.37) | 45 | 0.88 (0.86-0.91) |
| 60 | 0.79 (0.73-0.85) | 60 | 0.48 (0.43-0.54) | 60 | 0.77 (0.71-0.84) | 60 | 1.23 (1.15-1.33) | 60 | 0.85 (0.83-0.88) |
| 75 | 0.76 (0.69-0.82) | 75 | 0.44 (0.39-0.50) | 75 | 0.76 (0.70-0.84) | 75 | 1.14 (1.05-1.25) | 75 | 0.82 (0.79-0.85) |
| 90 | 0.73 (0.67-0.80) | 90 | 0.42 (0.37-0.48) | 90 | 0.76 (0.68-0.85) | 90 | 1.05 (0.93-1.17) | 90 | 0.79 (0.77-0.82) |
| 105 | 0.71 (0.65-0.78) | 105 | 0.41 (0.35-0.48) | 105 | 0.76 (0.66-0.87) | 105 | 0.95 (0.83-1.10) | 105 | 0.77 (0.74-0.80) |
| 120 | 0.70 (0.63-0.77) | 120 | 0.41 (0.35-0.48) | 120 | | 120 | | 120 | 0.75 (0.72-0.78) |
| 135 | 0.68 (0.61-0.76) | 135 | 0.41 (0.35-0.49) | 135 | | 135 | | 135 | 0.72 (0.69-0.75) |
| 150 | 0.67 (0.60-0.75) | 150 | 0.42 (0.35-0.50) | 150 | | 150 | | 150 | 0.70 (0.67-0.73) |
| 165 | 0.66 (0.59-0.74) | 165 | 0.43 (0.36-0.52) | 165 | | 165 | | 165 | 0.68 (0.65-0.71) |
| 180 | 0.65 (0.57-0.73) | 180 | 0.44 (0.36-0.54) | 180 | | 180 | | 180 | 0.66 (0.62-0.69) |
| 195 | 0.63 (0.56-0.72) | 195 | 0.45 (0.36-0.56) | 195 | | 195 | | 195 | 0.64 (0.60-0.67) |
| 210 | 0.62 (0.54-0.72) | 210 | 0.46 (0.37-0.58) | 210 | | 210 | | 210 | 0.62 (0.58-0.66) |
| 225 | 0.62 (0.53-0.71) | 225 | 0.47 (0.37-0.59) | 225 | | 225 | | 225 | 0.60 (0.57-0.64) |
| 240 | | 240 | | 240 | | 240 | | 240 | |
| $P_{nonlinearity}$ | 0.001 | | <0.0001 | | 0.003 | | <0.0001 | | 0.06 |

## Table S15: Subgroup analyses of whole grains and coronary heart disease, stroke, and cardiovascular disease, per 3 servings per day

| | Coronary heart disease | | | | | Stroke | | | | | Cardiovascular disease | | | | |
|---|---|---|---|---|---|---|---|---|---|---|---|---|---|---|---|
| | *n* | RR (95% CI) | $I^2$ | $P_h^1$ | $P_h^2$ | *n* | RR (95% CI) | $I^2$ | $P_h^1$ | $P_h^2$ | *n* | RR (95% CI) | $I^2$ | $P_h^1$ | $P_h^2$ |
| All studies | 7 | 0.81 (0.75-0.87) | 8.9 | 0.36 | | 6 | 0.88 (0.75-1.03) | 56.3 | 0.04 | | 10 | 0.78 (0.73-0.85) | 40.0 | 0.09 | |
| Duration of follow-up | | | | | | | | | | | | | | | |
| <10 yrs follow-up | 1 | 0.90 (0.78-1.04) | | | 0.24 | 0 | | | | NC | 1 | 0.83 (0.40-1.71) | | | 0.90 |
| ≥10 yrs follow-up | 6 | 0.80 (0.74-0.86) | 0 | 0.47 | | 6 | 0.88 (0.75-1.03) | 56.3 | 0.04 | | 9 | 0.78 (0.73-0.85) | 46.6 | 0.06 | |
| Outcome | | | | | | | | | | | | | | | |
| Incidence | 5 | 0.84 (0.77-0.92) | 33.8 | 0.20 | 0.61 | 3 | 0.84 (0.59-1.20) | 74.2 | 0.02 | 0.81 | 2 | 0.87 (0.78-0.97) | 0 | 0.85 | 0.15 |
| Mortality | 3 | 0.81 (0.74-0.89) | 9.8 | 0.33 | | 3 | 0.86 (0.74-0.99) | 0 | 0.90 | | 8 | 0.71 (0.61-0.82) | 72.1 | 0.001 | |
| Outcome subtype | | | | | | | | | | | | | | | |
| Myocardial infarction | 2 | 0.92 (0.87-0.97) | 0 | 0.69 | 0.05 | - | | | | - | - | | | | - |
| Coronary heart disease | 5 | 0.78 (0.72-0.86) | 12.3 | 0.34 | | - | | | | | - | | | | |
| Ischemic stroke | - | | | | | 4 | 0.89 (0.71-1.11) | 50.8 | 0.11 | 0.38 | - | | | | - |
| Hemorrhagic stroke | - | | | | | 2 | 1.11 (0.82-1.50) | 0 | 0.86 | | - | | | | |
| Gender | | | | | | | | | | | | | | | |
| Men | 2 | 0.80 (0.60-1.08) | 66.1 | 0.09 | 0.60/ | 1 | 0.84 (0.65-1.07) | | | 0.46/ | 2 | 0.82 (0.67-1.00) | 65.1 | 0.09 | 0.67/ |
| Women | 4 | 0.76 (0.66-0.88) | 51.8 | 0.10 | 0.58 | 3 | 0.84 (0.70-1.00) | 0 | 0.50 | NC | 4 | 0.75 (0.65-0.86) | 37.6 | 0.19 | 0.44 |
| Men and women | 2 | 0.78 (0.62-0.98) | 0 | 0.68 | | 3 | 0.90 (0.68-1.19) | 63.2 | 0.07 | | 5 | 0.79 (0.70-0.88) | 36.1 | 0.18 | |
| Geographic location | | | | | | | | | | | | | | | |
| Europe | 2 | 0.86 (0.79-0.93) | 0 | 0.51 | 0.11 | 2 | 0.98 (0.78-1.22) | 76.2 | 0.04 | 0.24 | 3 | 0.85 (0.79-0.91) | 0 | 0.97 | 0.06 |
| America | 4 | 0.72 (0.63-0.81) | 0 | 0.95 | | 3 | 0.79 (0.64-0.97) | 0 | 0.47 | | 6 | 0.74 (0.67-0.81) | 24.9 | 0.25 | |
| Asia | 1 | 0.83 (0.57-1.23) | | | | 1 | 0.80 (0.57-1.13) | | | | 1 | 0.82 (0.63-1.05) | | | |
| Australia | 0 | | | | | 0 | | | | | 0 | | | | |
| Number of cases | | | | | | | | | | | | | | | |
| Cases <500 | 1 | 0.83 (0.57-1.23) | | | 0.61 | 5 | 0.82 (0.72-0.93) | 0 | 0.76 | 0.04 | 2 | 0.62 (0.34-1.13) | 25.3 | 0.25 | 0.66 |
| Cases 500-<1000 | 2 | 0.73 (0.61-0.89) | 0 | 0.85 | | 1 | 1.08 (0.96-1.22) | | | | 1 | 0.82 (0.63-1.05) | | | |
| Cases ≥1000 | 4 | 0.81 (0.73-0.90) | 42.7 | 0.16 | | 0 | | | | | 7 | 0.79 (0.72-0.85) | 52.9 | 0.05 | |
| Validated dietary assessment | | | | | | | | | | | | | | | |
| Yes | 6 | 0.80 (0.74-0.87) | 24.0 | 0.25 | 0.86 | 4 | 0.83 (0.72-0.95) | 0 | 0.61 | 0.11 | 8 | 0.79 (0.73-0.85) | 45.1 | 0.08 | 0.81 |
| No | 1 | 0.83 (0.57-1.23) | | | | 2 | 0.98 (0.74-1.29) | 62.1 | 0.10 | | 2 | 0.67 (0.39-1.17) | 54.1 | 0.14 | |
| Study quality | | | | | | | | | | | | | | | |
| 0-3 stars | 0 | | | | 0.86 | 0 | | | | 0.71 | 0 | | | | 0.81 |
| 4-6 | 1 | 0.83 (0.57-1.23) | | | | 1 | 0.80 (0.57-1.12) | | | | 1 | 0.82 (0.63-1.05) | | | |
| 7-9 | 6 | 0.80 (0.74-0.87) | 24.0 | 0.25 | | 5 | 0.88 (0.74-1.05) | 61.6 | 0.03 | | 9 | 0.78 (0.72-0.85) | 46.5 | 0.06 | |
| Adjustment for confounding factors | | | | | | | | | | | | | | | |

| | | | | | | | | | | | | | | | | |
|---|---|---|---|---|---|---|---|---|---|---|---|---|---|---|---|---|
| Age | Yes | 7 | 0.81 (0.75-0.87) | 8.9 | 0.36 | NC | 6 | 0.88 (0.75-1.03) | 56.3 | 0.04 | NC | 10 | 0.78 (0.73-0.85) | 40.0 | 0.09 | NC |
| | No | 0 | | | | | 0 | | | | | 0 | | | | |
| Education | Yes | 4 | 0.83 (0.76-0.90) | 22.0 | 0.28 | 0.23 | 3 | 0.85 (0.73-0.99) | 0 | 0.66 | 0.64 | 7 | 0.80 (0.74-0.87) | 40.7 | 0.12 | 0.25 |
| | No | 3 | 0.73 (0.61-0.87) | 0 | 0.66 | | 3 | 0.88 (0.66-1.18) | 71.7 | 0.03 | | 3 | 0.71 (0.59-0.86) | 32.6 | 0.23 | |
| Family history of CHD | Yes | 3 | 0.78 (0.65-0.95) | 50.3 | 0.13 | 0.95 | 1 | 0.69 (0.48-1.00) | | | 0.31 | 2 | 0.66 (0.52-0.84) | 33.3 | 0.22 | 0.13 |
| | No | 4 | 0.81 (0.75-0.88) | 0 | 0.47 | | 5 | 0.91 (0.78-1.06) | 52.3 | 0.08 | | 8 | 0.80 (0.75-0.86) | 31.0 | 0.18 | |
| Body mass index | Yes | 7 | 0.81 (0.75-0.87) | 8.9 | 0.36 | NC | 6 | 0.88 (0.75-1.03) | 56.3 | 0.04 | NC | 9 | 0.78 (0.72-0.84) | 43.1 | 0.08 | 0.39 |
| | No | 0 | | | | | 0 | | | | | 1 | 0.89 (0.71-1.11) | | | |
| Smoking | Yes | 7 | 0.81 (0.75-0.87) | 8.9 | 0.36 | NC | 6 | 0.88 (0.75-1.03) | 56.3 | 0.04 | NC | 10 | 0.78 (0.73-0.85) | 40.0 | 0.09 | NC |
| | No | 0 | | | | | 0 | | | | | 0 | | | | |
| Alcohol | Yes | 7 | 0.81 (0.75-0.87) | 8.9 | 0.36 | NC | 5 | 0.82 (0.72-0.93) | 0 | 0.76 | 0.04 | 10 | 0.78 (0.73-0.85) | 40.0 | 0.09 | NC |
| | No | 0 | | | | | 1 | 1.08 (0.96-1.22) | | | | 0 | | | | |
| Physical activity | Yes | 5 | 0.77 (0.69-0.87) | 28.0 | 0.24 | 0.43 | 4 | 0.88 (0.69-1.11) | 64.5 | 0.04 | 0.72 | 8 | 0.76 (0.70-0.84) | 37.7 | 0.13 | 0.26 |
| | No | 2 | 0.84 (0.77-0.93) | 0 | 0.93 | | 2 | 0.84 (0.72-1.00) | 0 | 0.75 | | 2 | 0.84 (0.78-0.91) | 0 | 0.78 | |
| Hypertension | Yes | 3 | 0.82 (0.71-0.95) | 24.2 | 0.27 | 0.70 | 2 | 0.69 (0.52-0.93) | 0 | 0.99 | 0.17 | 3 | 0.64 (0.51-0.80) | 21.9 | 0.28 | 0.08 |
| | No | 4 | 0.79 (0.71-0.88) | 20.2 | 0.29 | | 4 | 0.94 (0.80-1.09) | 52.8 | 0.10 | | 7 | 0.81 (0.76-0.86) | 23.5 | 0.25 | |
| Hypercholesterolemia, serum cholesterol | Yes | 2 | 0.83 (0.67-1.01) | 51.3 | 0.15 | 0.54 | 2 | 0.90 (0.58-1.38) | 80.4 | 0.02 | 0.35 | 2 | 0.66 (0.52-0.84) | 33.3 | 0.22 | 0.13 |
| | No | 5 | 0.80 (0.74-0.87) | 0 | 0.41 | | 4 | 0.84 (0.73-0.96) | 0 | 0.82 | | 8 | 0.80 (0.75-0.86) | 31.0 | 0.18 | |
| Coffee, caffeine | Yes | 1 | 0.72 (0.60-0.87) | | | 0.22 | 1 | 0.89 (0.67-1.19) | | | 0.90 | 1 | 0.75 (0.63-0.89) | | | 0.68 |
| | No | 6 | 0.83 (0.77-0.89) | 0 | 0.46 | | 5 | 0.86 (0.71-1.04) | 64.4 | 0.02 | | 9 | 0.79 (0.73-0.86) | 45.0 | 0.07 | |
| Sugar-sweetened beverages | Yes | 0 | | | | NC | 0 | | | | NC | 2 | 0.66 (0.52-0.84) | 33.3 | 0.22 | 0.13 |
| | No | 7 | 0.81 (0.75-0.87) | 8.9 | 0.36 | | 6 | 0.88 (0.75-1.03) | 56.3 | 0.04 | | 8 | 0.80 (0.75-0.86) | 31.0 | 0.18 | |
| Red or processed meat | Yes | 1 | 0.72 (0.60-0.87) | | | 0.22 | 1 | 0.89 (0.67-1.19) | | | 0.90 | 4 | 0.73 (0.68-0.78) | 0 | 0.49 | 0.02 |
| | No | 6 | 0.83 (0.77-0.89) | 0 | 0.46 | | 5 | 0.86 (0.71-1.04) | 64.4 | 0.02 | | 6 | 0.85 (0.80-0.90) | 0 | 0.69 | |
| Fish | Yes | 2 | 0.71 (0.60-0.83) | 0 | 0.62 | 0.11 | 1 | 0.89 (0.67-1.19) | | | 0.90 | 1 | 0.75 (0.63-0.89) | | | 0.68 |
| | No | 5 | 0.84 (0.78-0.90) | 0 | 0.62 | | 5 | 0.86 (0.71-1.04) | 64.4 | 0.02 | | 9 | 0.79 (0.73-0.86) | 45.0 | 0.07 | |
| Fruits and vegetables | Yes | 2 | 0.71 (0.60-0.83) | 0 | 0.62 | 0.11 | 1 | 0.89 (0.67-1.19) | | | 0.90 | 5 | 0.66 (0.57-0.76) | 54.2 | 0.07 | 0.02 |
| | No | 5 | 0.84 (0.78-0.90) | 0 | 0.62 | | 5 | 0.86 (0.71-1.04) | 64.4 | 0.02 | | 5 | 0.85 (0.80-0.91) | 0 | 0.55 | |
| Dairy | Yes | 0 | | | | NC | 0 | | | | NC | 0 | | | | NC |
| | No | 7 | 0.81 (0.75-0.87) | 8.9 | 0.36 | | 5 | 0.86 (0.71-1.04) | 64.4 | 0.02 | | 10 | 0.78 (0.73-0.85) | 40.0 | 0.09 | |
| Energy intake | Yes | 6 | 0.80 (0.74-0.87) | 24.0 | 0.25 | 0.86 | 5 | 0.88 (0.74-1.05) | 61.6 | 0.03 | 0.71 | 9 | 0.78 (0.72-0.85) | 46.5 | 0.06 | 0.81 |
| | No | 1 | 0.83 (0.57-1.23) | | | | 1 | 0.80 (0.57-1.12) | | | | 1 | 0.82 (0.63-1.05) | | | |

*n*denotes the number of studies.
[1] P for heterogeneity within each subgroup,
[2] P for heterogeneity between subgroups with meta-regression analysis,
[3] P for heterogeneity between men and women (studies with genders mixed were excluded),
NC = not calculable

**Table S16: Subgroup analyses of whole grains and total cancer and all-cause mortality, per 3 servings per day**

| | | Total cancer | | | | | All-cause mortality | | | | |
|---|---|---|---|---|---|---|---|---|---|---|---|
| | | *n* | RR (95% CI) | $I^2$ | $P_h$[1] | $P_h$[2] | *n* | RR (95% CI) | $I^2$ | $P_h$[1] | $P_h$[2] |
| All studies | | 6 | 0.85 (0.80-0.91) | 37.0 | 0.16 | | 11 | 0.83 (0.77-0.90) | 82.9 | <0.0001 | |
| Duration of follow-up | | | | | | | | | | | |
| <10 yrs follow-up | | 1 | 0.77 (0.43-1.38) | | | 0.74 | 1 | 0.98 (0.67-1.44) | | | 0.55 |
| ≥10 yrs follow-up | | 5 | 0.86 (0.80-0.92) | 49.0 | 0.10 | | 10 | 0.82 (0.76-0.89) | 84.3 | <0.0001 | |
| Outcome | | | | | | | | | | | |
| Incidence | | 0 | | | | NC | - | | | | NC |
| Mortality | | 6 | 0.87 (0.81-0.93) | 51.2 | 0.07 | | 10 | 0.82 (0.75-0.89) | 83.9 | <0.0001 | |
| Gender | | | | | | | | | | | |
| Men | | 2 | 0.83 (0.77-0.89) | 0 | 0.36 | 0.63/ 0.13 | 3 | 0.93 (0.80-1.08) | 86.2 | 0.001 | 0.39/ 0.27 |
| Women | | 3 | 0.92 (0.86-0.98) | 0 | 0.92 | | 5 | 0.78 (0.68-0.88) | 80.5 | <0.0001 | |
| Men and women | | 2 | 0.79 (0.74-0.84) | 0 | 0.94 | | 5 | 0.81 (0.73-0.91) | 41.1 | 0.15 | |
| Geographic location | | | | | | | | | | | |
| Europe | | 2 | 0.86 (0.81-0.91) | 0 | 0.72 | 0.87 | 3 | 0.94 (0.76-1.16) | 90.7 | <0.0001 | 0.53 |
| America | | 4 | 0.87 (0.78-0.97) | 58.5 | 0.07 | | 7 | 0.77 (0.70-0.85) | 72.6 | 0.001 | |
| Asia | | 0 | | | | | 1 | 0.94 (0.80-1.16) | | | |
| Australia | | 0 | | | | | 0 | | | | |
| Number of cases | | | | | | | | | | | |
| Cases <500 | | 1 | 0.77 (0.43-1.38) | | | 0.74 | 2 | 0.91 (0.67-1.23) | 0 | 0.52 | 0.82 |
| Cases 500-<1000 | | 0 | | | | | 1 | 0.77 (0.61-0.96) | | | |
| Cases ≥1000 | | 5 | 0.86 (0.80-0.92) | 49.0 | 0.10 | | 8 | 0.83 (0.76-0.90) | 87.8 | <0.0001 | |
| Validated dietary assessment | | | | | | | | | | | |
| Yes | | 6 | 0.85 (0.80-0.91) | 37.0 | 0.16 | NC | 9 | 0.82 (0.75-0.89) | 85.7 | <0.0001 | 0.60 |
| No | | 0 | | | | | 2 | 0.92 (0.78-1.10) | 0 | 0.55 | |
| Study quality | | | | | | | | | | | |
| 0-3 stars | | 0 | | | | NC | 0 | | | | 0.51 |
| 4-6 | | 0 | | | | | 1 | 0.94 (0.80-1.16) | | | |
| 7-9 | | 6 | 0.85 (0.80-0.91) | 37.0 | 0.16 | | 10 | 0.82 (0.76-0.89) | 83.9 | <0.0001 | |
| Adjustment for confounding factors | | | | | | | | | | | |
| Age | Yes | 6 | 0.85 (0.80-0.91) | 37.0 | 0.16 | NC | 11 | 0.83 (0.77-0.90) | 82.9 | <0.0001 | NC |
| | No | 0 | | | | | 0 | | | | |
| Education | Yes | 4 | 0.84 (0.78-0.90) | 51.1 | 0.11 | 0.36 | 8 | 0.83 (0.76-0.91) | 84.5 | <0.0001 | 0.97 |
| | No | 2 | 0.93 (0.80-1.08) | 0 | 0.66 | | 3 | 0.83 (0.67-1.03) | 84.4 | 0.002 | |
| Family history of CHD | Yes | 2 | 0.93 (0.80-1.08) | 0 | 0.66 | 0.36 | 2 | 0.78 (0.57-1.06) | 90.6 | 0.001 | 0.67 |

| | | | | | | | | | | | |
|---|---|---|---|---|---|---|---|---|---|---|---|
| | No | 4 | 0.84 (0.78-0.90) | 51.1 | 0.11 | | 9 | 0.84 (0.77-0.92) | 83.3 | <0.0001 | |
| Body mass index | Yes | 6 | 0.85 (0.80-0.91) | 37.0 | 0.16 | NC | 10 | 0.84 (0.78-0.91) | 82.2 | <0.0001 | 0.02 |
| | No | 0 | | | | | 1 | 0.30 (0.15-0.60) | | | |
| Smoking | Yes | 6 | 0.85 (0.80-0.91) | 37.0 | 0.16 | NC | 11 | 0.83 (0.77-0.90) | 82.9 | <0.0001 | NC |
| | No | 0 | | | | | 0 | | | | |
| Alcohol | Yes | 6 | 0.85 (0.80-0.91) | 37.0 | 0.16 | NC | 10 | 0.80 (0.75-0.86) | 71.0 | <0.0001 | 0.07 |
| | No | 0 | | | | | 1 | 1.05 (0.95-1.17) | | | |
| Physical activity | Yes | 5 | 0.86 (0.78-0.95) | 45.2 | 0.12 | 0.95 | 9 | 0.81 (0.72-0.91) | 85.5 | <0.0001 | 0.67 |
| | No | 1 | 0.86 (0.81-0.91) | | | | 2 | 0.85 (0.76-0.96) | 51.9 | 0.15 | |
| Hypertension | Yes | 2 | 0.93 (0.80-1.08) | 0 | 0.66 | 0.36 | 5 | 0.84 (0.69-1.02) | 85.6 | <0.0001 | 0.74 |
| | No | 4 | 0.84 (0.78-0.90) | 51.1 | 0.11 | | 6 | 0.81 (0.75-0.87) | 75.4 | 0.001 | |
| Hypercholesterolemia | Yes | 2 | 0.93 (0.80-1.08) | 0 | 0.66 | 0.36 | 2 | 0.78 (0.57-1.06) | 90.6 | 0.001 | 0.67 |
| | No | 4 | 0.84 (0.78-0.90) | 51.1 | 0.11 | | 9 | 0.84 (0.77-0.92) | 83.3 | <0.0001 | |
| Coffee | Yes | 1 | 0.92 (0.81-1.04) | | | 0.36 | 1 | 0.80 (0.73-0.87) | | | 0.99 |
| | No | 5 | 0.84 (0.79-0.89) | 30.2 | 0.22 | | 10 | 0.83 (0.76-0.91) | 84.6 | <0.0001 | |
| Sugar-sweetened beverages | Yes | 2 | 0.93 (0.80-1.08) | 0 | 0.66 | 0.36 | 3 | 0.67 (0.47-0.95) | 89.0 | <0.0001 | 0.25 |
| | No | 4 | 0.84 (0.78-0.90) | 51.1 | 0.11 | | 8 | 0.85 (0.79-0.93) | 82.4 | <0.0001 | |
| Red or processed meat | Yes | 4 | 0.87 (0.78-0.97) | 58.5 | 0.07 | 0.87 | 5 | 0.77 (0.69-0.86) | 81.7 | <0.0001 | 0.17 |
| | No | 2 | 0.86 (0.81-0.91) | 0 | 0.72 | | 6 | 0.90 (0.78-1.03) | 78.9 | <0.0001 | |
| Fish | Yes | 1 | 0.92 (0.81-1.04) | | | 0.36 | 1 | 0.80 (0.73-0.87) | | | 0.99 |
| | No | 5 | 0.84 (0.79-0.89) | 30.2 | 0.22 | | 10 | 0.83 (0.76-0.91) | 84.6 | <0.0001 | |
| Fruit and vegetables | Yes | 5 | 0.86 (0.78-0.95) | 45.2 | 0.12 | 0.95 | 6 | 0.78 (0.70-0.87) | 78.6 | <0.0001 | 0.27 |
| | No | 1 | 0.86 (0.81-0.91) | | | | 5 | 0.89 (0.77-1.03) | 82.7 | <0.0001 | |
| Dairy | Yes | 0 | | | | NC | 1 | 0.30 (0.15-0.60) | | | 0.02 |
| | No | 6 | 0.85 (0.80-0.91) | 37.0 | 0.16 | | 10 | 0.84 (0.78-0.91) | 82.2 | <0.0001 | |
| Energy intake | Yes | 6 | 0.85 (0.80-0.91) | 37.0 | 0.16 | NC | 10 | 0.82 (0.76-0.89) | 83.9 | <0.0001 | 0.51 |
| | No | 0 | | | | | 1 | 0.94 (0.80-1.16) | | | |

*n*denotes the number of studies.
[1] P for heterogeneity within each subgroup,
[2] P for heterogeneity between subgroups with meta-regression analysis,
[3] P for heterogeneity between men and women (studies with genders mixed were excluded),
NC = not calculable

**Table S17: Study quality of studies included in the analysis of whole grains and coronary heart disease**

| Author, publication year | Represen-tativeness | Selection of non-exposed cohort | Exposure-ascertainment[1] | Demonstration of outcome not present at start | Adjustment for age | Adjustment for any other factor | Assess-ment of outcome | Long enough follow-up | Adequacy of follow-up[2] | Total score |
|---|---|---|---|---|---|---|---|---|---|---|
| Helnæs, 2016 | 1 | 1 | 1 | 1 | 1 | 1 | 1 | 1 | 1 | 9 |
| Wang, 2016 | 0 | 1 | 0 | 0 | 1 | 1 | 1 | 1 | 0 | 5 |
| Johnsen, 2015 | 1 | 1 | 1 | 0 | 1 | 1 | 1 | 1 | 1 | 8 |
| Rautiainen, 2012 | 1 | 1 | 1 | 1 | 1 | 1 | 1 | 1 | 1 | 9 |
| Jacobs, 2007 | 1 | 1 | 1 | 1 | 1 | 1 | 1 | 1 | 0 | 8 |
| Jensen, 2004 | 0 | 1 | 1 | 1 | 1 | 1 | 1 | 1 | 1 | 8 |
| Steffen, 2003 | 1 | 1 | 1 | 1 | 1 | 1 | 1 | 1 | 1 | 9 |
| Liu, 1999 | 0 | 1 | 1 | 1 | 1 | 1 | 1 | 1 | 1 | 8 |

[1] 1 point for validated self-reported questionnairesor interview
[2] 1 point for loss-to-follow-up less than 10%

**Table S18: Study quality of studies included in the analysis of whole grains and stroke**

| Author, publication year | Represen-tativeness | Selection of non-exposed cohort | Exposure-ascertainment[1] | Demonstration of outcome not present at start | Adjustment for age | Adjustment for any other factor | Assess-ment of outcome | Long enough follow-up | Adequacy of follow-up[2] | Total score |
|---|---|---|---|---|---|---|---|---|---|---|
| Wang, 2016 | 0 | 1 | 0 | 0 | 1 | 1 | 1 | 1 | 0 | 5 |
| Johnsen, 2015 | 1 | 1 | 1 | 0 | 1 | 1 | 1 | 1 | 1 | 8 |
| Mizrahi, 2009 | 1 | 1 | 1 | 1 | 1 | 1 | 1 | 1 | 0 | 8 |
| Jacobs, 2007 | 1 | 1 | 1 | 1 | 1 | 1 | 1 | 1 | 0 | 8 |
| Steffen, 2003 | 1 | 1 | 1 | 1 | 1 | 1 | 1 | 1 | 1 | 9 |
| Liu, 2000 | 0 | 1 | 1 | 1 | 1 | 1 | 1 | 1 | 1 | 8 |

[1] 1 point for validated self-reported questionnairesor interview
[2] 1 point for loss-to-follow-up less than 10%

**Table S19: Study quality of studies included in the analysis of whole grains and cardiovascular disease**

| Author, publication year | Represen-tativeness | Selection of non-exposed cohort | Exposure-ascertainment[1] | Demonstration of outcome not present at start | Adjustment for age | Adjustment for any other factor | Assess-ment of outcome | Long enough follow-up | Adequacy of follow-up[2] | Total score |
|---|---|---|---|---|---|---|---|---|---|---|
| Wang, 2016 | 0 | 1 | 0 | 0 | 1 | 1 | 1 | 1 | 0 | 5 |
| Huang, 2015 | 1 | 1 | 1 | 1 | 1 | 1 | 1 | 1 | 0 | 8 |
| Johnsen, 2015 | 1 | 1 | 1 | 0 | 1 | 1 | 1 | 1 | 1 | 8 |
| Sonestedt, 2015 | 1 | 1 | 1 | 1 | 1 | 1 | 1 | 1 | 0 | 8 |
| Wu, 2015, HPFS | 0 | 1 | 1 | 1 | 1 | 1 | 1 | 1 | 1 | 8 |
| Wu, 2015, NHS | 0 | 1 | 1 | 1 | 1 | 1 | 1 | 1 | 1 | 8 |
| Buil-Cosiales, 2014 | 0 | 1 | 1 | 1 | 1 | 1 | 1 | 1 | 0 | 7 |
| Fitzgerald, 2012 | 1 | 1 | 1 | 1 | 1 | 1 | 1 | 1 | 1 | 9 |
| Jacobs, 2007 | 1 | 1 | 1 | 1 | 1 | 1 | 1 | 1 | 0 | 8 |
| Sahyoun, 2006 | 1 | 1 | 1 | 1 | 1 | 1 | 1 | 1 | 0 | 8 |

[1] 1 point for validated self-reported questionnairesor interview
[2] 1 point for loss-to-follow-up less than 10%

**Table S20: Study quality of studies included in the analysis of whole grains and total cancer**

| Author, publication year | Represen-tativeness | Selection of non-exposed cohort | Exposure-ascertainment[1] | Demonstration of outcome not present at start | Adjustment for age | Adjustment for any other factor | Assess-ment of outcome | Long enough follow-up | Adequacy of follow-up[2] | Total score |
|---|---|---|---|---|---|---|---|---|---|---|
| Huang, 2015 | 1 | 1 | 1 | 1 | 1 | 1 | 1 | 1 | 0 | 8 |
| Johnsen, 2015 | 1 | 1 | 1 | 0 | 1 | 1 | 1 | 1 | 1 | 8 |
| Wu, 2015, HPFS | 0 | 1 | 1 | 1 | 1 | 1 | 1 | 1 | 1 | 8 |
| Wu, 2015, NHS | 0 | 1 | 1 | 1 | 1 | 1 | 1 | 1 | 1 | 8 |
| Buil-Cosiales, 2014 | 0 | 1 | 1 | 1 | 1 | 1 | 1 | 1 | 0 | 7 |
| Jacobs, 2007 | 1 | 1 | 1 | 1 | 1 | 1 | 1 | 1 | 0 | 8 |

[1] 1 point for validated self-reported questionnaires or interview
[2] 1 point for loss-to-follow-up less than 10%

**Table S21: Study quality of studies included in the analysis of whole grains and all-cause mortality**

| Author, publication year | Represen-tativeness | Selection of non-exposed cohort | Exposure-ascertainment[1] | Demonstration of outcome not present at start[2] | Adjustment for age | Adjustment for any other factor | Assess-ment of outcome | Long enough follow-up | Adequacy of follow-up[3] | Total score |
|---|---|---|---|---|---|---|---|---|---|---|
| Wang, 2016 | 0 | 1 | 0 | 0 | 1 | 1 | 1 | 1 | 0 | 5 |
| Huang, 2015 | 1 | 1 | 1 | 1 | 1 | 1 | 1 | 1 | 0 | 8 |
| Johnsen, 2015 | 1 | 1 | 1 | 0 | 1 | 1 | 1 | 1 | 1 | 8 |
| Wu, 2015, HPFS | 0 | 1 | 1 | 1 | 1 | 1 | 1 | 1 | 1 | 8 |
| Wu, 2015, NHS | 0 | 1 | 1 | 1 | 1 | 1 | 1 | 1 | 1 | 8 |
| Boggs, 2014 | 1 | 1 | 1 | 1 | 1 | 1 | 1 | 1 | 1 | 9 |
| Buil-Cosiales, 2014 | 0 | 1 | 1 | 1 | 1 | 1 | 1 | 1 | 0 | 7 |
| van den Brandt, 2011 | 1 | 1 | 1 | 1 | 1 | 1 | 1 | 1 | 1 | 9 |
| Jacobs, 2007 | 1 | 1 | 1 | 1 | 1 | 1 | 1 | 1 | 0 | 8 |
| Sahyoun, 2006 | 1 | 1 | 1 | 1 | 1 | 1 | 1 | 1 | 0 | 8 |
| Steffen, 2003 | 1 | 1 | 1 | 1 | 1 | 1 | 1 | 1 | 1 | 9 |

[1] 1 point for validated self-reported questionnaires or interview
[2] 1 point for exclusion of prevalent cardiovascular disease or cancer cases
[3] 1 point for loss-to-follow-up less than 10%
