# Supplementary material for: Whole grain consumption and risk of cardiovascular disease, cancer, and all cause and cause specific mortality: systematic review and dose-response meta-analysis of prospective studies
Source: BMJ. 2016 Jun 14;353:i2716. doi: 10.1136/bmj.i2716 (PMC4908315; doi:10.1136/bmj.i2716)
Supplement: Supplementary file 2 — Appendix 2: Supplementary figures S1-S109 [file aund029745.ww2_default.pdf]

Supplementary material to Aune D, Keum N, Giovannucci E, Fadnes LT, Boffetta P, Greenwood DC, Tonstad S, Vatten LJ, Riboli E, Norat T. Whole grain consumption and risk of cardiovascular disease, cancer and all cause and cause specific mortality: systematic review and dose-response meta-analysis of prospective studies. *BMJ* 2016;353:i2716

Appendix 2: Supplementary figures [posted as supplied by author]

Figure S1. Whole grains and coronary heart disease, high versus low analysis

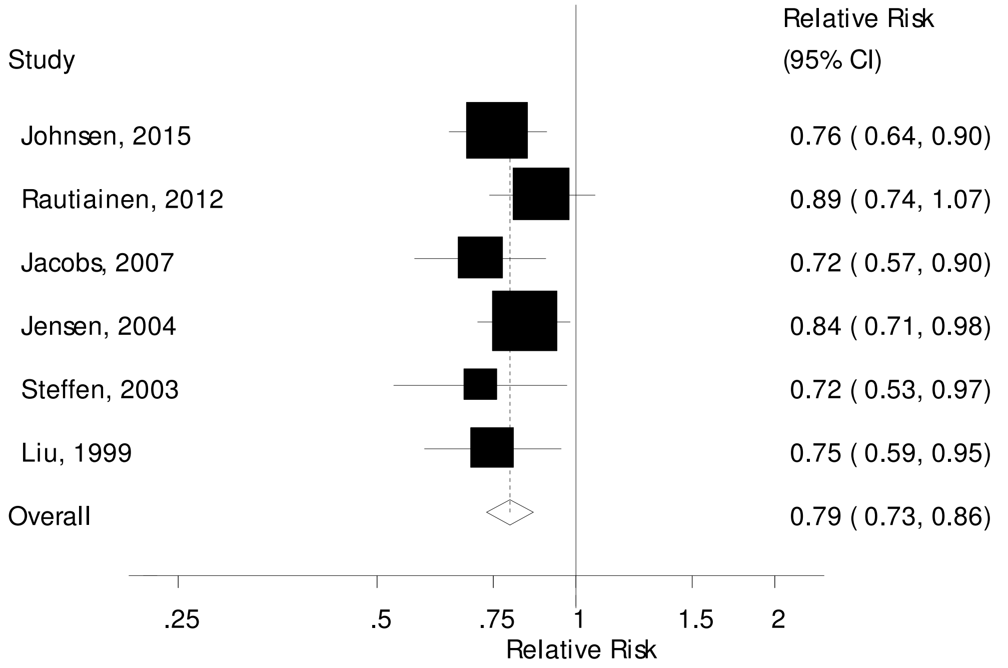

Figure S2.Whole grains and coronary heart disease, scatter plot from nonlinear dose-response analysis

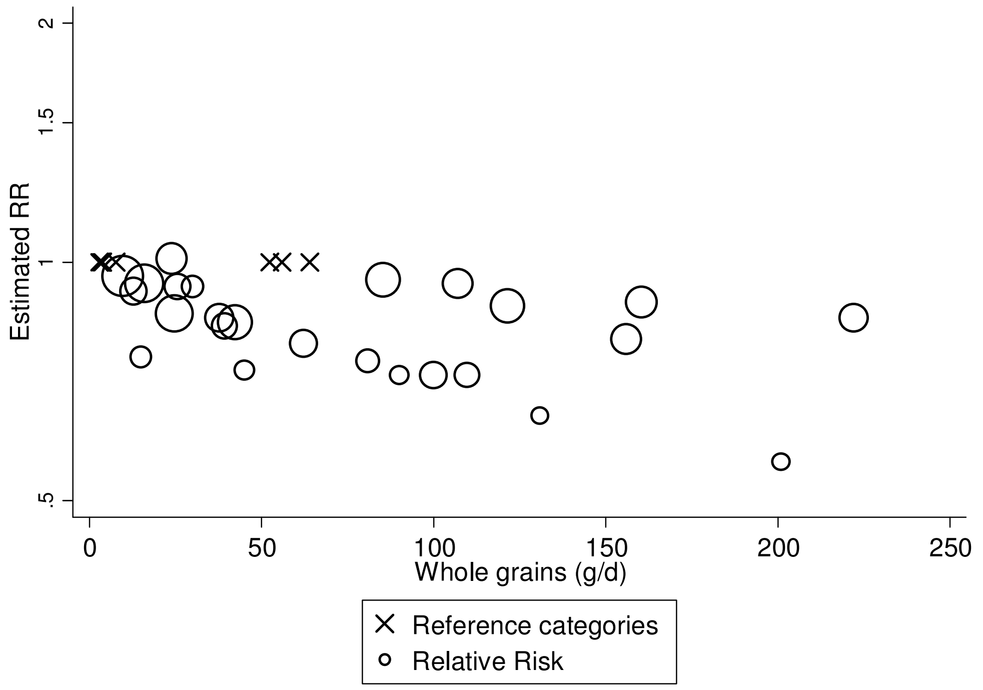

Figure S3. Whole grains and stroke, high vs. low analysis

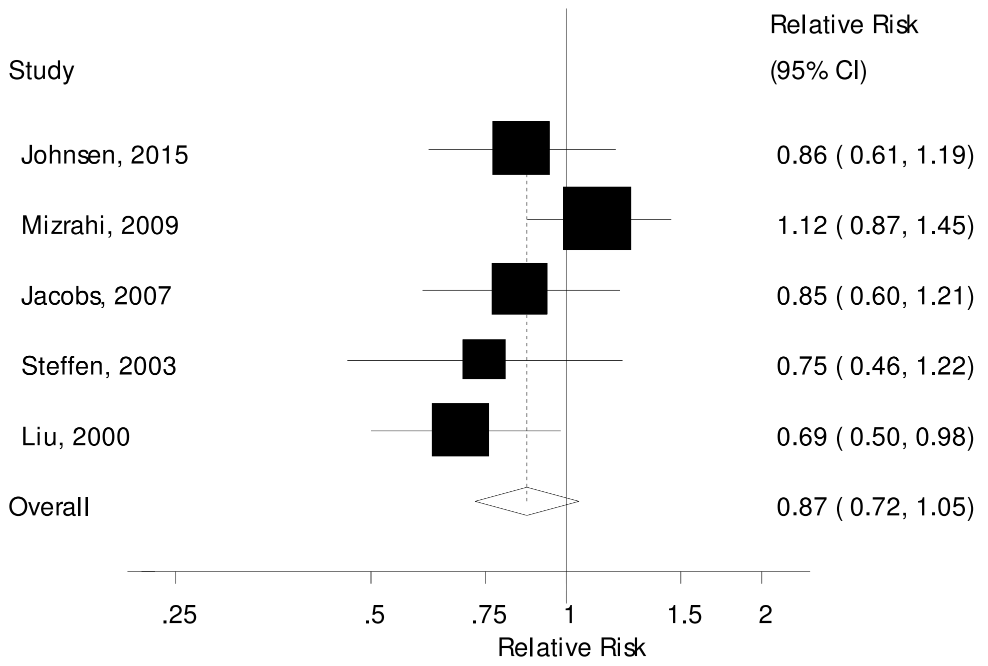

Figure S4. Whole grains and stroke, scatter plot from nonlinear dose-response analysis

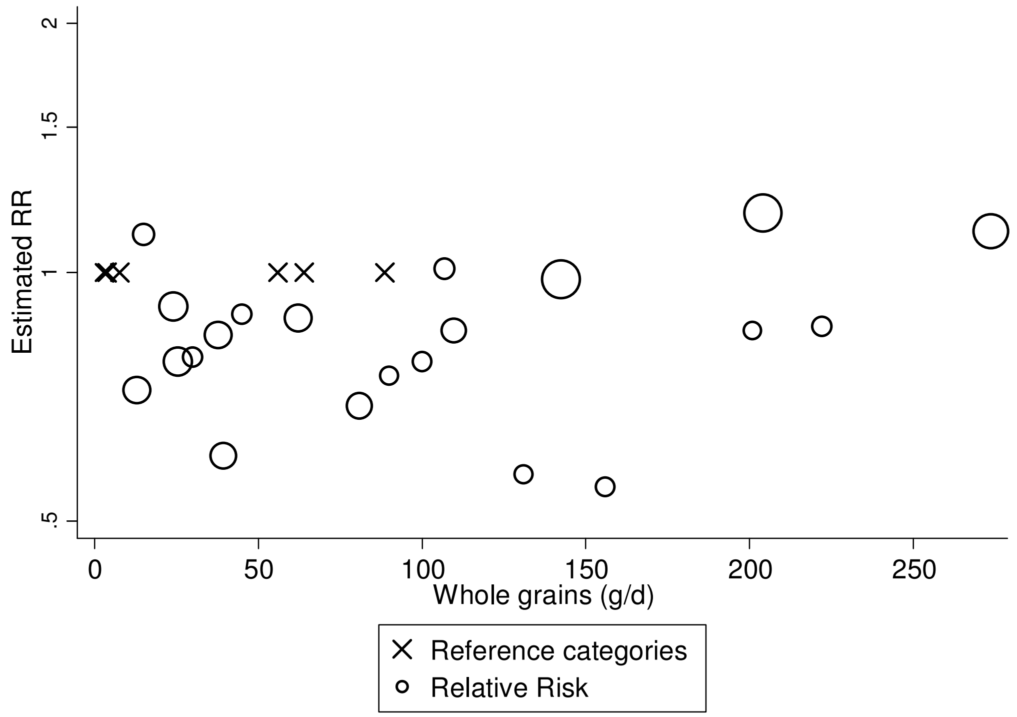

Figure S5. Whole grains and cardiovascular disease, high vs. low analysis

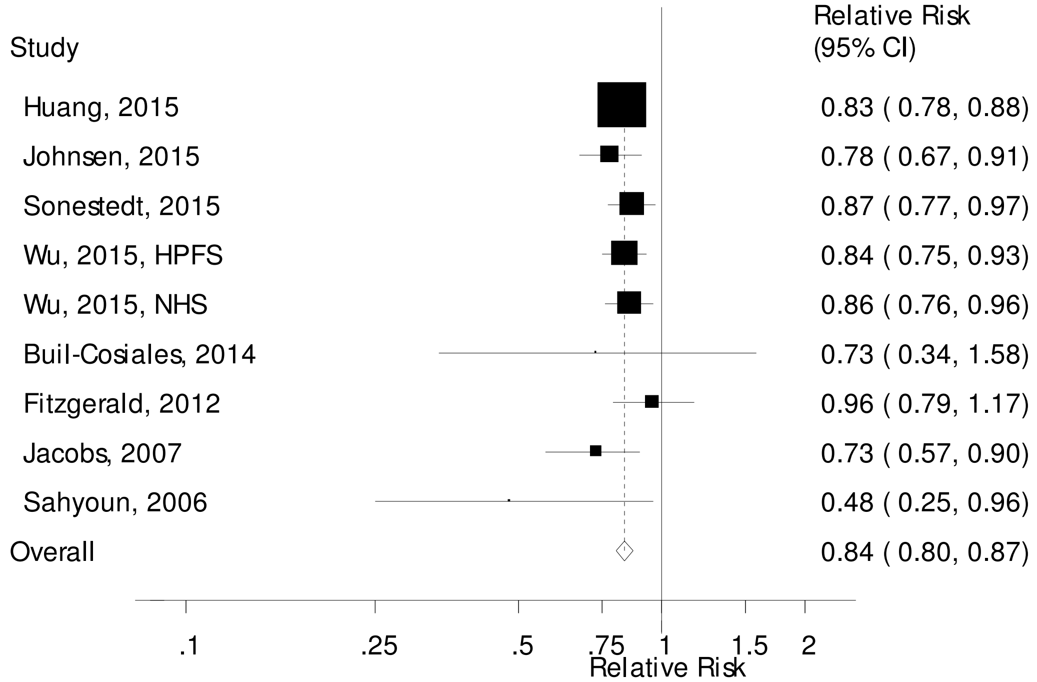

Figure S6. Whole grains and cardiovascular disease, scatter plot from nonlinear dose-response analysis

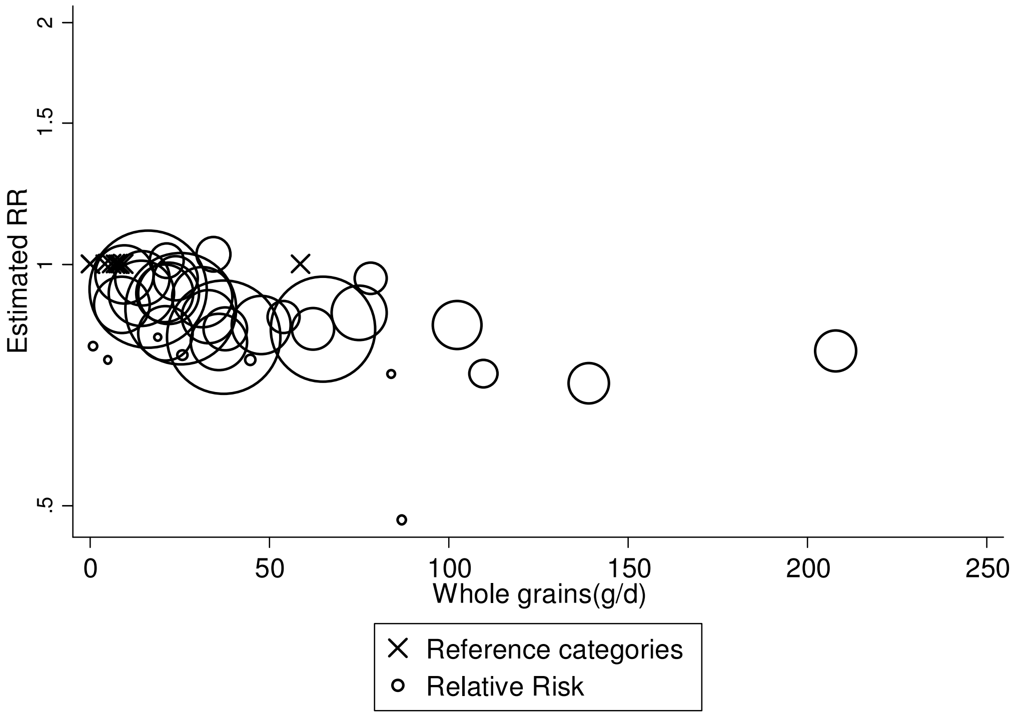

Figure S7. Whole grains and total cancer mortality, high vs. low analysis

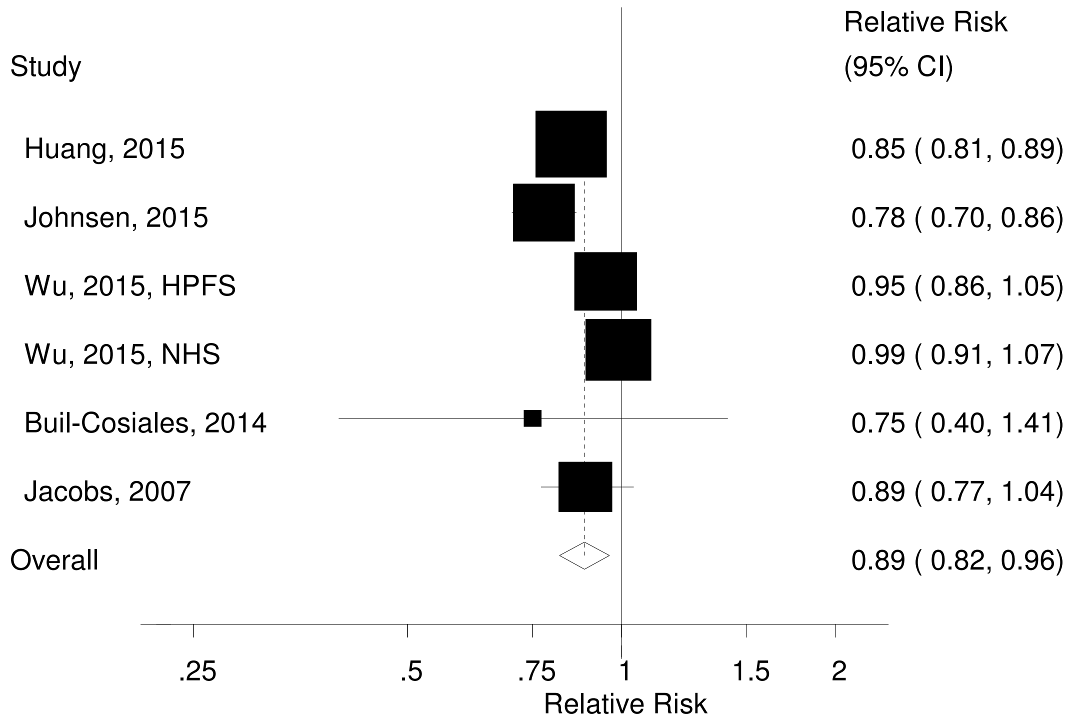

Figure S8. Whole grains and total cancer mortality, scatter plot from nonlinear dose-response analysis

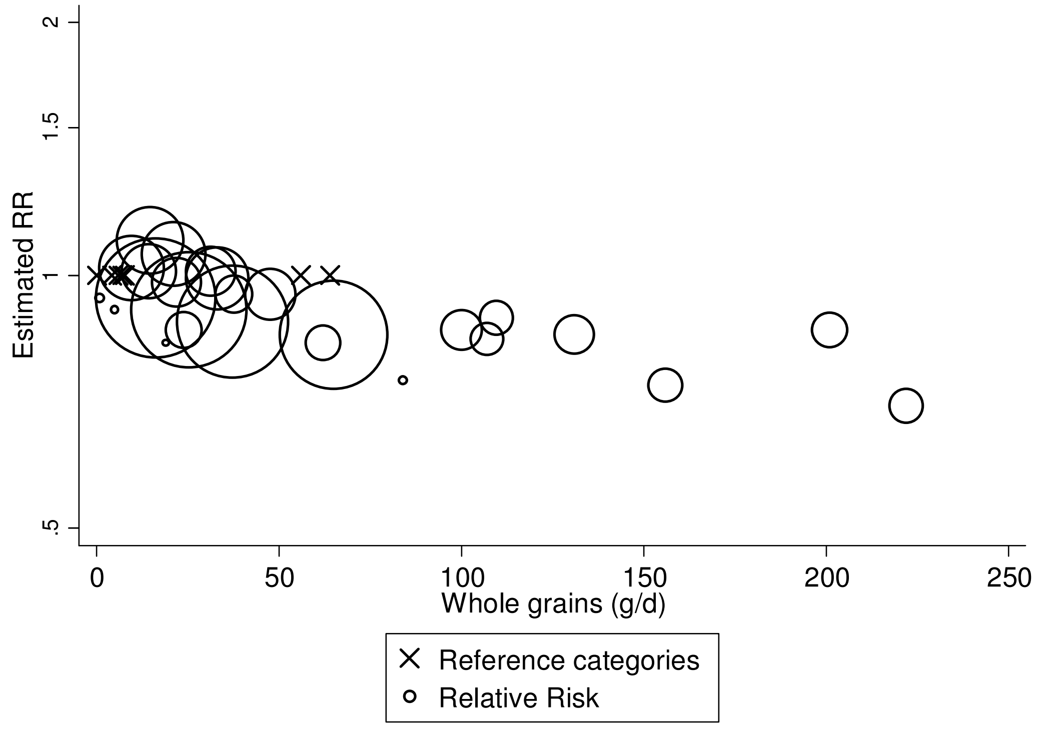

Figure S9. Whole grains and all-cause mortality, high vs. low analysis

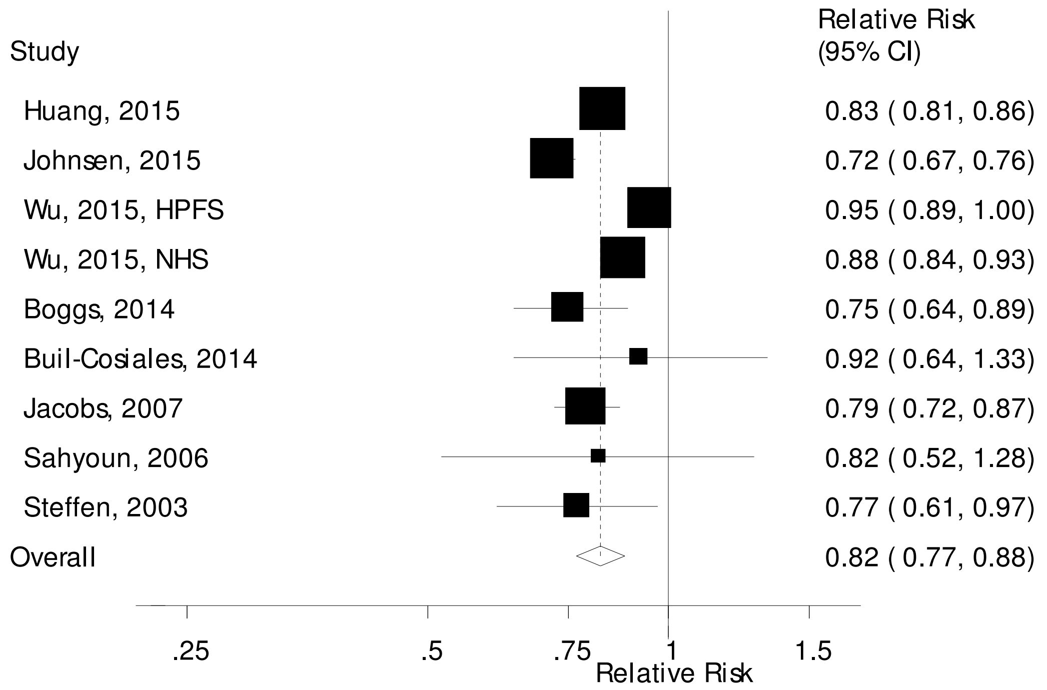

Figure S10. Whole grains and all-cause mortality, scatter plot from nonlinear dose-response analysis

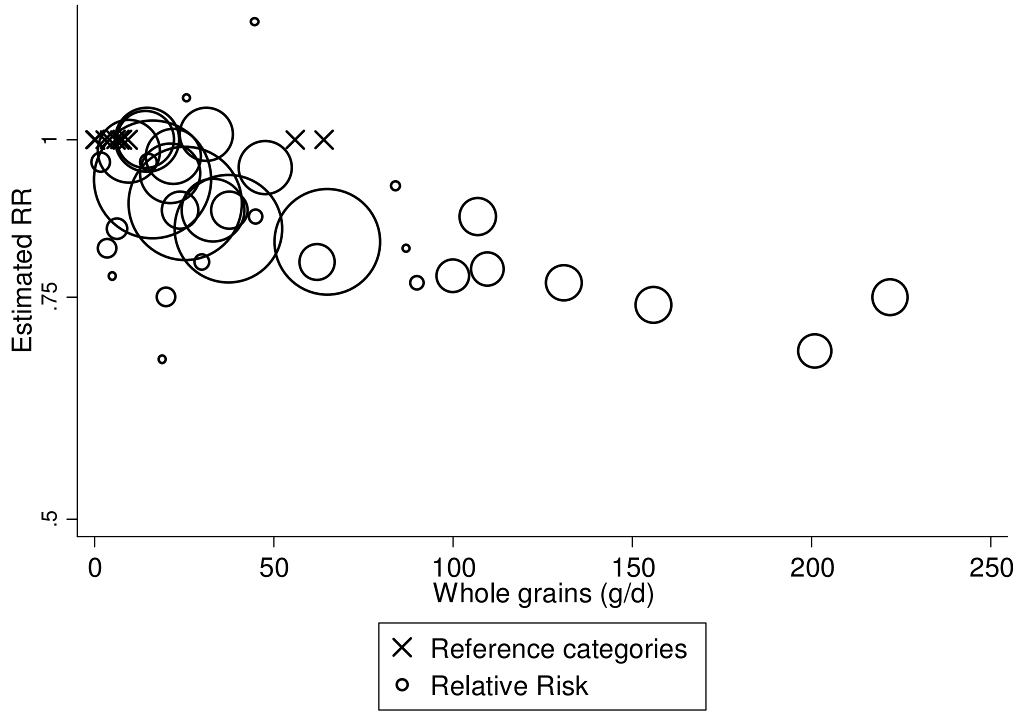

Figure S11. Whole grains and respiratory disease mortality, high vs. low analysis

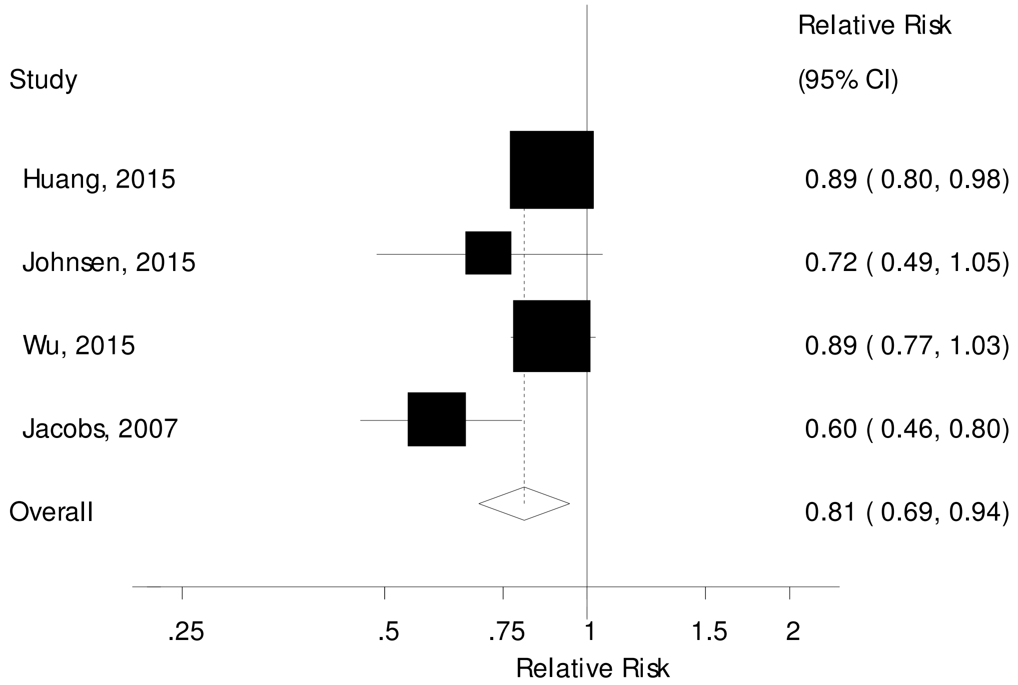

Figure S12. Whole grains and respiratory disease mortality, scatter plot from nonlinear dose-response analysis

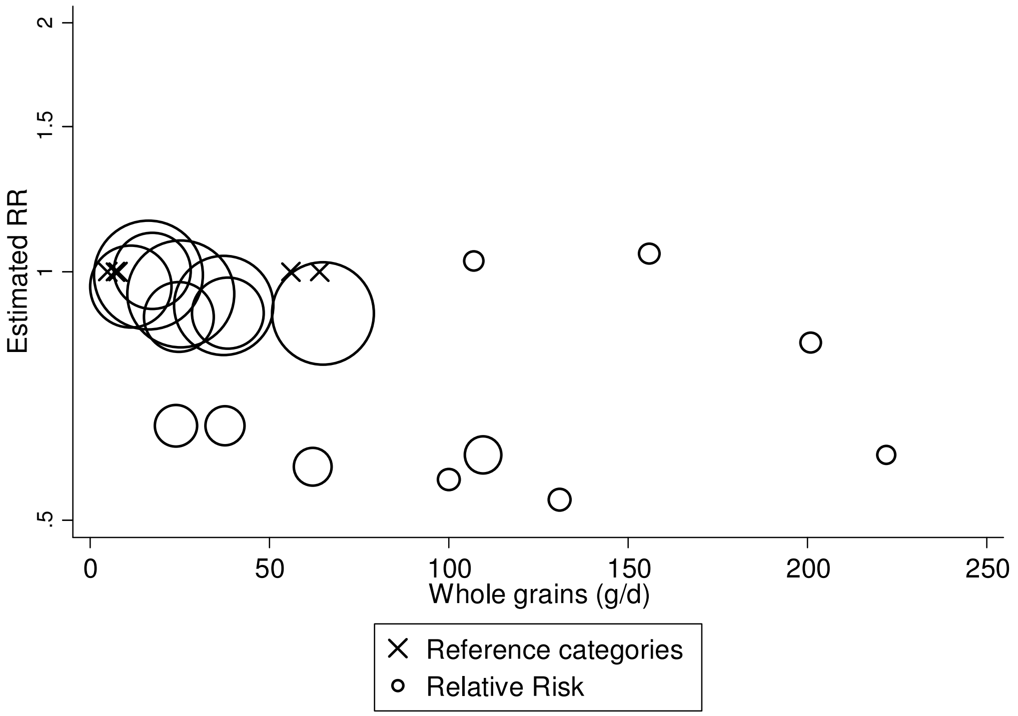

Figure S13. Whole grains and diabetes mortality, high vs. low analysis

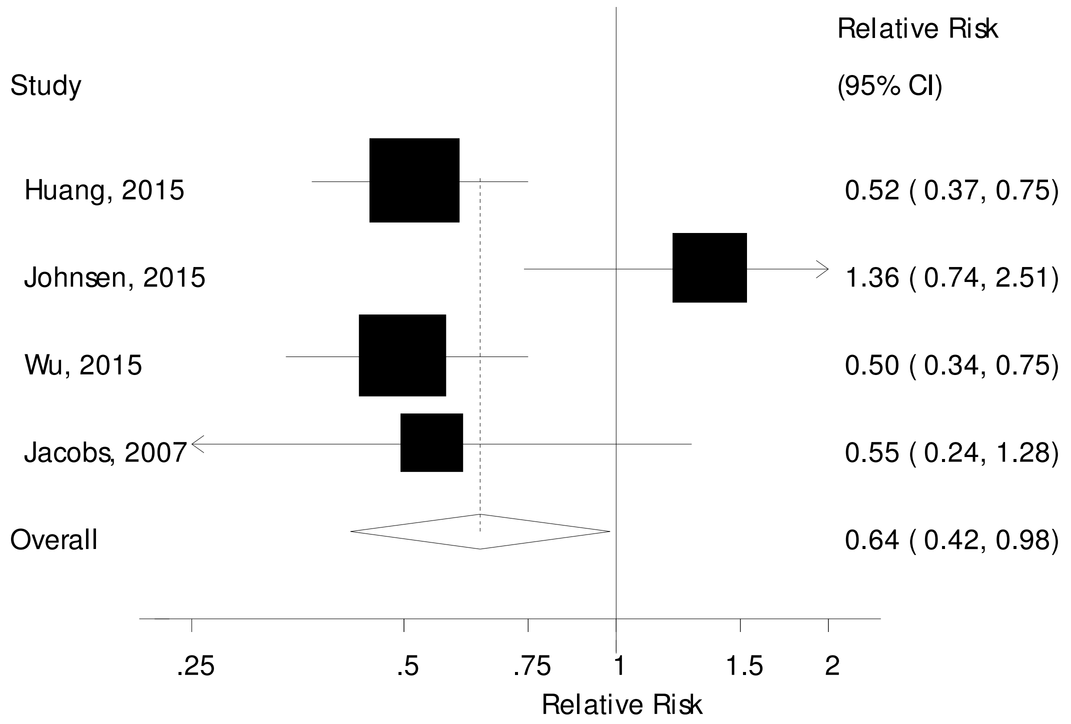

Figure S14. Whole grains and diabetes mortality, scatter plot from nonlinear dose-response analysis

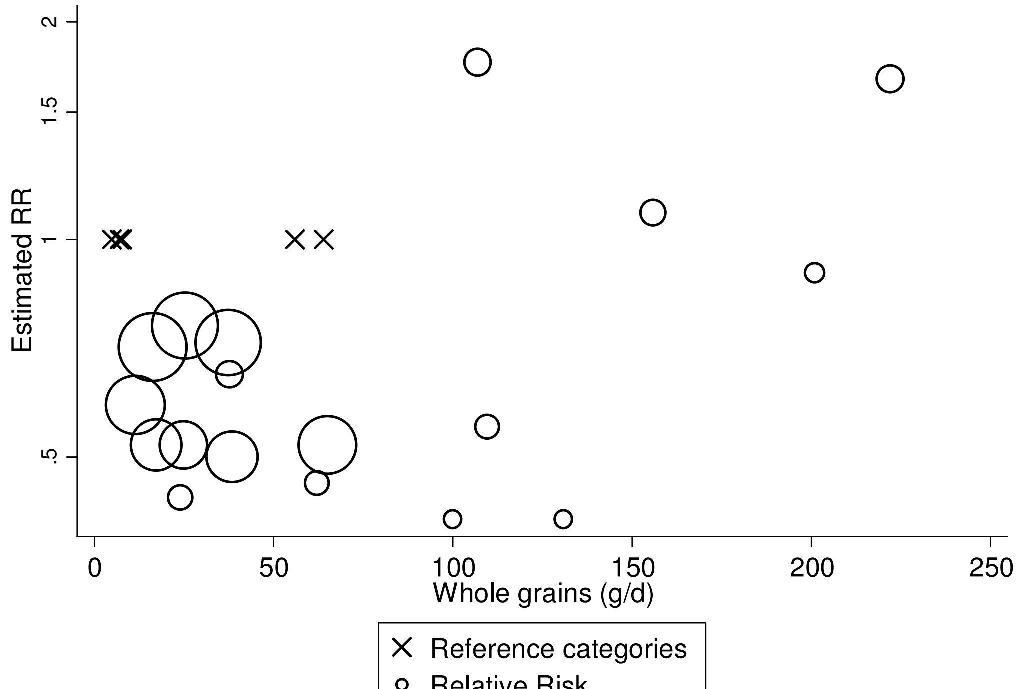

Figure S15. Whole grains and infectious disease mortality, high vs. low analysis

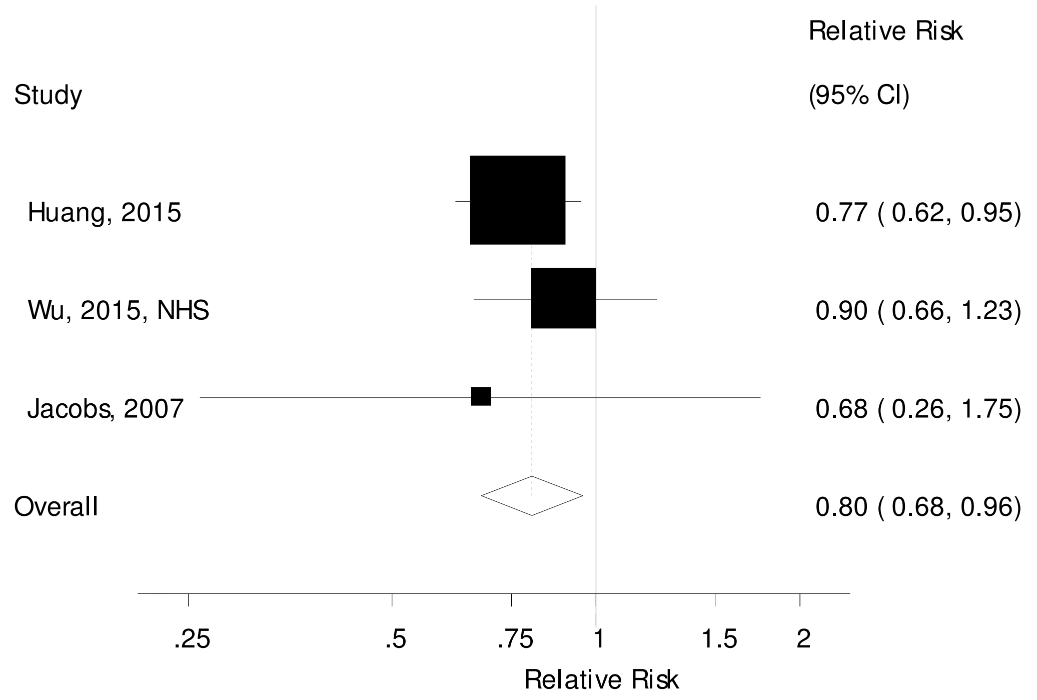

Figure S16. Whole grains and infectious disease mortality, scatter plot from nonlinear dose-response analysis

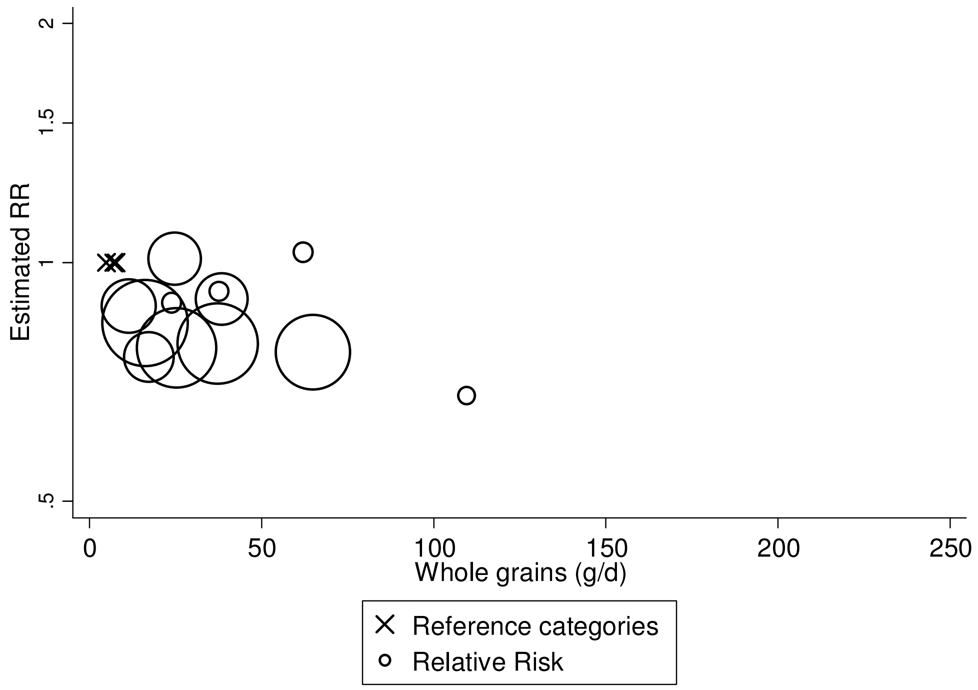

Figure S17. Whole grains and nervous system disease mortality, high vs. low analysis

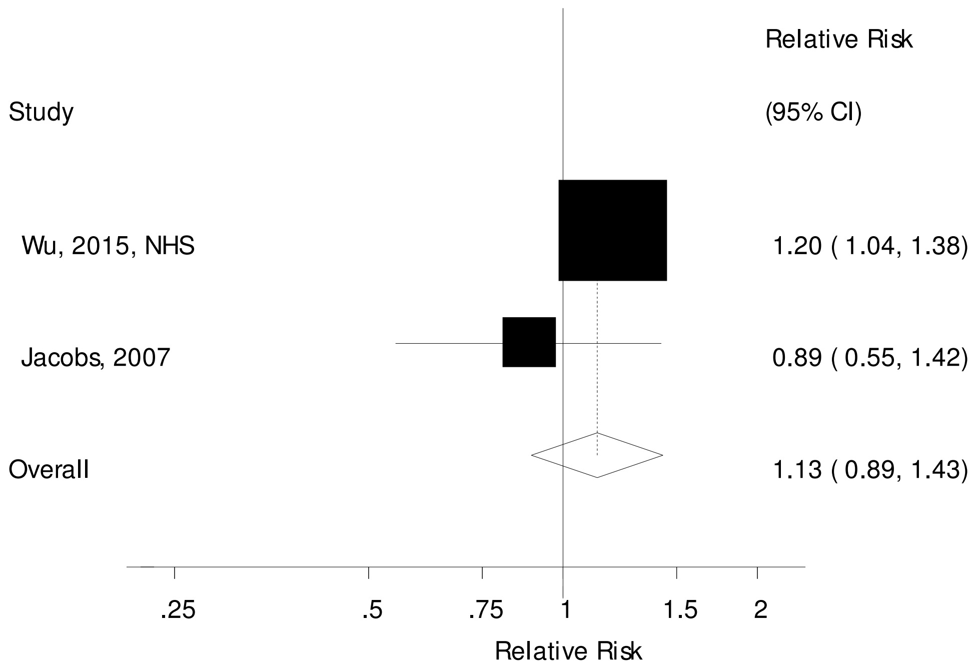

Figure S18. Whole grains and nervous system disease mortality, scatter plot from nonlinear dose-response analysis

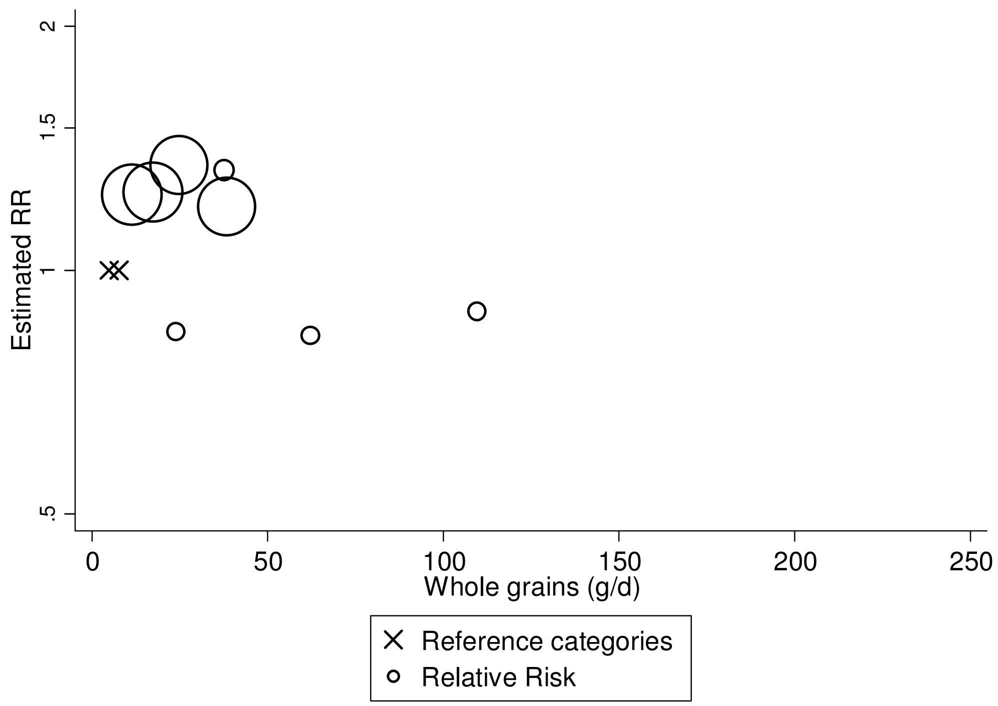

Figure S19. Whole grains and non-cardiovascular, non-cancer causes of death, high vs. low analysis

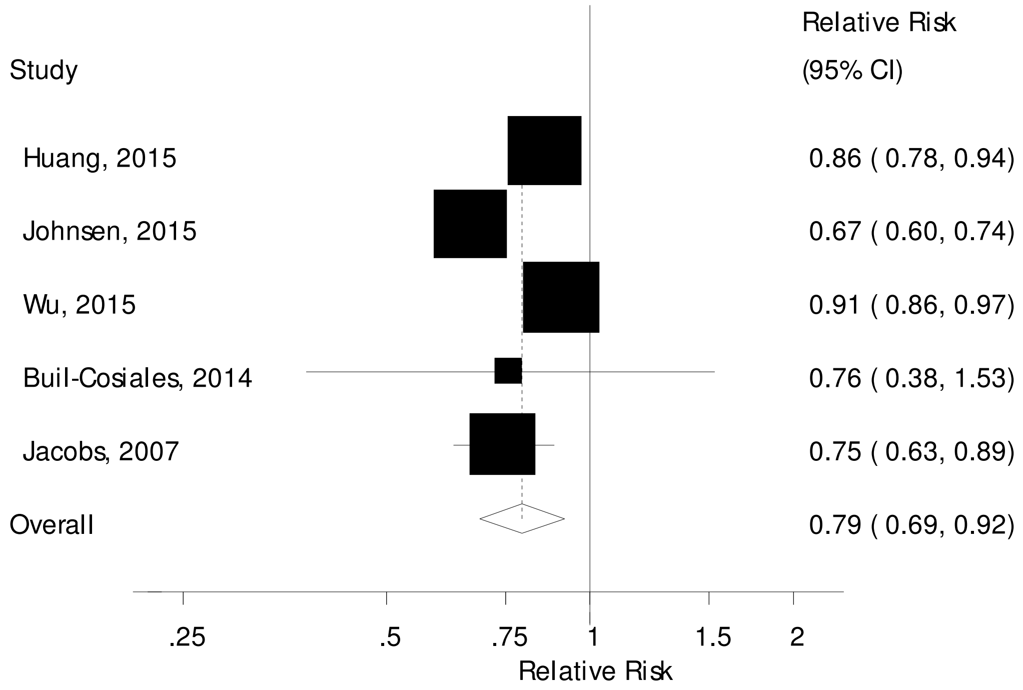

Figure S20. Whole grains and non-cardiovascular, non-cancer causes of death, scatter plot from nonlinear dose-response analysis

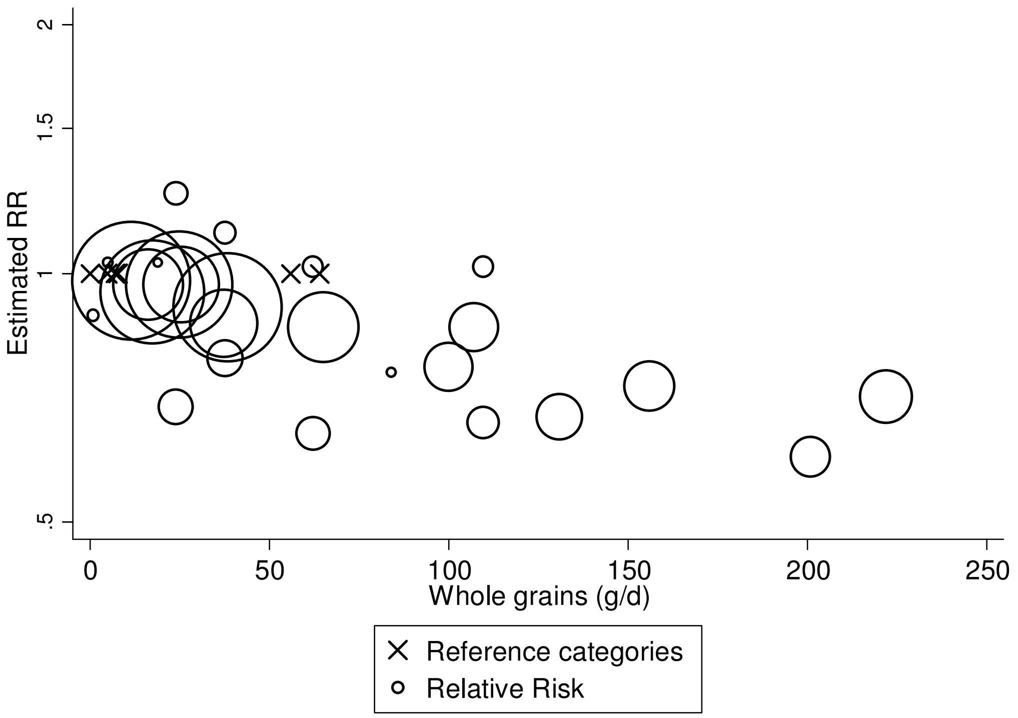

Figure S21. Whole grain bread and coronary heart disease, high vs. low

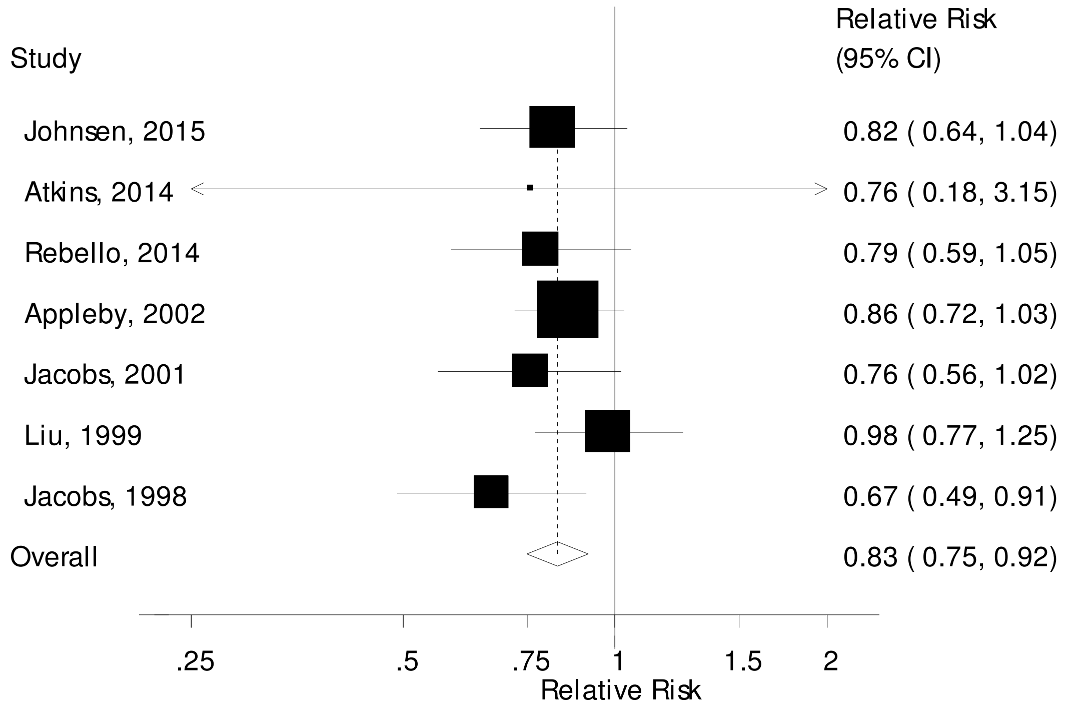

Figure S22. Whole grain bread and coronary heart disease, per 90 g/d

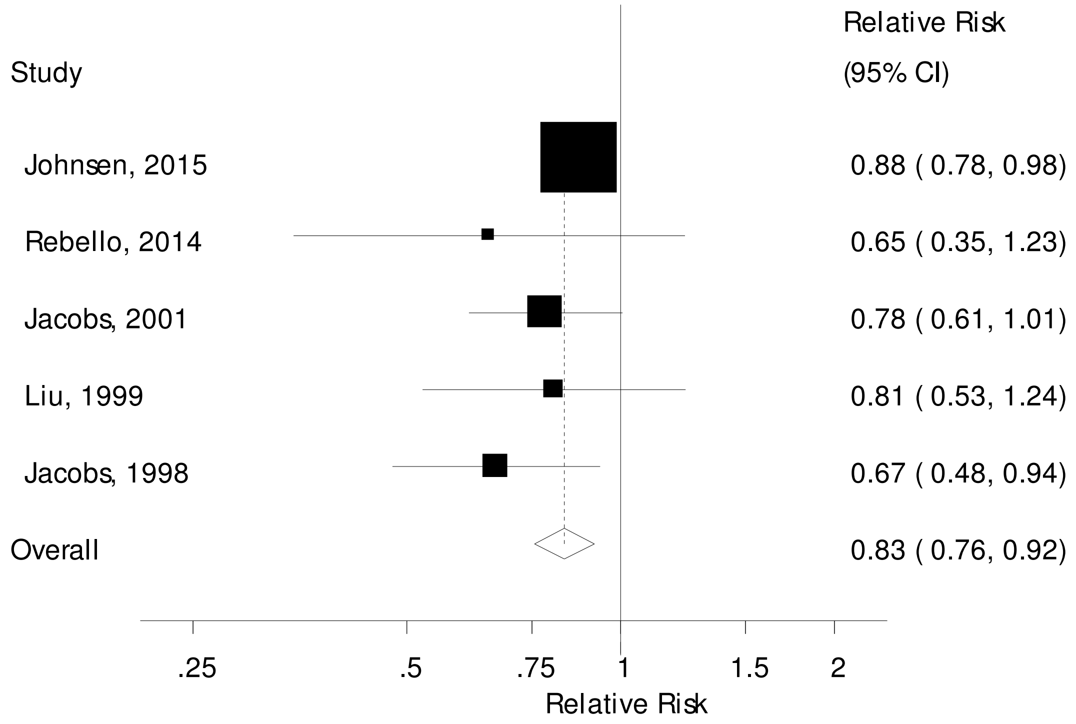

Figure S23. Whole grain bread and coronary heart disease, nonlinear dose-response

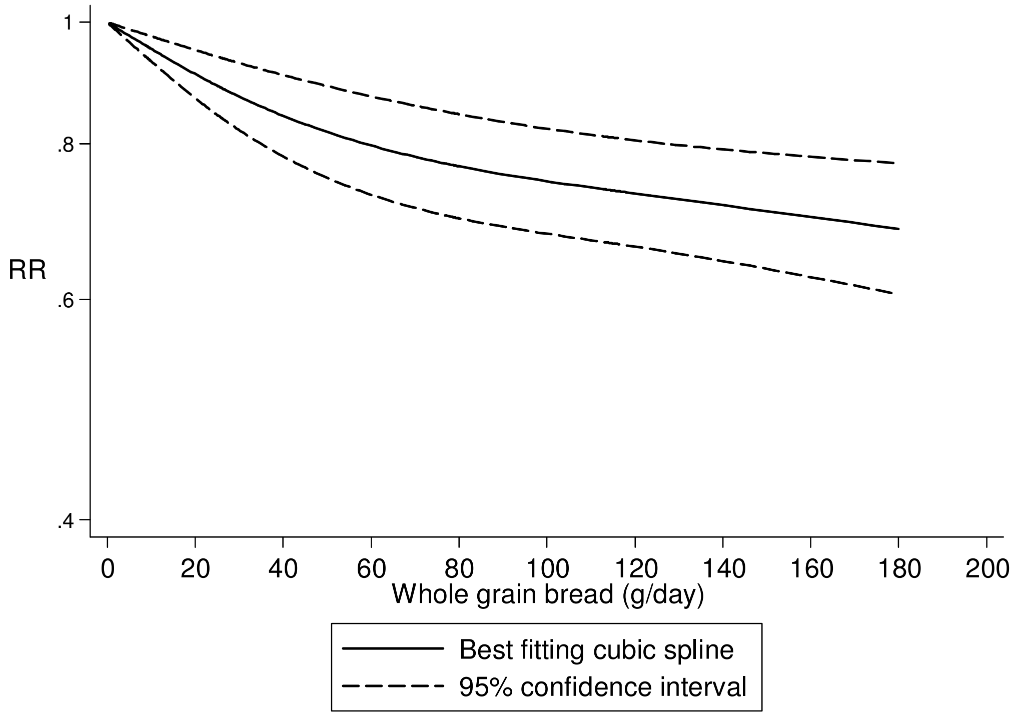

Figure S24. Whole grain breakfast cereal and coronary heart disease, high vs. low

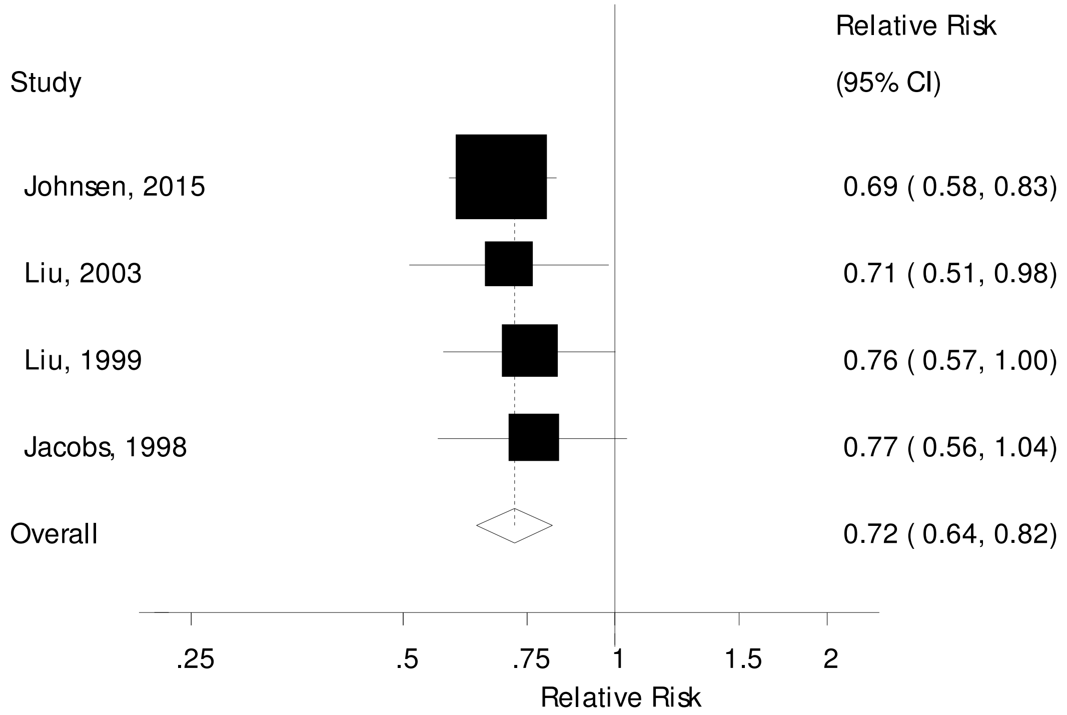

Figure S25. Whole grain breakfast cereal and coronary heart disease, per 30 g/d

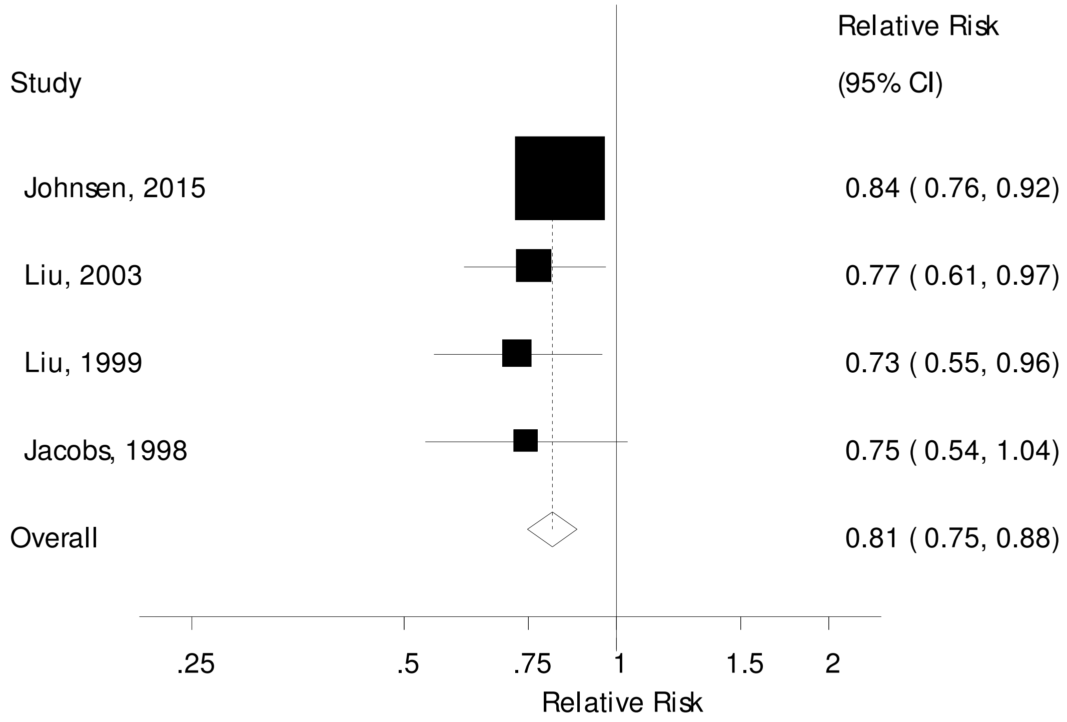

Figure S26. Whole grain breakfast cereal and coronary heart disease, nonlinear dose-response

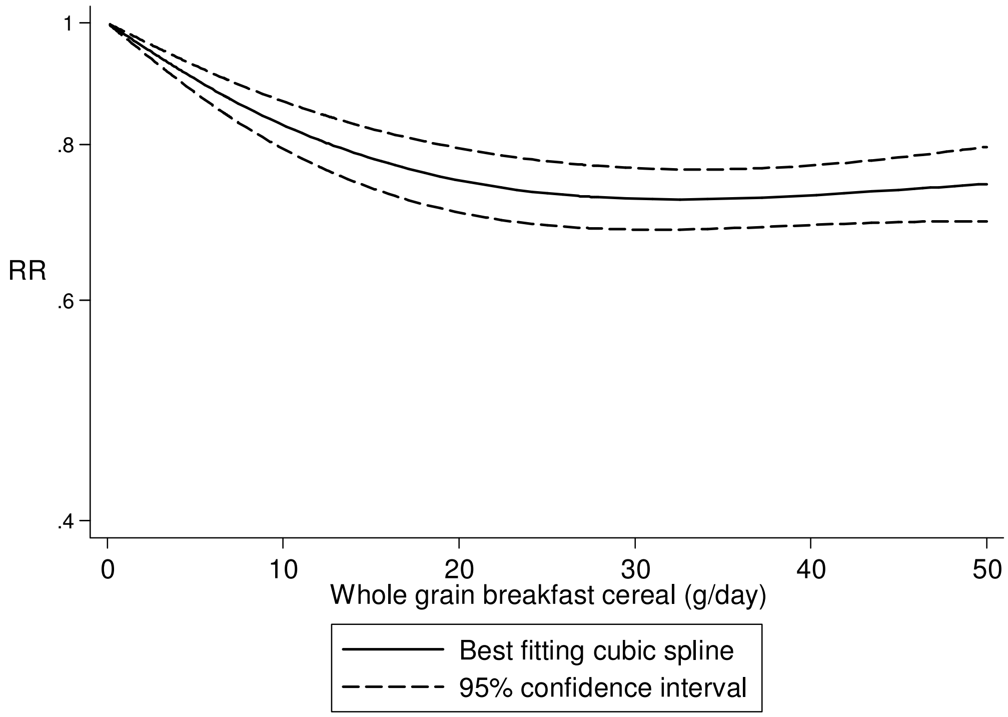

Figure S27. Rye and coronary heart disease, high vs. low

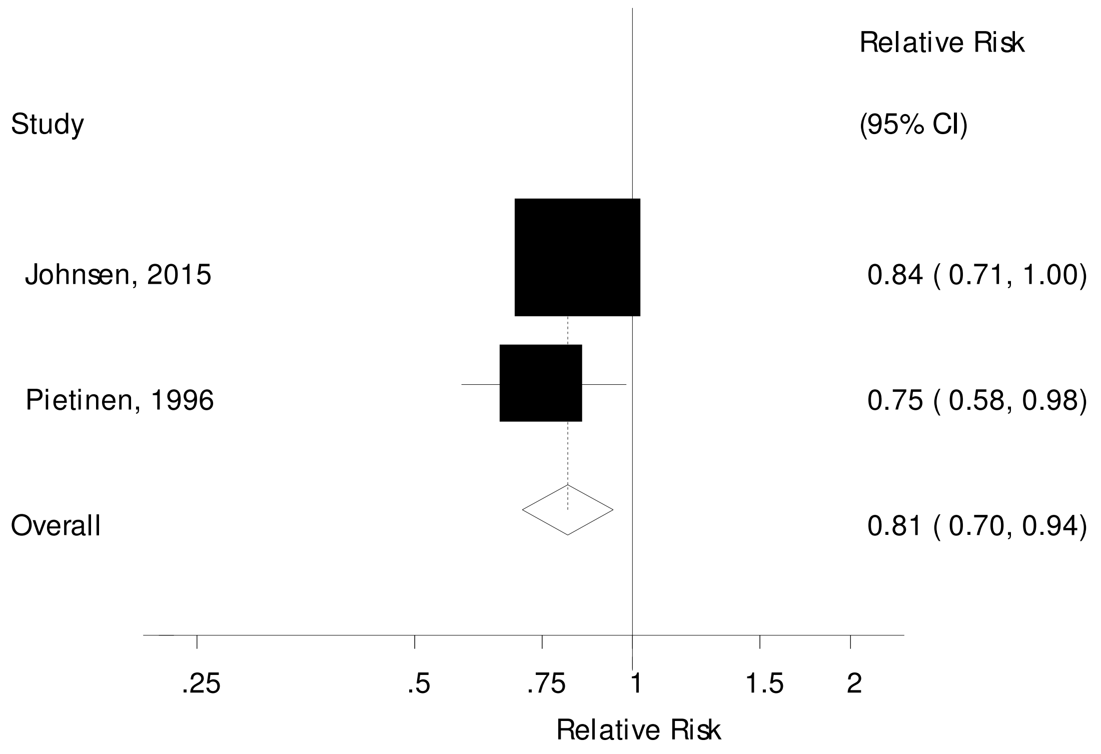

Figure S28. Rye and coronary heart disease, per 30 g/d

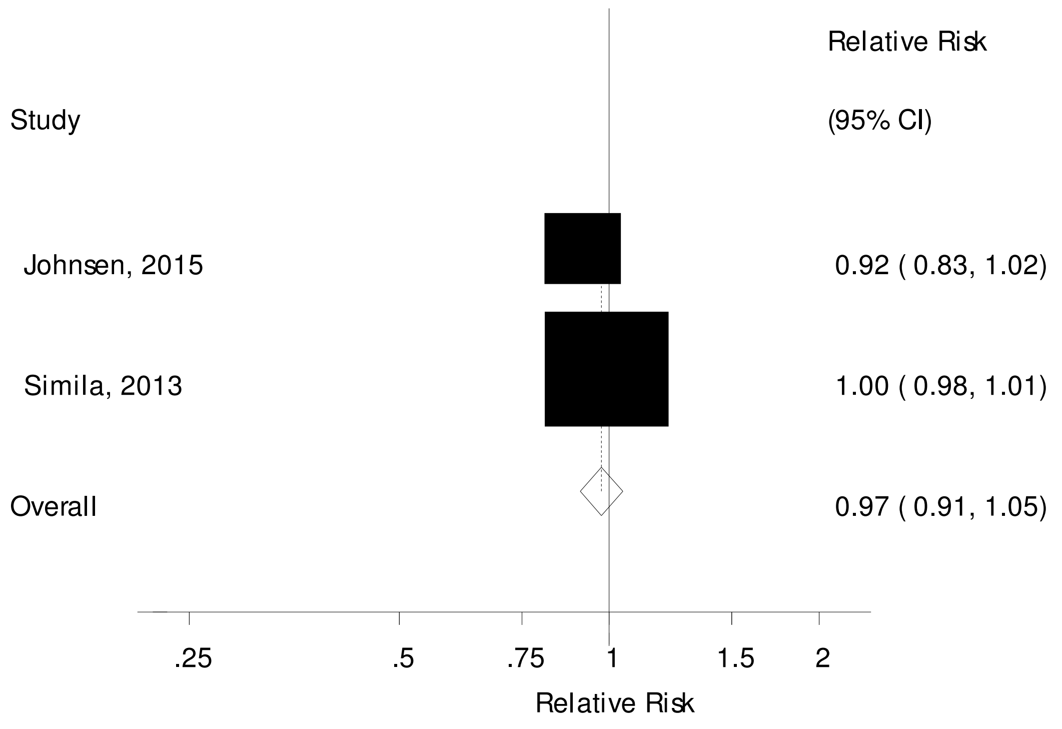

Figure S29. Bran and coronary heart disease, high vs. low

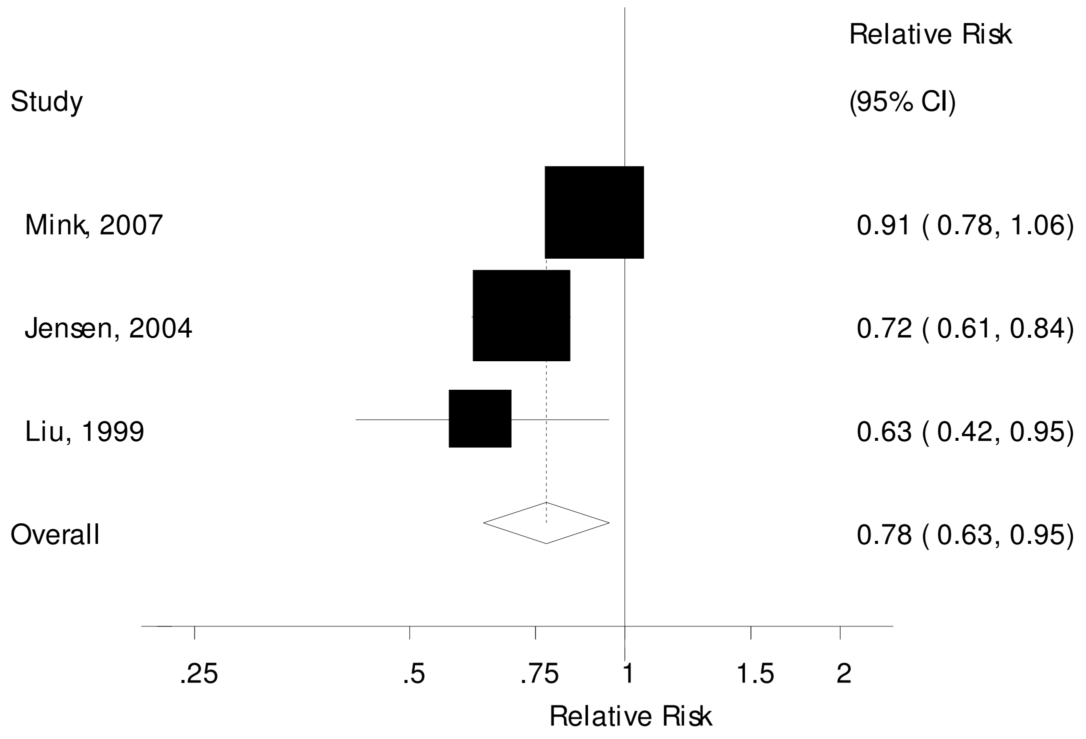

Figure S30. Bran and coronary heart disease, per 10 g/d

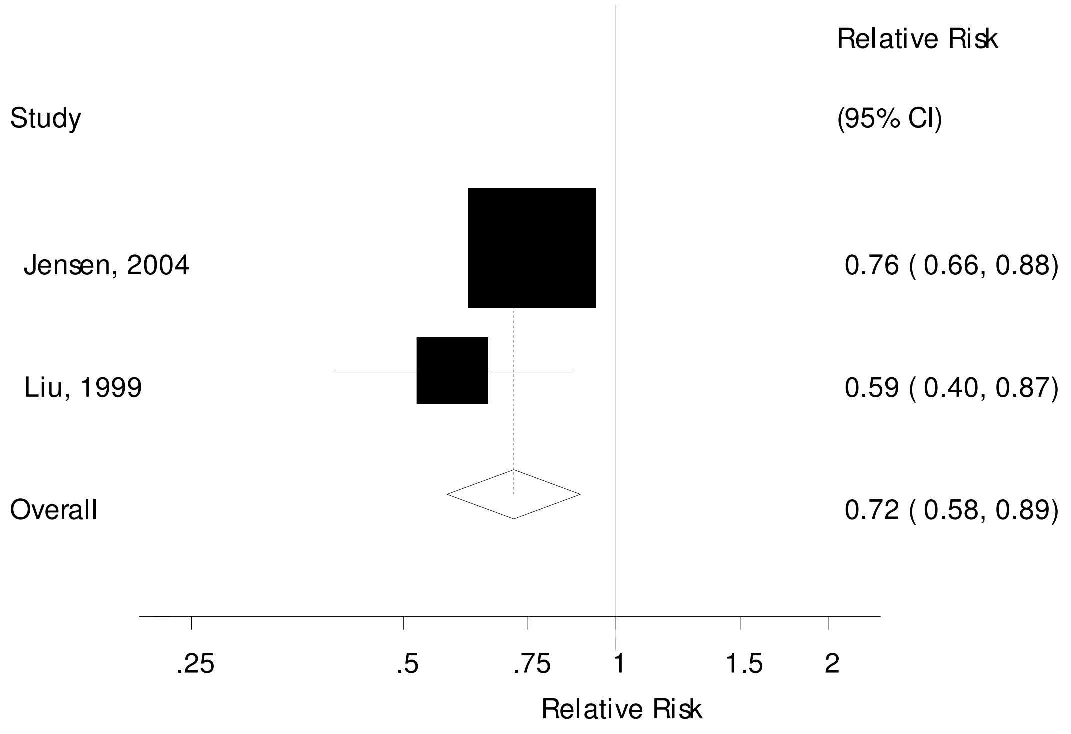

Figure S31. Germ and coronary heart disease, high vs. low

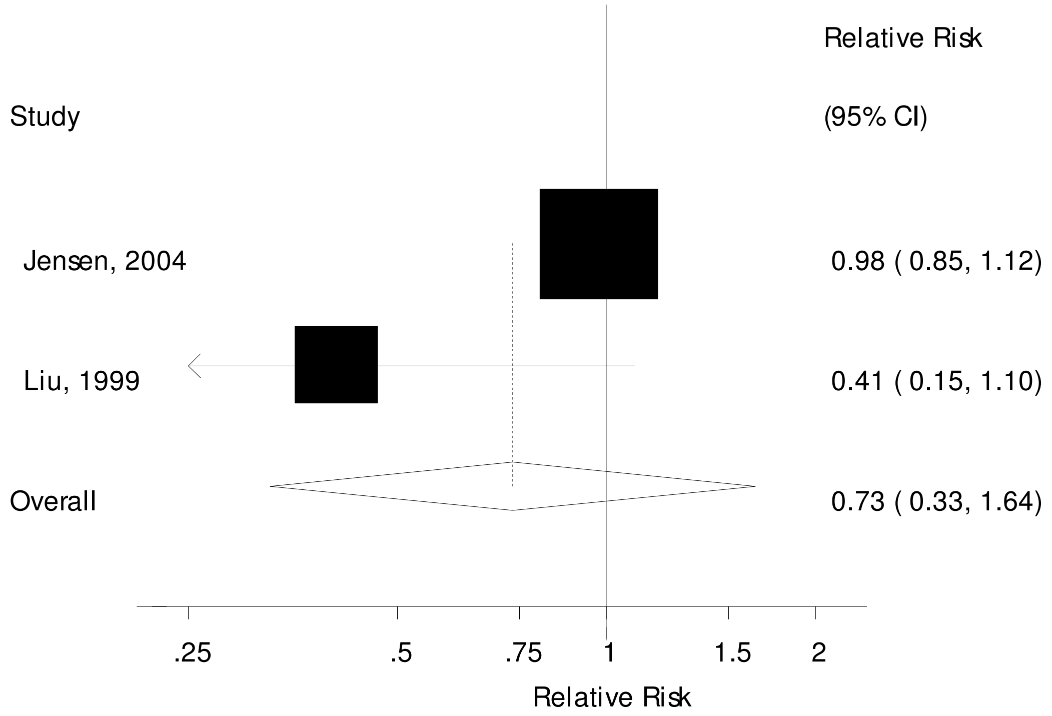

Figure S32. Germ and coronary heart disease, per 2 g/d

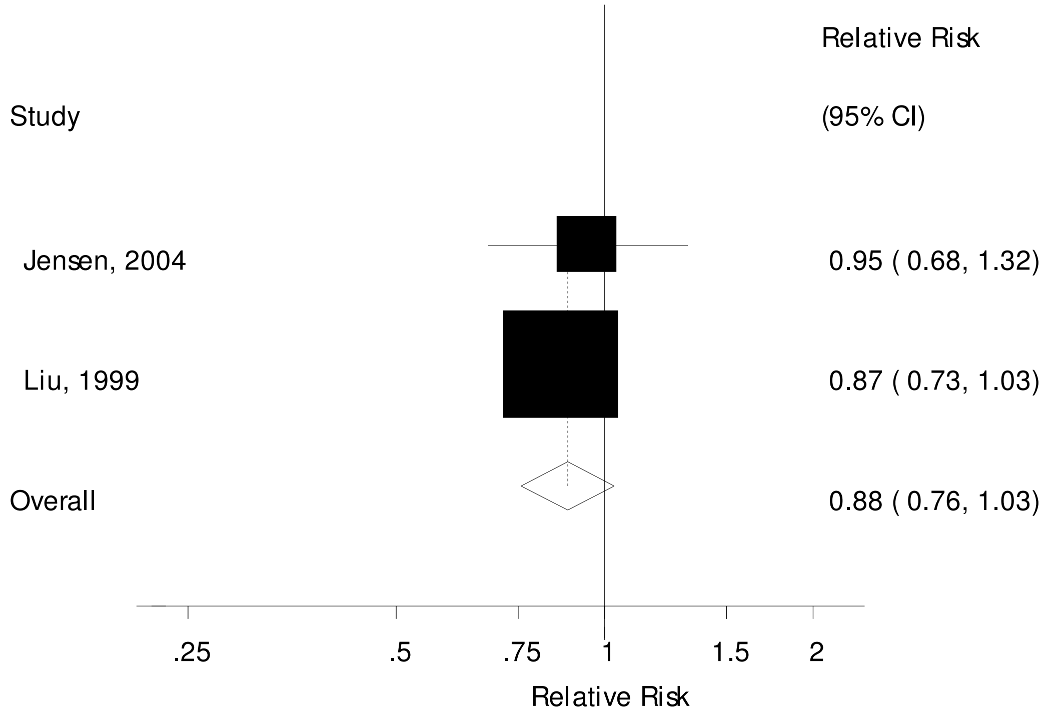

Figure S33. Refined grains and coronary heart disease, high vs. low

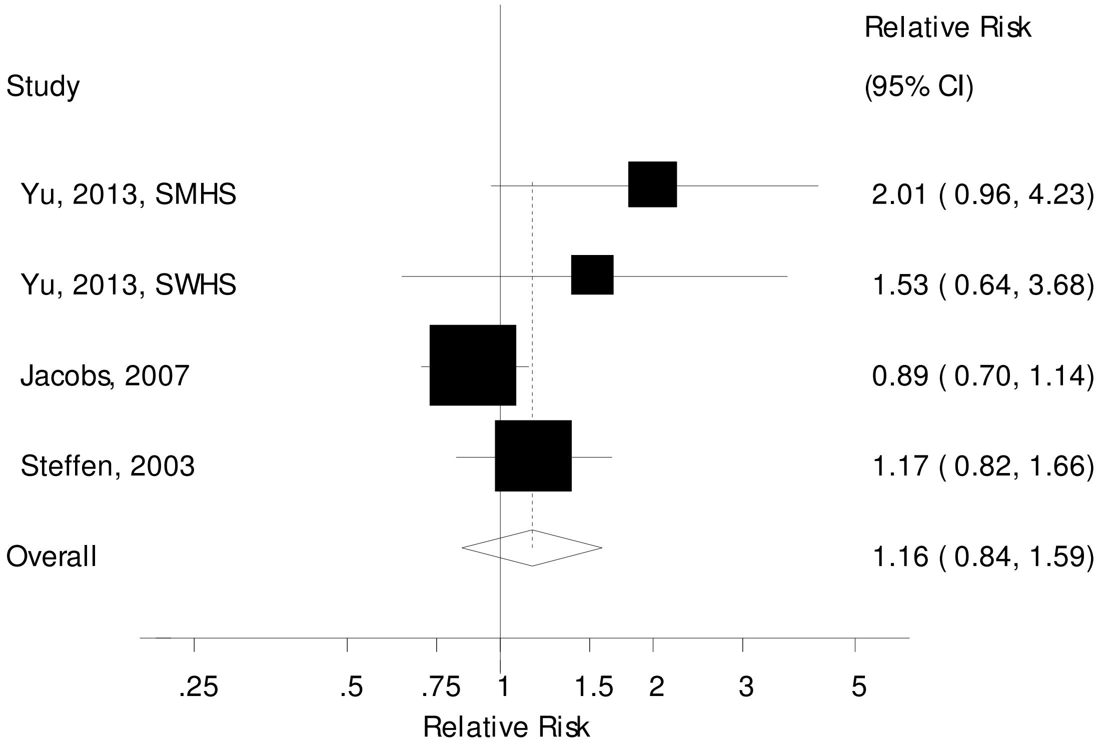

Figure S34. Refined grains and coronary heart disease, per 90 g/d

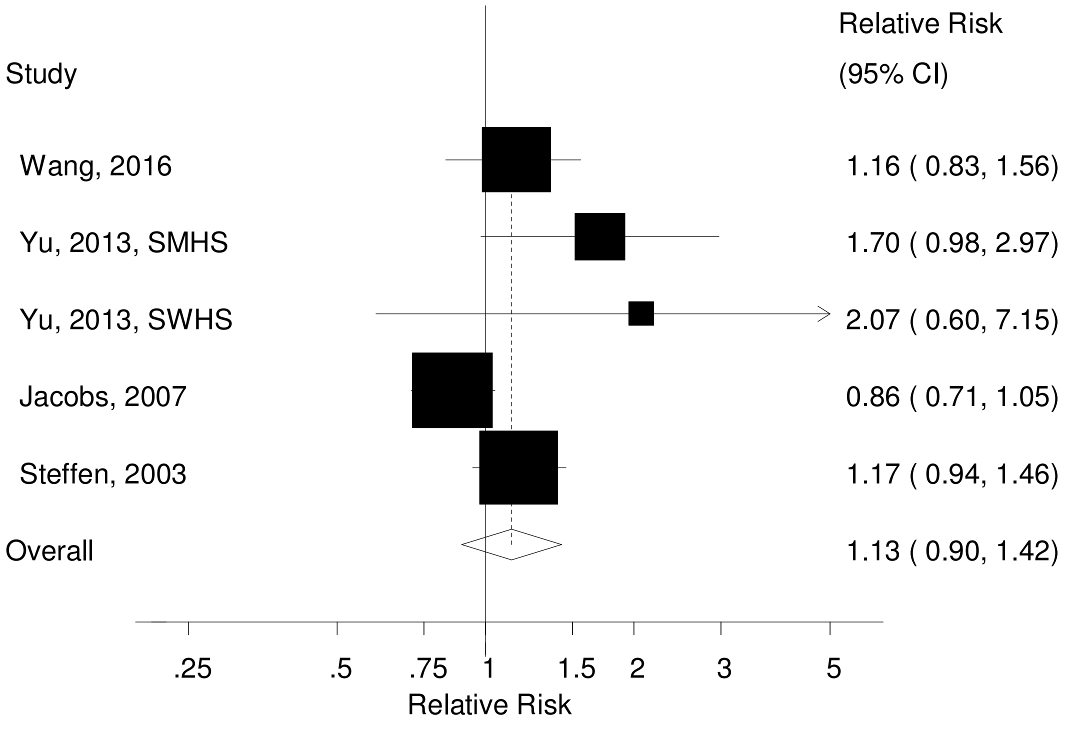

Figure S35. Refined grains and coronary heart disease, nonlinear dose-response

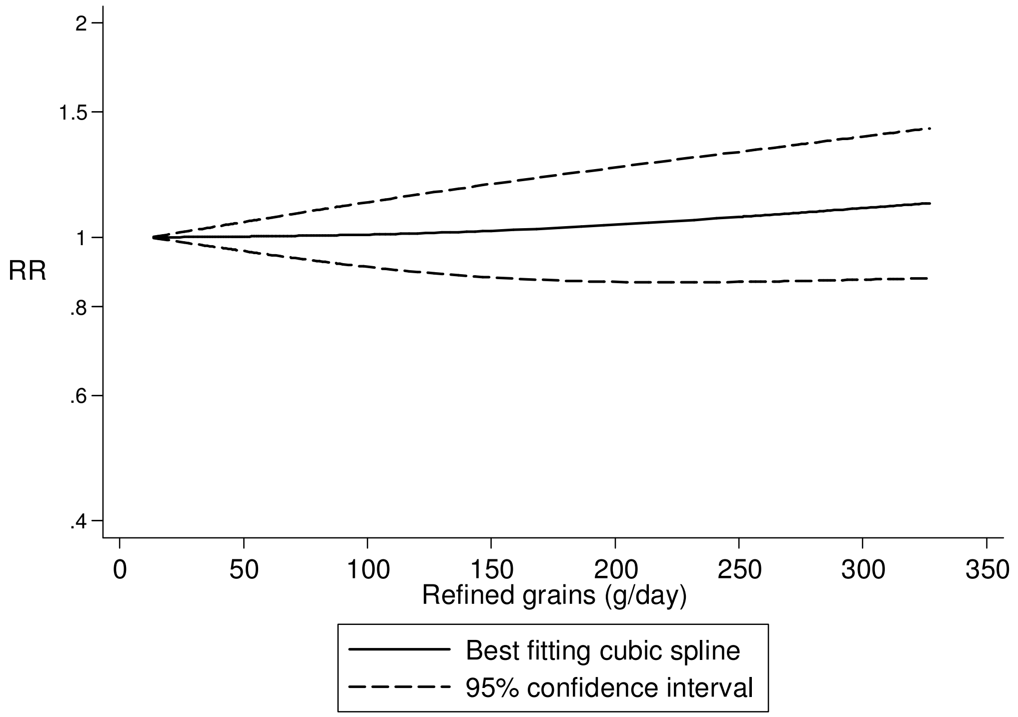

Figure S36. White bread and coronary heart disease, high vs. low

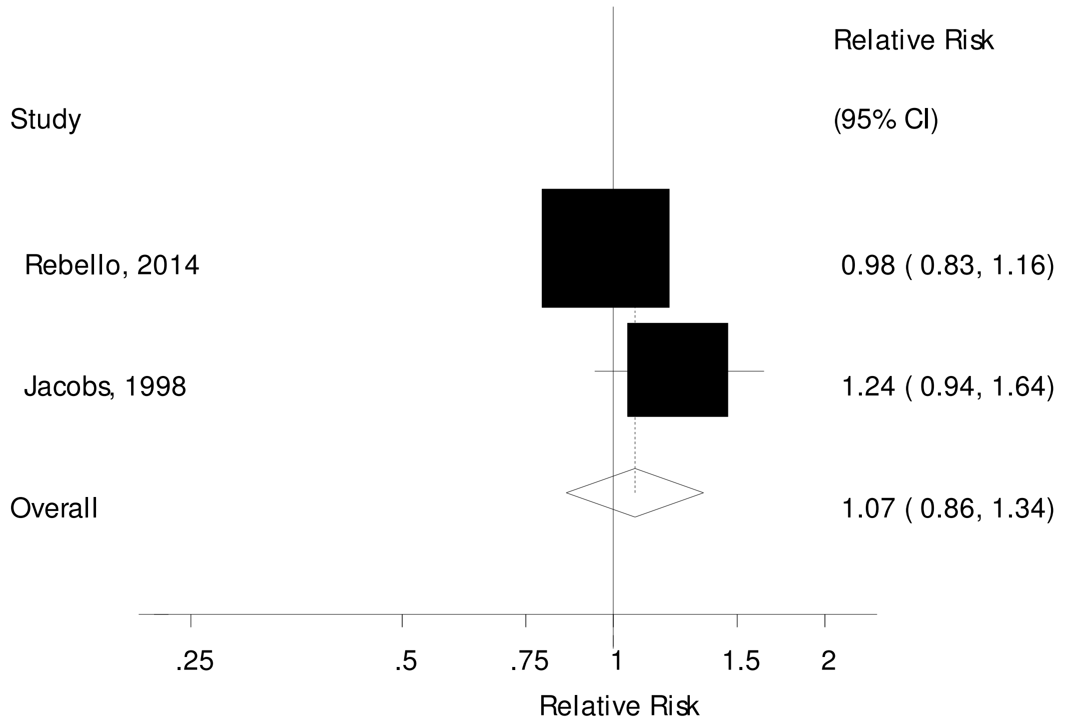

Figure S37. White bread and coronary heart disease, per 90 g/d

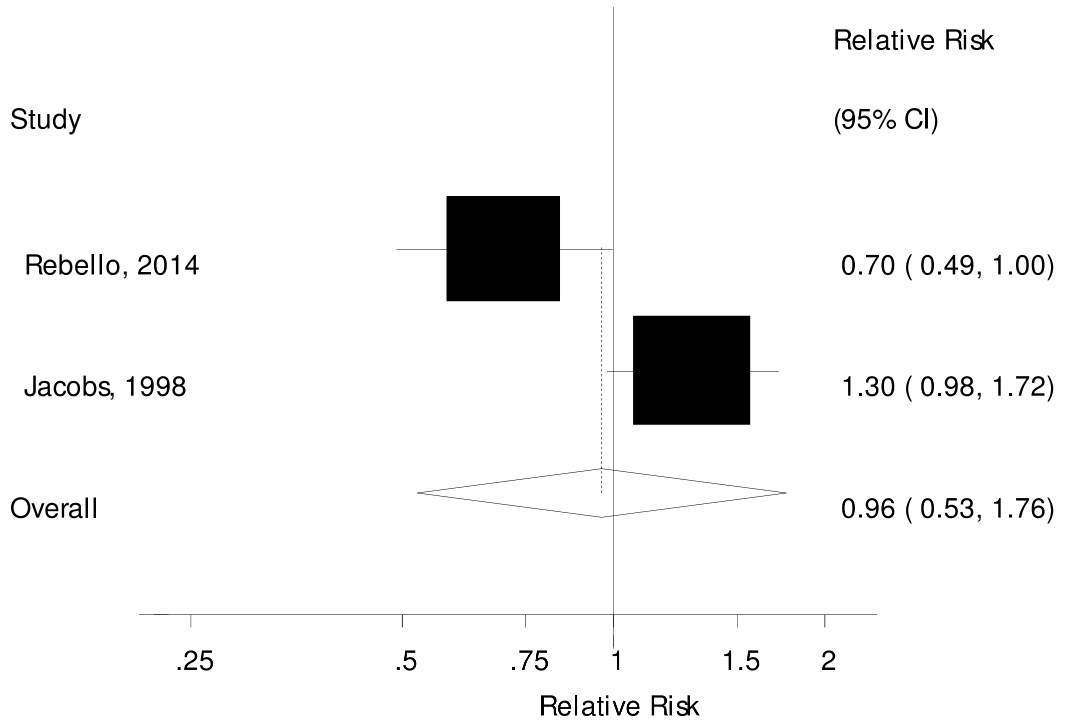

Figure S38. Refined grain breakfast cereals and coronary heart disease, high vs. low

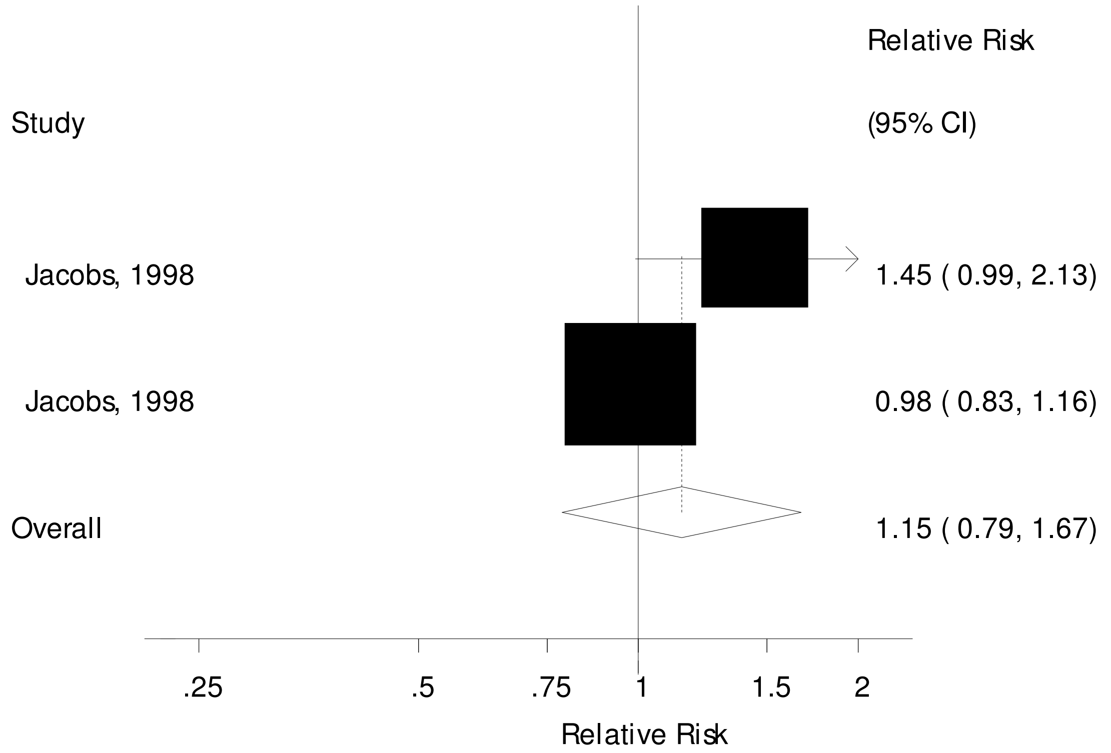

Figure S39. Refined grain breakfast cereals and coronary heart disease, per 90 g/d

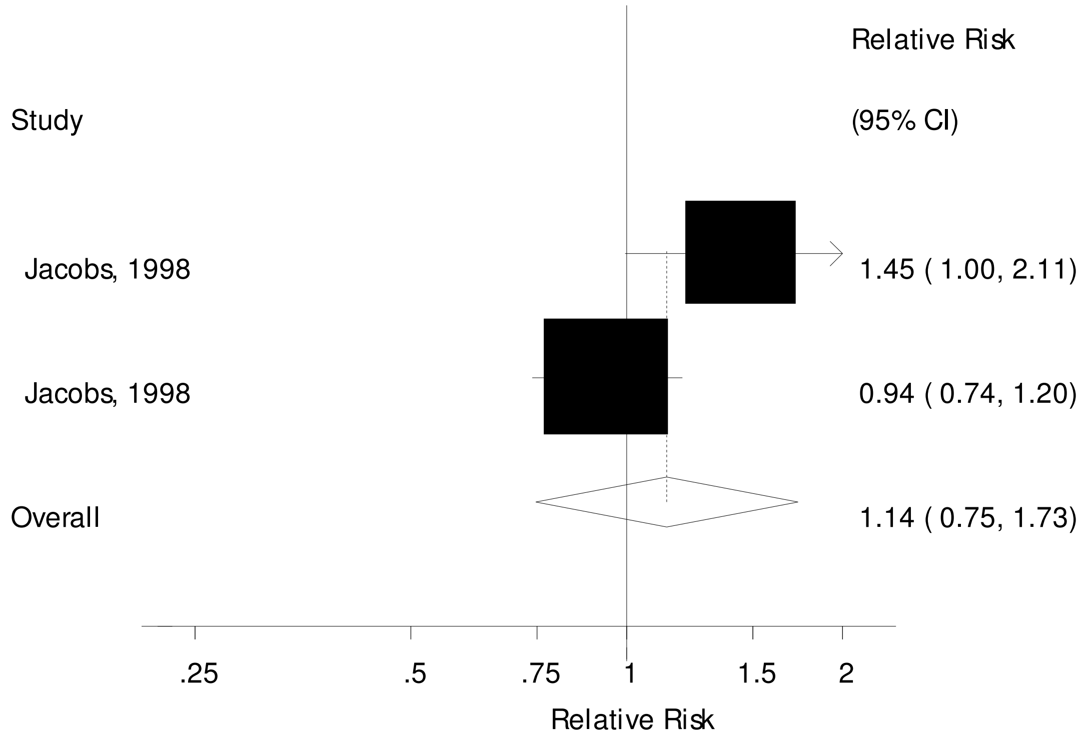

Figure S40. Total rice and coronary heart disease, high vs. low

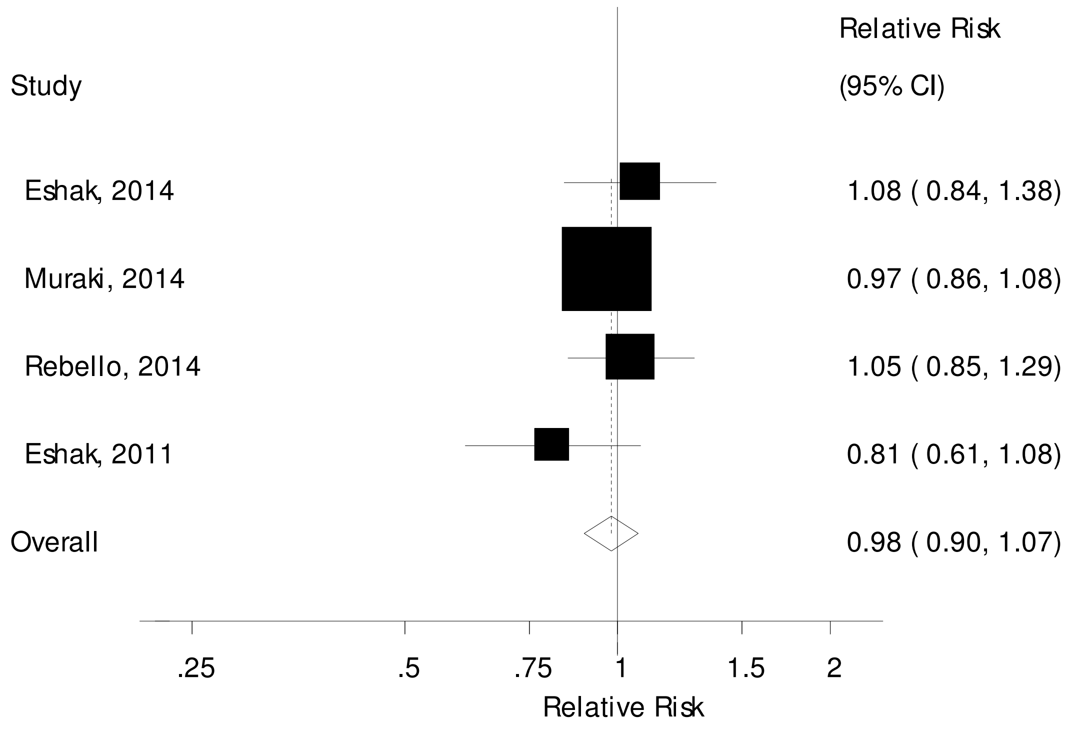

Figure S41. Total rice and coronary heart disease, per 100 g/d

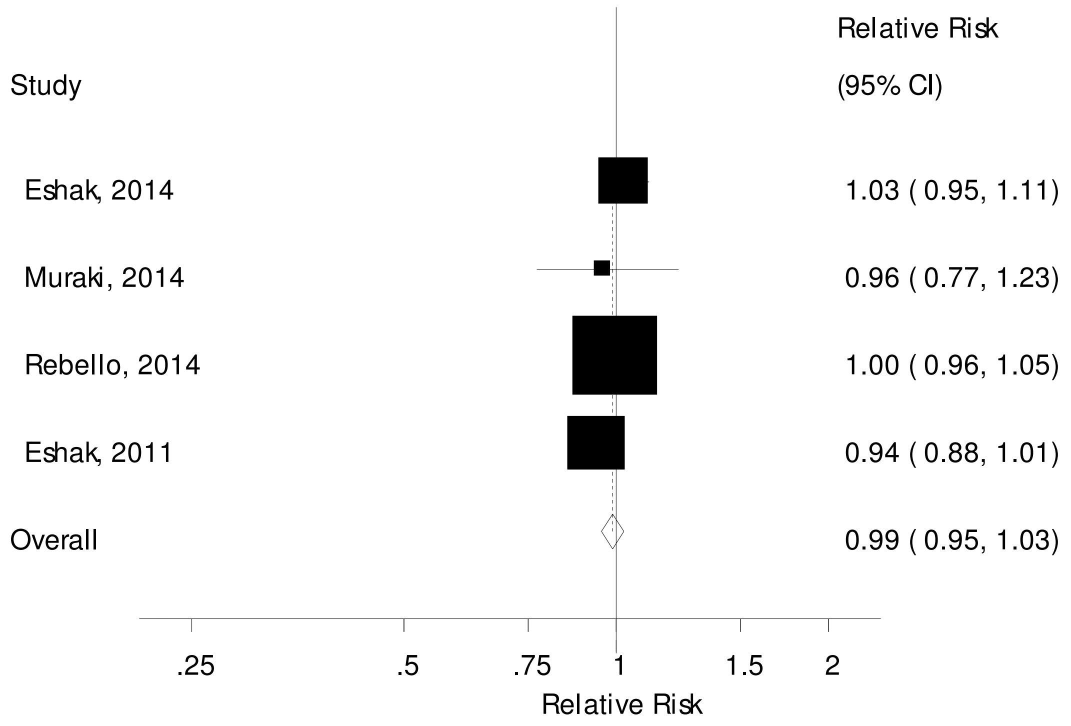

Figure S42. Total rice and coronary heart disease, nonlinear dose-response

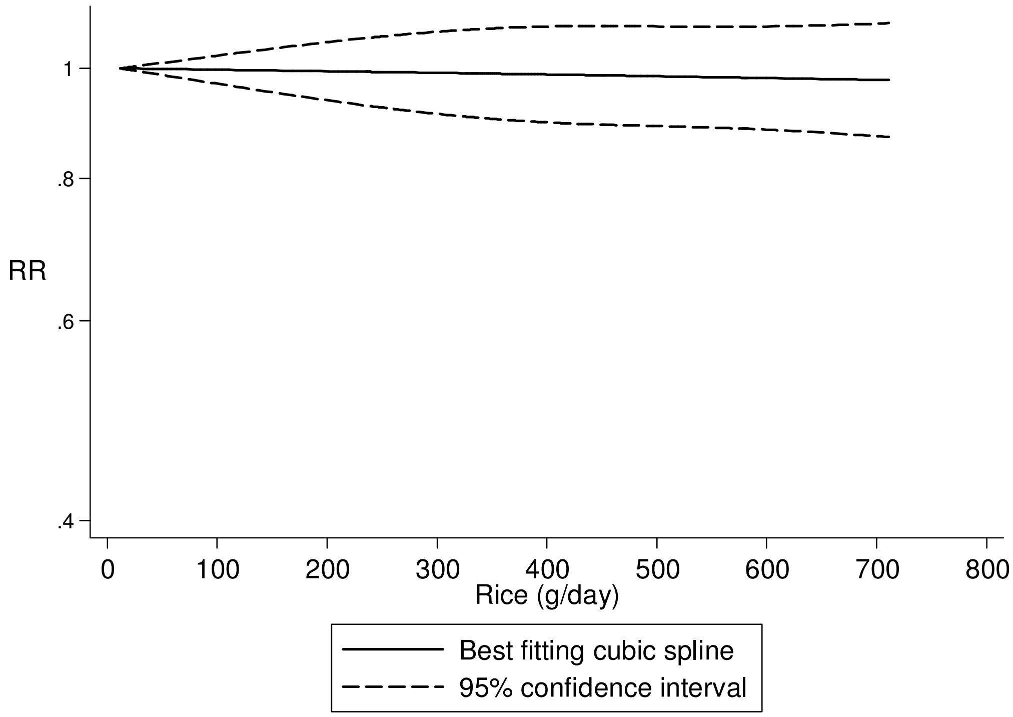

Figure S43. Total grains and coronary heart disease, high vs. low

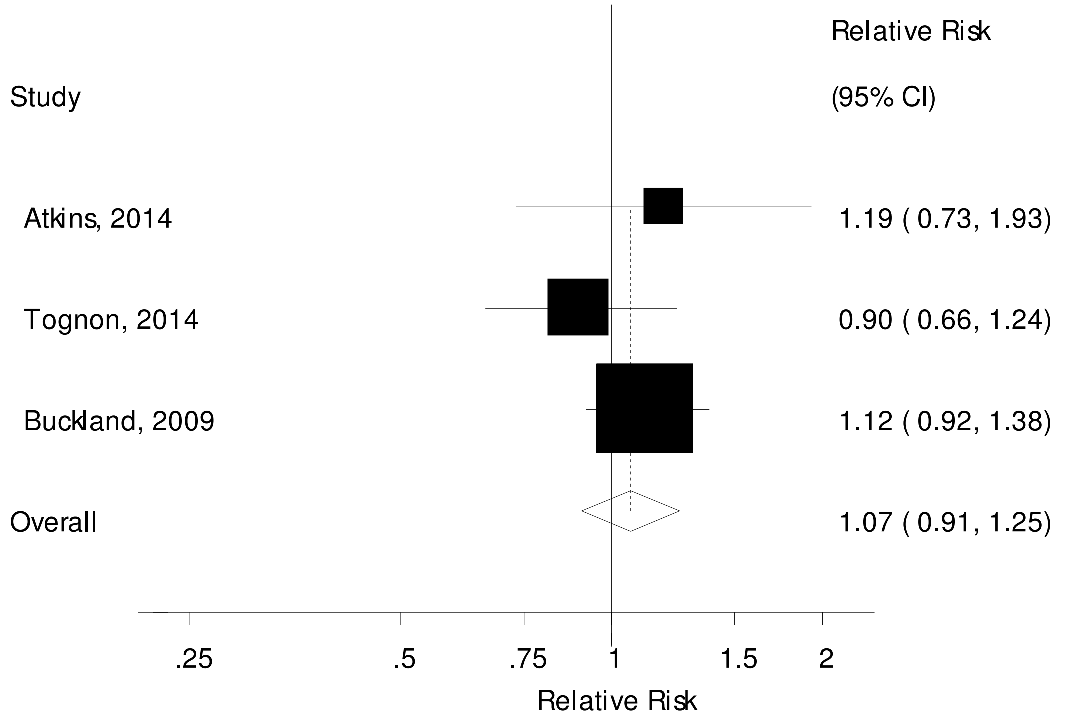

Figure S44. Total grains and coronary heart disease, per 90 g/d

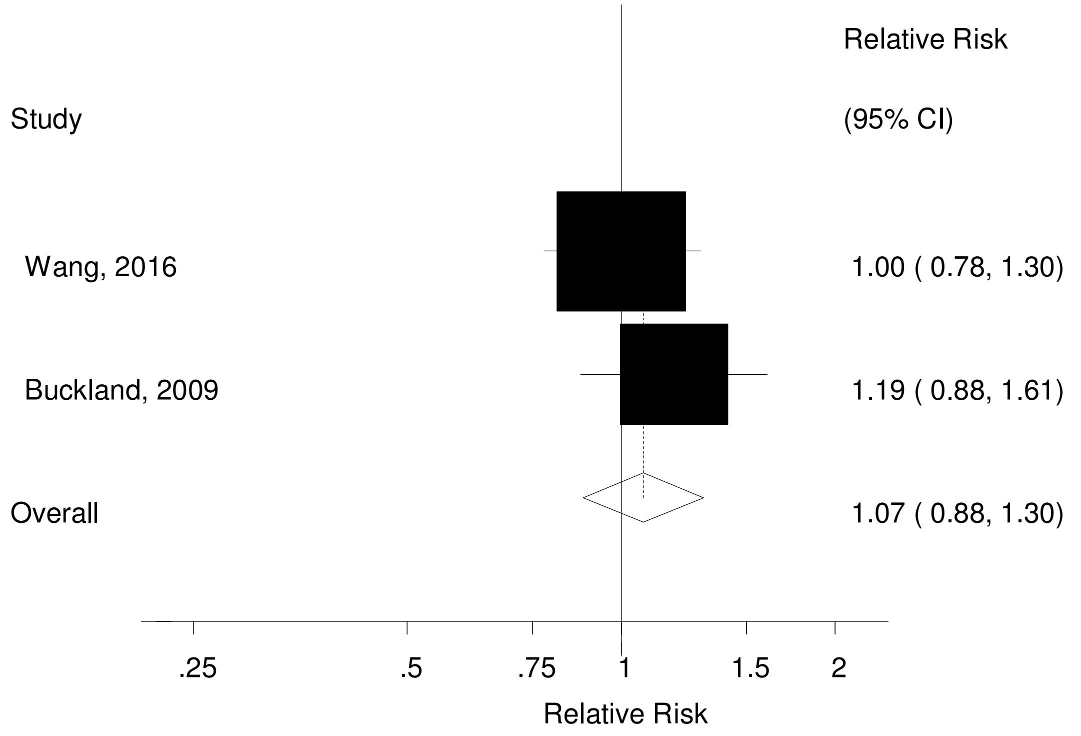

Figure S45. Whole grain bread and stroke, high vs. low

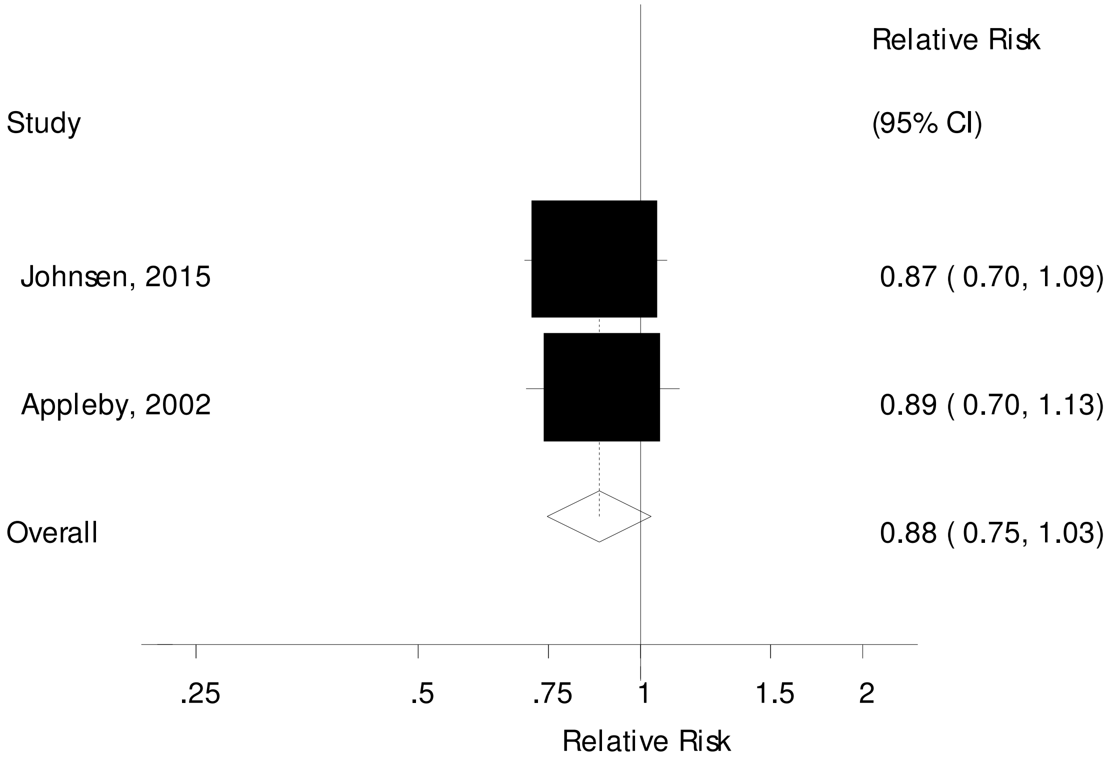

Figure S46. Whole grain breakfast cereals and stroke, high vs. low

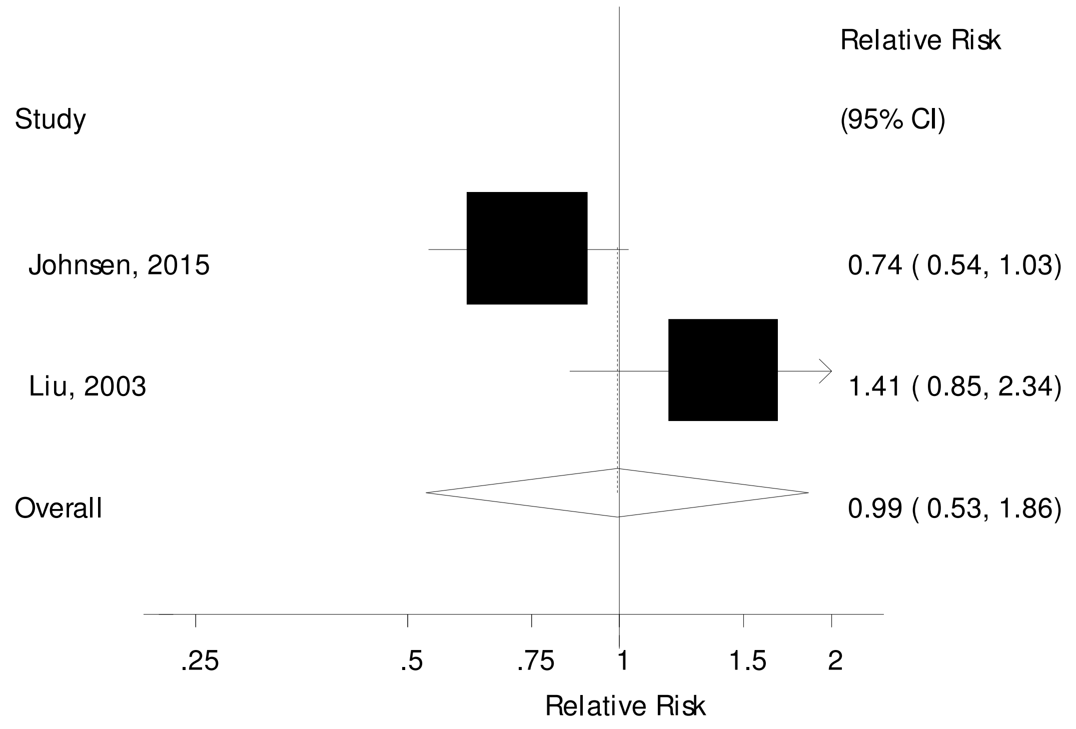

Figure S47. Whole grain breakfast cereals and stroke, per 90 g/d

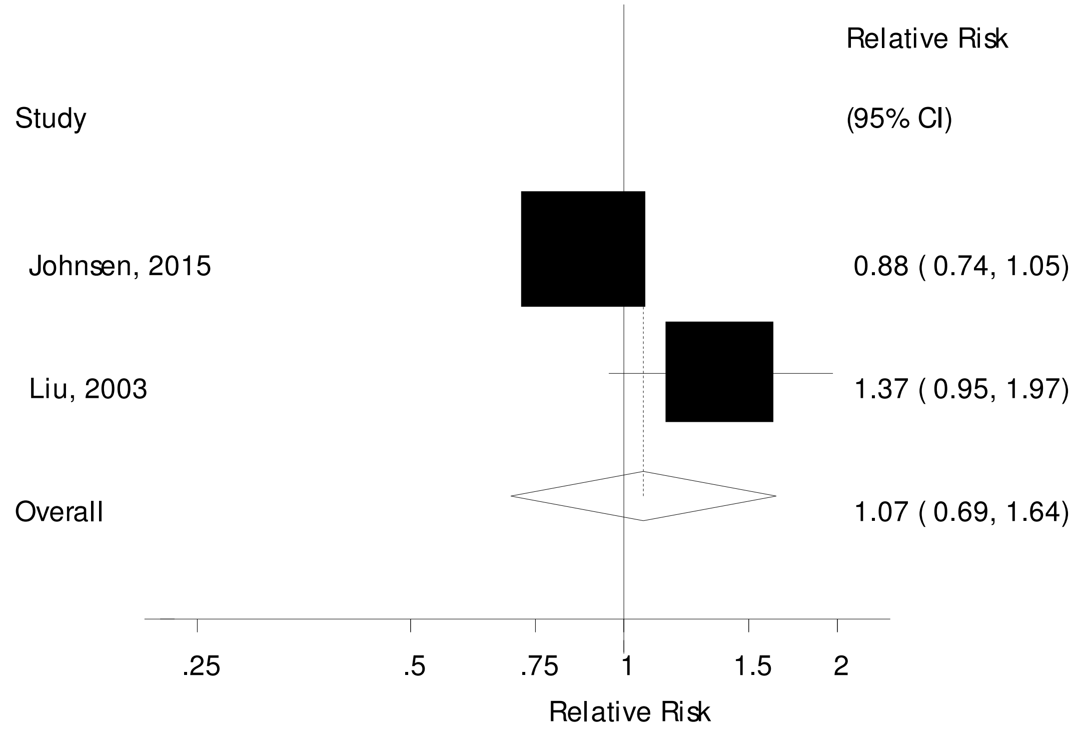

Figure S48. Refined grains and stroke, high vs. low

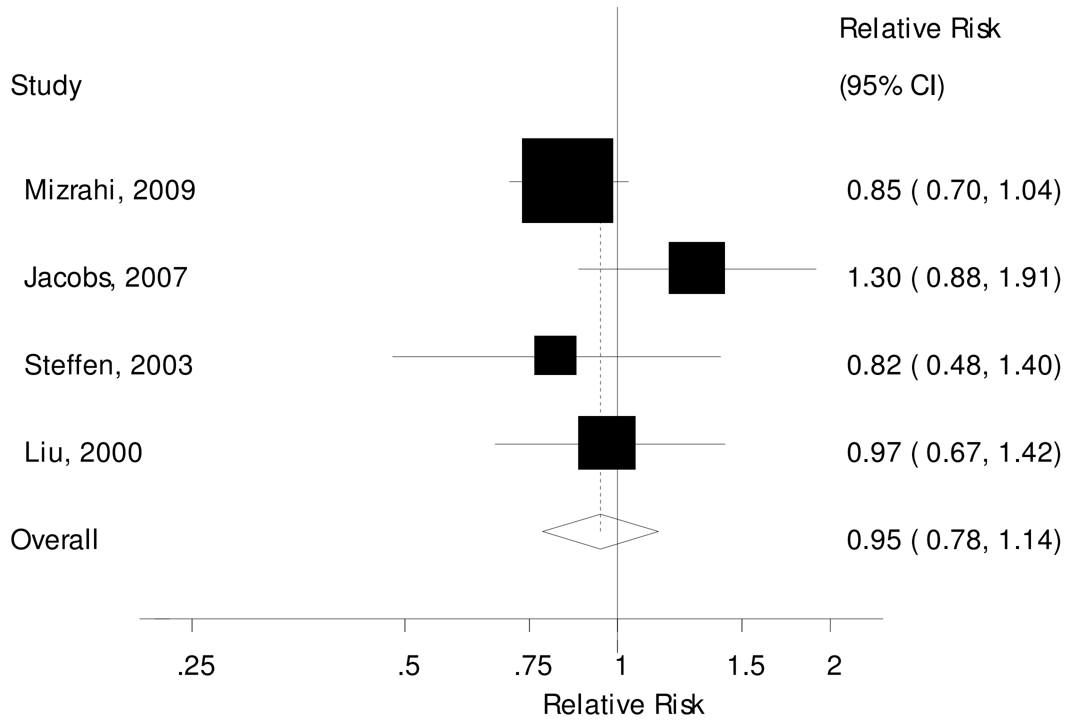

Figure S49. Refined grains and stroke, per 90 g/d

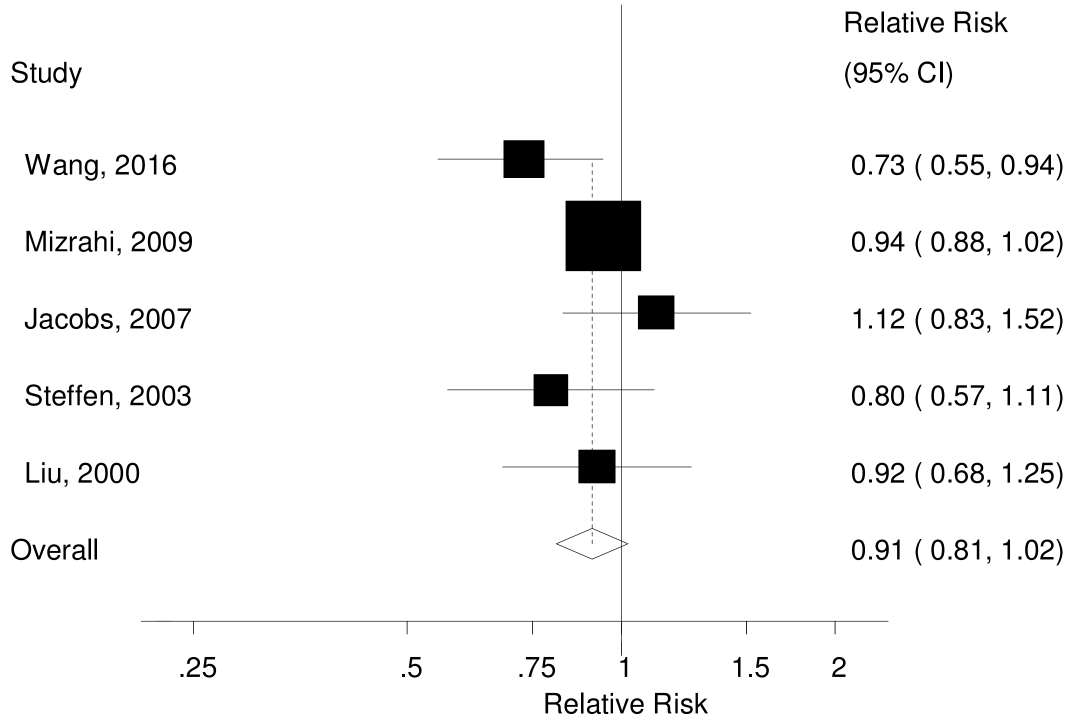

Figure S50. Refined grains and stroke, nonlinear dose-response

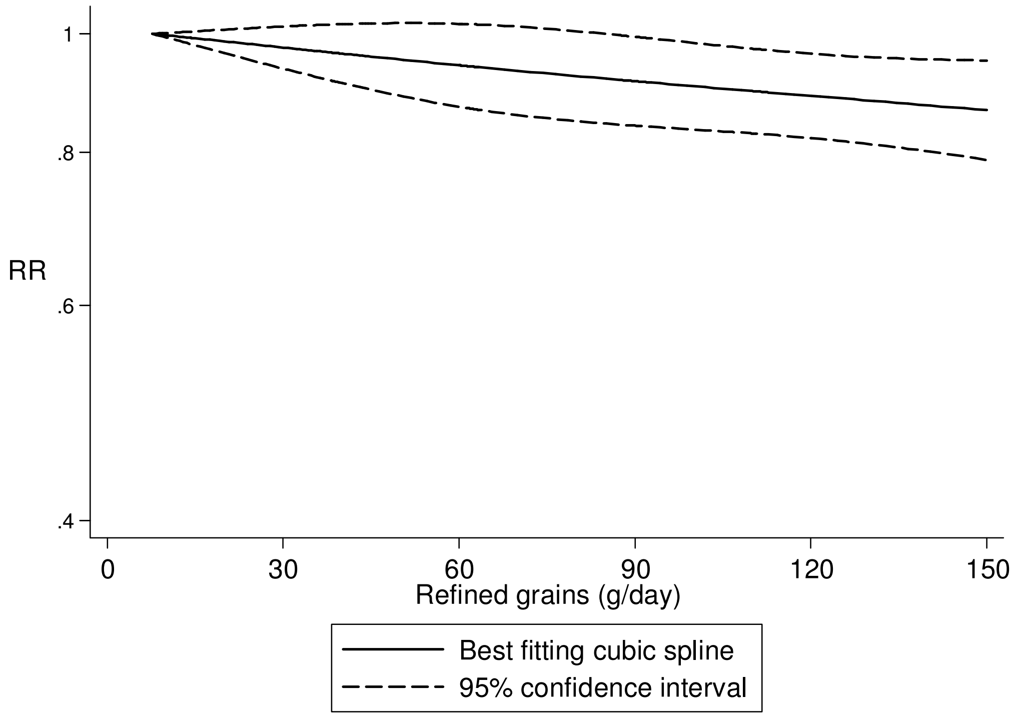

Figure S51. Total rice and stroke, high vs. low

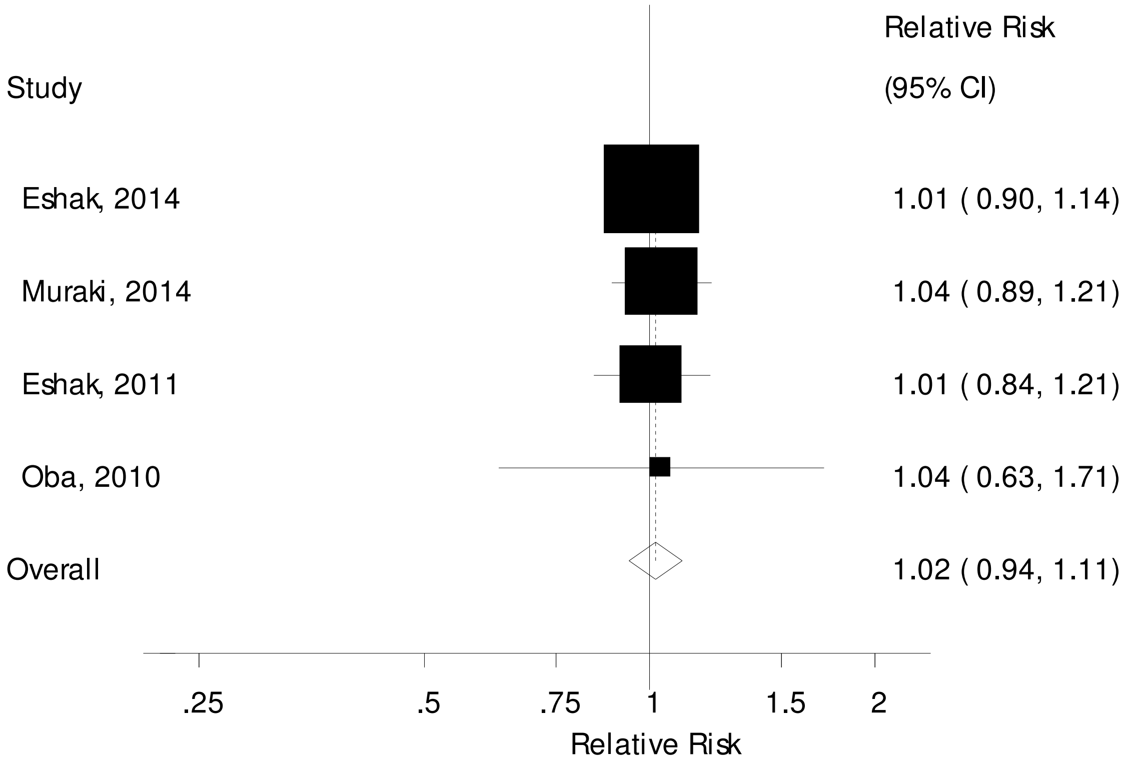

Figure S52. Total rice and stroke, per 100 g/d

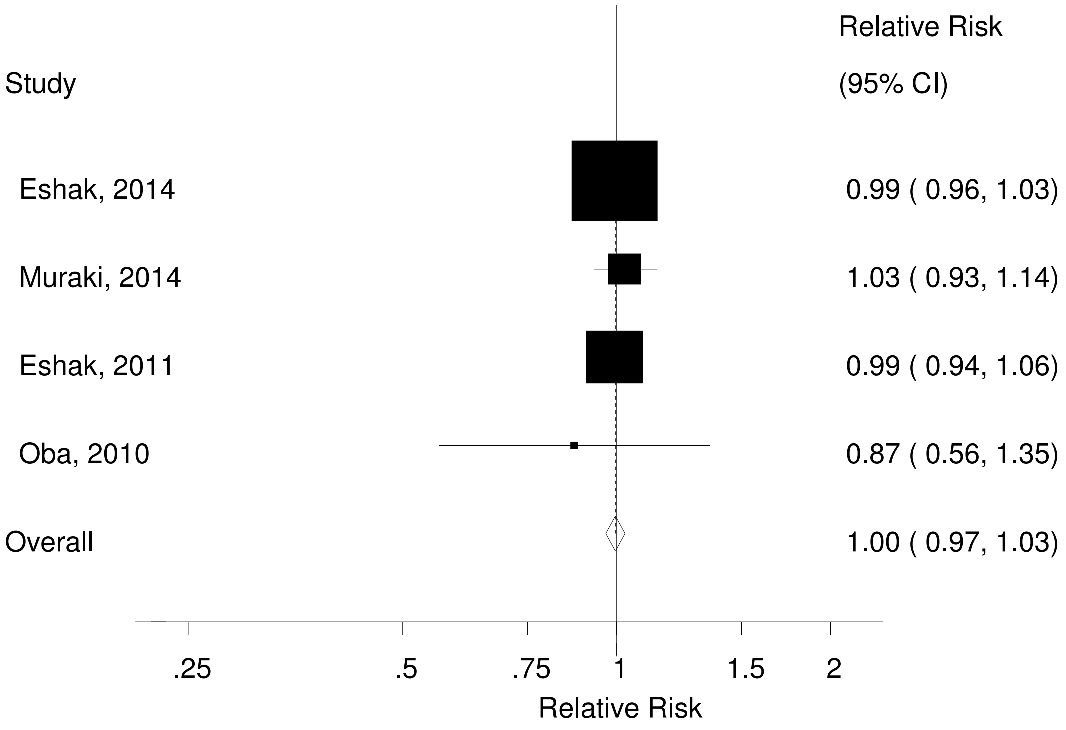

Figure S53. Total rice and stroke, nonlinear dose-response

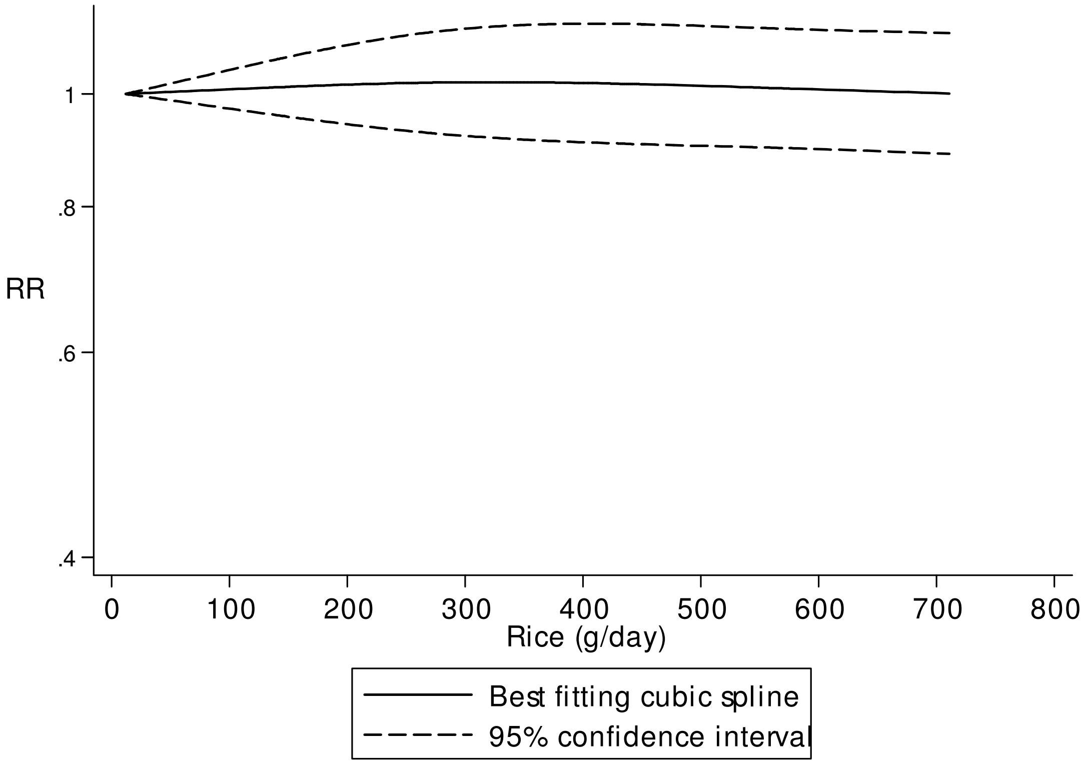

Figure S54. Total grains and stroke, high vs. low

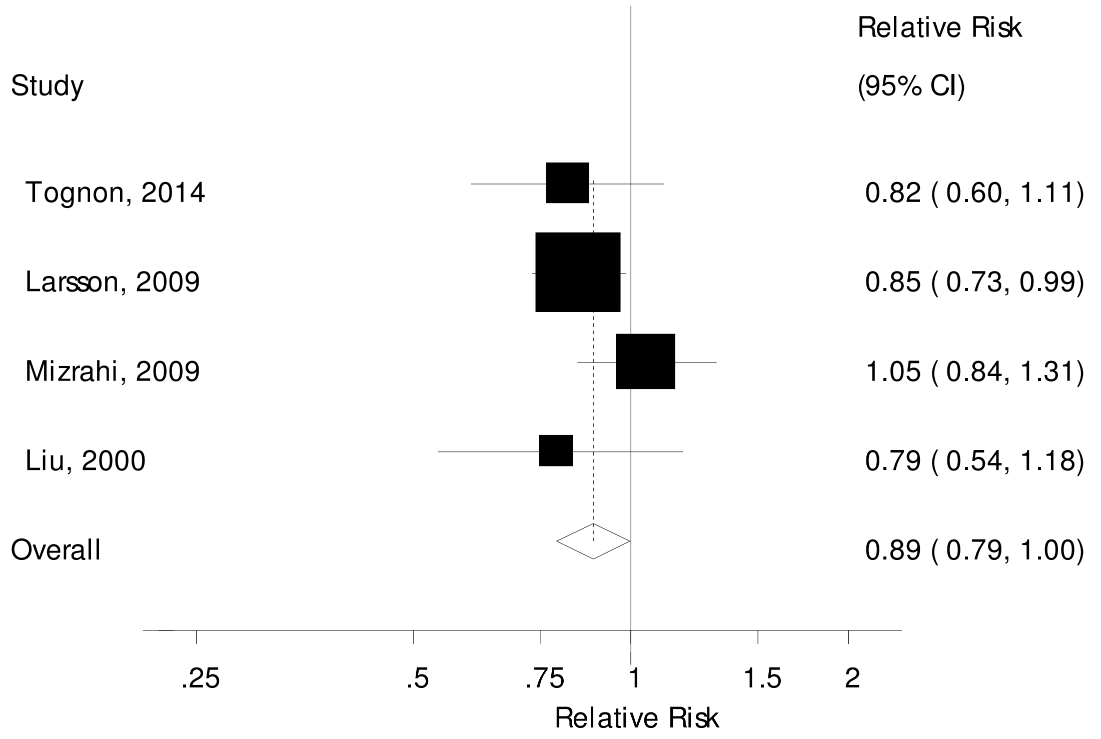

Figure S55. Total grains and stroke, per 90 g/d

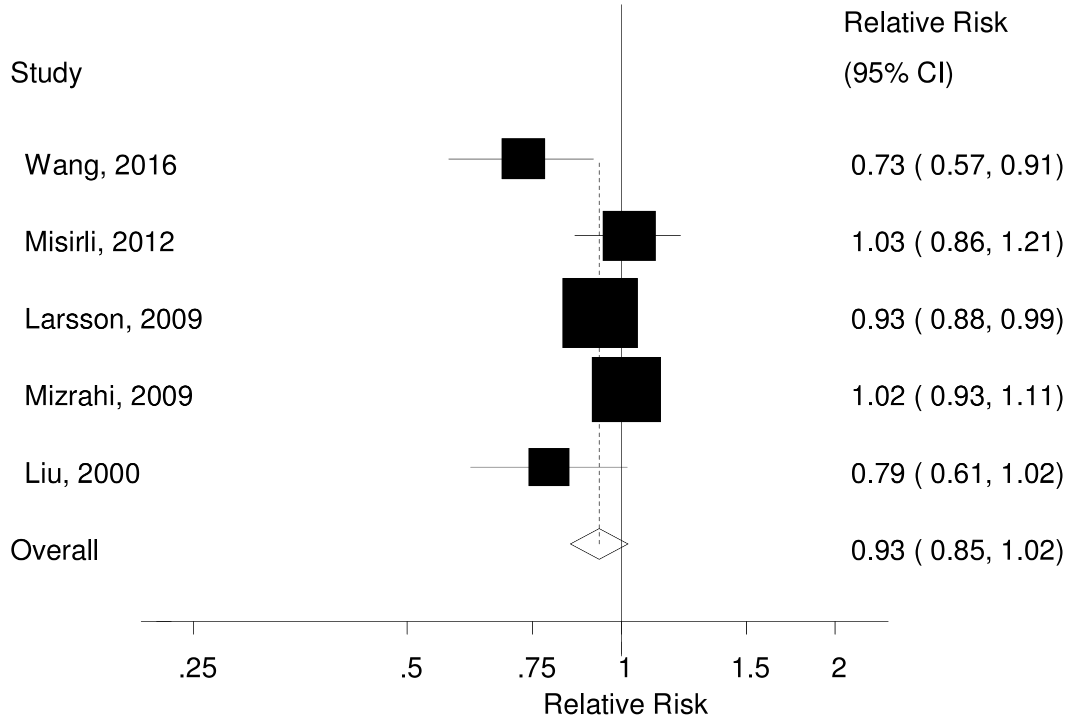

Figure S56. Total grains and stroke, nonlinear dose-response

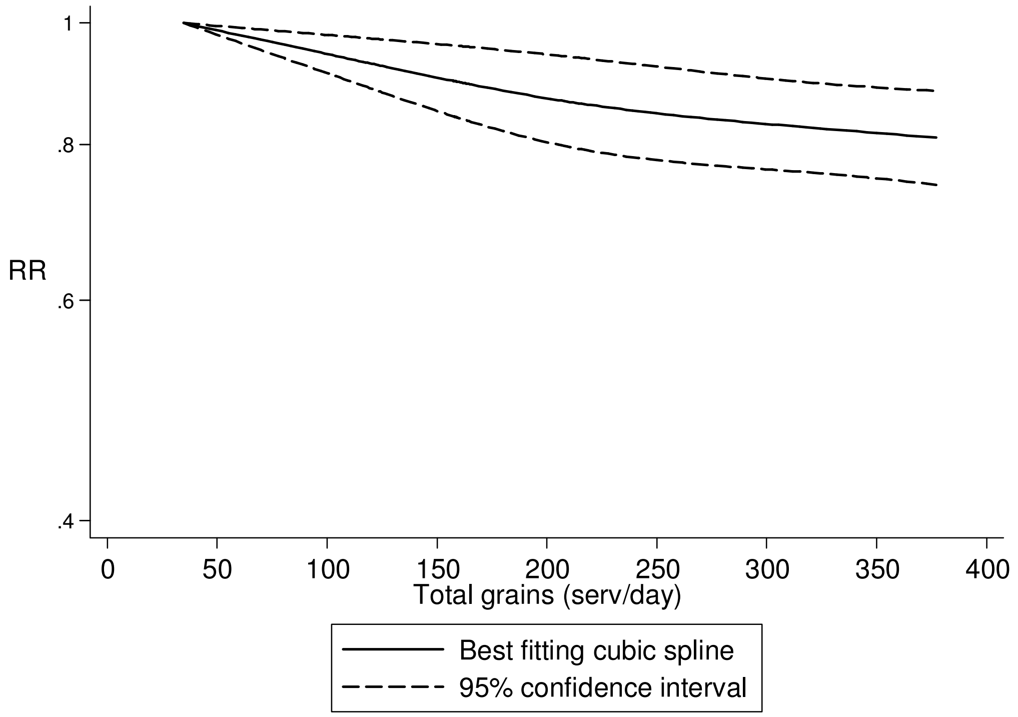

Figure S57. Whole grain bread and cardiovascular disease, high vs. low

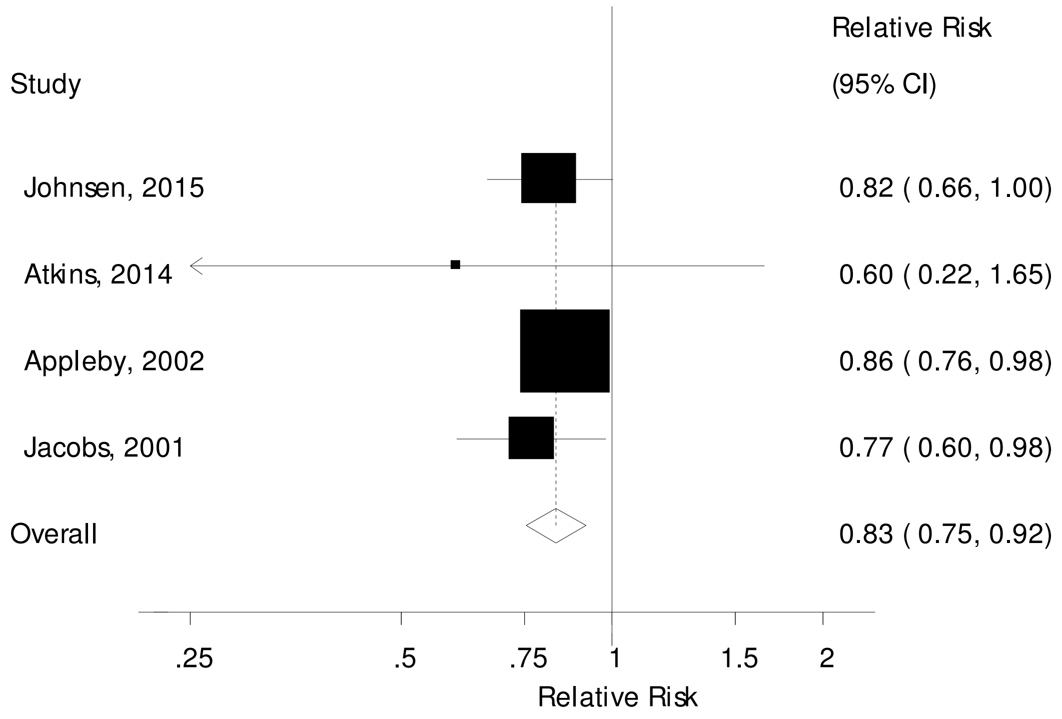

Figure S58. Whole grain bread and cardiovascular disease, per 90 g/d

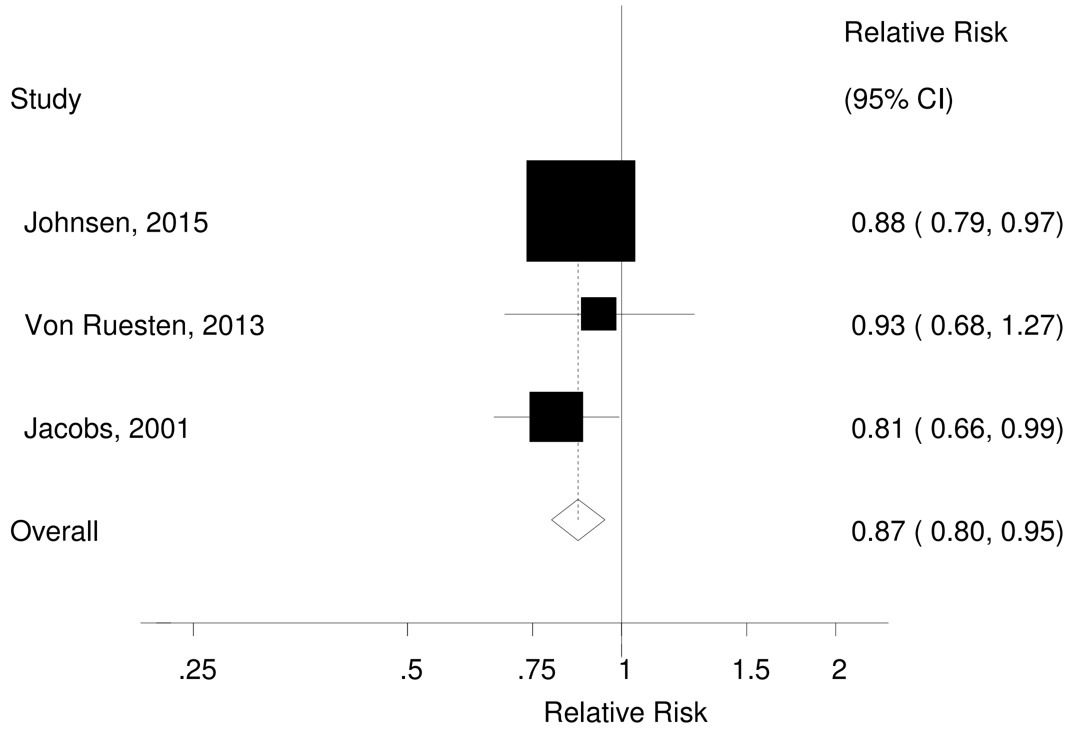

Figure S59. Whole grain breakfast cereals and cardiovascular disease, high vs. low

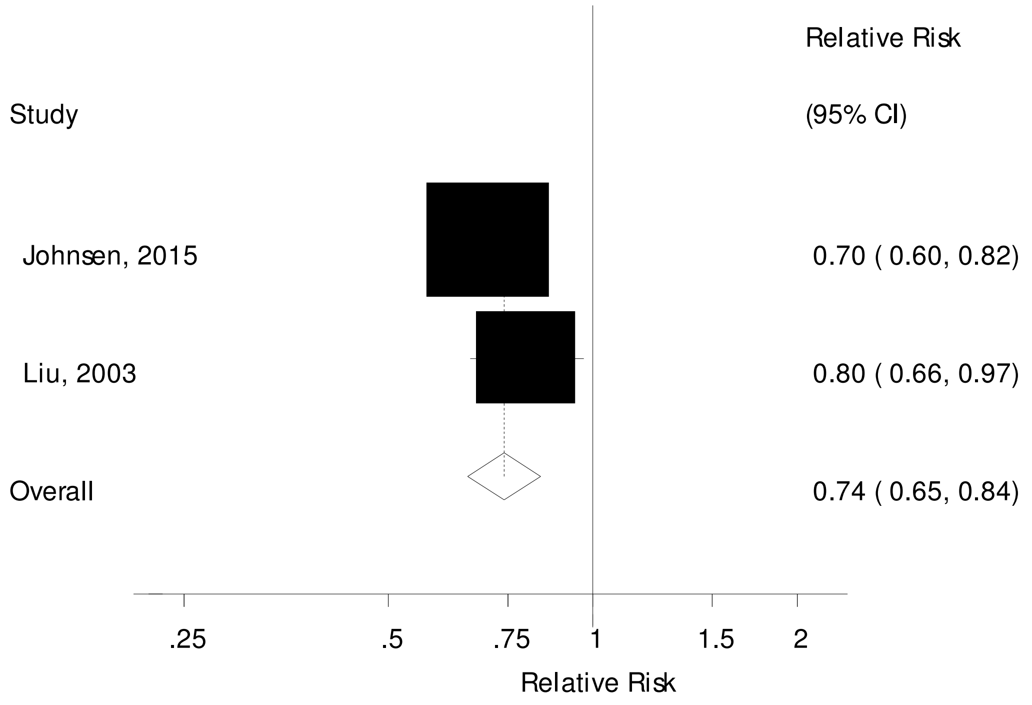

Figure S60. Whole grain breakfast cereals and cardiovascular disease, per 30 g/d

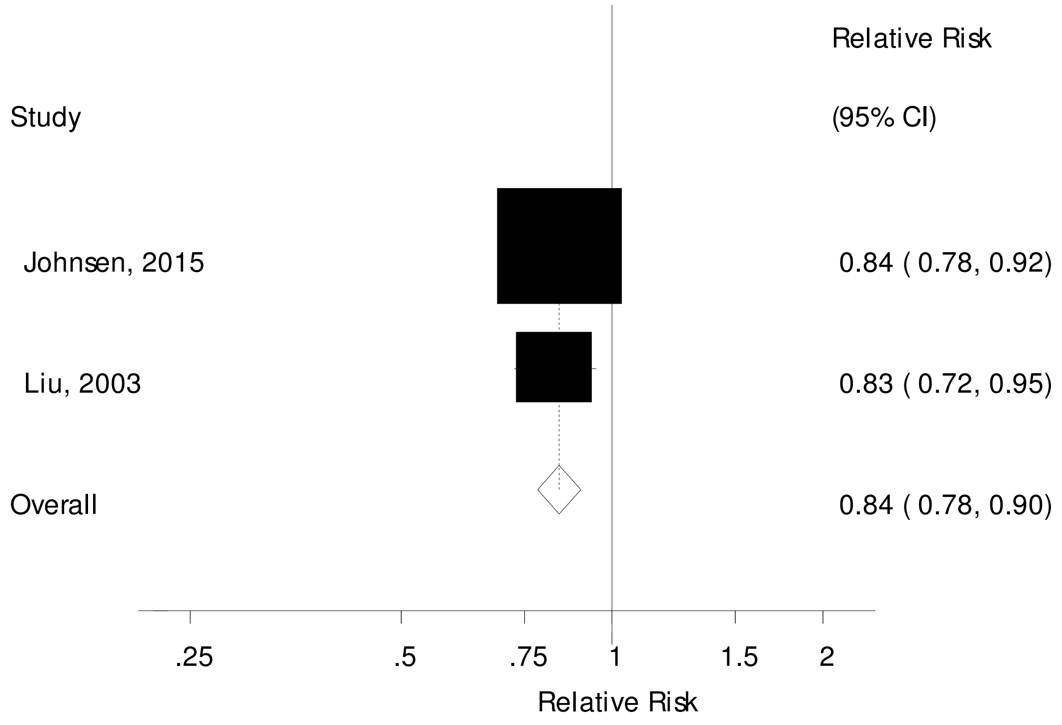

Figure S61. Bran and cardiovascular disease, high vs. low

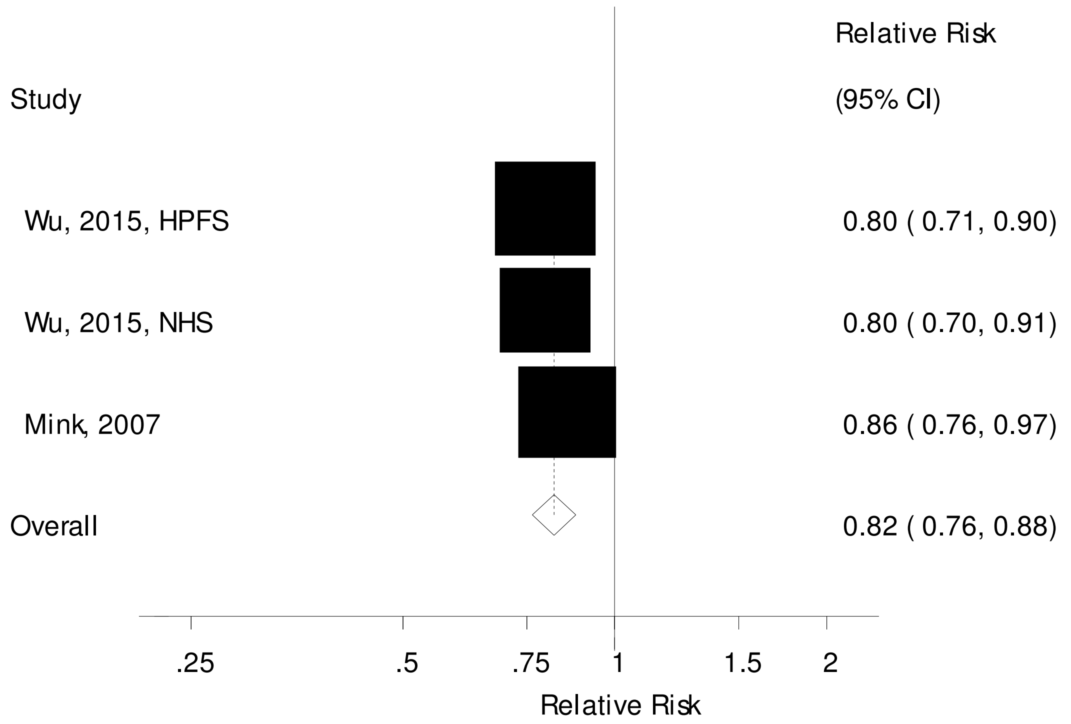

Figure S62. Bran and cardiovascular disease, per 10 g/d

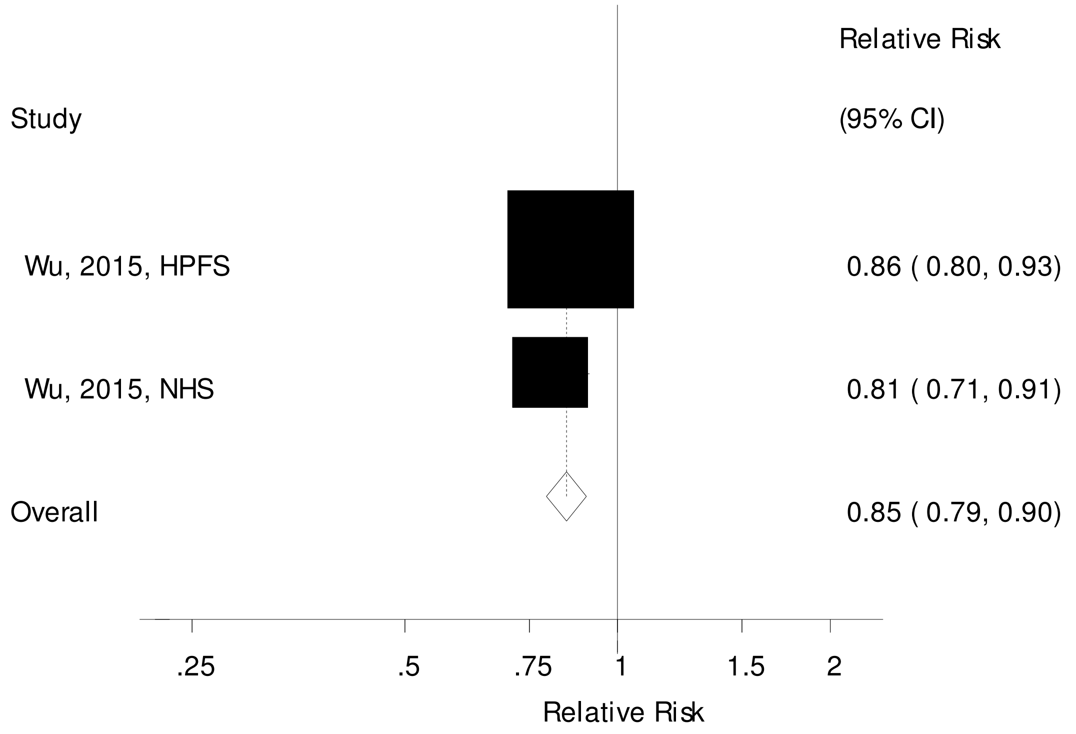

Figure S63. Germ and cardiovascular disease, high vs. low

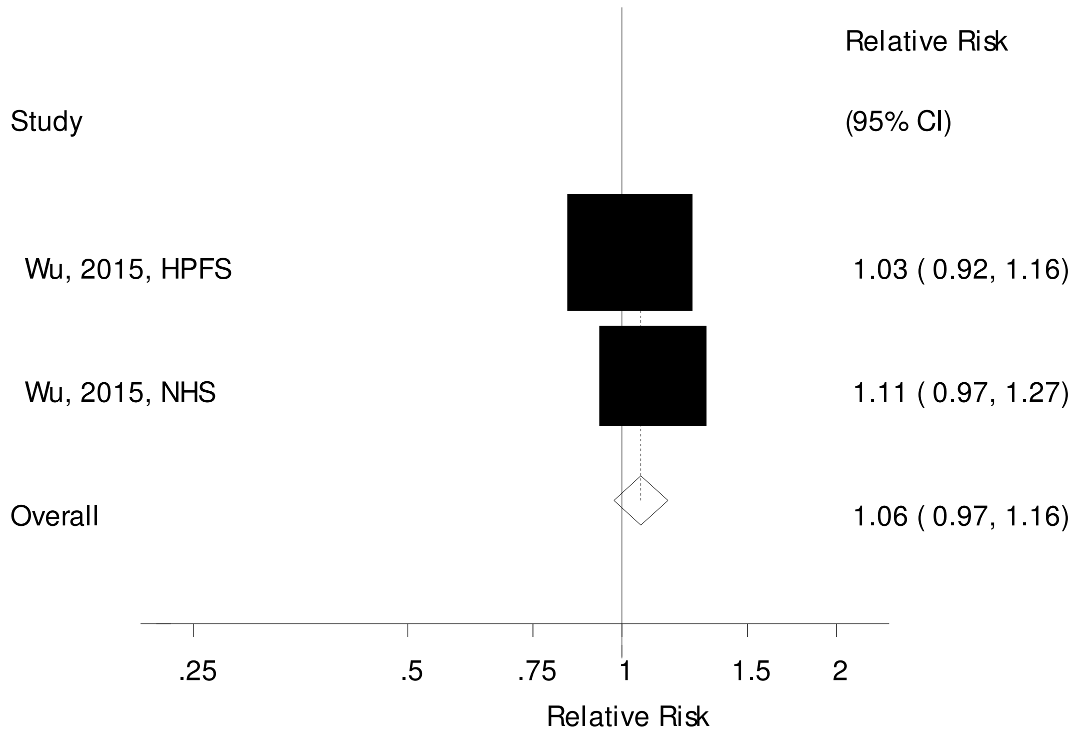

Figure S64. Germ and cardiovascular disease, per 2 g/d

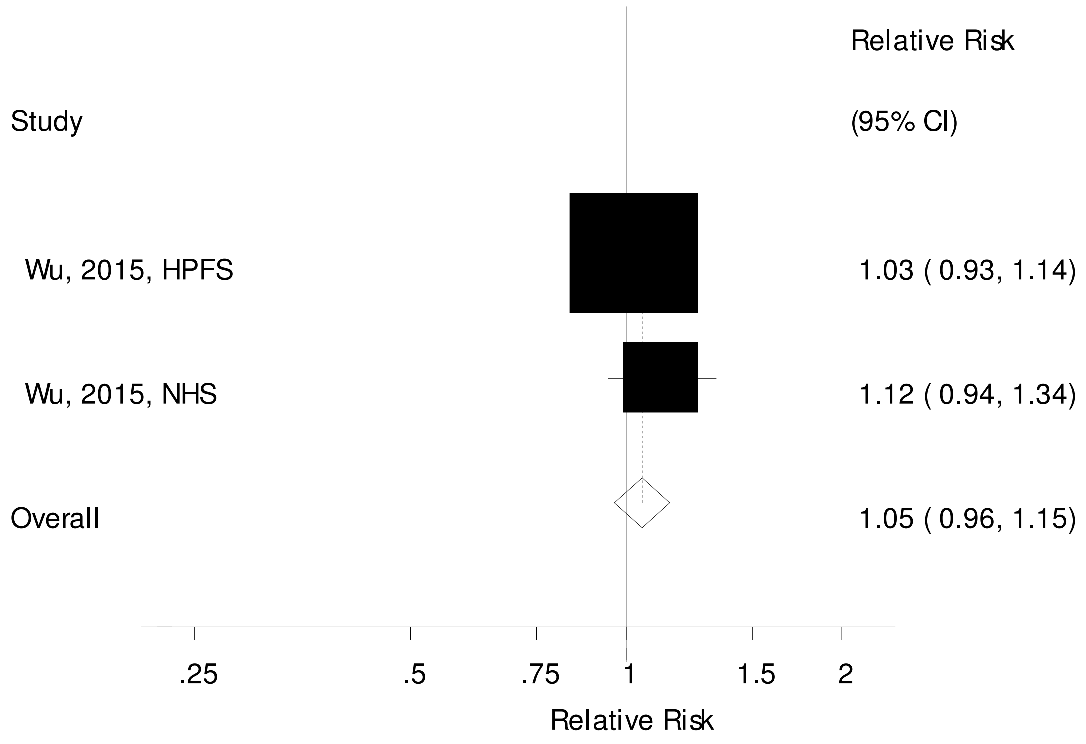

Figure S65. Refined grains and cardiovascular disease, high vs. low

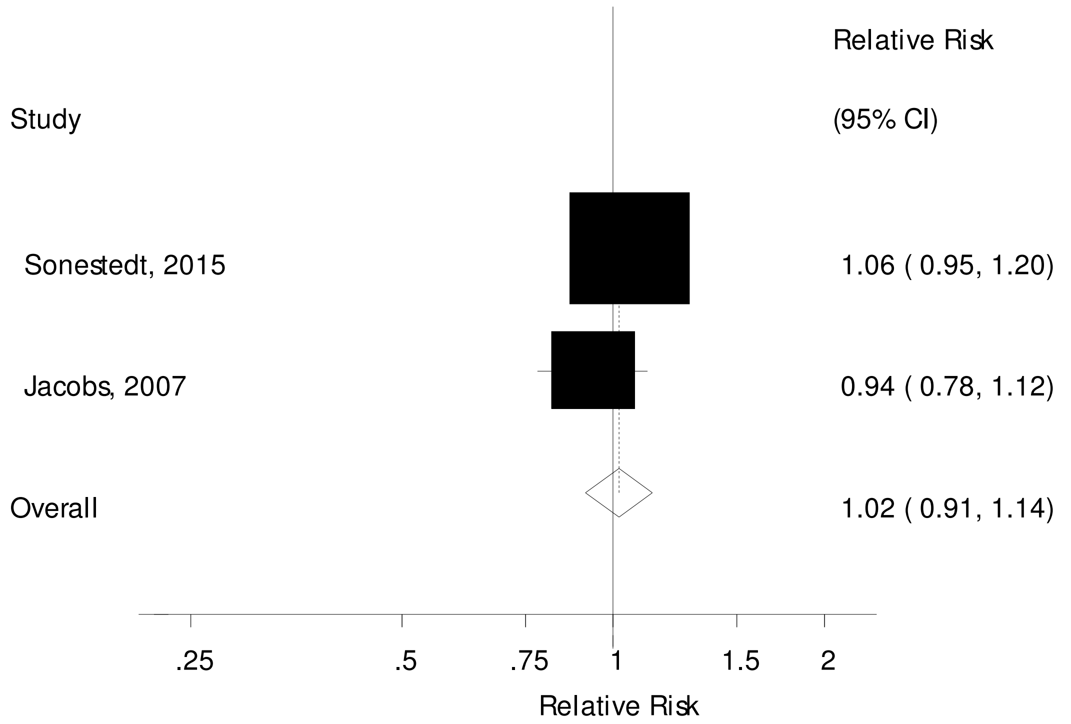

Figure S66. Refined grains and cardiovascular disease, per 90 g/d

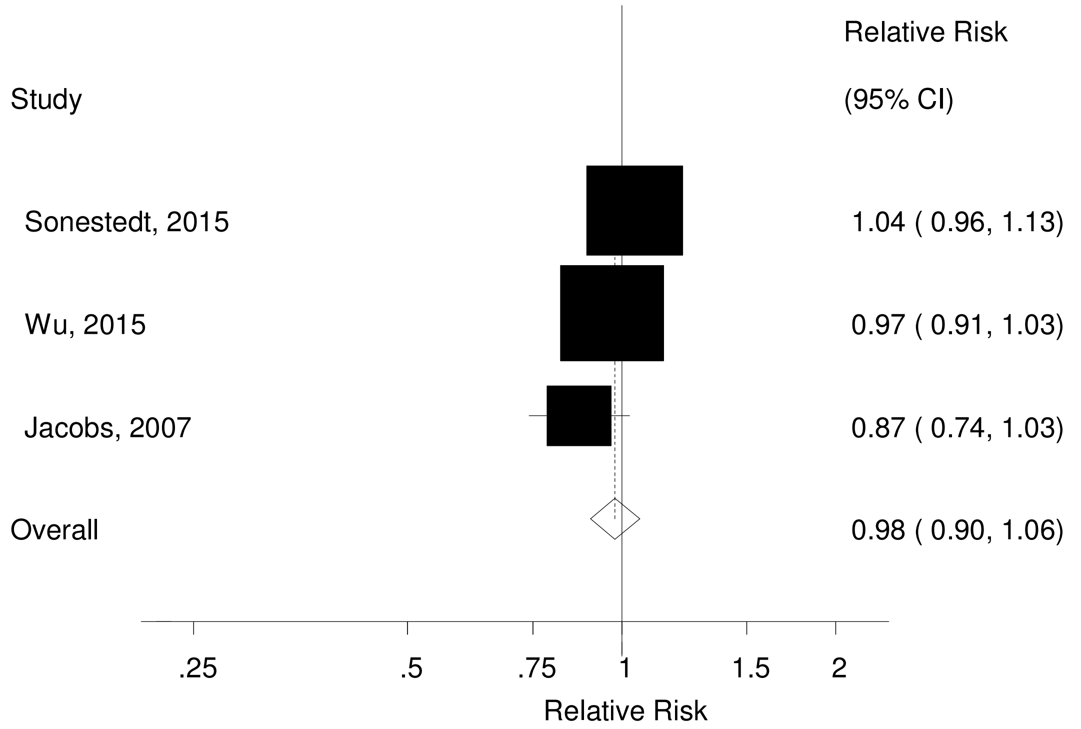

Figure S67. Total breakfast cereals and cardiovascular disease, high vs. low

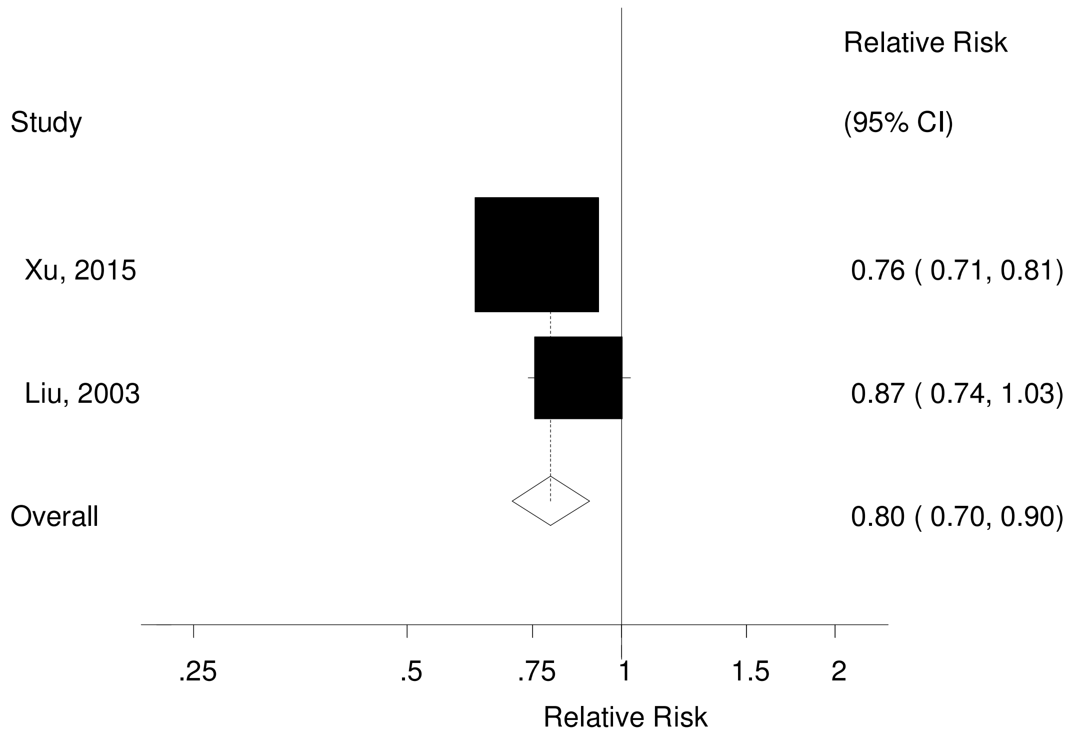

Figure S68. Total breakfast cereals and cardiovascular disease, per 30 g/d

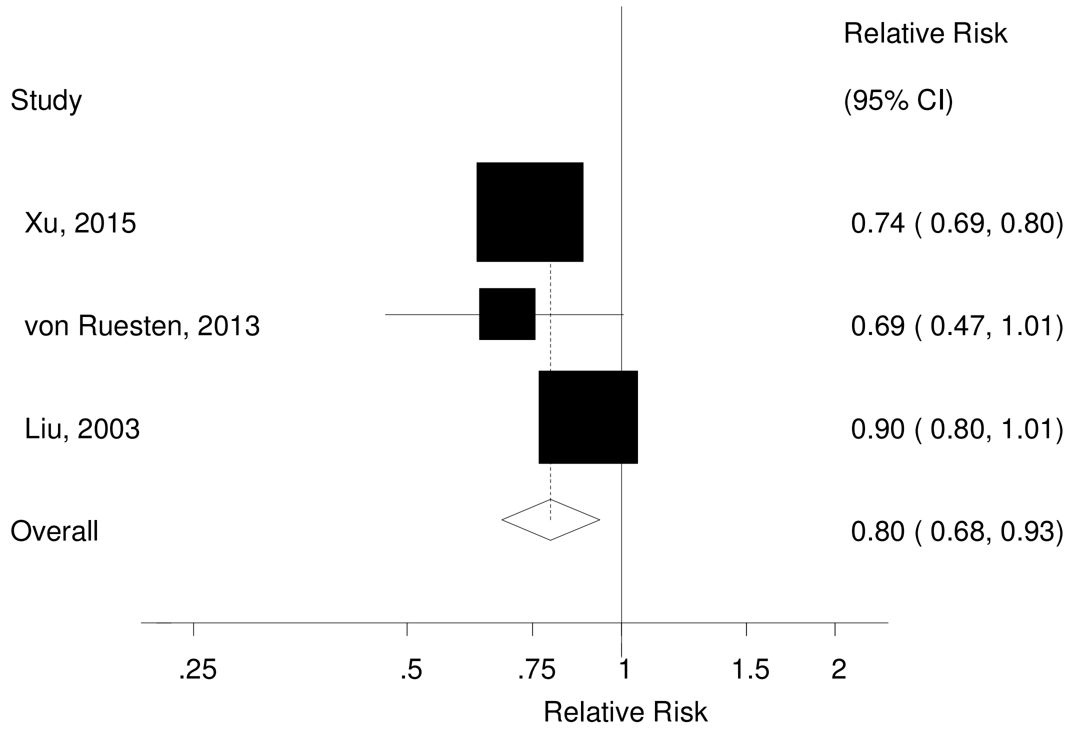

Figure S69. Total rice and cardiovascular disease, high vs. low

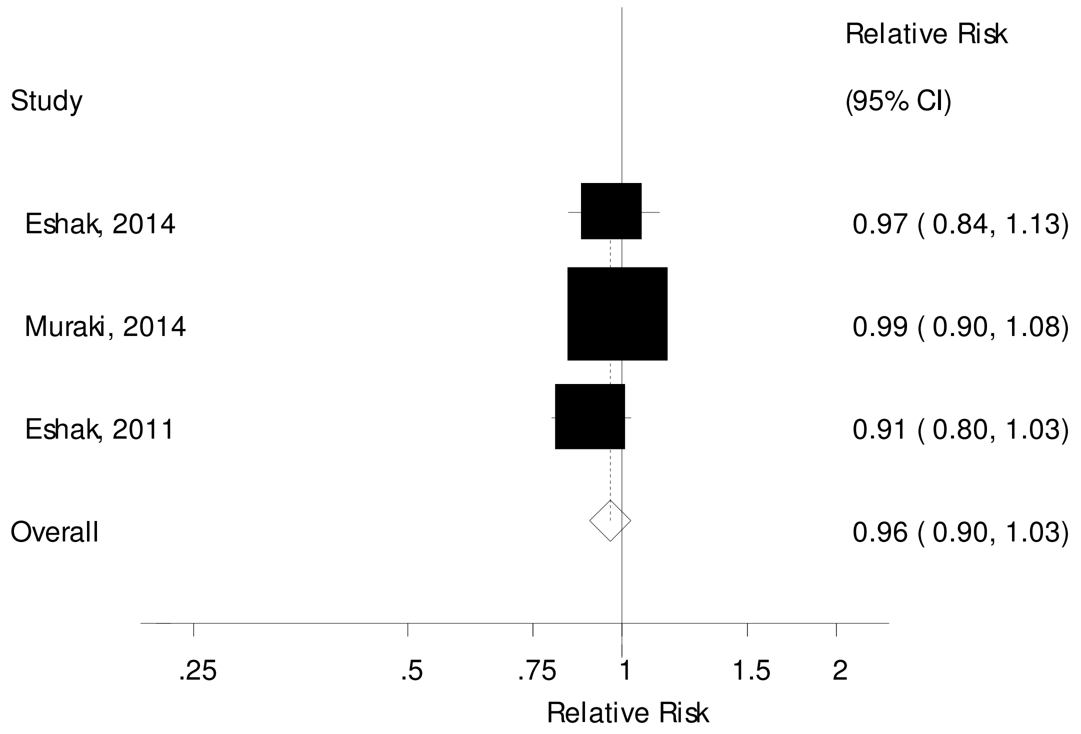

Figure S70. Total rice and cardiovascular disease, per 100 g/d

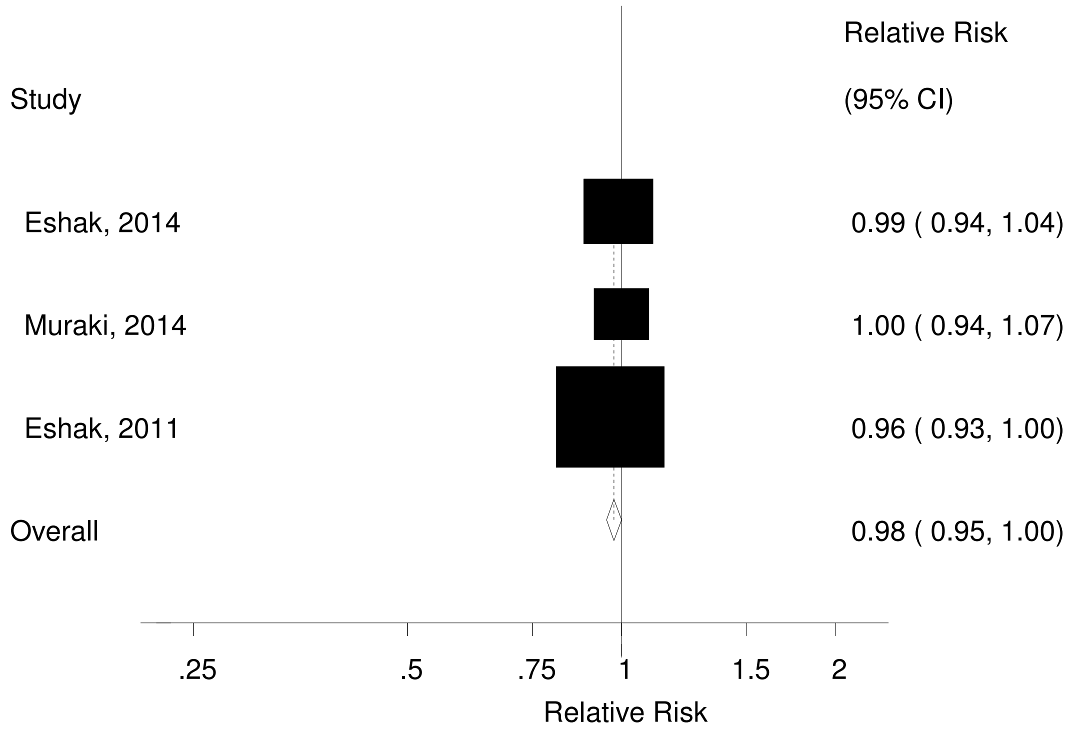

Figure S71. Total rice and cardiovascular disease, high vs. low

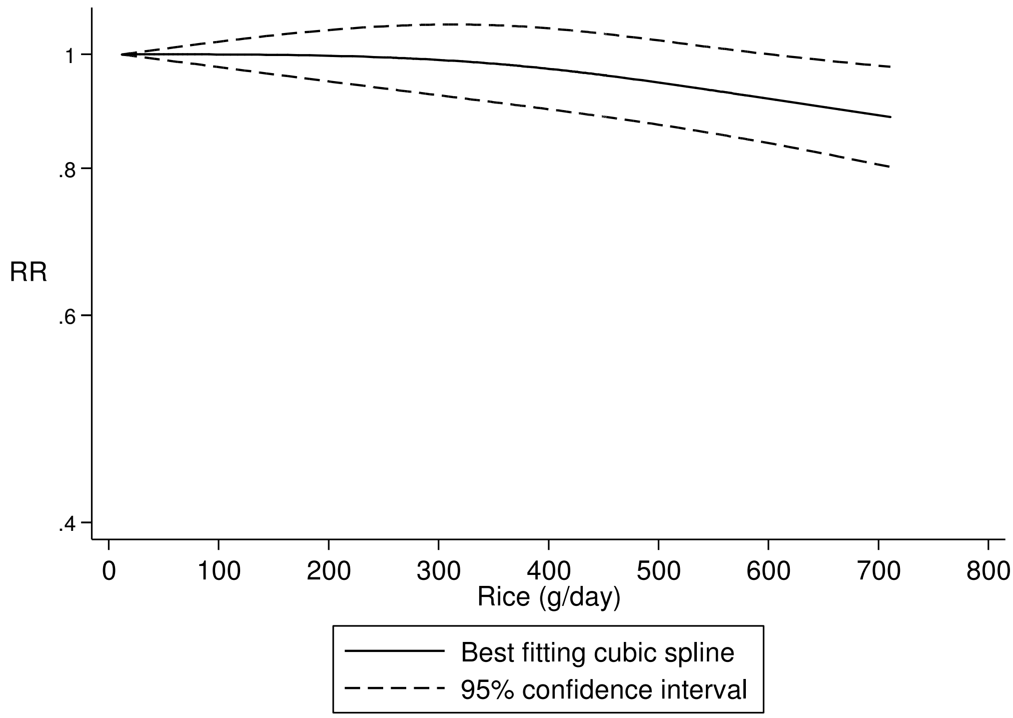

Figure S72. Total grains and cardiovascular disease, high vs. low

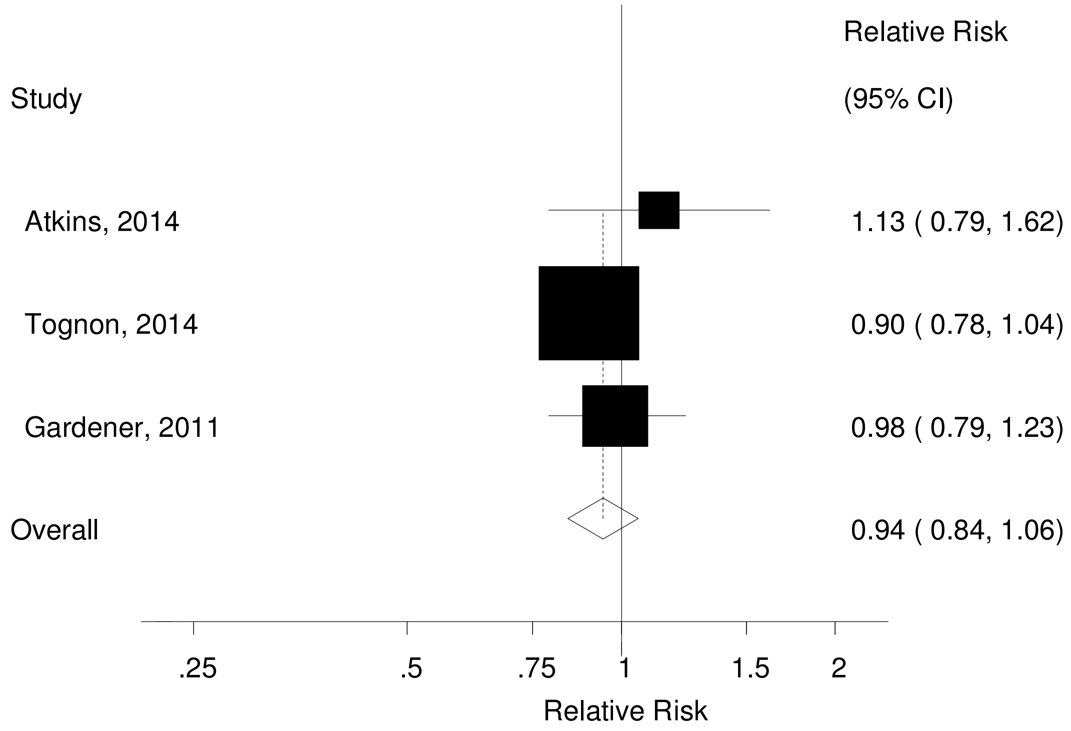

Figure S73. Whole grain bread and total cancer, high vs. low

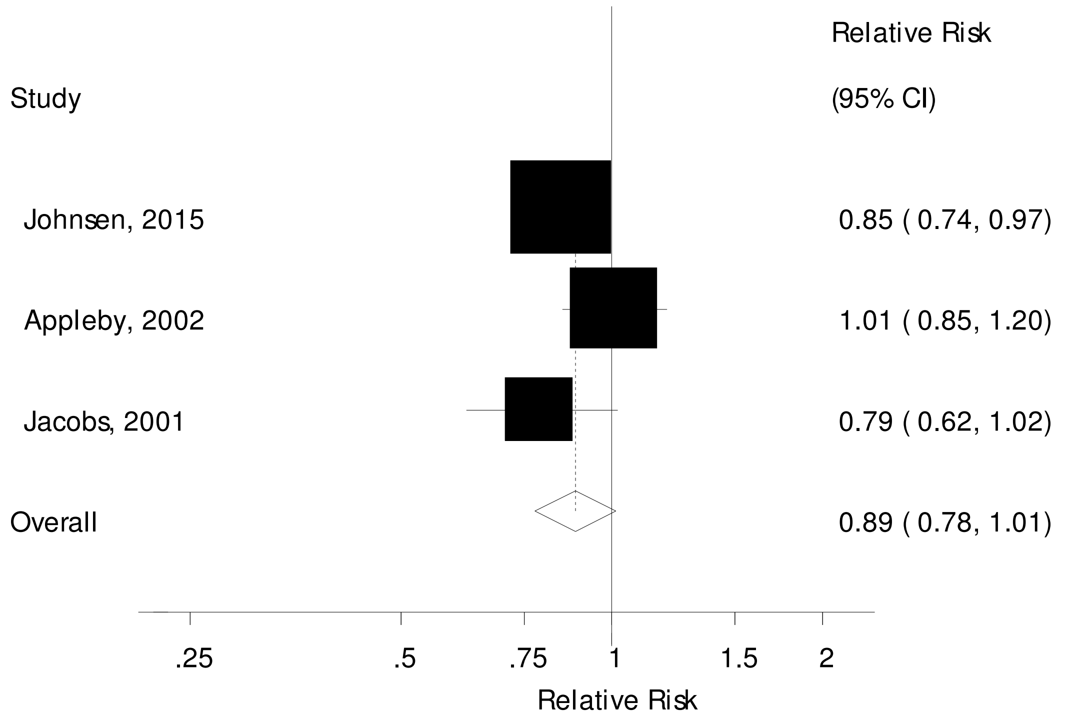

Figure S74. Whole grain bread and total cancer, per 90 g/d

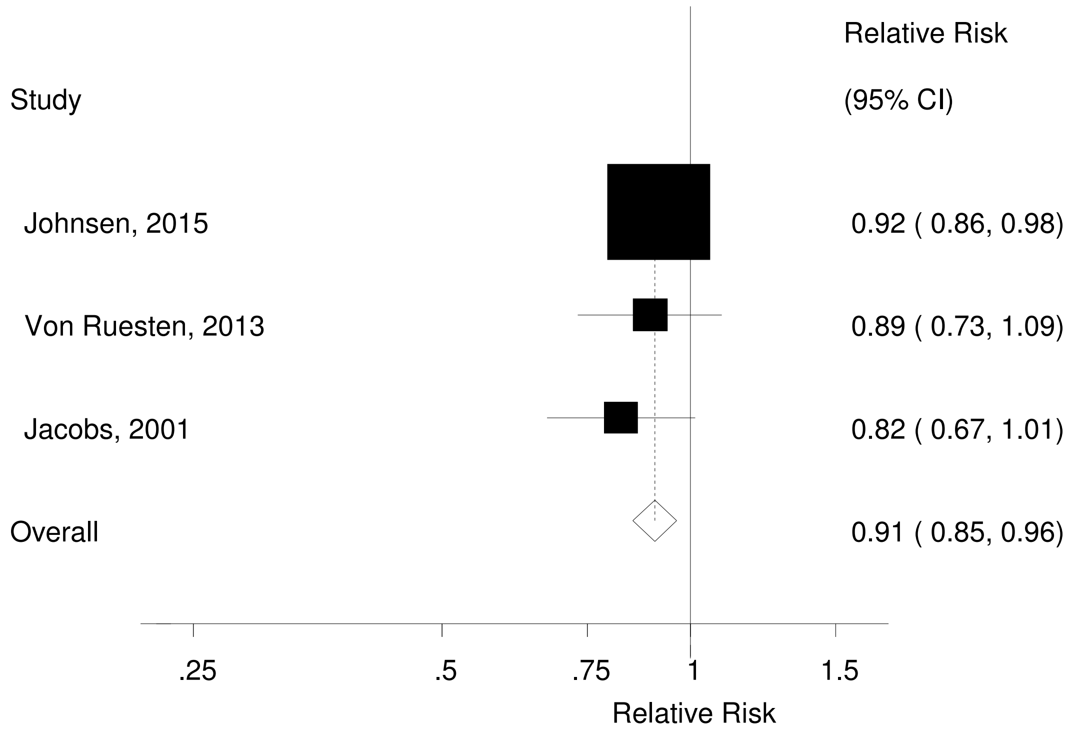

Figure S75. Brown rice and total cancer, high vs. low

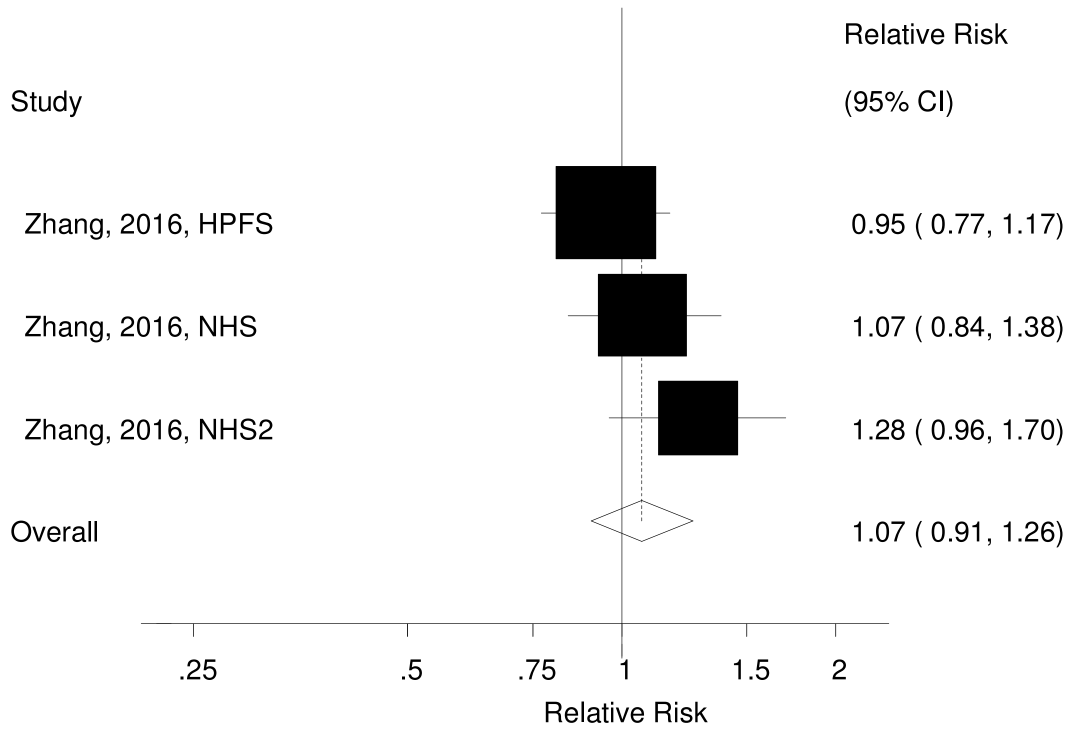

Figure S76. Brown rice and total cancer, per 100 g/d

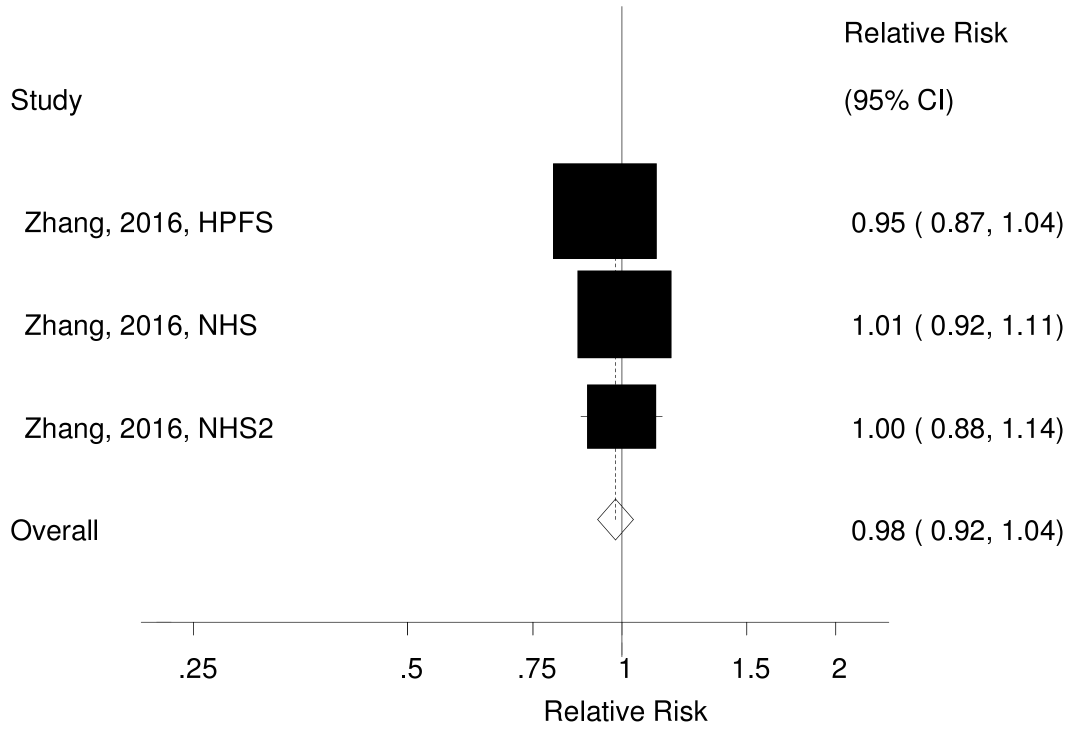

Figure S77. Brown rice and total cancer, nonlinear dose-response

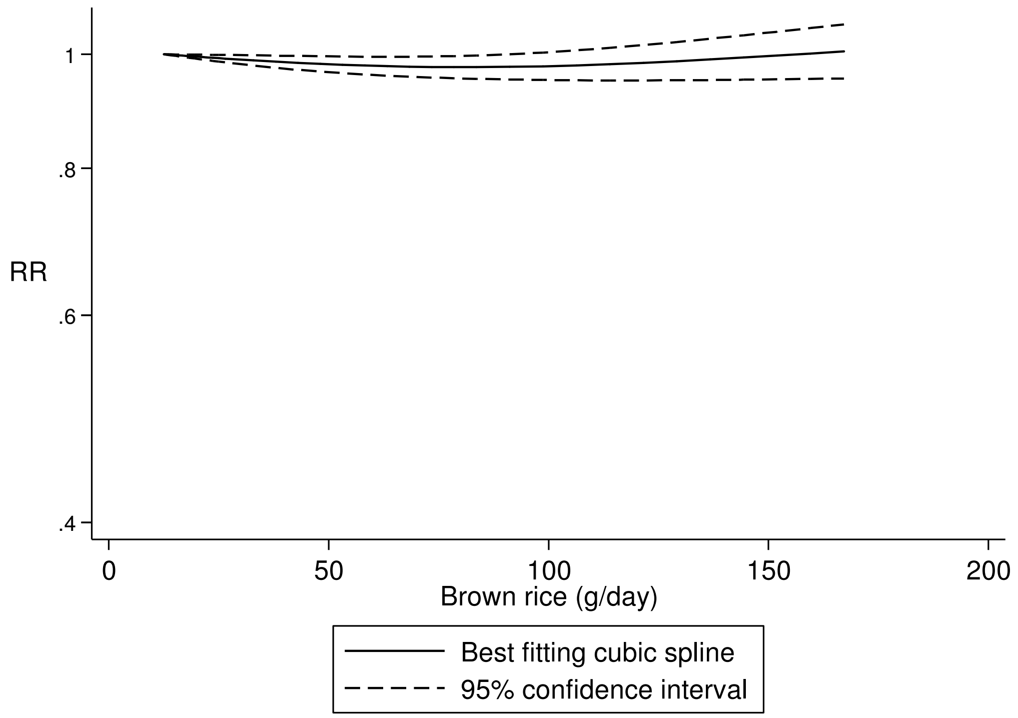

Figure S78. Refined grains and total cancer, per 90 g/d

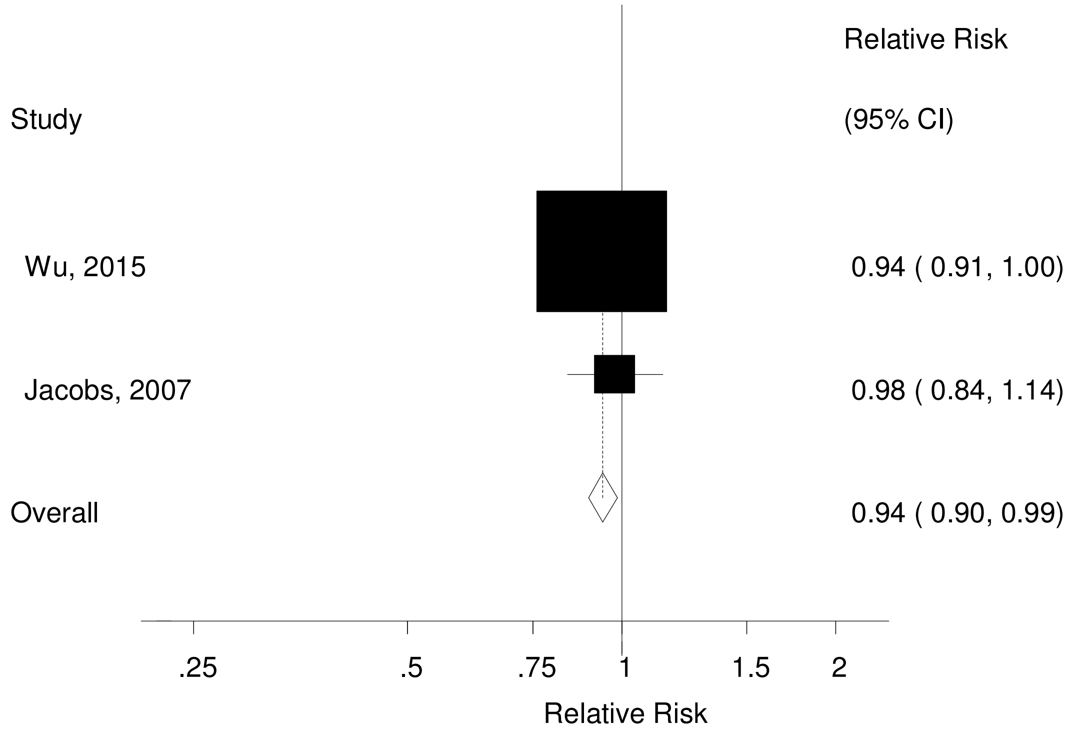

Figure S79. White rice and total cancer, high vs. low

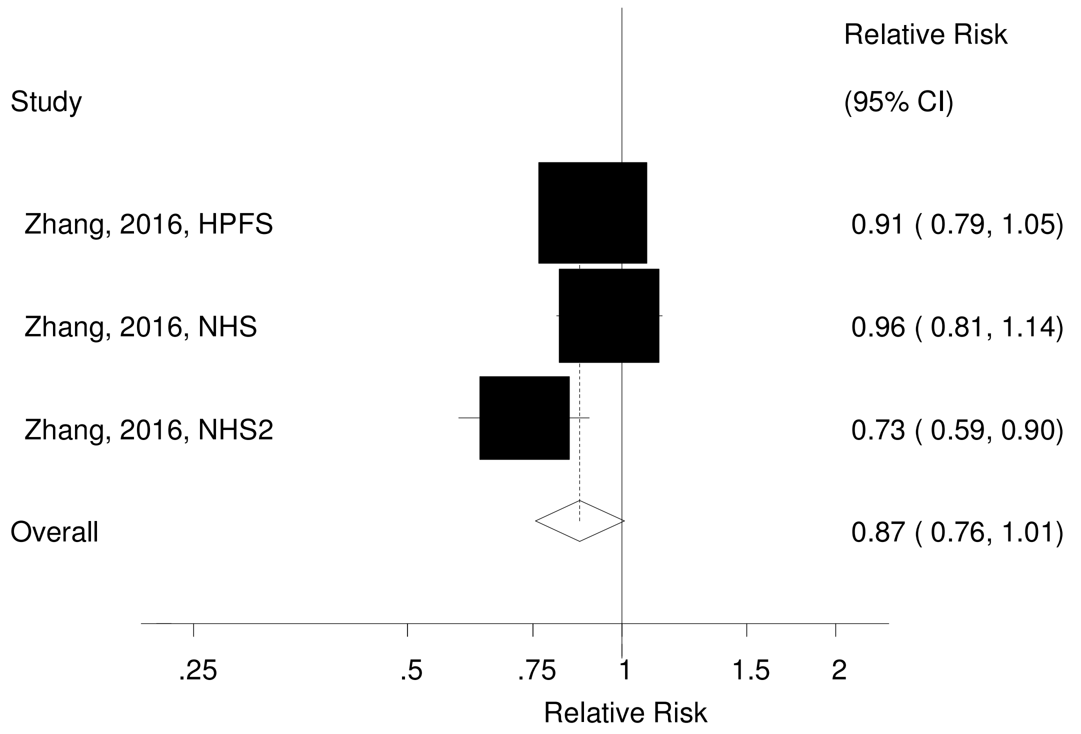

Figure S80. White rice and total cancer, per 100 g/d

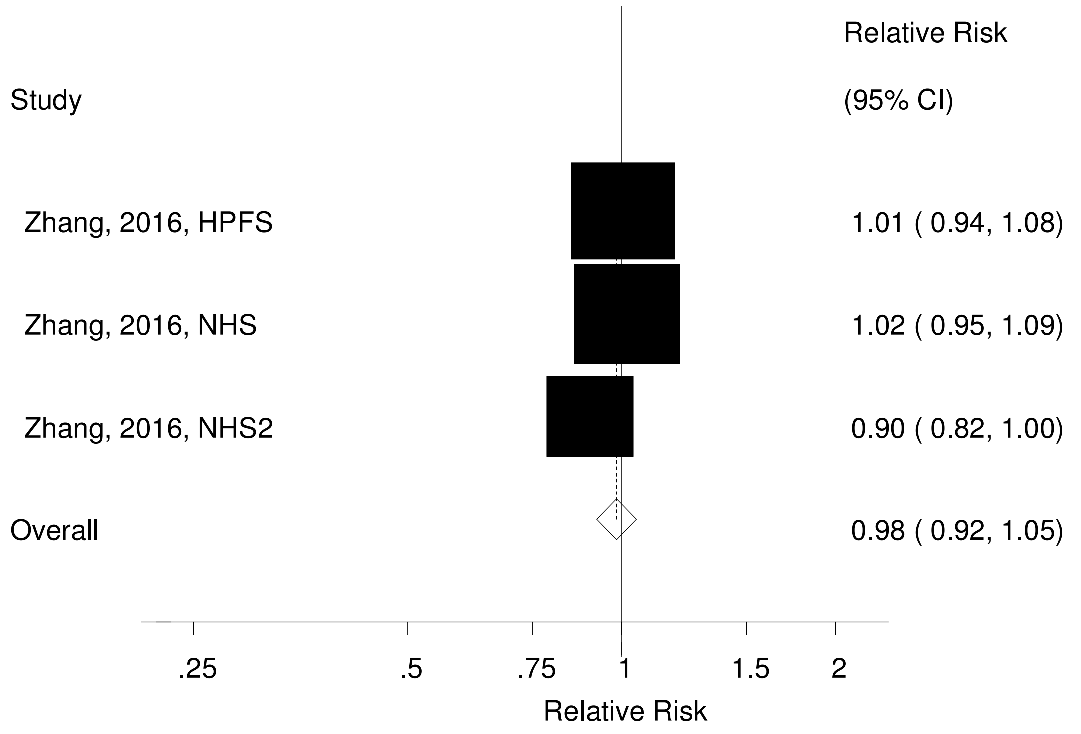

Figure S81. White rice and total cancer, nonlinear dose-response

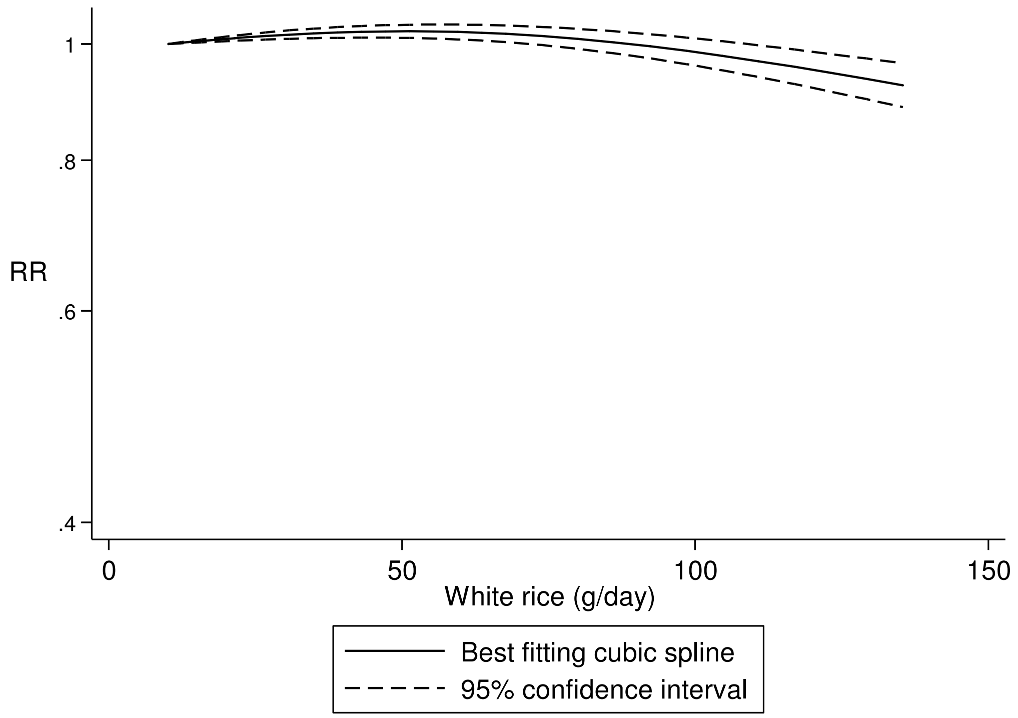

Figure S82. Total breakfast cereals and total cancer, high vs. low

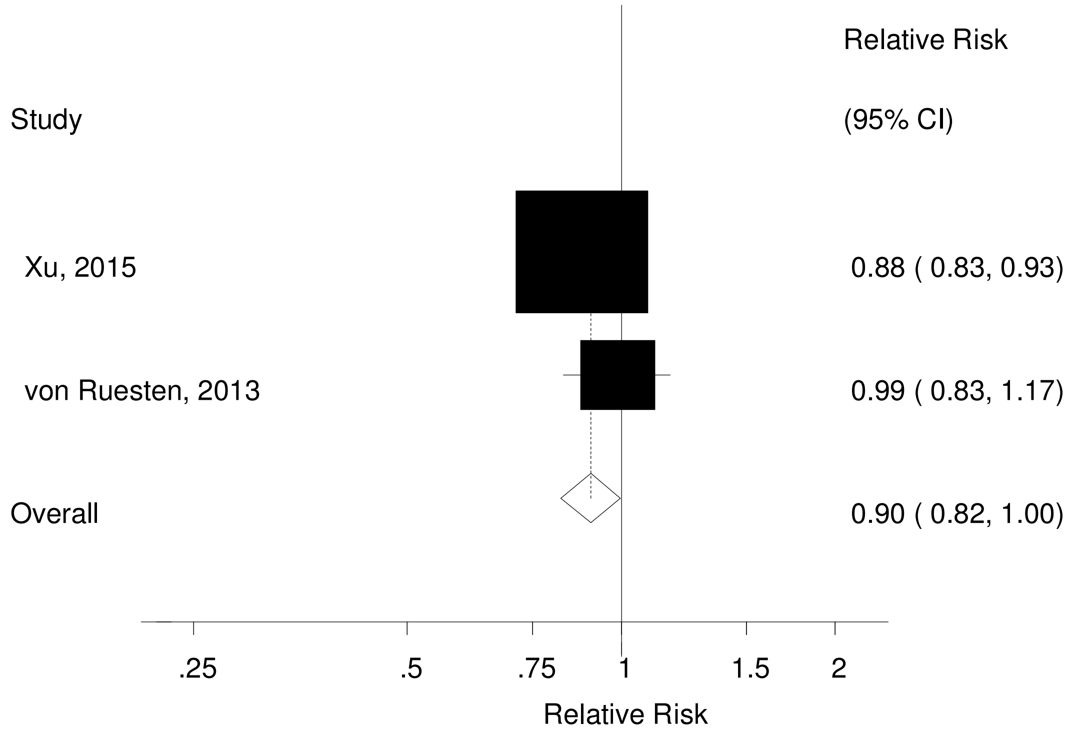

Figure S83. Total rice and total cancer, high vs. low

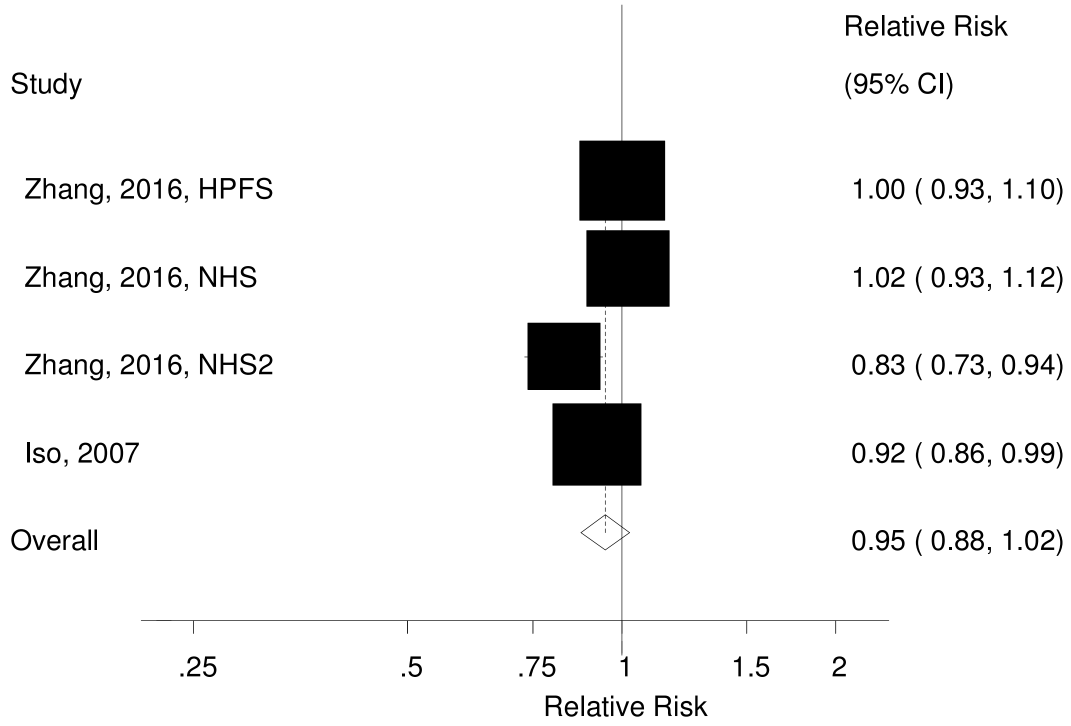

Figure S84. Total rice and total cancer, per 100 g/d

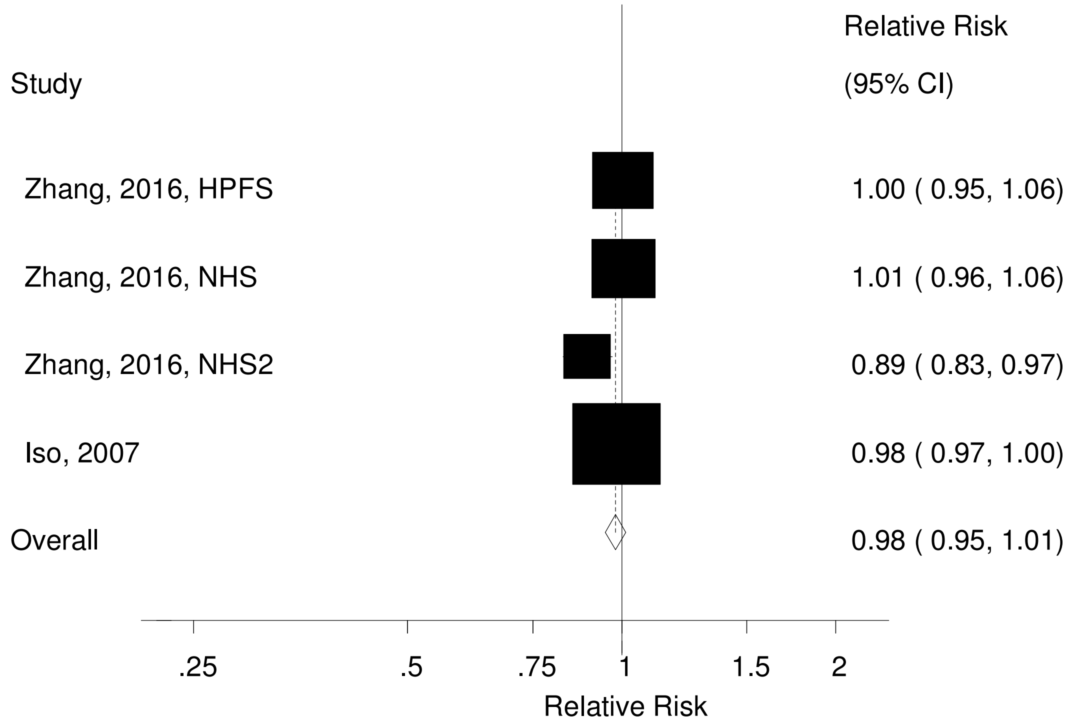

Figure S85. Total rice and total cancer, nonlinear dose-response

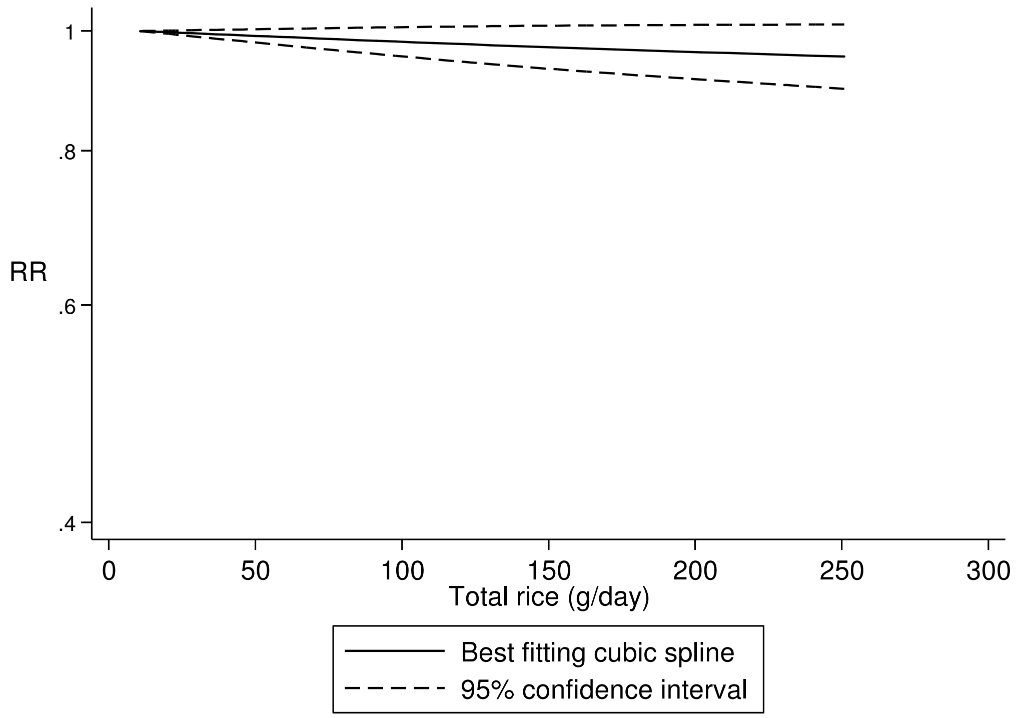

Figure S86. Total grains and total cancer, per 90 g/d

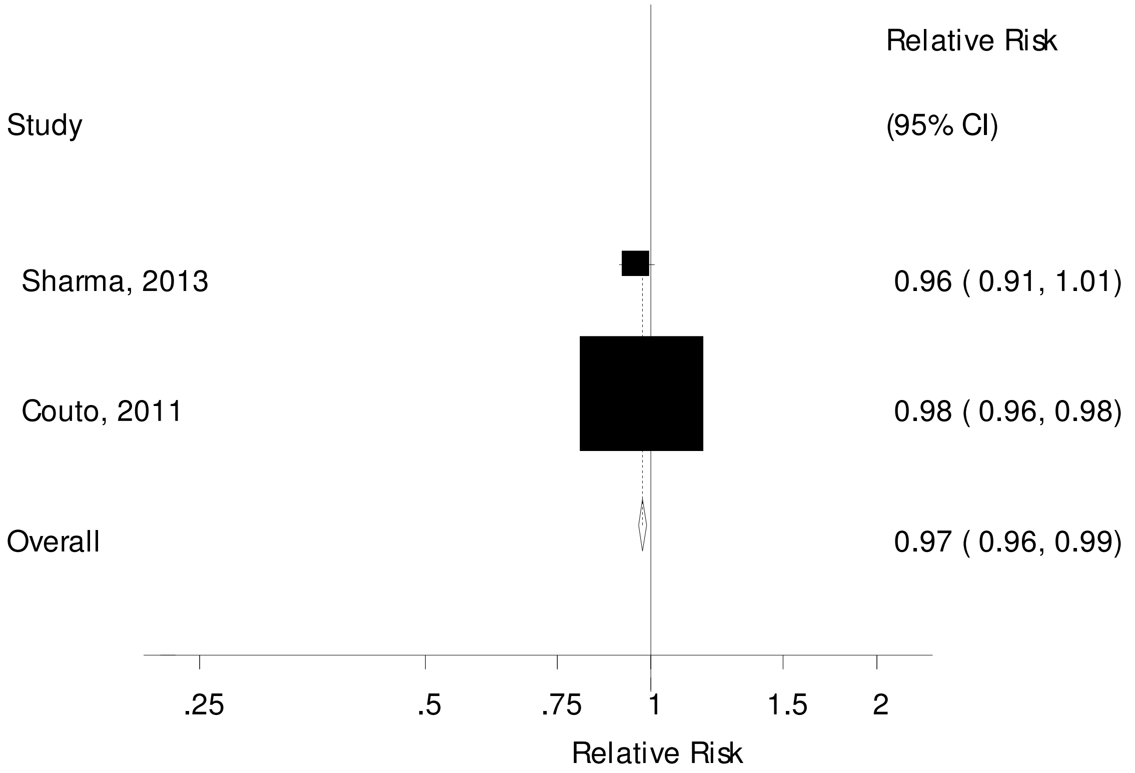

Figure S87. Whole grains bread and all-cause mortality, high vs. low

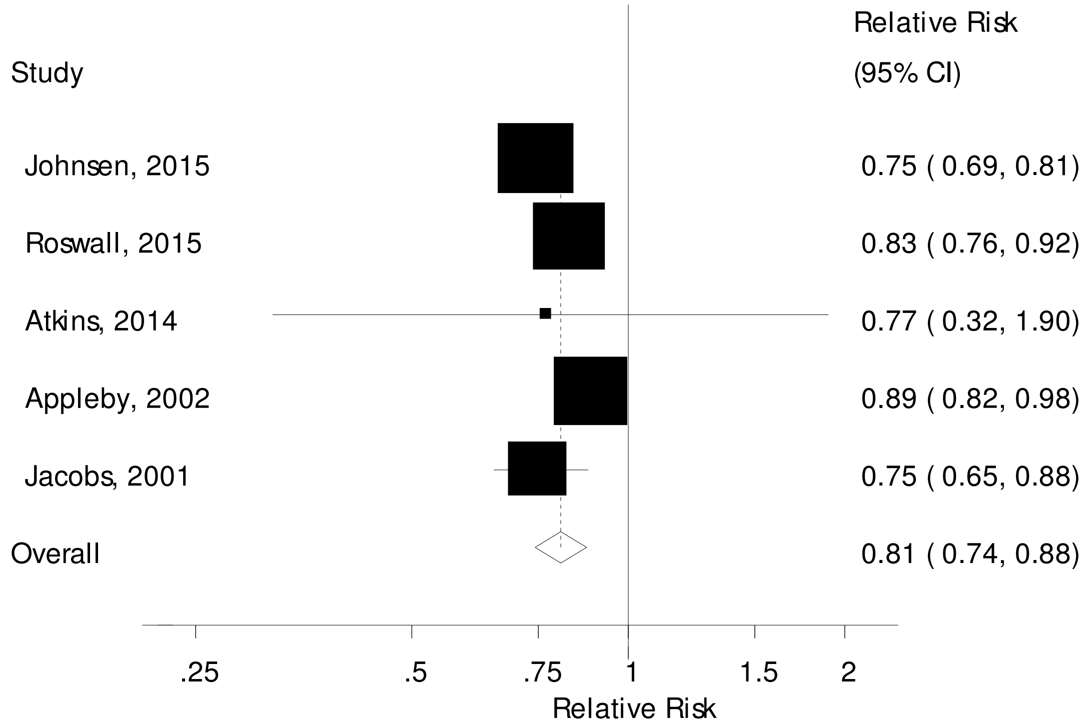

Figure S88. Whole grains bread and all-cause mortality, per 90 g/d

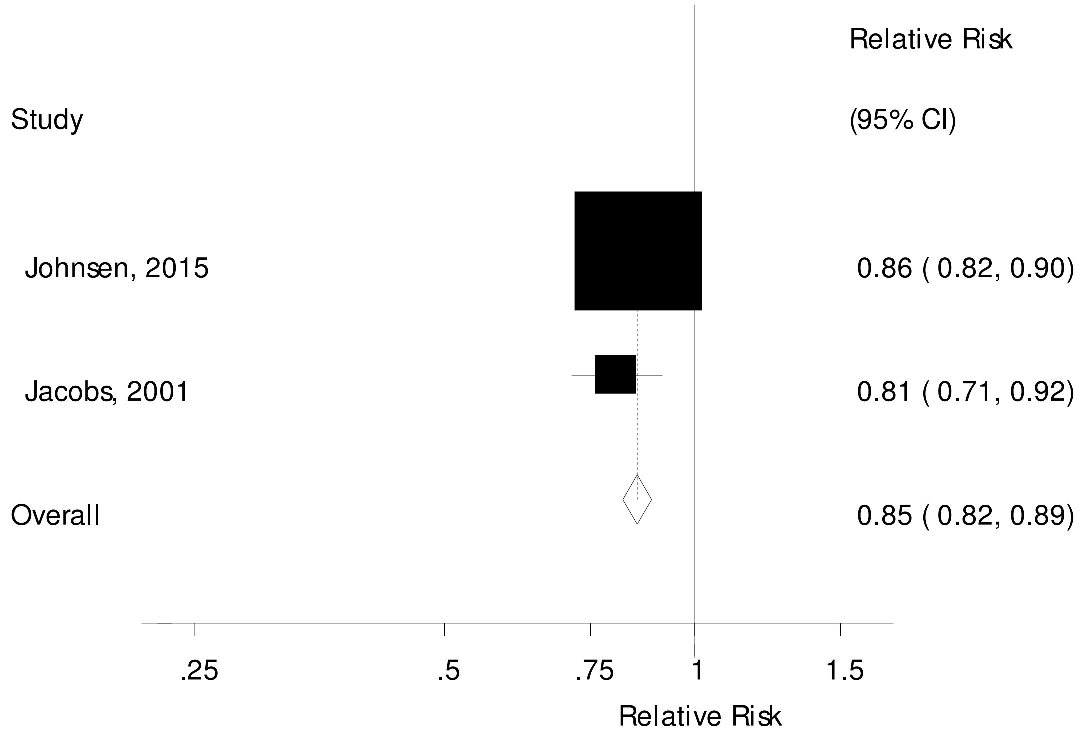

Figure S89. Whole grain breakfast cereals and all-cause mortality, high vs. low

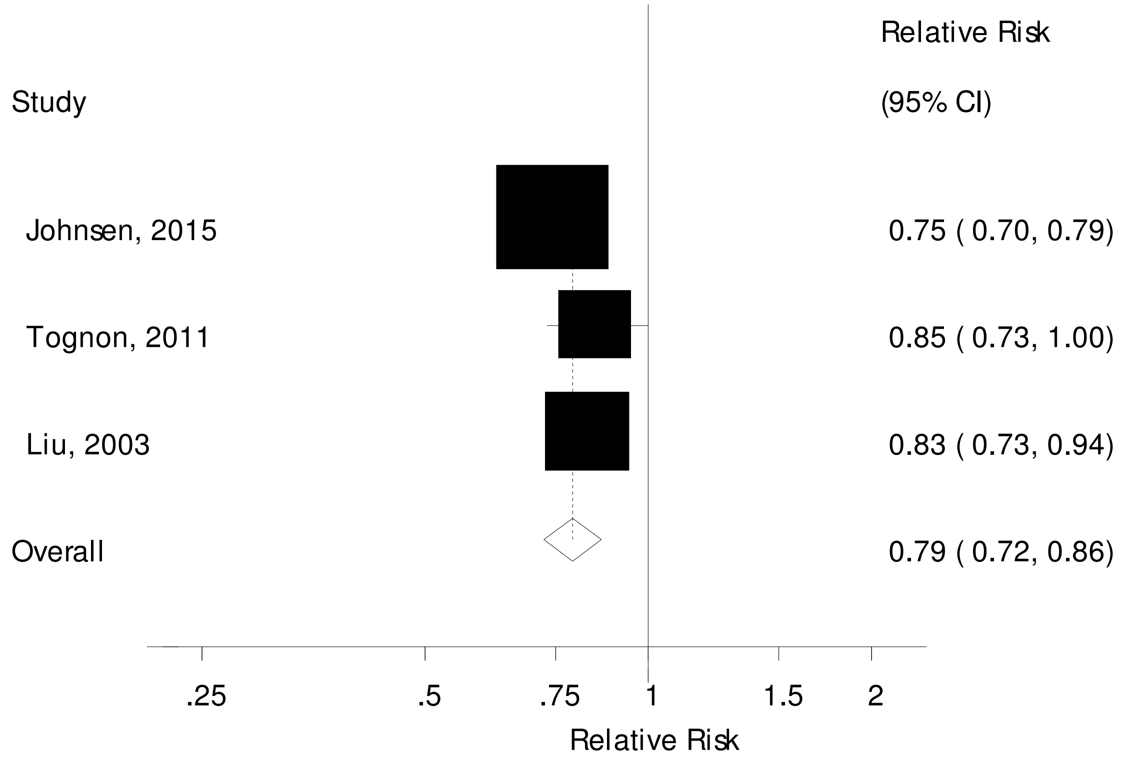

Figure S90. Whole grain breakfast cereals and all-cause mortality, per 1 serv/d

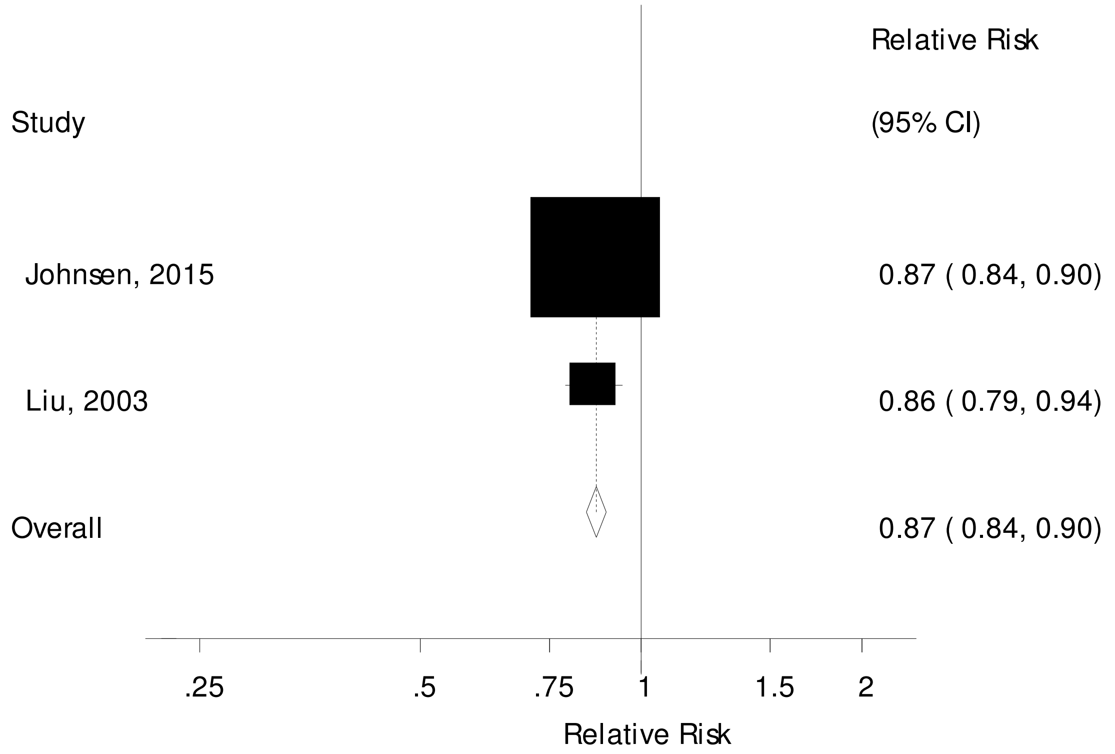

Figure S91. Oats or oatmeal and all-cause mortality, high vs. low

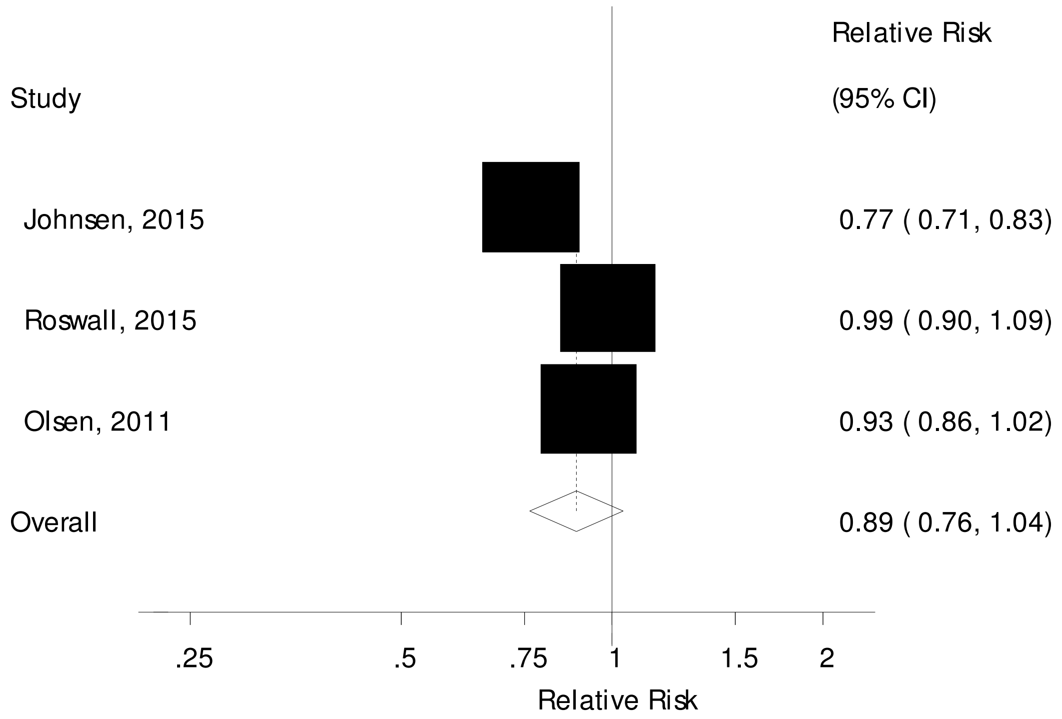

Figure S92. Refined grains and all-cause mortality, high vs. low

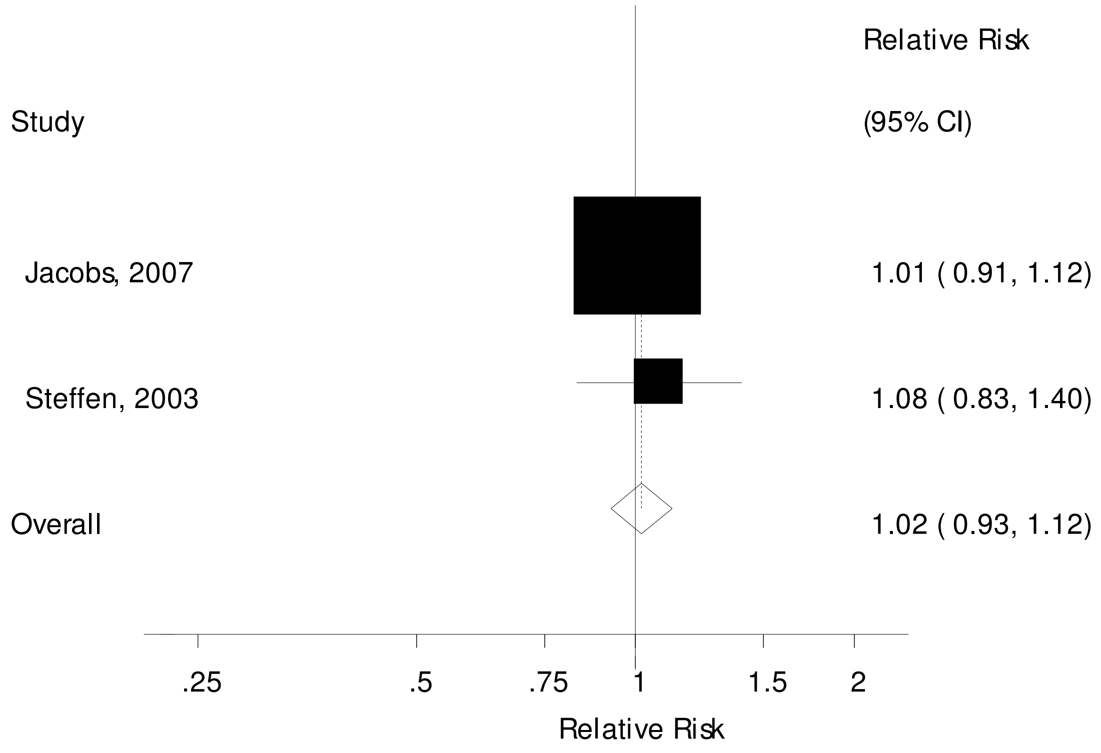

Figure S93. Refined grains and all-cause mortality, per 90 g/d

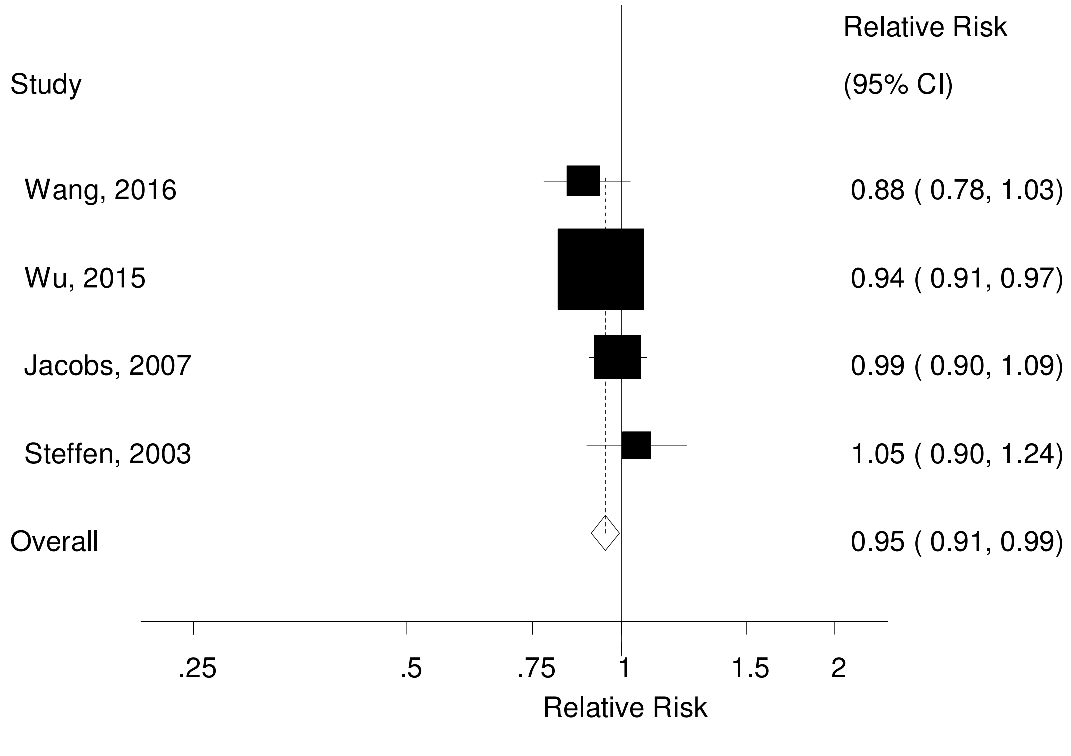

Figure S94. Pasta and all-cause mortality, per 150 g/d

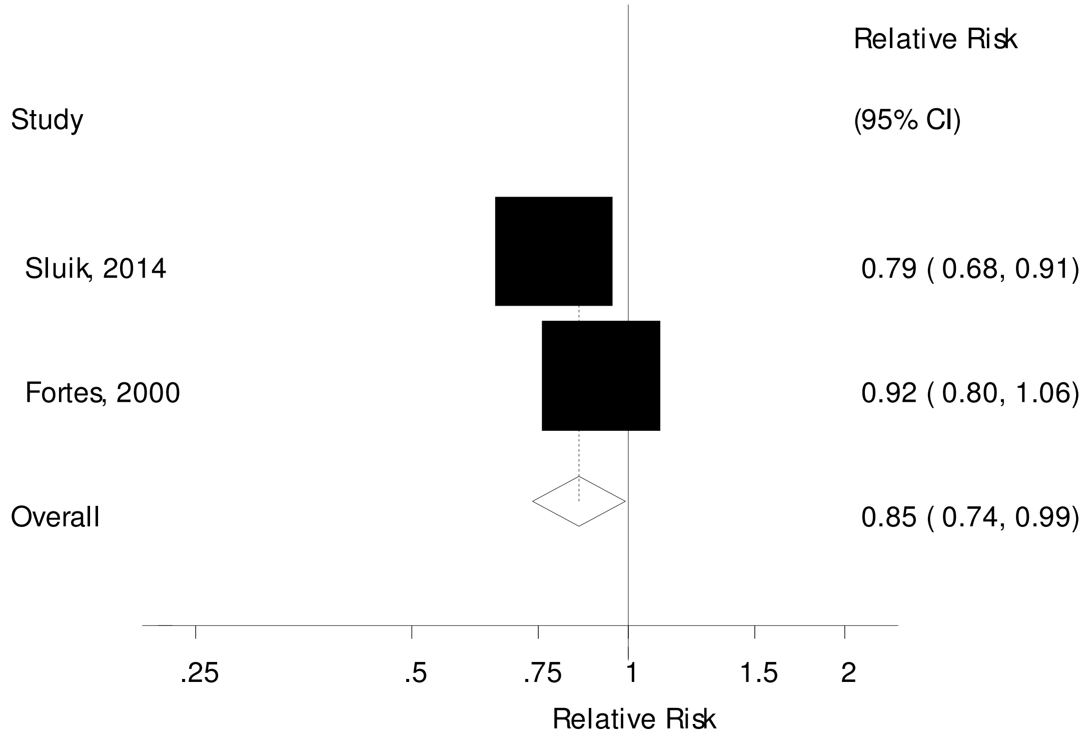

Figure S95. Total bread and all-cause mortality, high vs. low

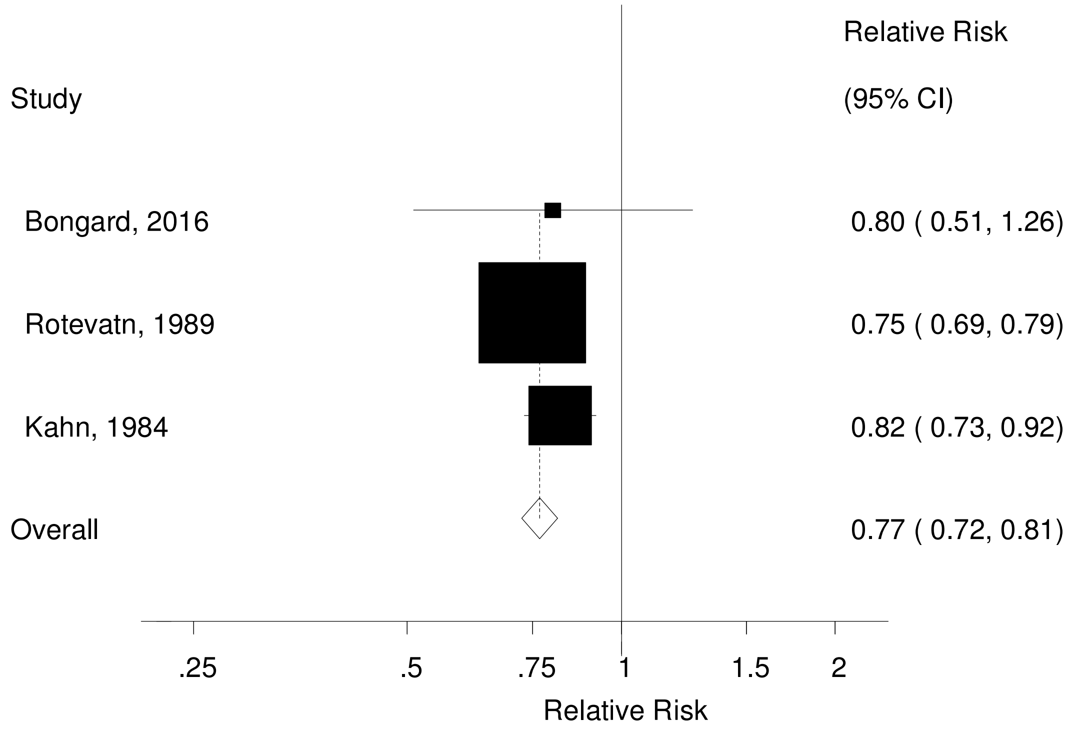

Figure S96. Total bread and all-cause mortality, per 90 g/d

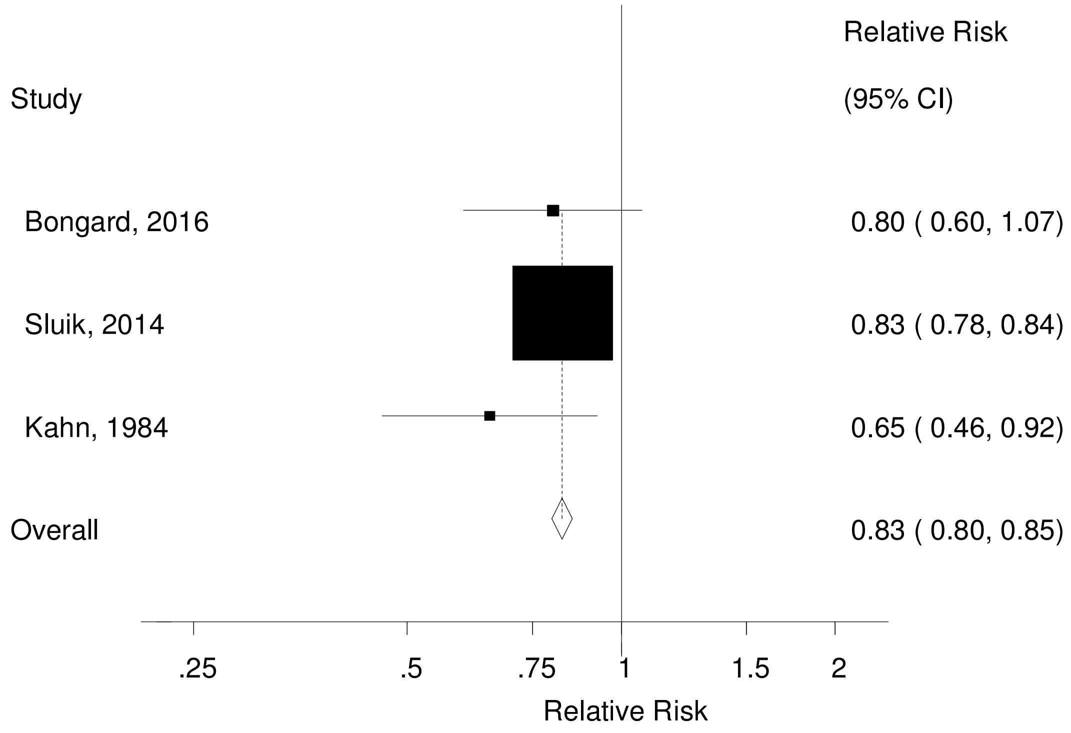

Figure S97. Total bread and all-cause mortality, nonlinear dose-response

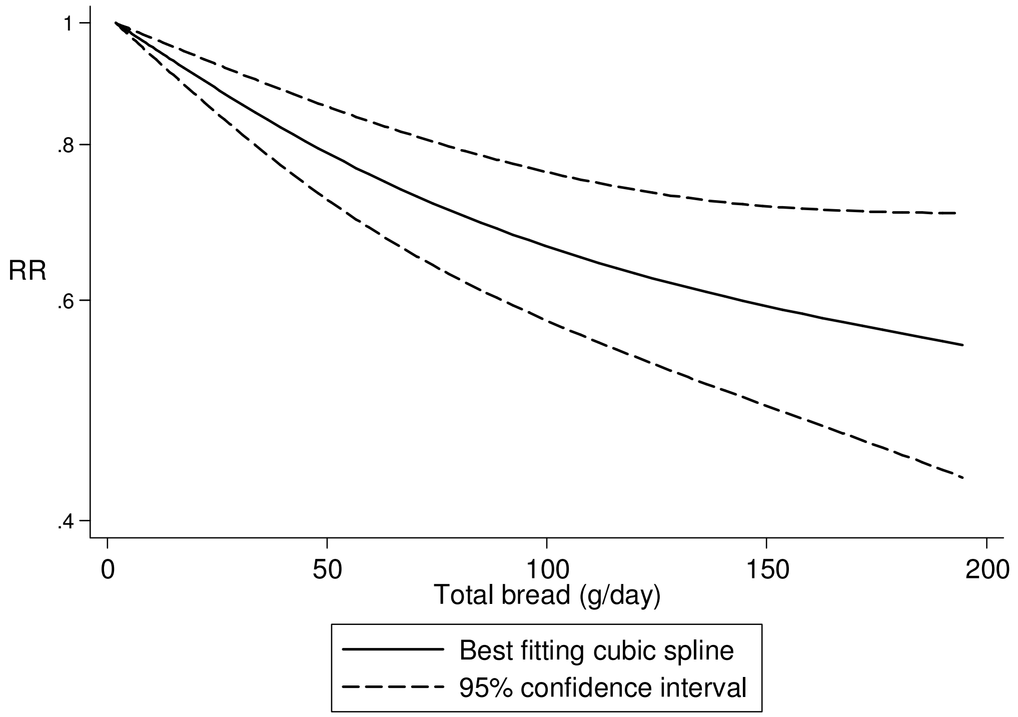

Figure S98. Total breakfast cereals and all-cause mortality, high vs. low

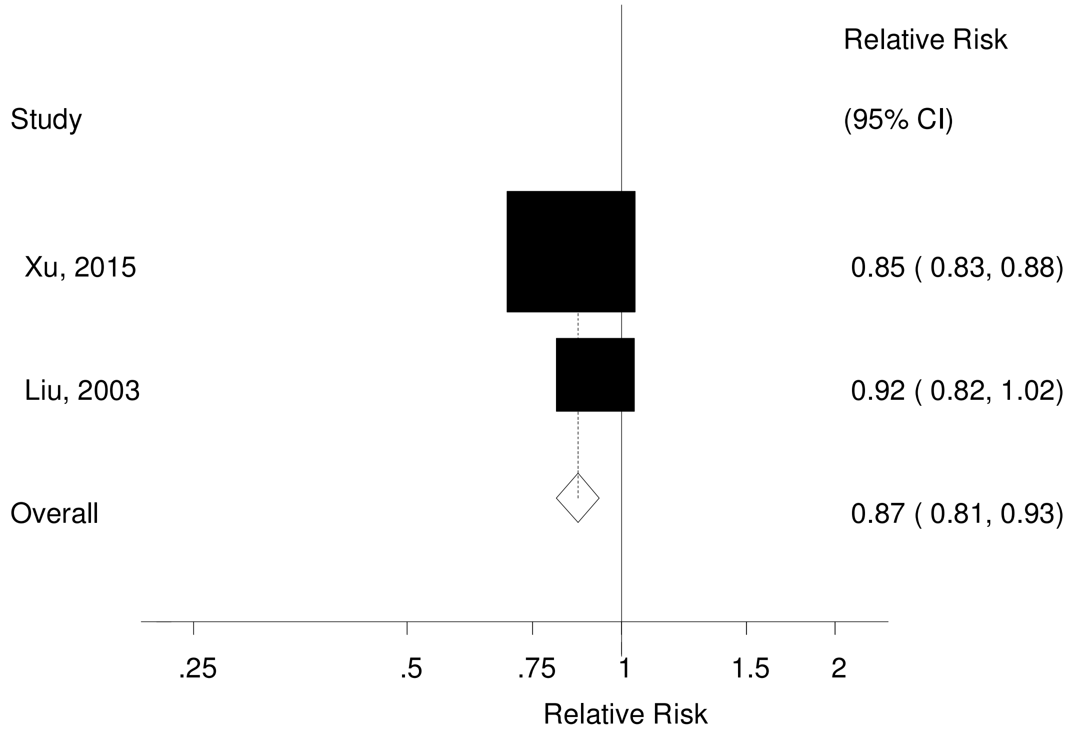

Figure S99. Total breakfast cereals and all-cause mortality, per 30 g/d

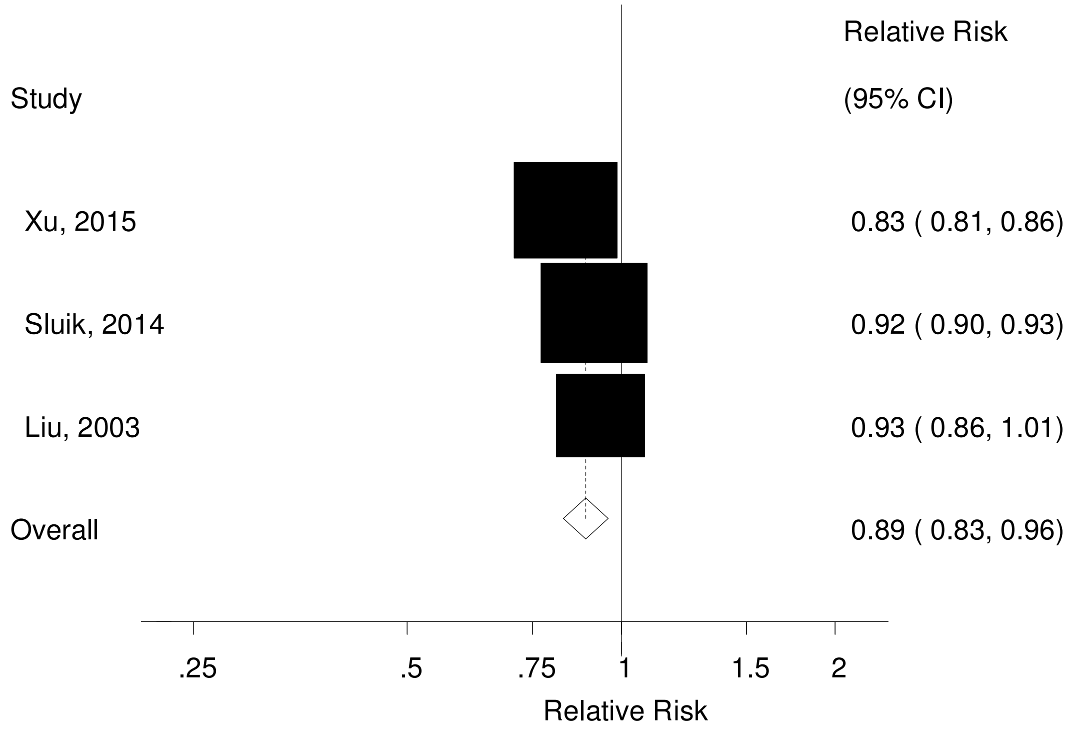

Figure S100. Total grains and all-cause mortality, high vs. low

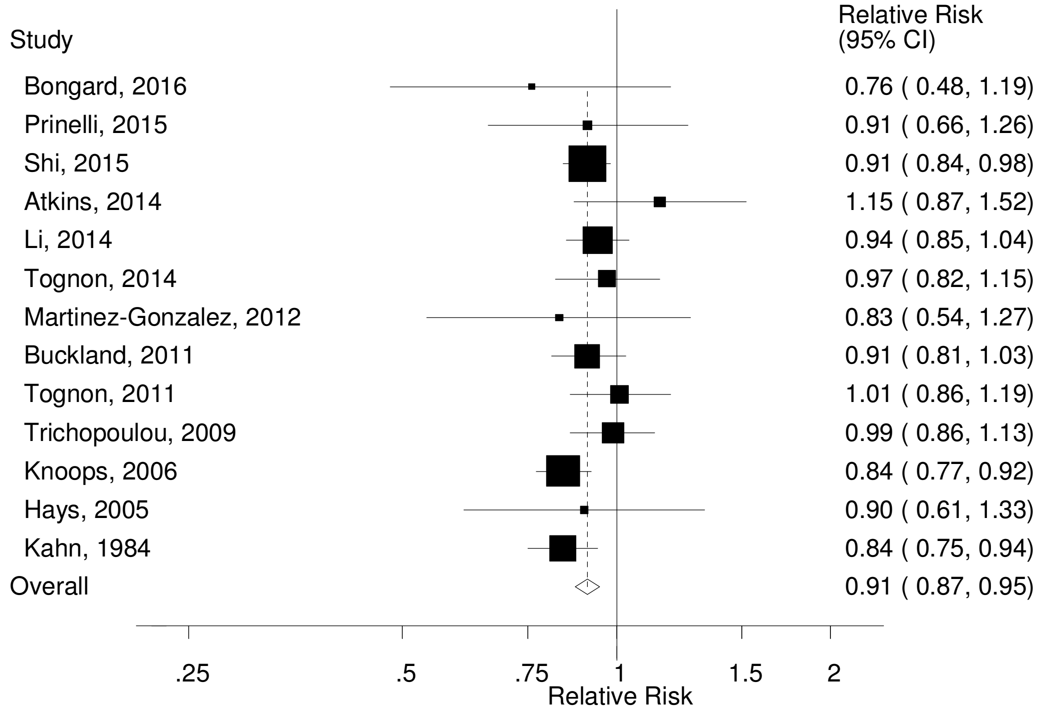

Figure S101. Total grains and all-cause mortality, per 90 g/d

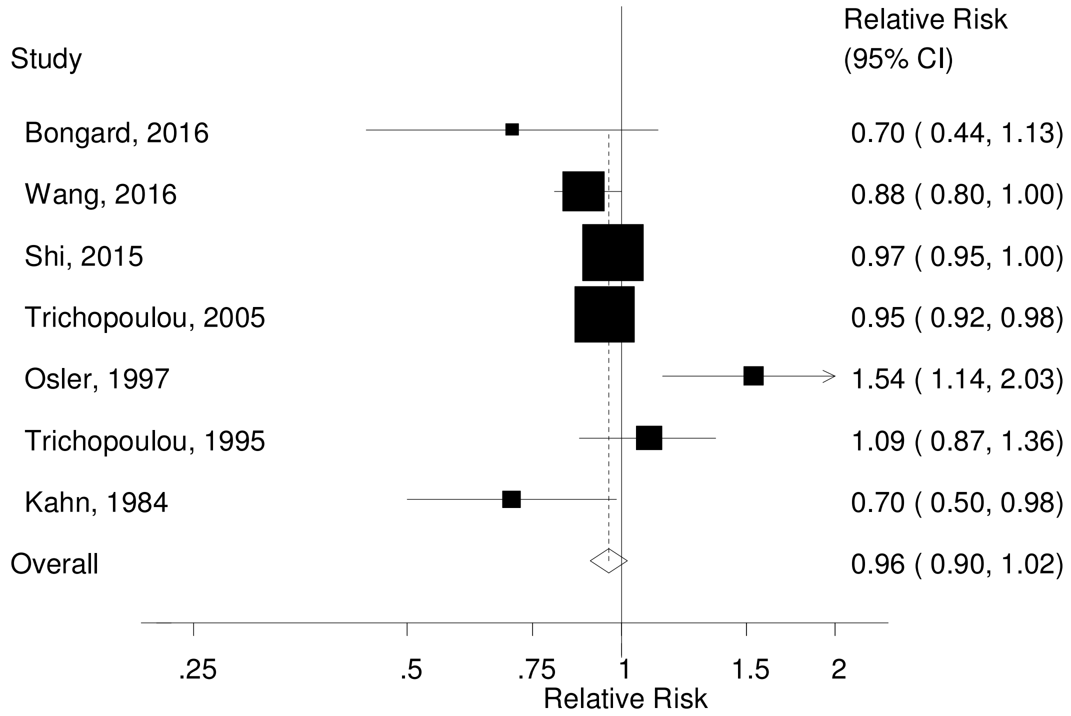

Figure S102. Total grains and all-cause mortality, nonlinear dose-response

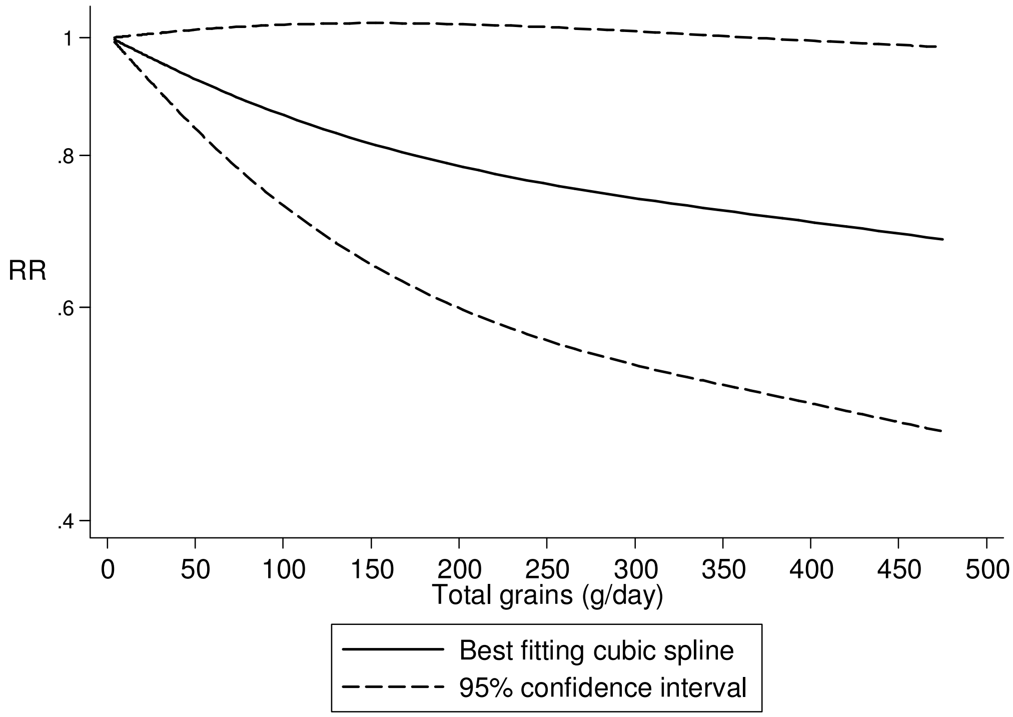

Figure S103. Funnel plot of whole grains and cardiovascular disease

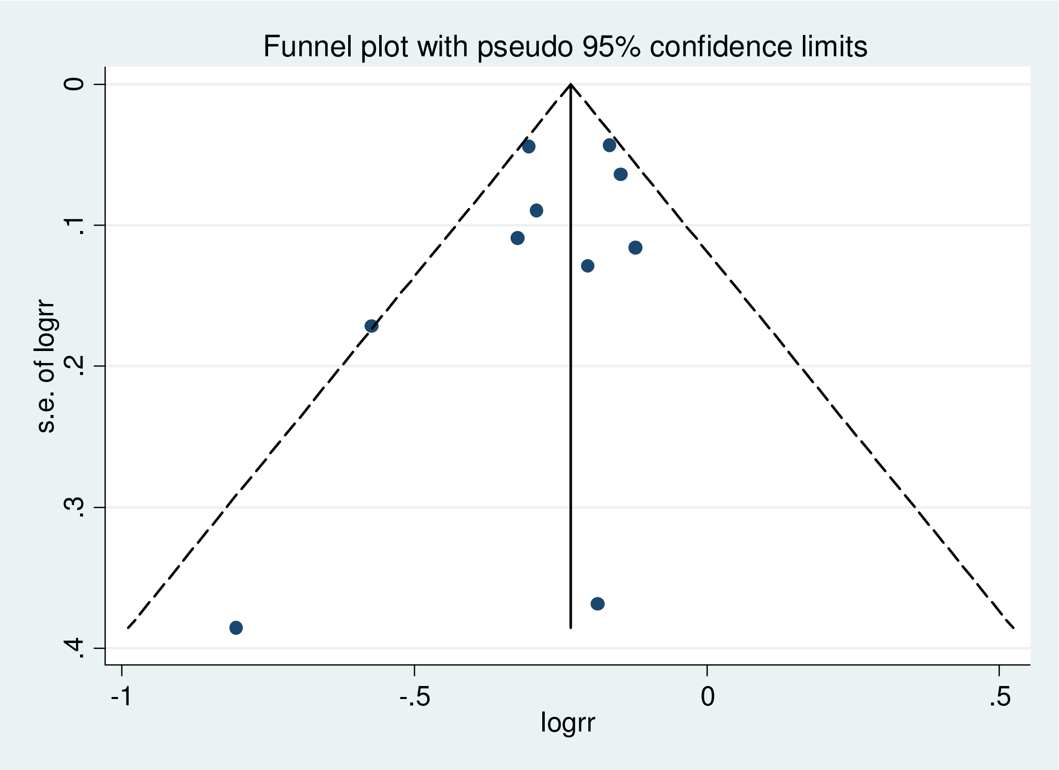

Figure S104. Funnel plot of whole grains and all-cause mortality

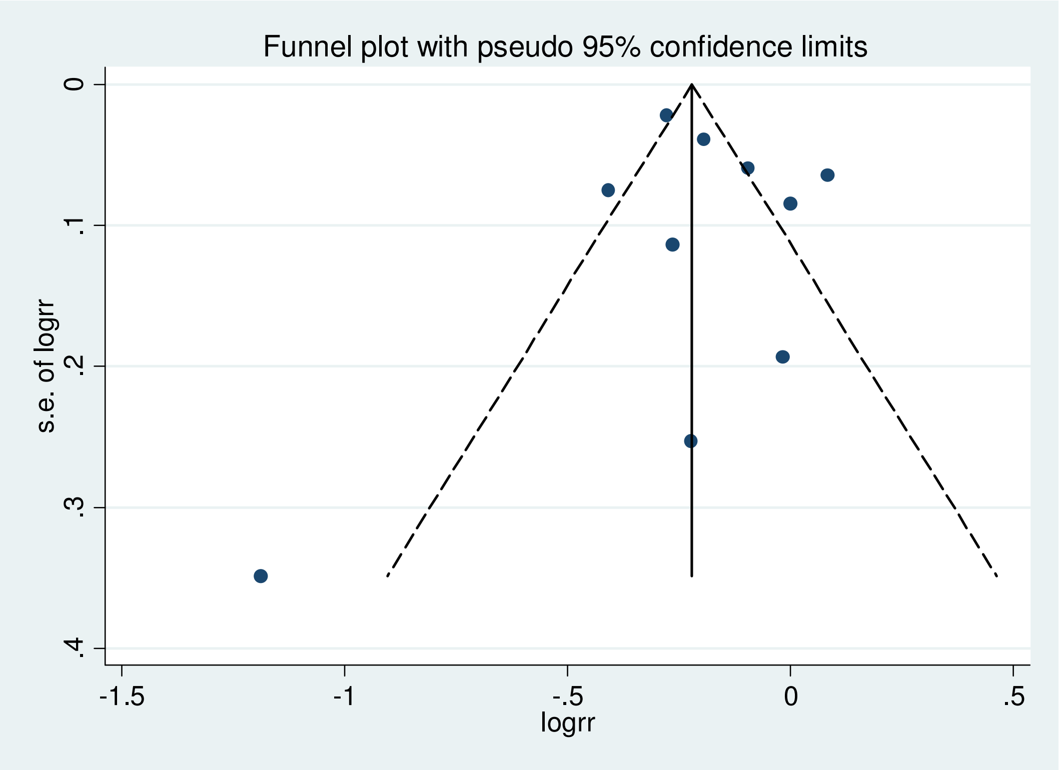

Figure S105. Influence analysis of whole grains and coronary heart disease

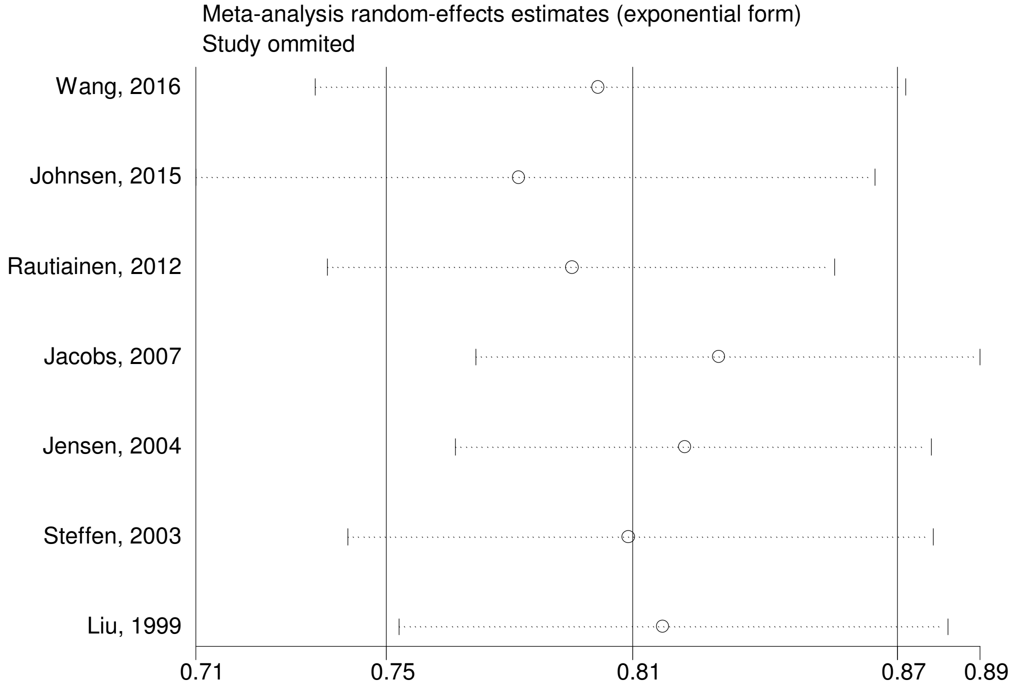

Study omitted	e^coef.	[95% Conf.	Interval]
Wang, 2016	0.80325192	0.73750728	0.87485725
Johnsen, 2015	0.78475595	0.70969582	0.8677547
Rautiainen, 2012	0.79712176	0.74025631	0.85835552
Jacobs, 2007	0.83140707	0.77479708	0.89215332
Jensen, 2004	0.82358283	0.77009189	0.88078934
Steffen, 2003	0.81029981	0.74506956	0.88124096
Liu, 1999	0.81833088	0.75697881	0.88465548
Combined	0.81134143	0.75406713	0.87296592

Figure S106. Influence analysis of whole grains and stroke

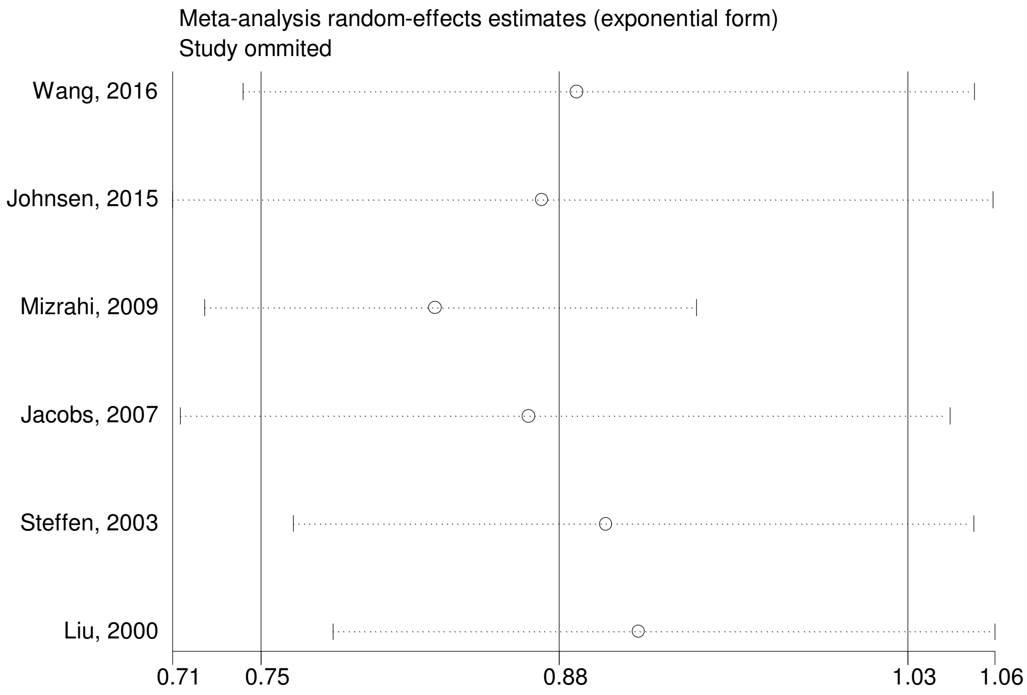

Study omitted	e^coef.	[95% Conf.	Interval]
Wang, 2016	0.88287657	0.73981452	1.0536033
Johnsen, 2015	0.86779642	0.7094658	1.0614616
Mizrahi, 2009	0.82212949	0.72333467	0.93441796
Jacobs, 2007	0.86227262	0.71278179	1.043116
Steffen, 2003	0.89551038	0.76146871	1.0531474
Liu, 2000	0.90933239	0.77837229	1.0623263
Combined	0.87530632	0.74741362	1.0250832

Figure S107. Influence analysis of whole grains and cardiovascular disease

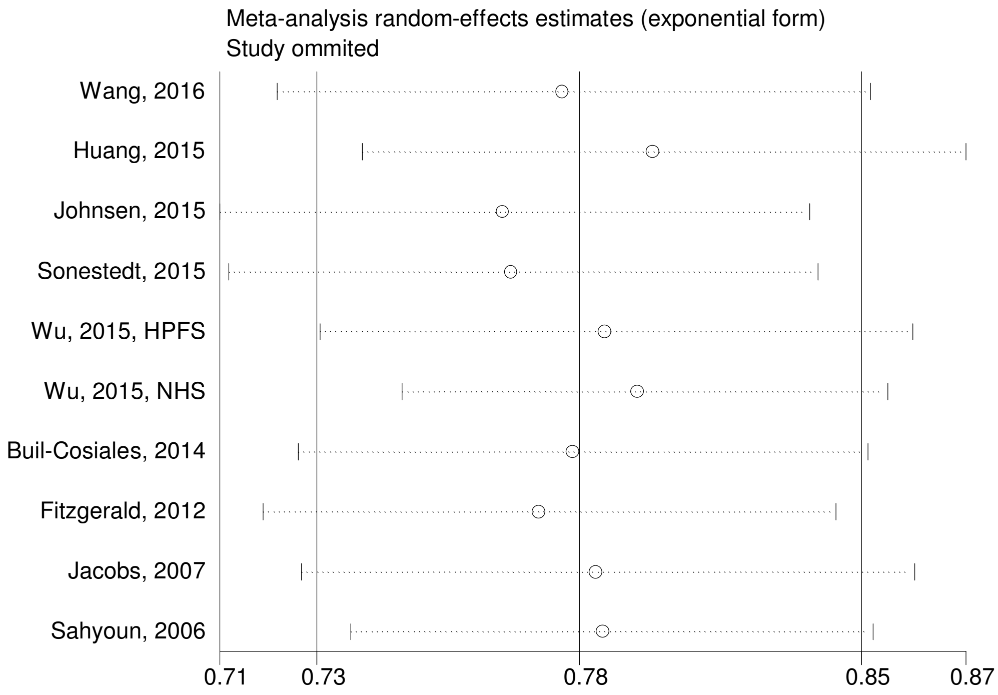

Study omitted	e^coef.	[95% Conf.	Interval]
Wang, 2016	0.78125685	0.72075218	0.84684068
Huang, 2015	0.80042845	0.7388069	0.86718959
Johnsen, 2015	0.76861632	0.70844555	0.83389759
Sonestedt, 2015	0.77045089	0.71031886	0.83567339
Wu, 2015, HPFS	0.7903021	0.72978294	0.85584003
Wu, 2015, NHS	0.79728162	0.74731356	0.85059083
Buil-Cosiales, 2014	0.78345406	0.72522616	0.84635699
Fitzgerald, 2012	0.7762832	0.7177375	0.83960456
Jacobs, 2007	0.78840691	0.72586286	0.85634005
Sahyoun, 2006	0.78992122	0.73630428	0.84744245
Combined	0.78494631	0.72913752	0.84502676

Figure S108. Influence analysis of whole grains and total cancer

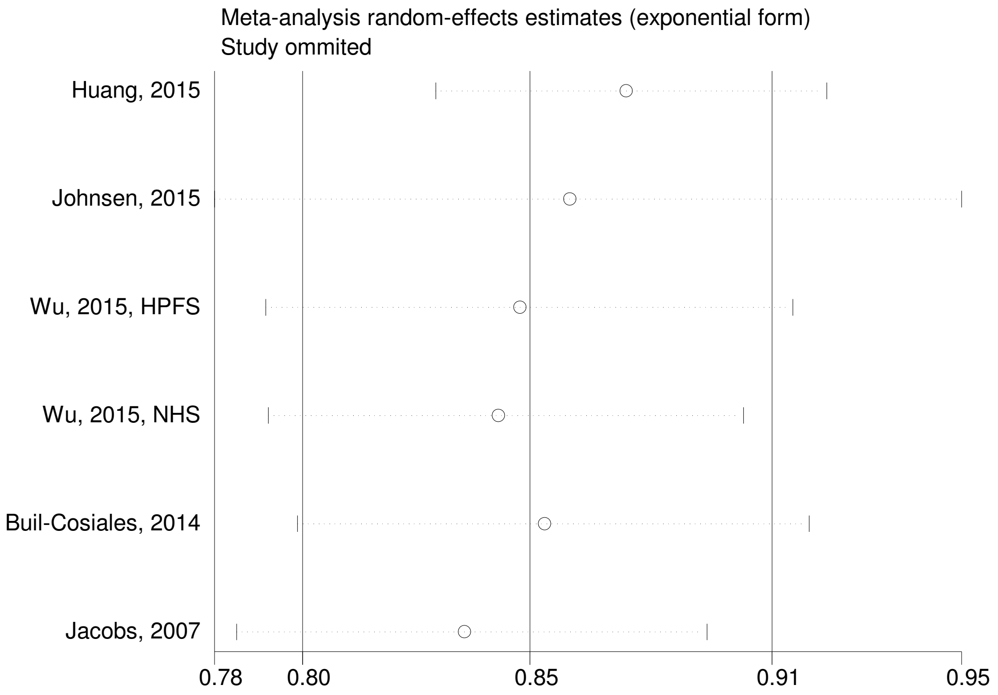

Study omitted	e^coef.	[95% Conf.	Interval]
Huang, 2015	0.87488538	0.83192068	0.9200691
Johnsen, 2015	0.86217254	0.78203869	0.95051759
Wu, 2015, HPFS	0.85095882	0.7936067	0.91245568
Wu, 2015, NHS	0.84606177	0.79419458	0.90131629
Buil-Cosiales, 2014	0.85649318	0.80077398	0.91608936
Jacobs, 2007	0.83840358	0.78703421	0.89312589
Combined	0.85318668	0.80188278	0.90777297

Figure S109. Influence analysis of whole grains and all-cause mortality

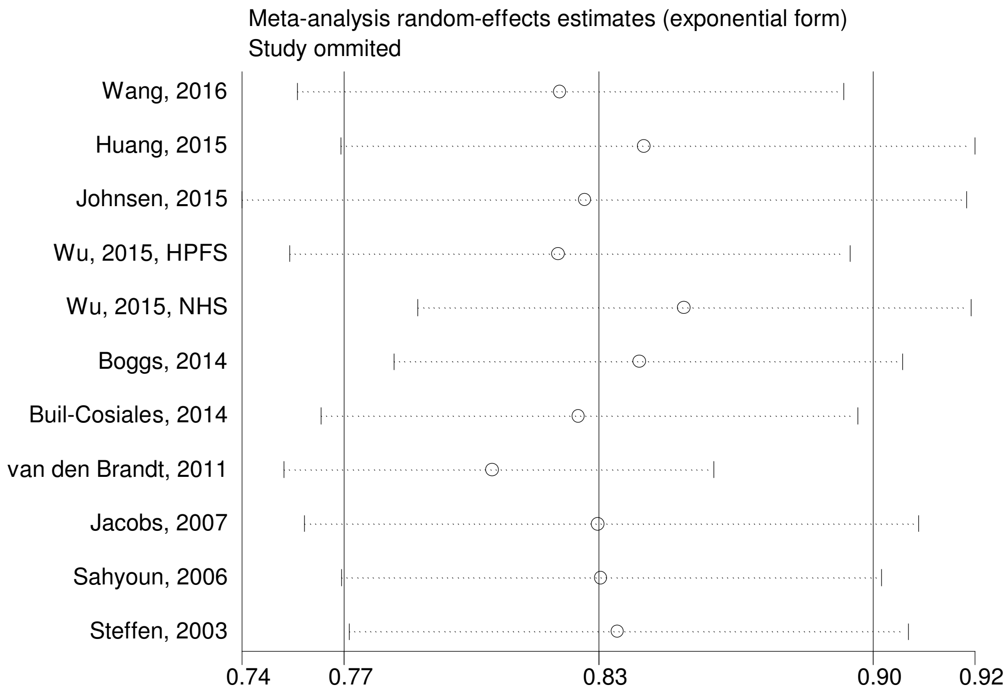

Study omitted	e^coef.	[95% Conf.	Interval]
Wang, 2016	0.82020313	0.75534874	0.89062589
Huang, 2015	0.84093648	0.76616013	0.92301089
Johnsen, 2015	0.82641751	0.74155837	0.92098737
Wu, 2015, HPFS	0.81984508	0.75342447	0.8921212
Wu, 2015, NHS	0.8509171	0.78520554	0.92212784
Boggs, 2014	0.83985263	0.77924085	0.90517902
Buil-Cosiales, 2014	0.8249197	0.76118785	0.8939876
van den Brandt, 2011	0.80349523	0.75208527	0.85841936
Jacobs, 2007	0.82963741	0.75716662	0.90904456
Sahyoun, 2006	0.83042234	0.76625842	0.89995903
Steffen, 2003	0.83451754	0.76816779	0.90659827
Combined	0.82986897	0.76699136	0.89790125
